# Supplementary material for: Nickel-catalysed retro-hydroamidocarbonylation of aliphatic amides to olefins
Source: Nat Commun. 2017 May 5;8:14993. doi: 10.1038/ncomms14993 (PMC5424121; doi:10.1038/ncomms14993)
Supplement: Supplementary Information — Supplementary figures, supplementary methods and supplementary references. [file ncomms14993-s1.pdf]

Supplementary Figures

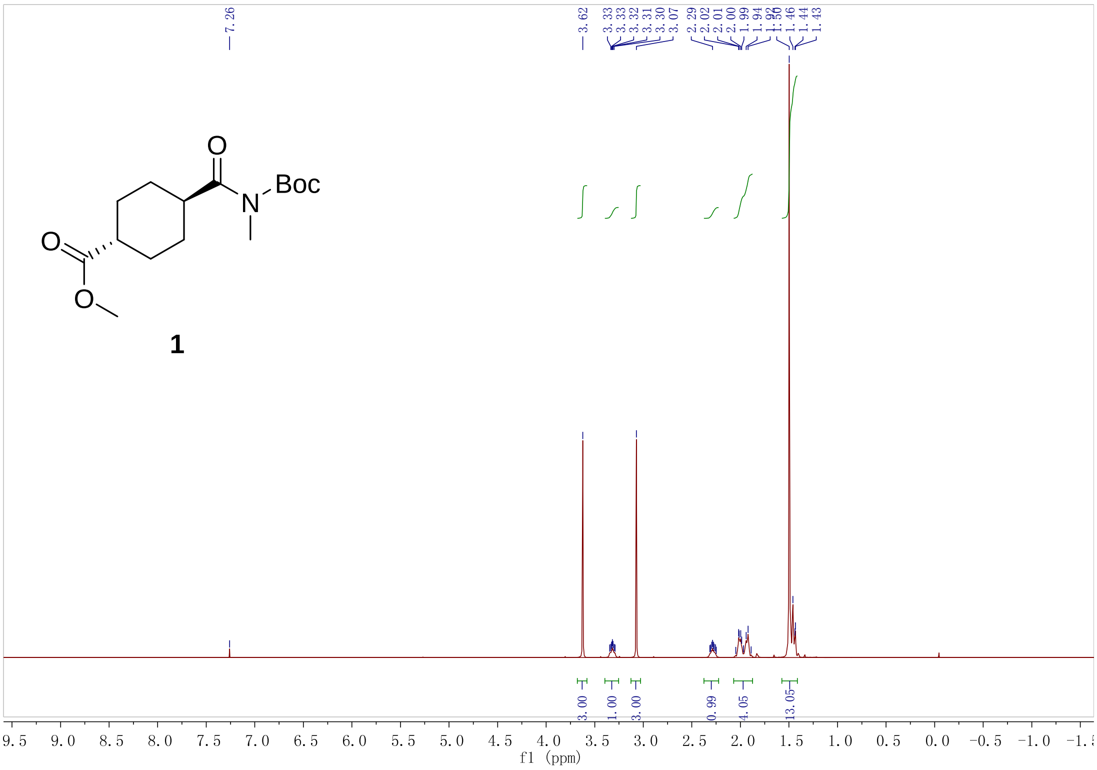

Supplementary Figure 1. ^{1}H NMR (400 MHz, $CDCl_3$) spectrum of compound **1**

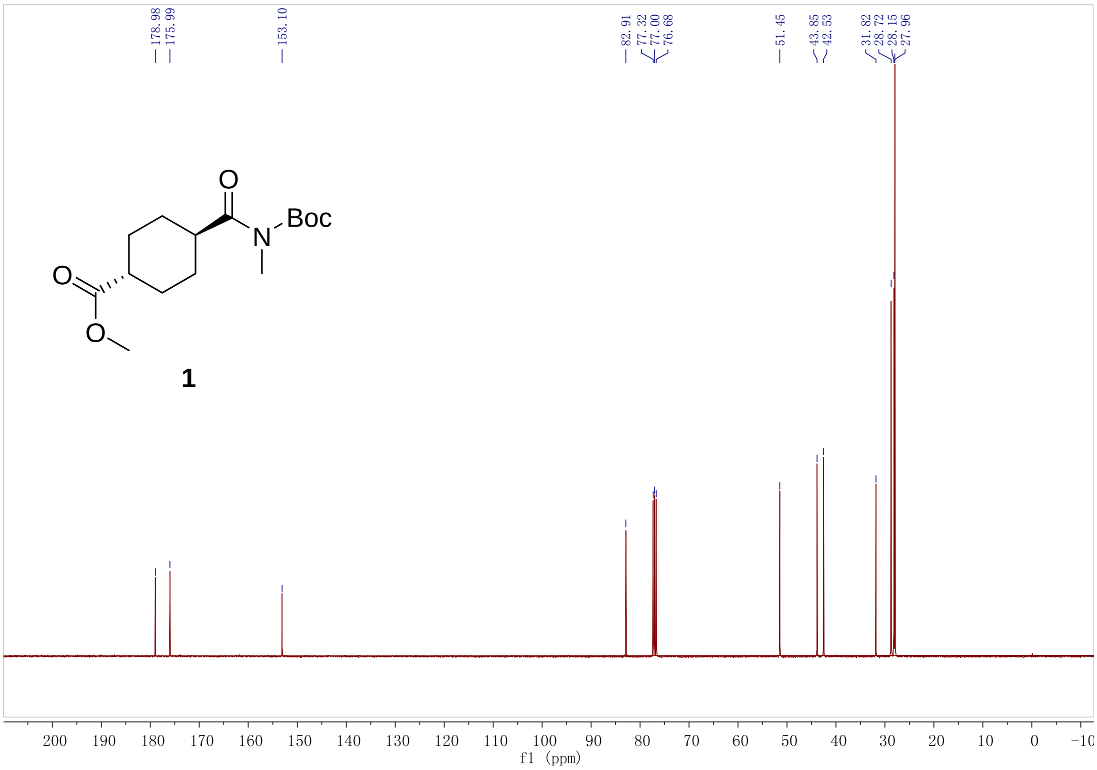

Supplementary Figure 2. ^{13}C NMR (400 MHz, $CDCl_3$) spectrum of compound **1**

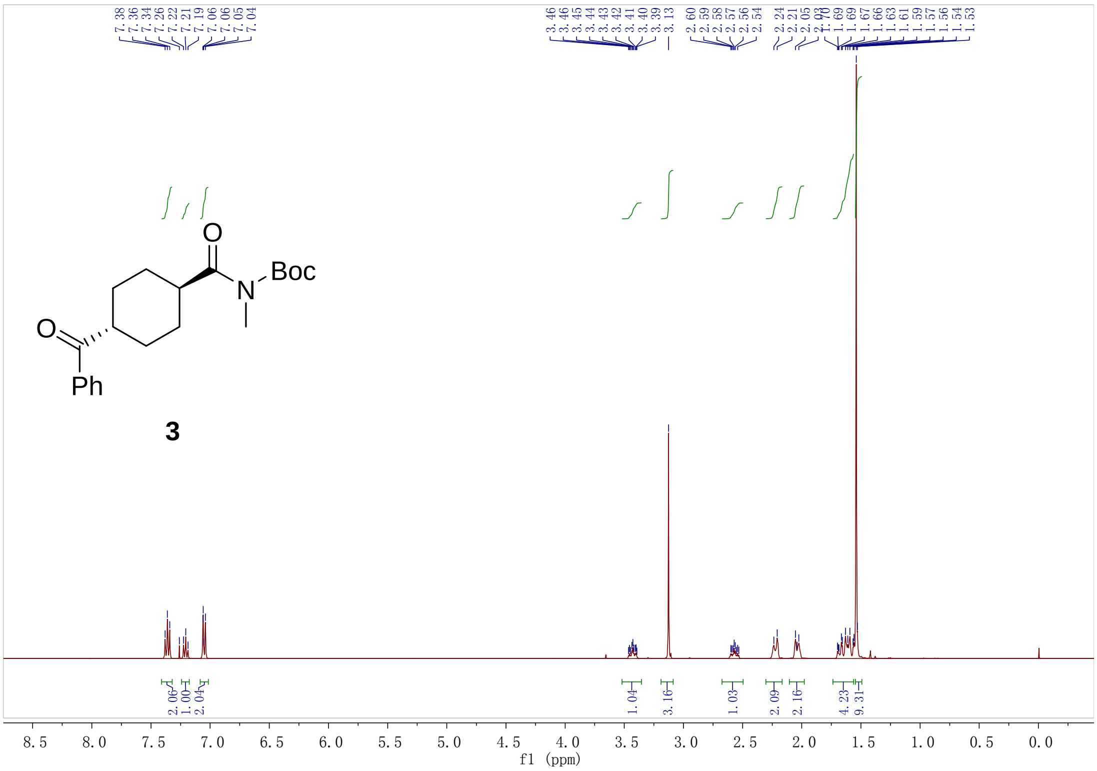

Supplementary Figure 3. ^{1}H NMR (400 MHz, $CDCl_3$) spectrum of compound **3**

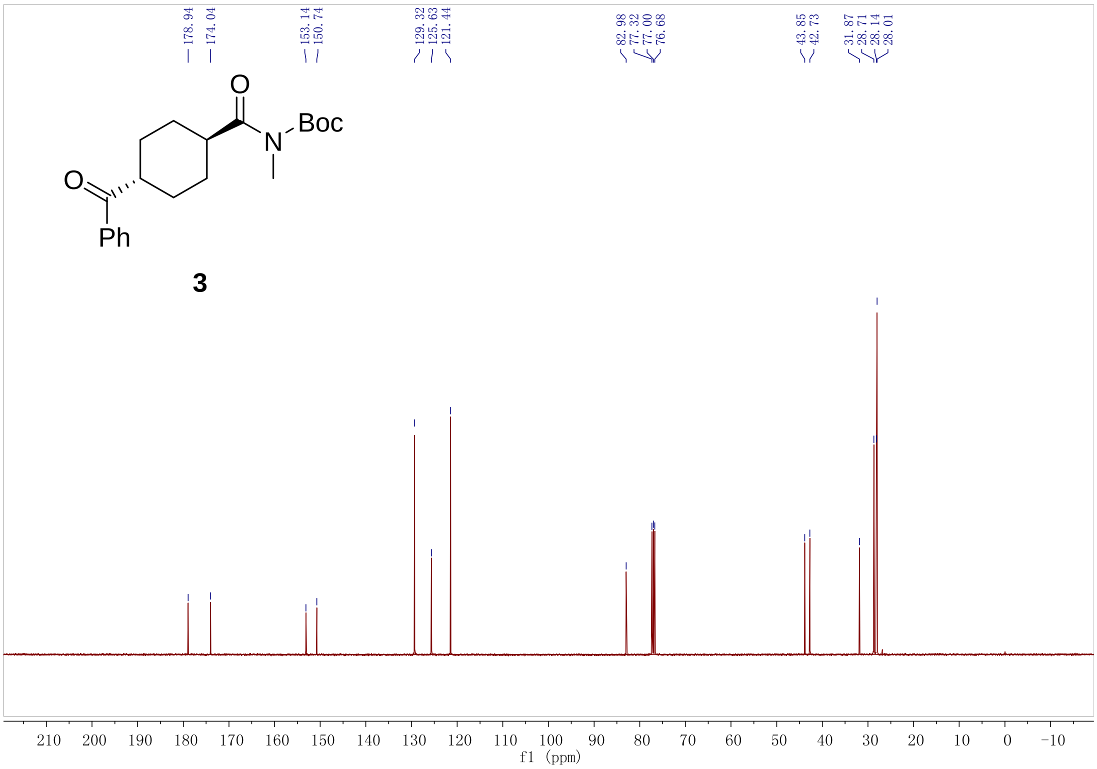

Supplementary Figure 4. ^{13}C NMR (400 MHz, $CDCl_3$) spectrum of compound **3**

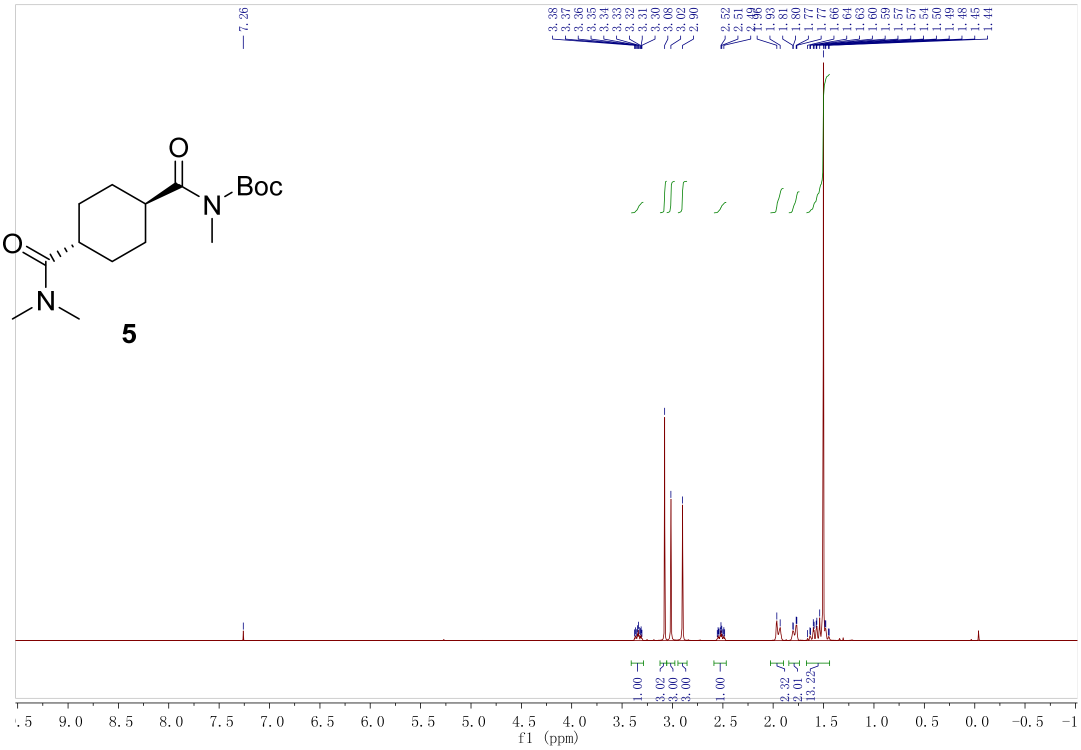

Supplementary Figure 5. ^{1}H NMR (400 MHz, $CDCl_3$) spectrum of compound **5**

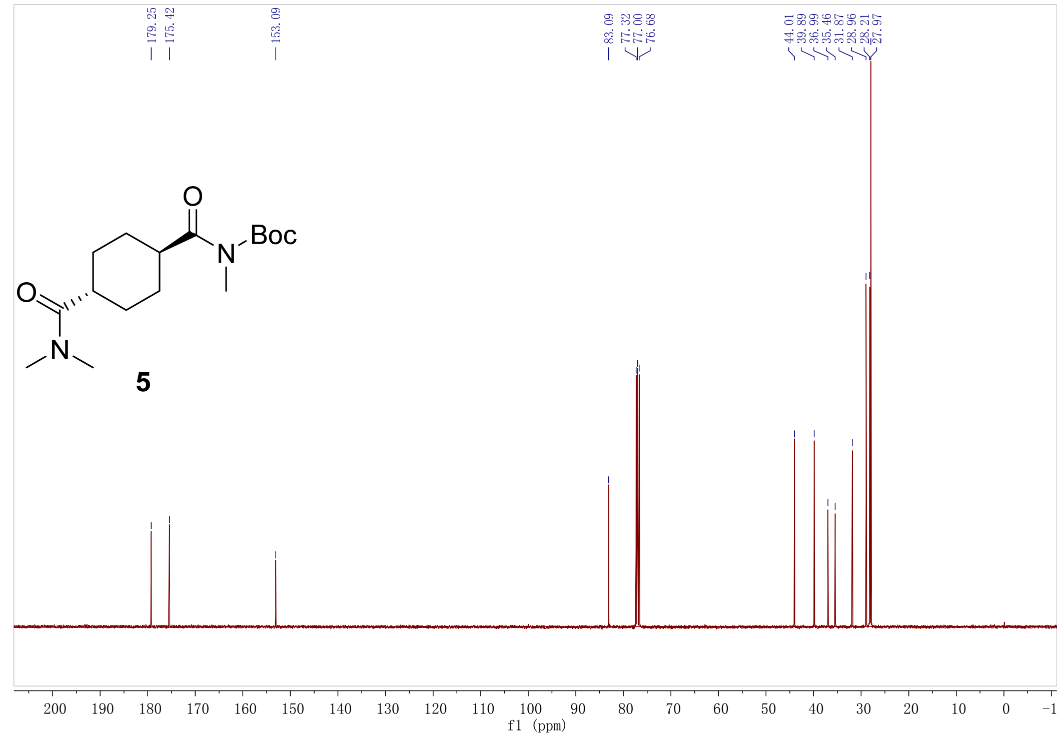

Supplementary Figure 6. ^{13}C NMR (400 MHz, $CDCl_3$) spectrum of compound **5**

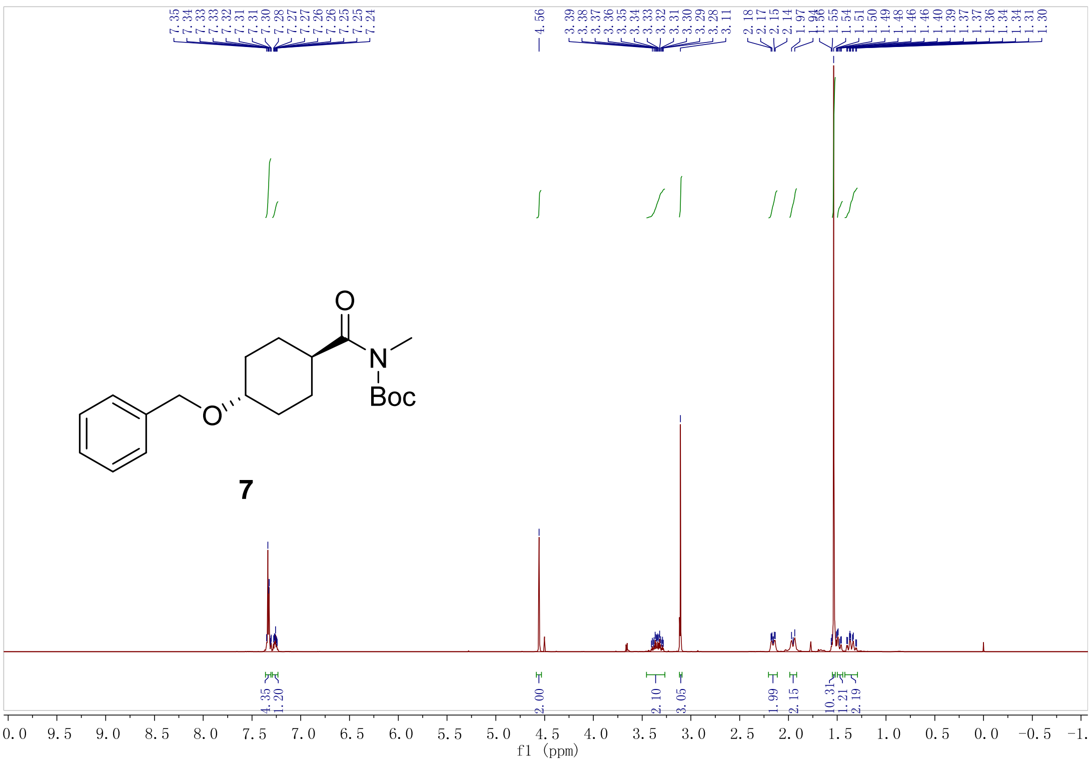

Supplementary Figure 7. ^{1}H NMR (400 MHz, $CDCl_3$) spectrum of compound **7**

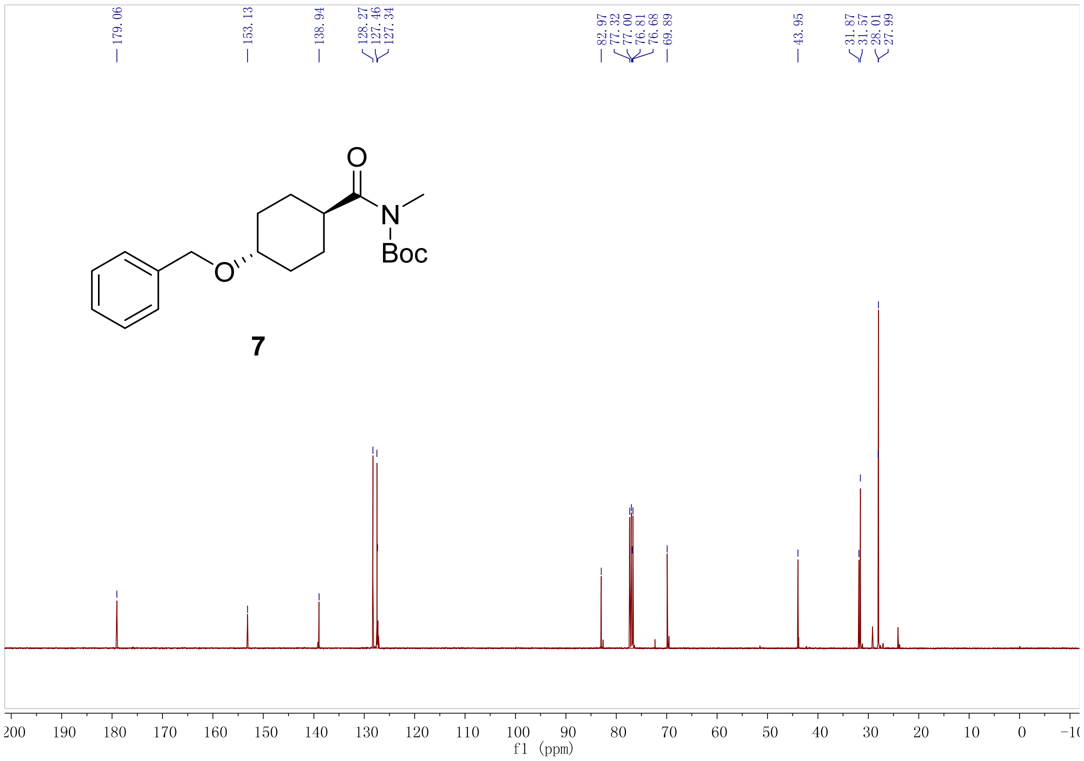

Supplementary Figure 8. ^{13}C NMR (400 MHz, $CDCl_3$) spectrum of compound **7**

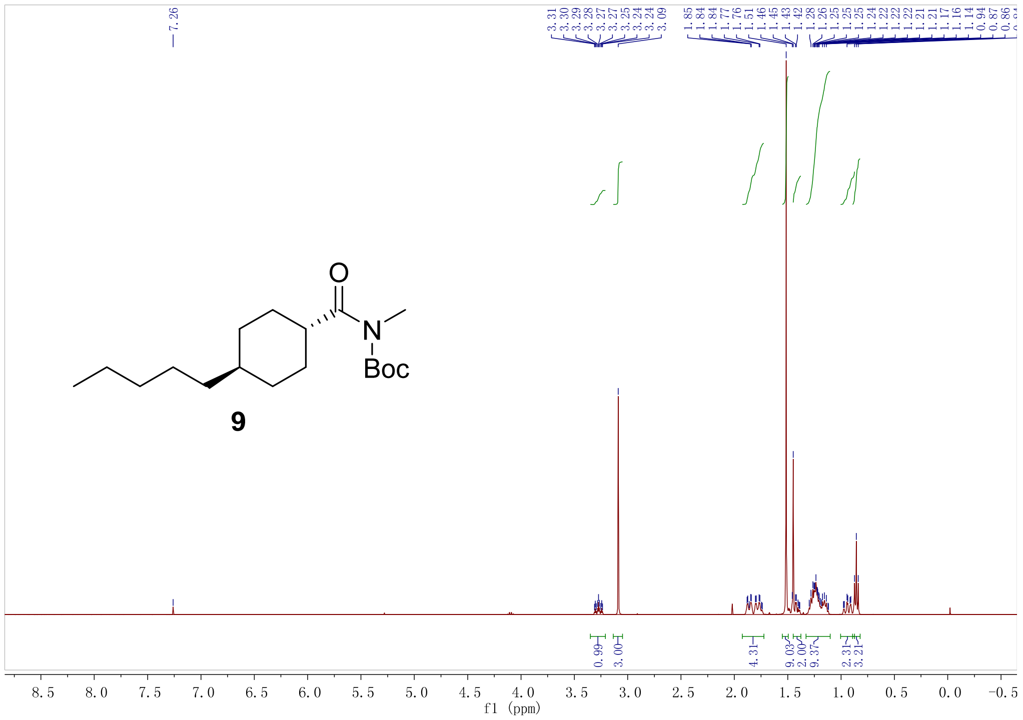

Supplementary Figure 9. ^{1}H NMR (400 MHz, $CDCl_3$) spectrum of compound **9**

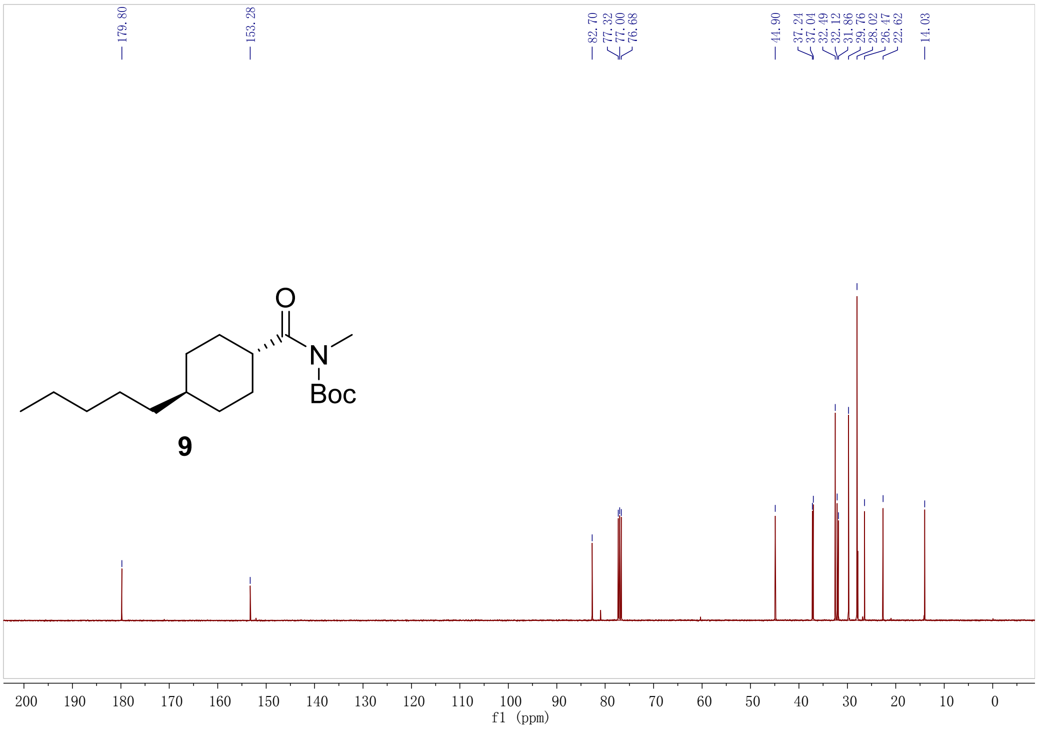

Supplementary Figure 10. ^{13}C NMR (400 MHz, $CDCl_3$) spectrum of compound **9**

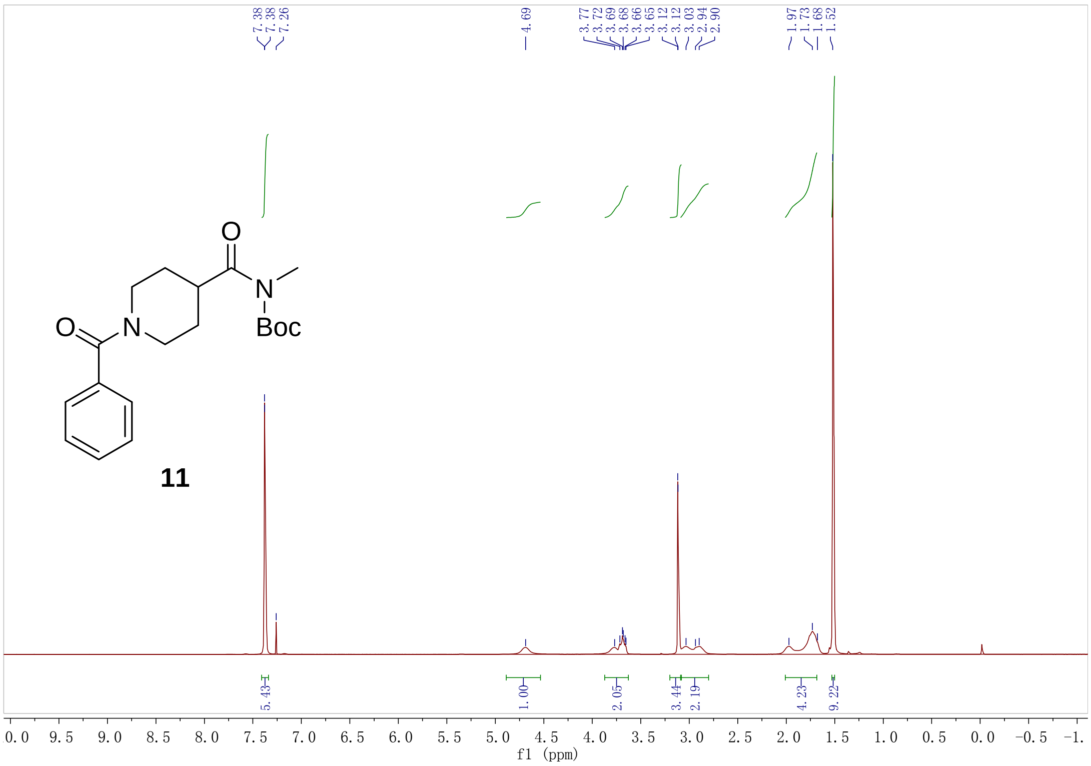

Supplementary Figure 11. ^{1}H NMR (400 MHz, $CDCl_3$) spectrum of compound **11**

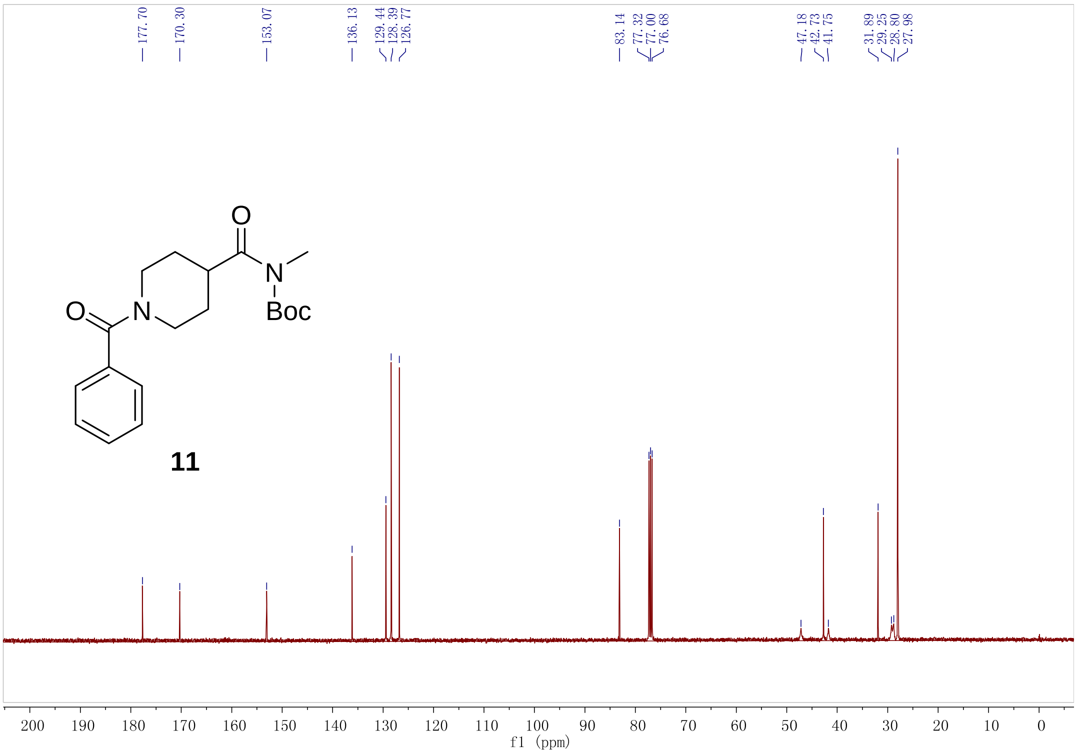

Supplementary Figure 12. ^{13}C NMR (400 MHz, $CDCl_3$) spectrum of compound **11**

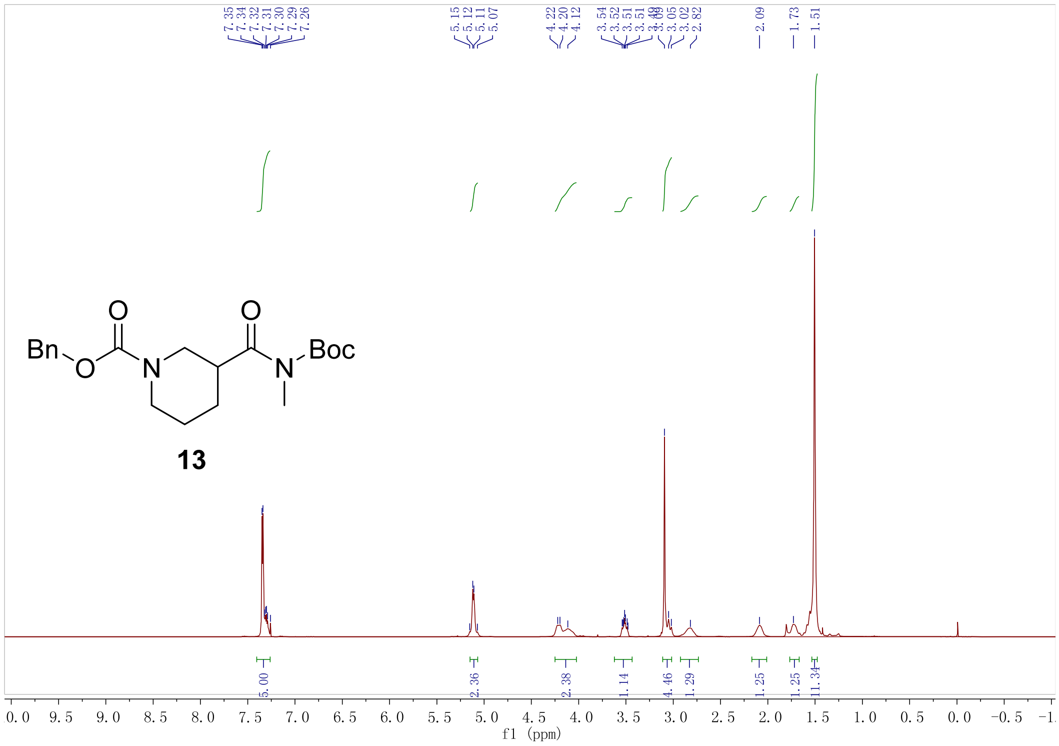

Supplementary Figure 13. ^{1}H NMR (400 MHz, $CDCl_3$) spectrum of compound **13**

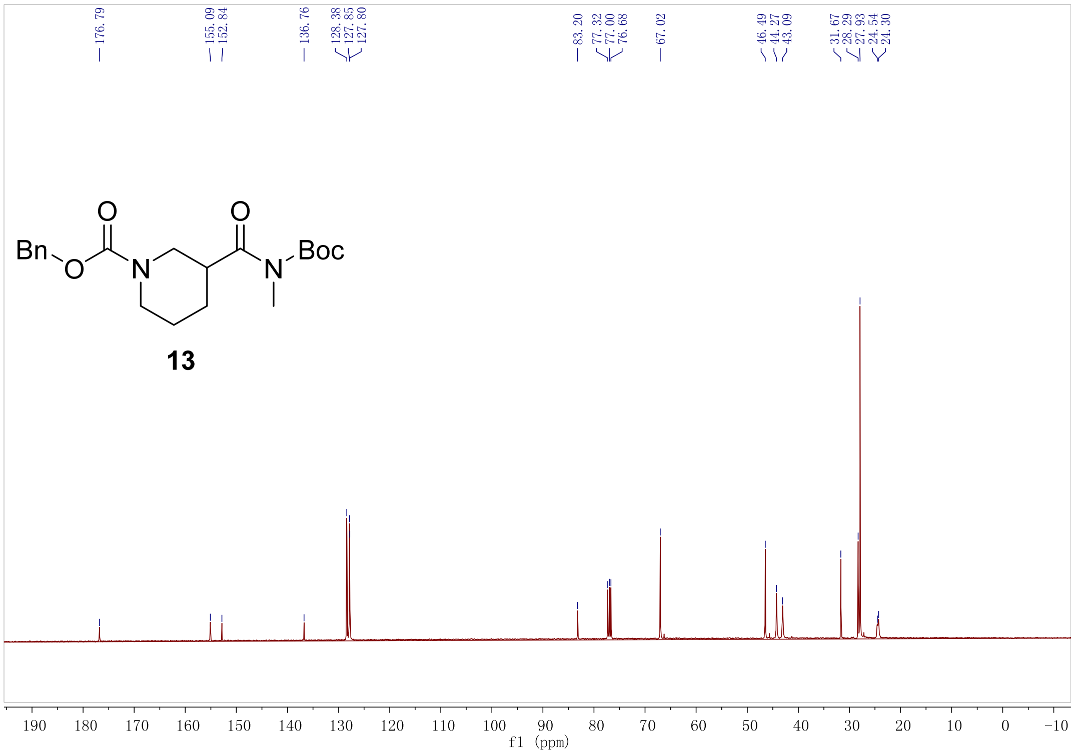

Supplementary Figure 14. ^{13}C NMR (400 MHz, $CDCl_3$) spectrum of compound **13**

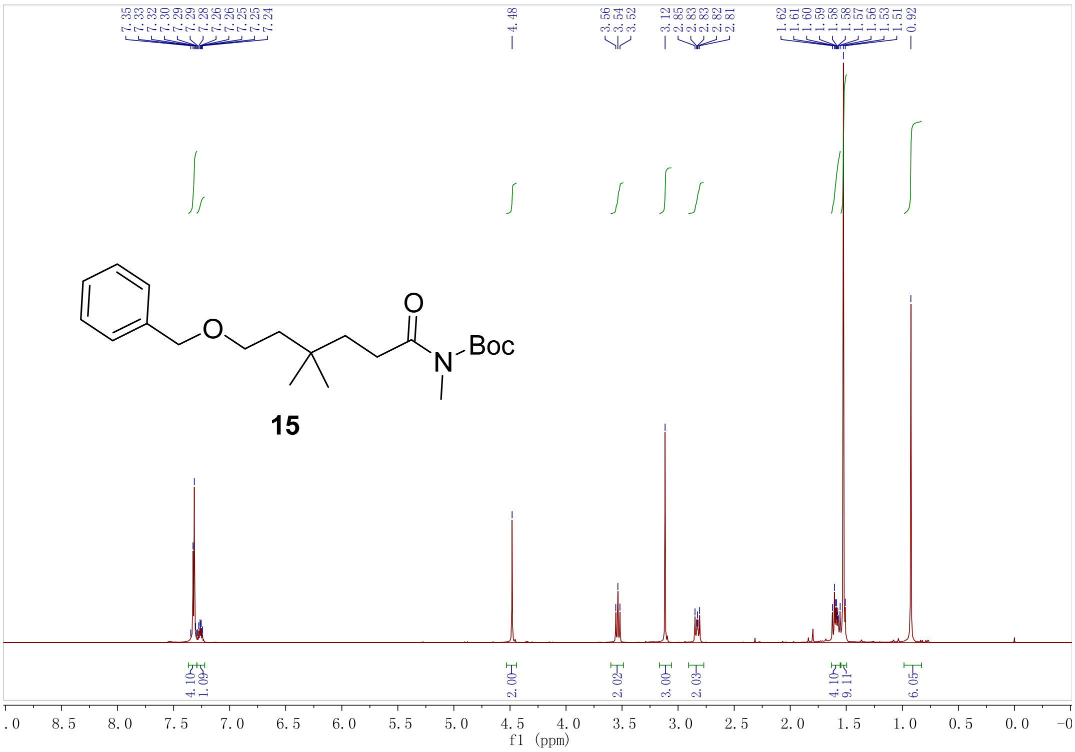

Supplementary Figure 15. ^{1}H NMR (400 MHz, $CDCl_3$) spectrum of compound **15**

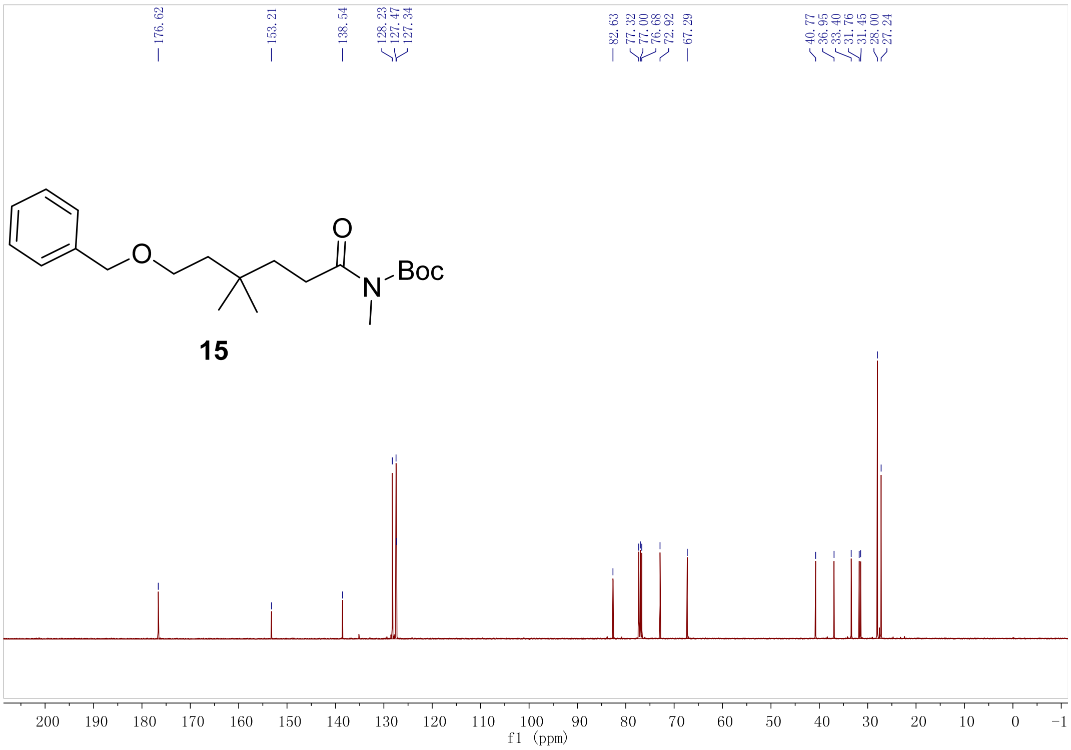

Supplementary Figure 16. ^{13}C NMR (400 MHz, $CDCl_3$) spectrum of compound **15**

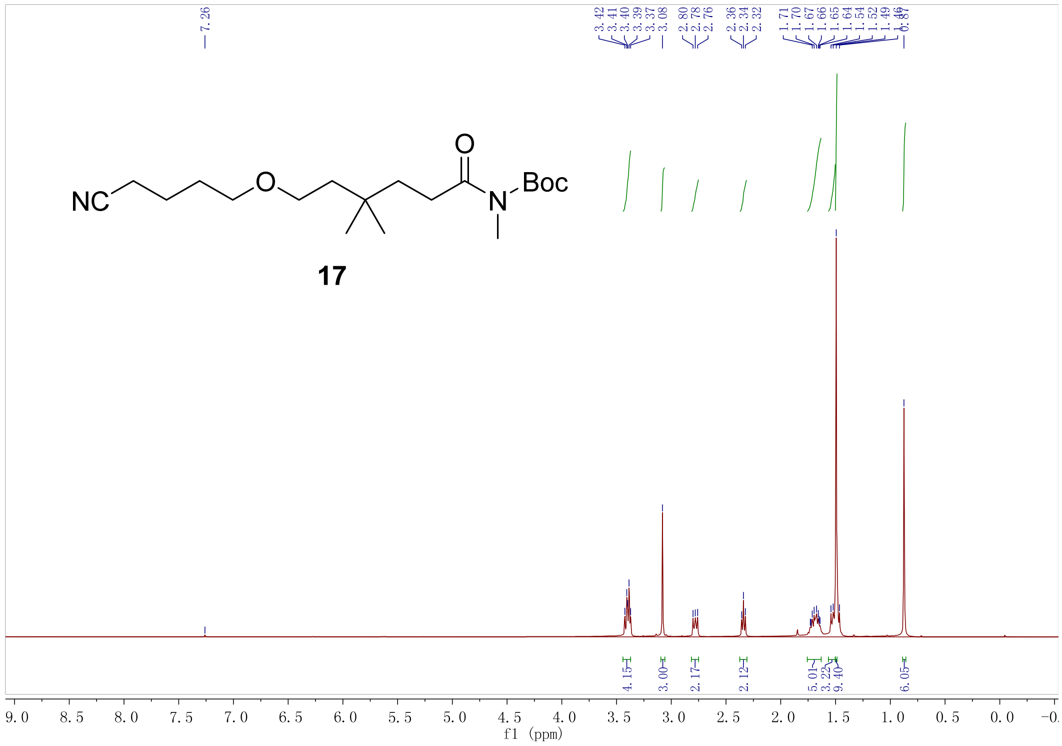

Supplementary Figure 17. ^{1}H NMR (400 MHz, $CDCl_3$) spectrum of compound **17**

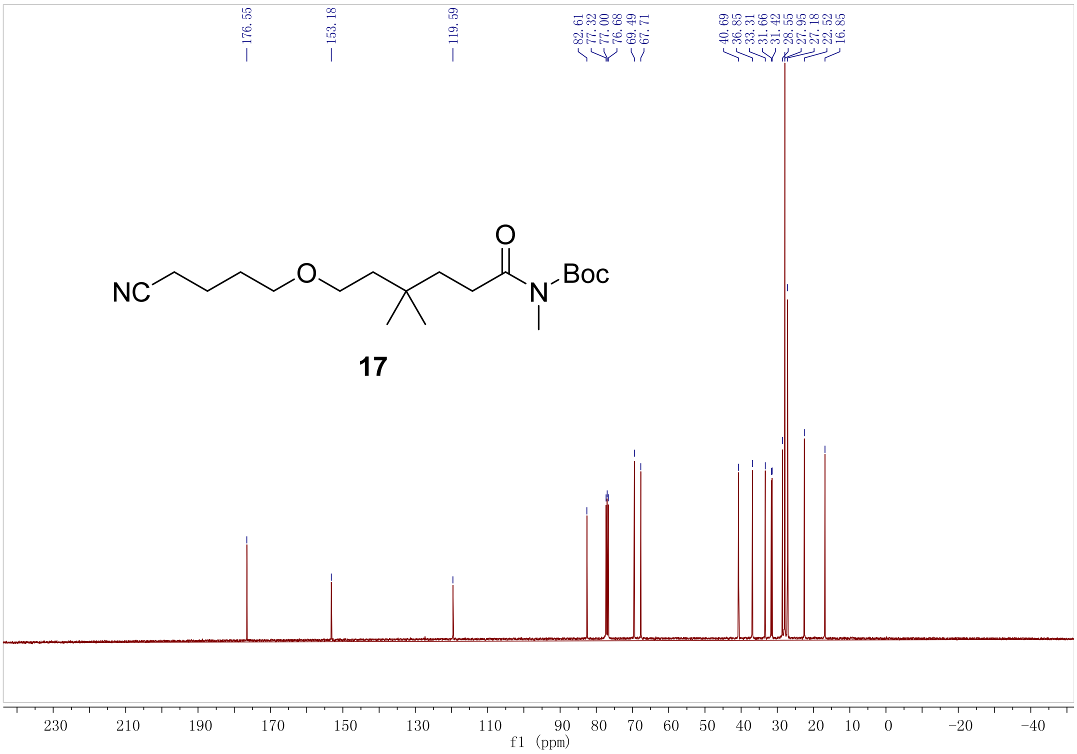

Supplementary Figure 18. ^{13}C NMR (400 MHz, $CDCl_3$) spectrum of compound **17**

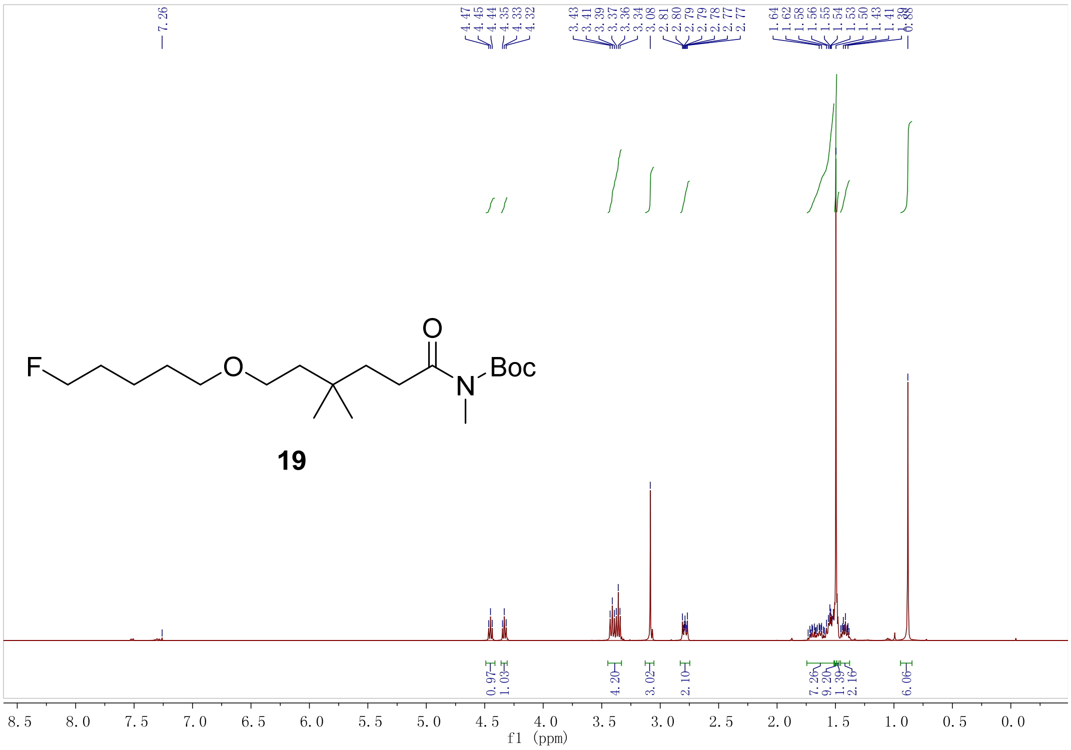

Supplementary Figure 19. ^{1}H NMR (400 MHz, $CDCl_3$) spectrum of compound **19**

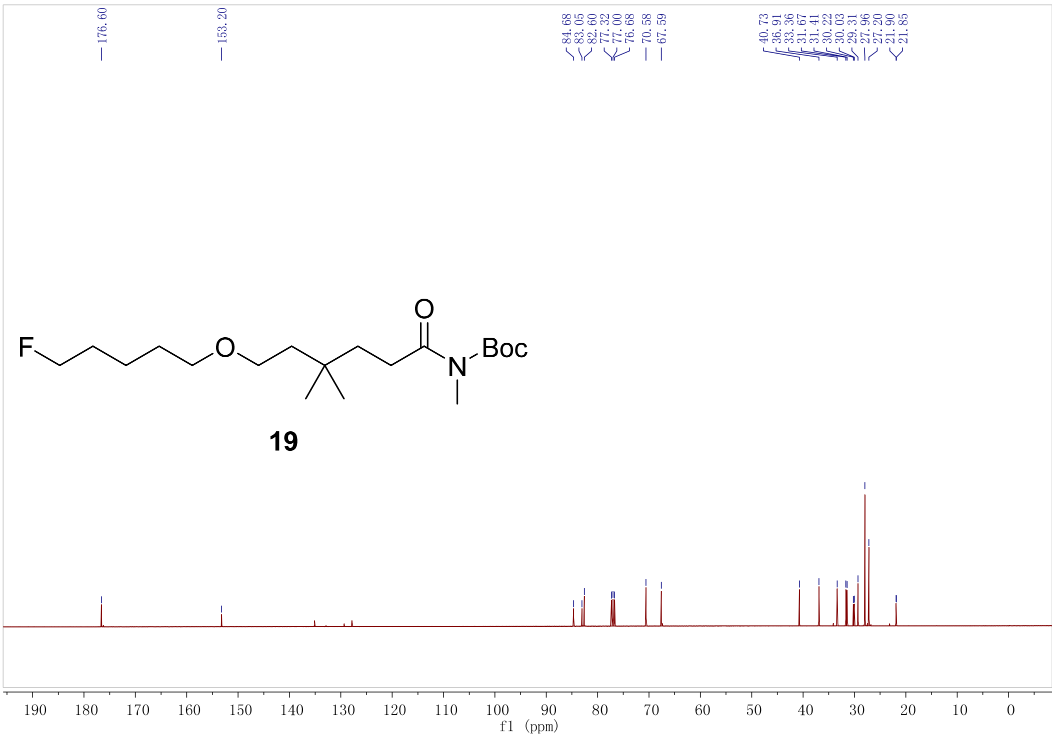

Supplementary Figure 20. ^{13}C NMR (400 MHz, $CDCl_3$) spectrum of compound **19**

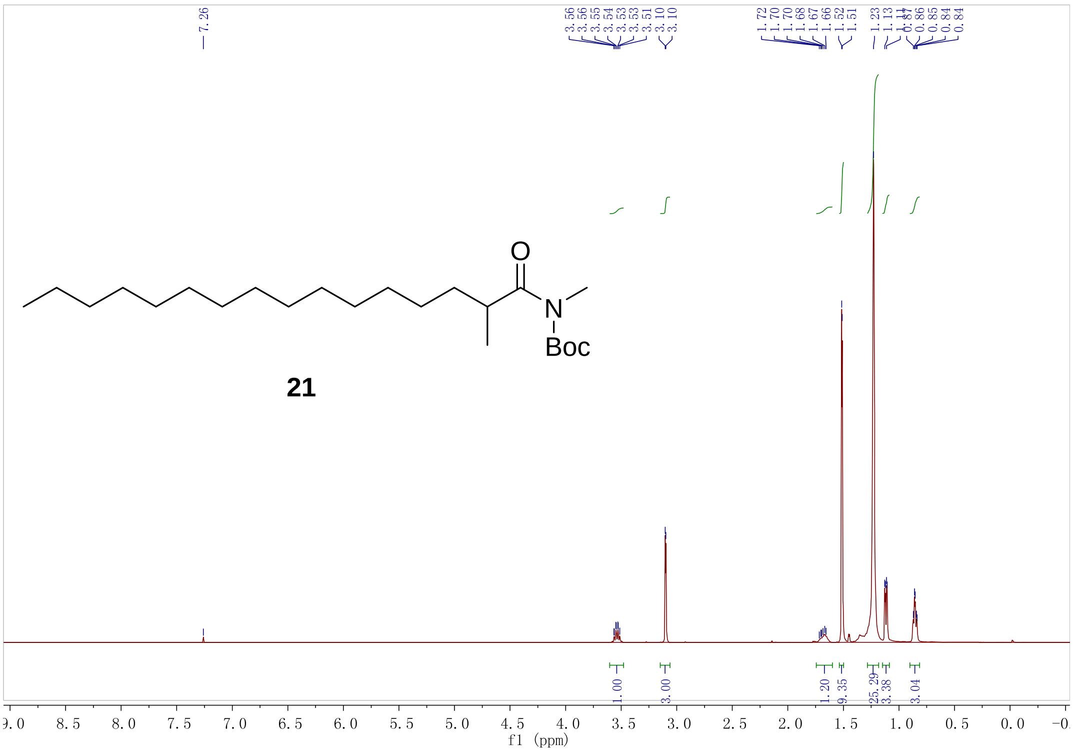

Supplementary Figure 21. ^{1}H NMR (400 MHz, $CDCl_3$) spectrum of compound **21**

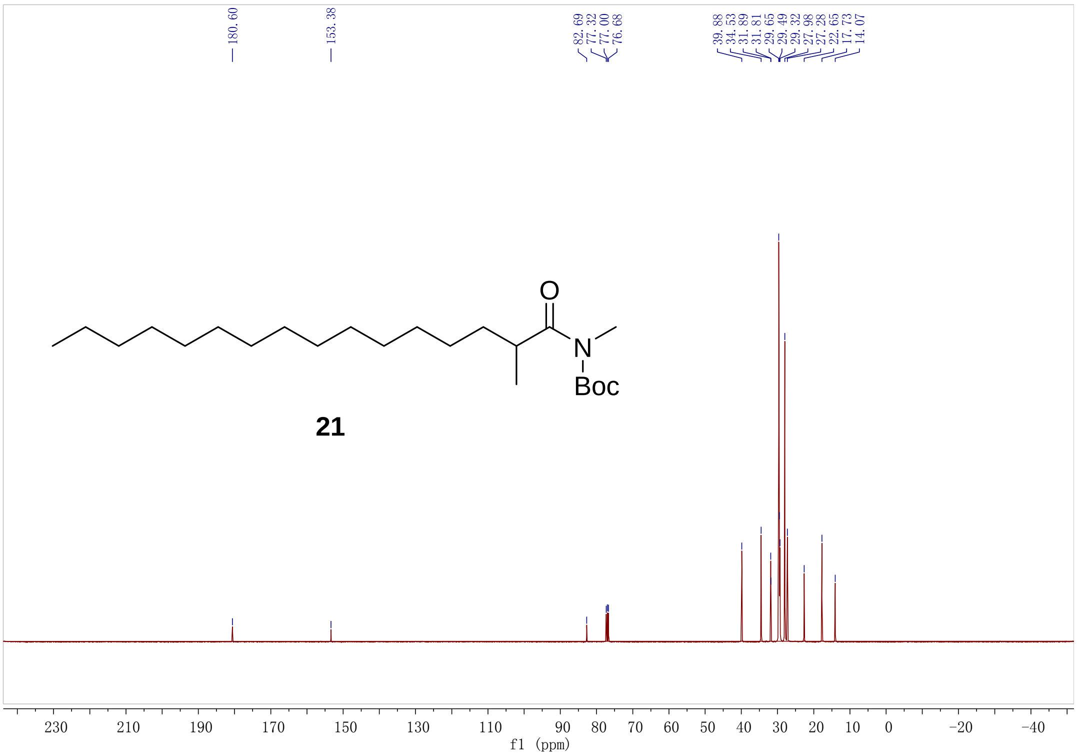

Supplementary Figure 22. ^{13}C NMR (400 MHz, $CDCl_3$) spectrum of compound **21**

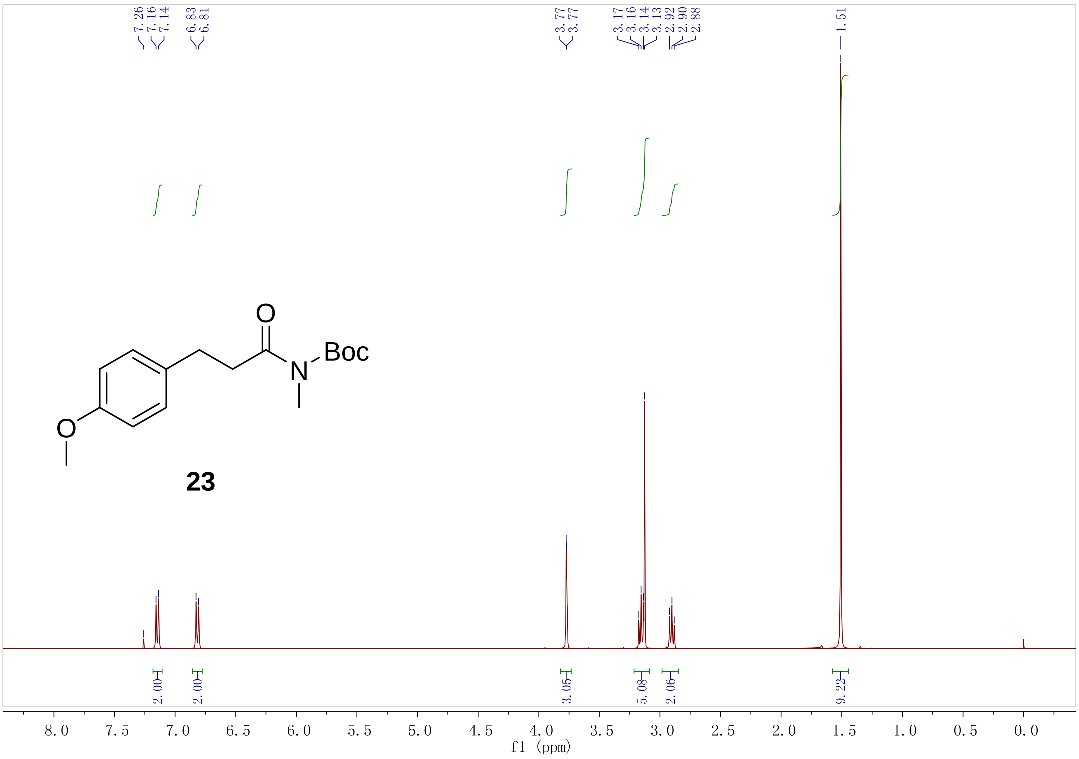

Supplementary Figure 23. ^{1}H NMR (400 MHz, $CDCl_3$) spectrum of compound **23**

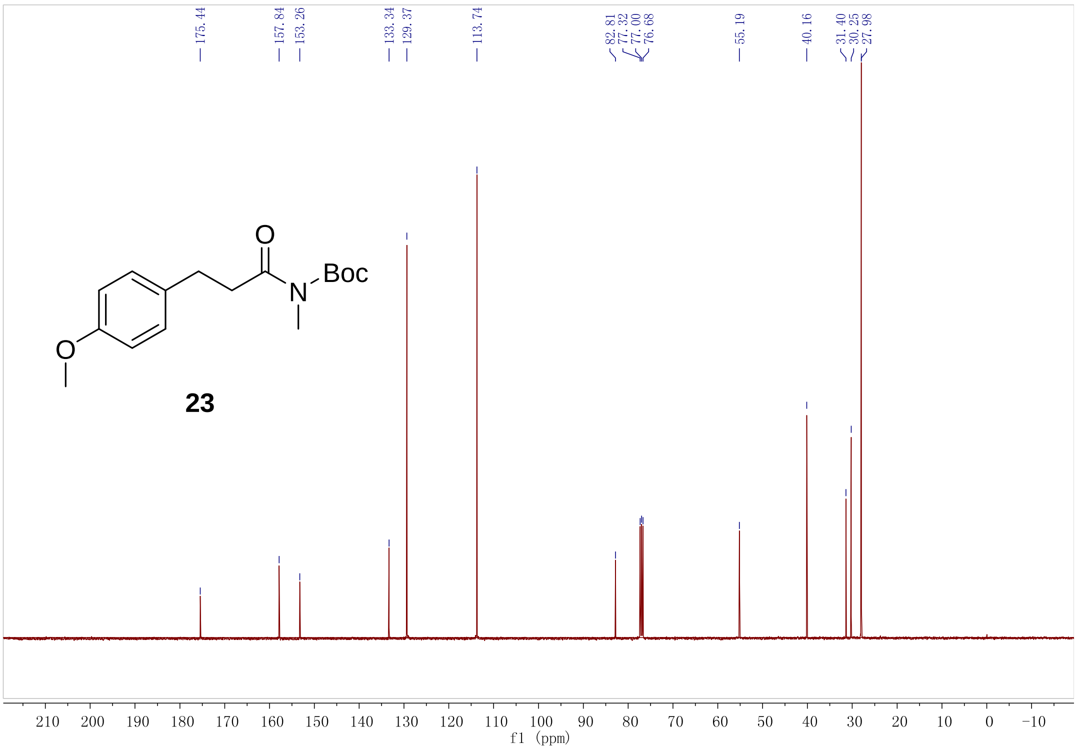

Supplementary Figure 24. ^{13}C NMR (400 MHz, $CDCl_3$) spectrum of compound **23**

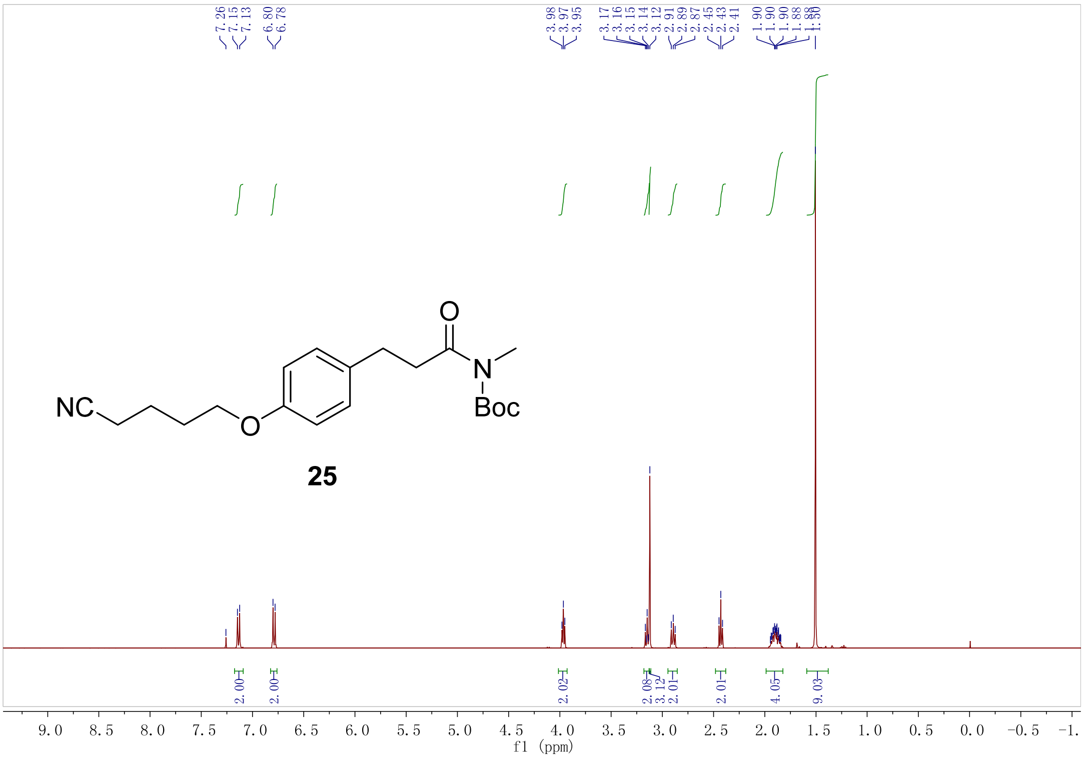

Supplementary Figure 25. ^{1}H NMR (400 MHz, $CDCl_3$) spectrum of compound **25**

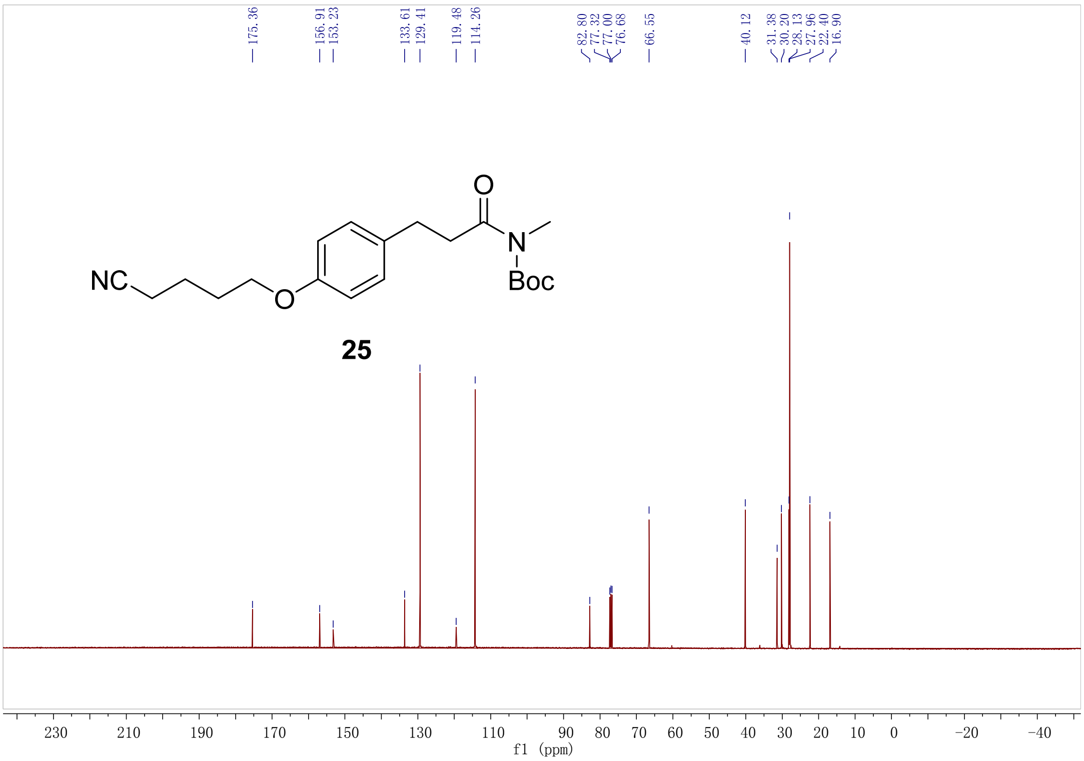

Supplementary Figure 26. ^{13}C NMR (400 MHz, $CDCl_3$) spectrum of compound **25**

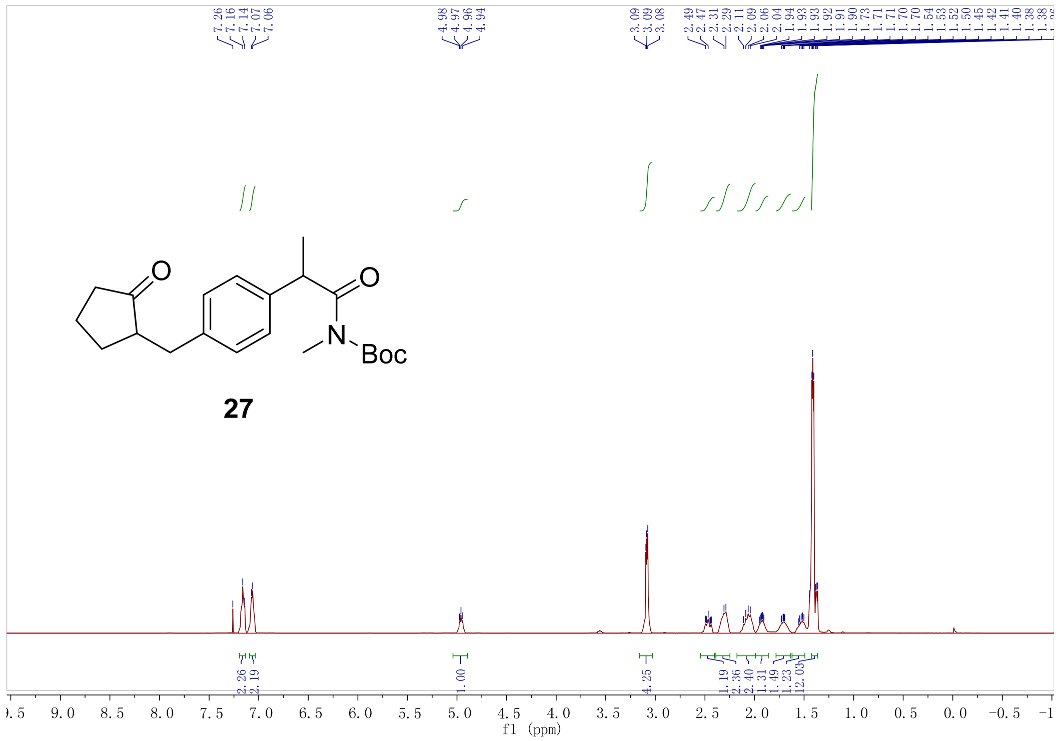

Supplementary Figure 27. ^{1}H NMR (400 MHz, $CDCl_3$) spectrum of compound **27**

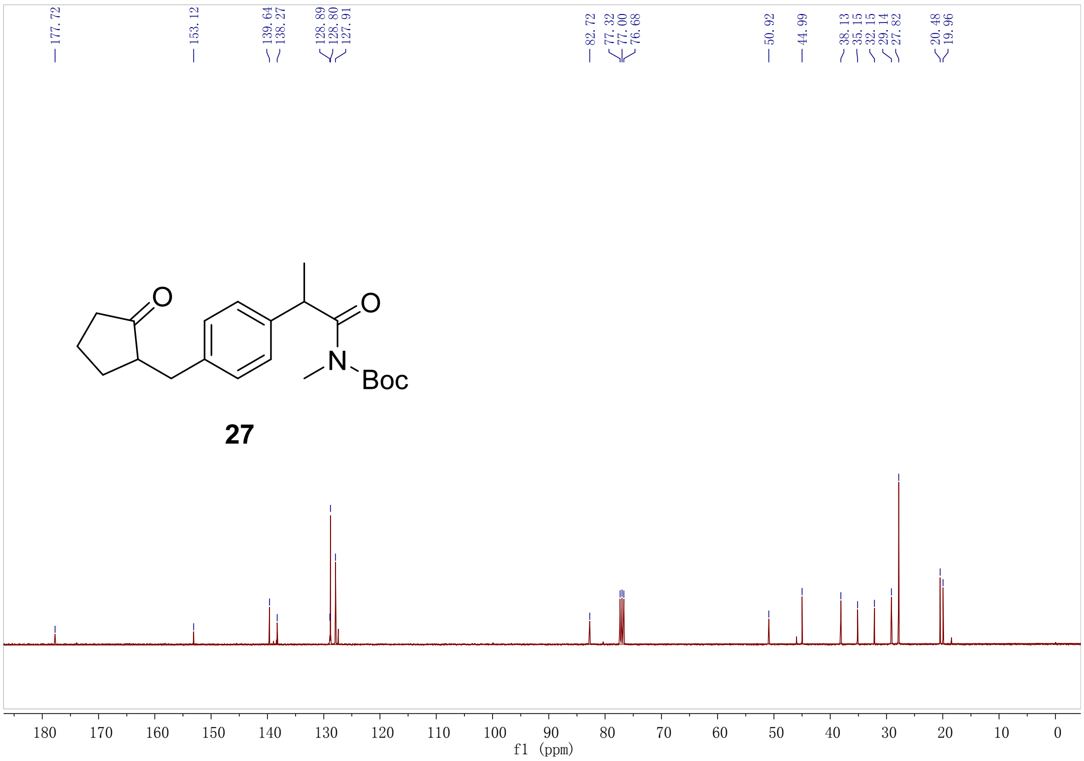

Supplementary Figure 28. ^{13}C NMR (400 MHz, $CDCl_3$) spectrum of compound **27**

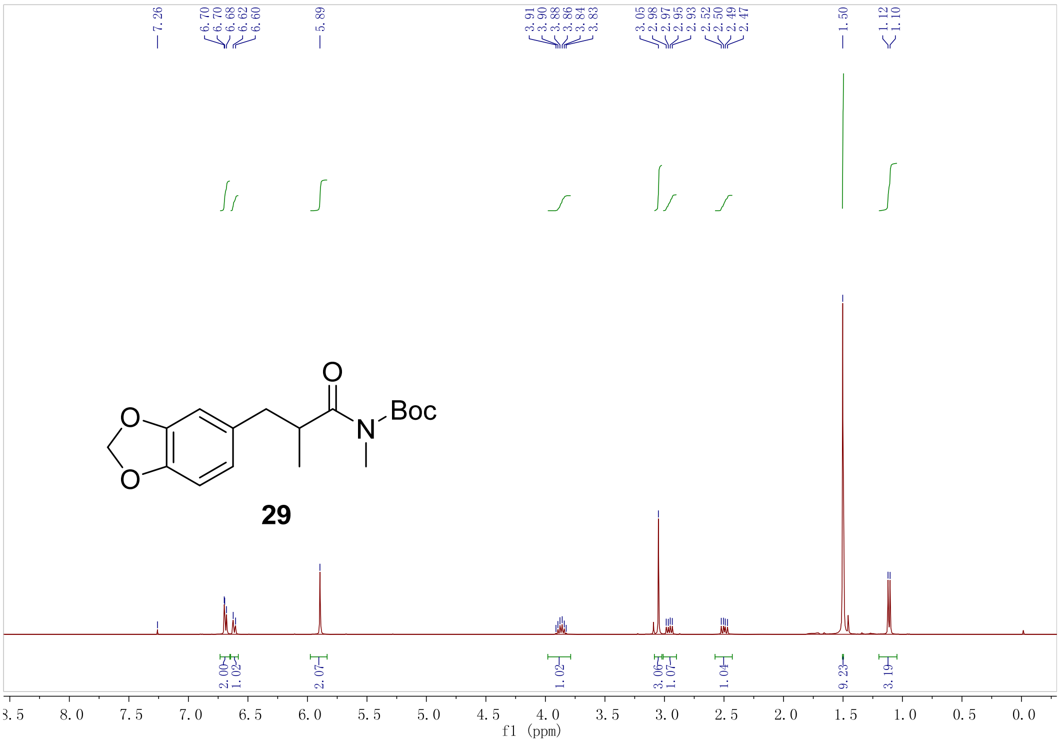

Supplementary Figure 29. ^{1}H NMR (400 MHz, $CDCl_3$) spectrum of compound **29**

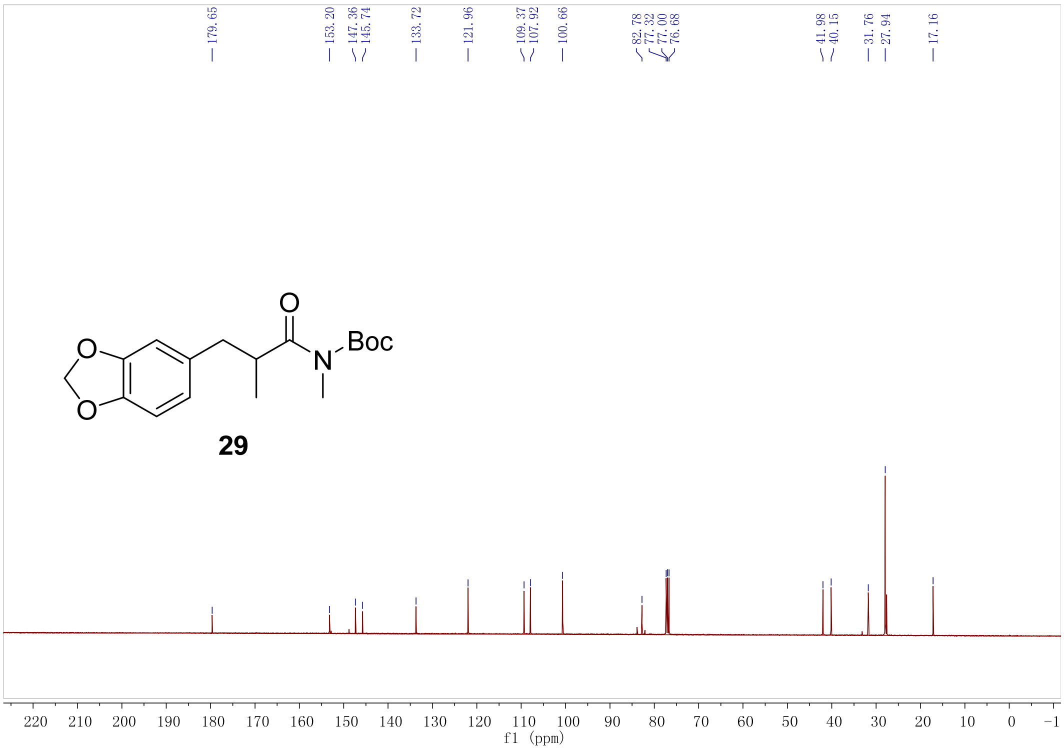

Supplementary Figure 30. ^{13}C NMR (400 MHz, $CDCl_3$) spectrum of compound **29**

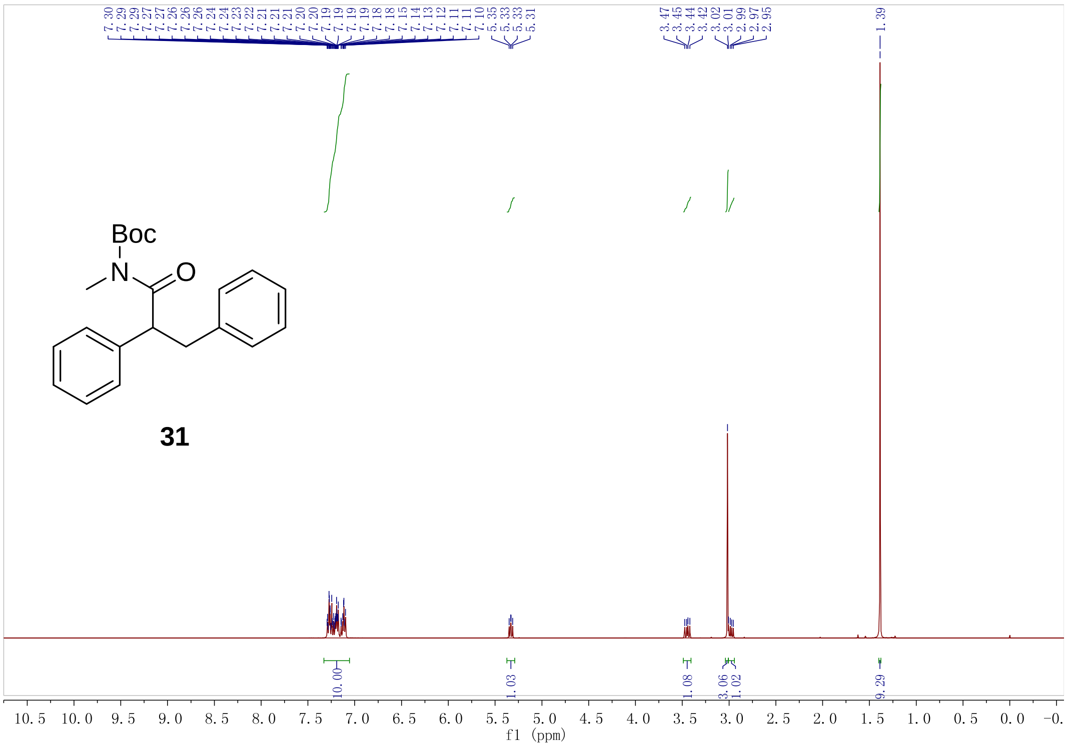

Supplementary Figure 31. ^{1}H NMR (400 MHz, $CDCl_3$) spectrum of compound **31**

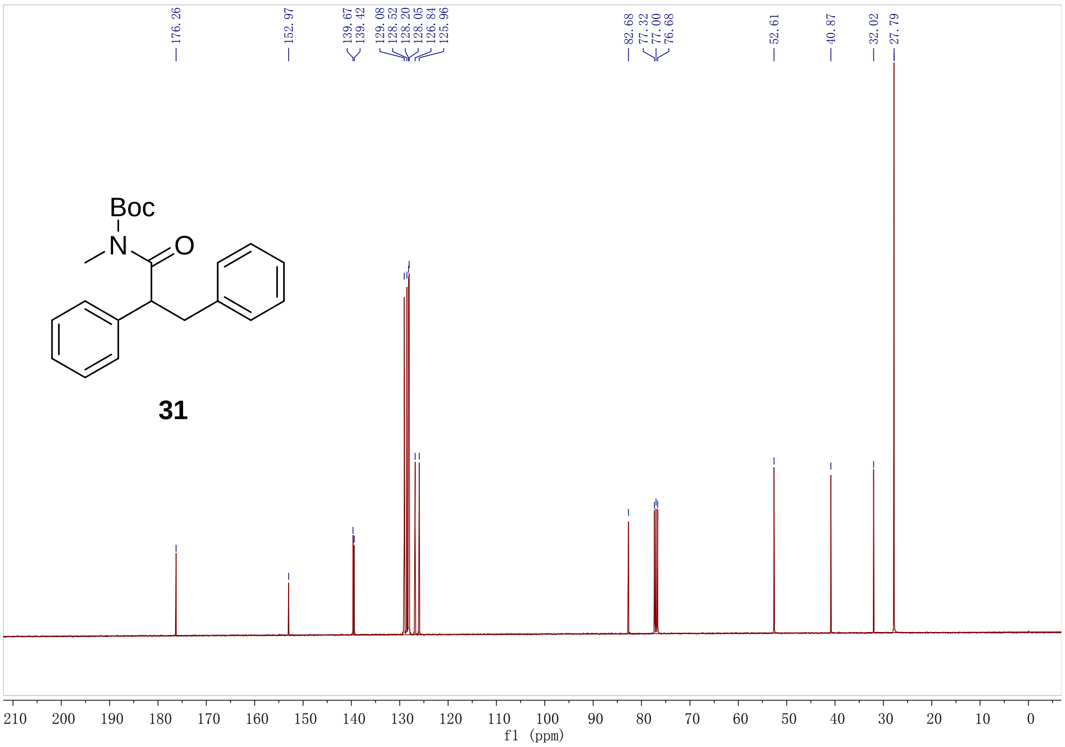

Supplementary Figure 32. ^{13}C NMR (400 MHz, $CDCl_3$) spectrum of compound **32**

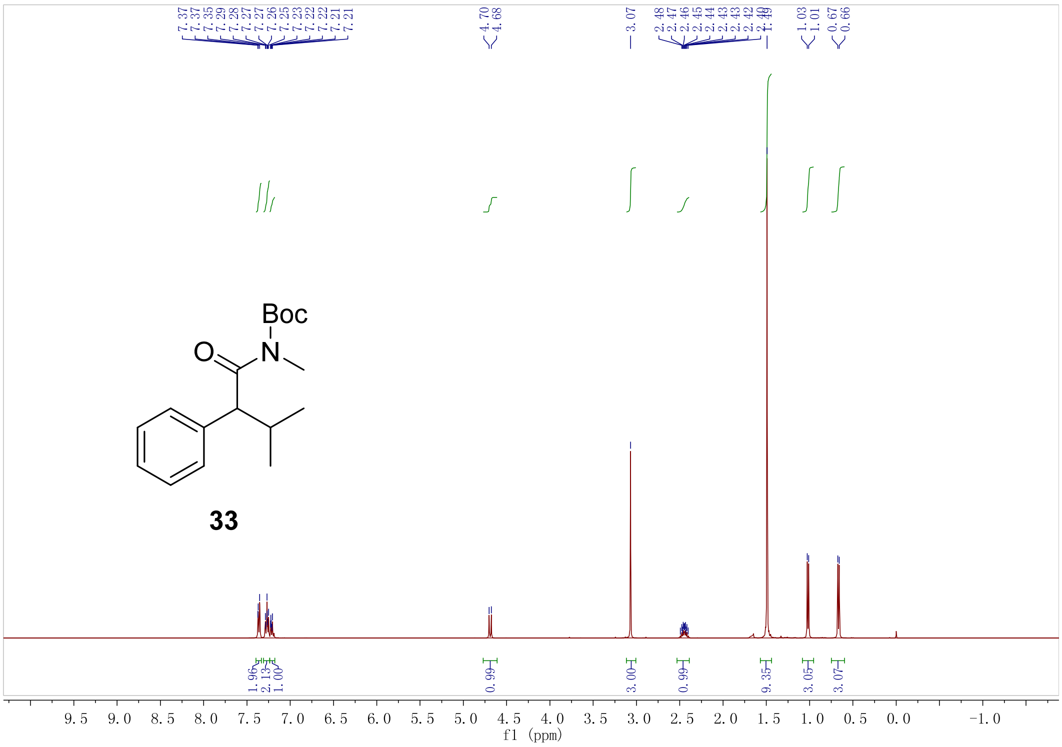

Supplementary Figure 33. ^{1}H NMR (400 MHz, $CDCl_3$) spectrum of compound **33**

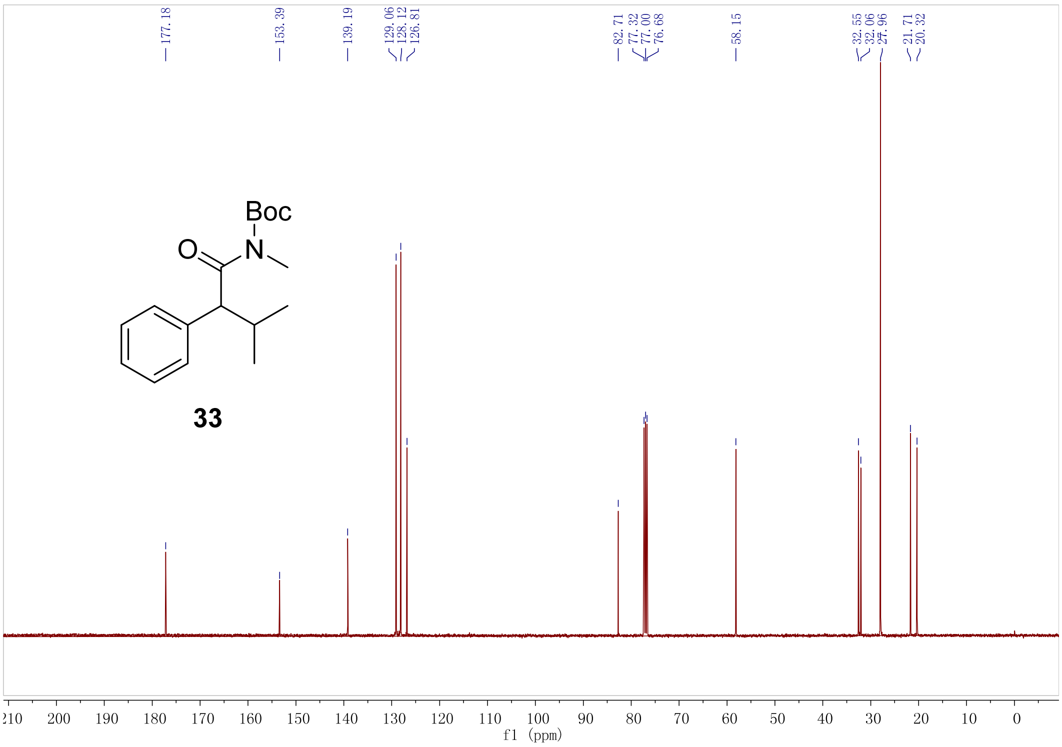

Supplementary Figure 34. ^{13}C NMR (400 MHz, $CDCl_3$) spectrum of compound **33**

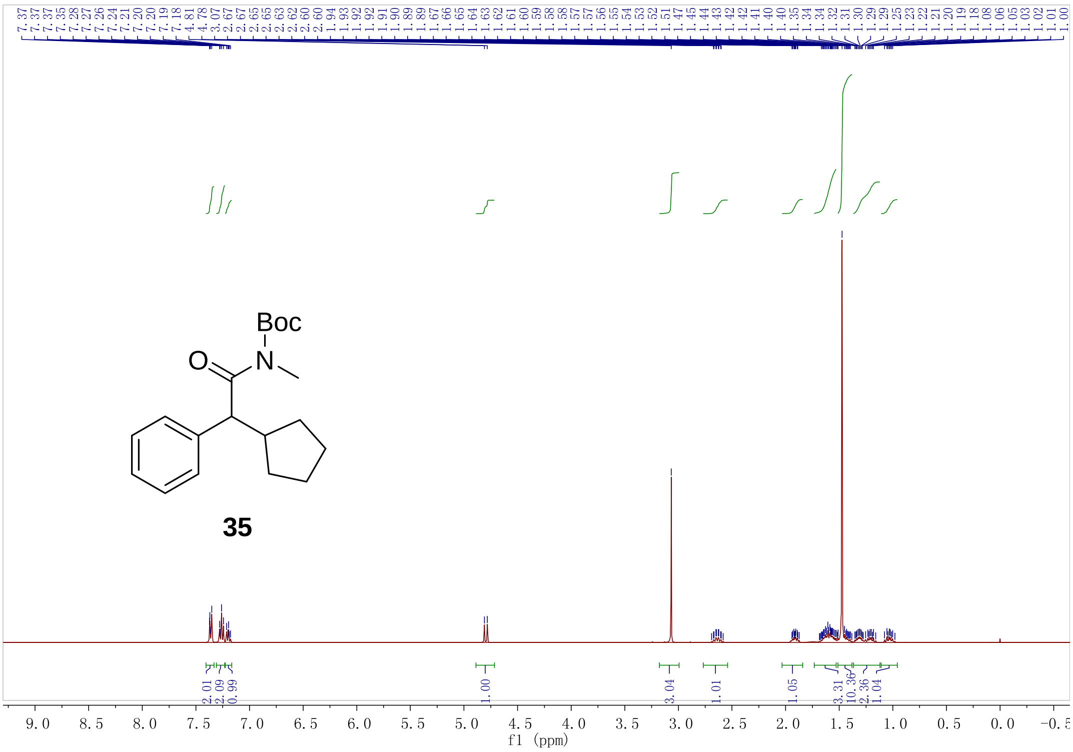

Supplementary Figure 35. ^{1}H NMR (400 MHz, $CDCl_3$) spectrum of compound **35**

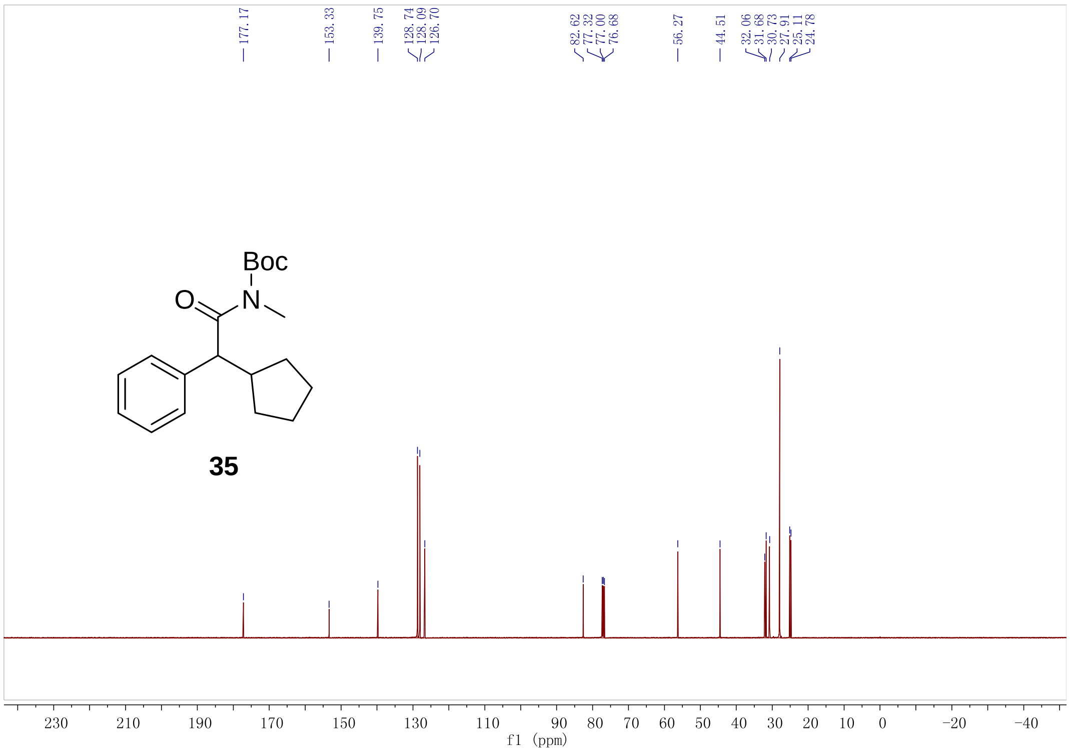

Supplementary Figure 36. ^{13}C NMR (400 MHz, $CDCl_3$) spectrum of compound **35**

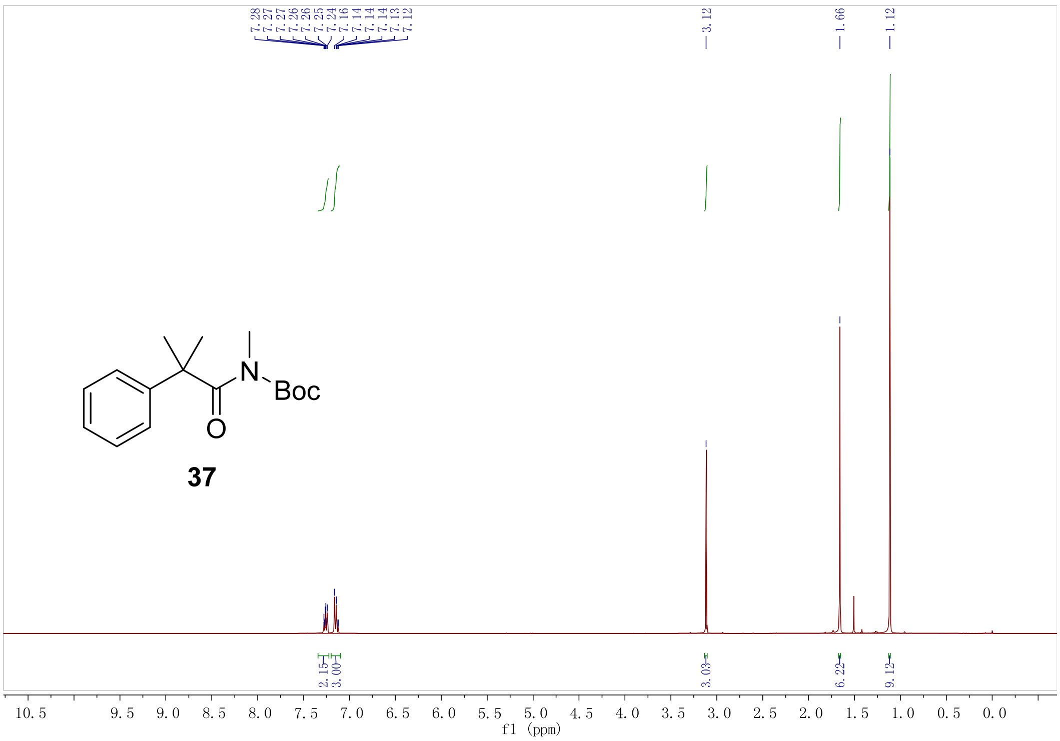

Supplementary Figure 37. ^{1}H NMR (400 MHz, $CDCl_3$) spectrum of compound **37**

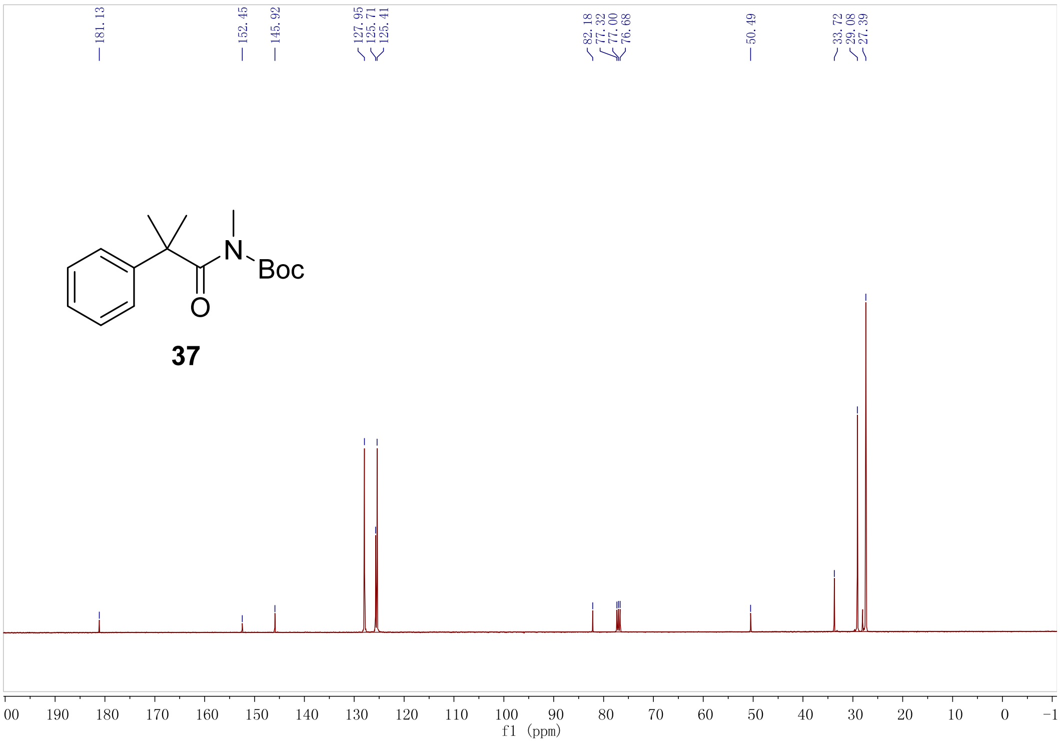

Supplementary Figure 38. ^{13}C NMR (400 MHz, $CDCl_3$) spectrum of compound **37**

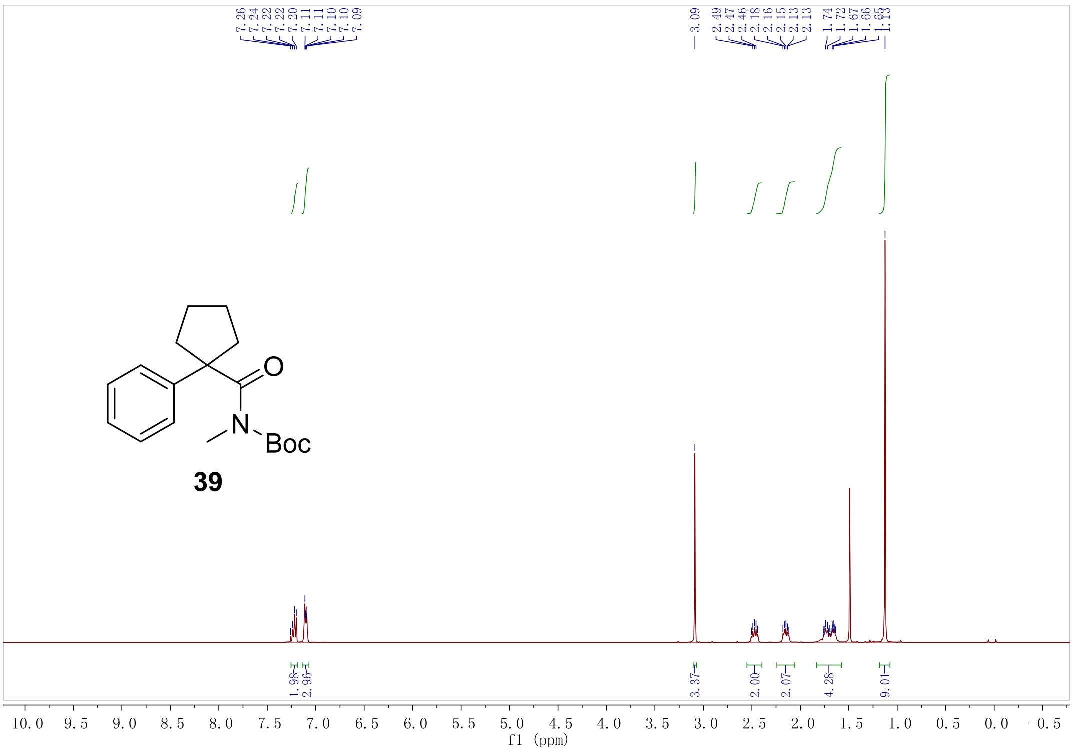

Supplementary Figure 39. ^{1}H NMR (400 MHz, $CDCl_3$) spectrum of compound **39**

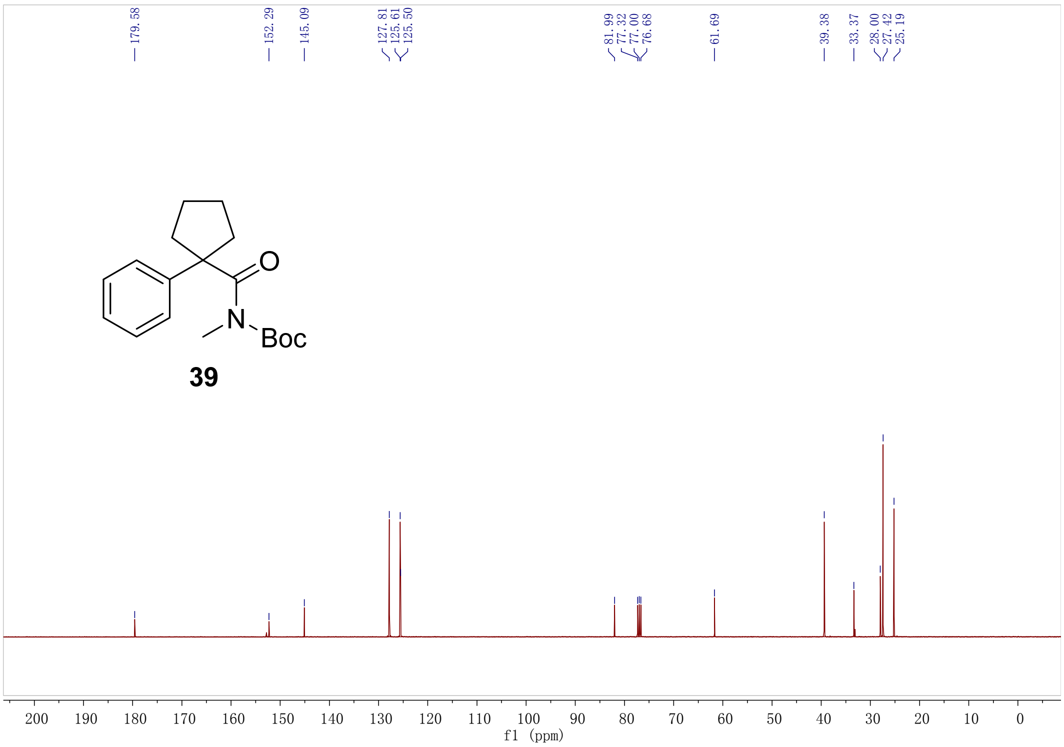

Supplementary Figure 40. ^{13}C NMR (400 MHz, $CDCl_3$) spectrum of compound **39**

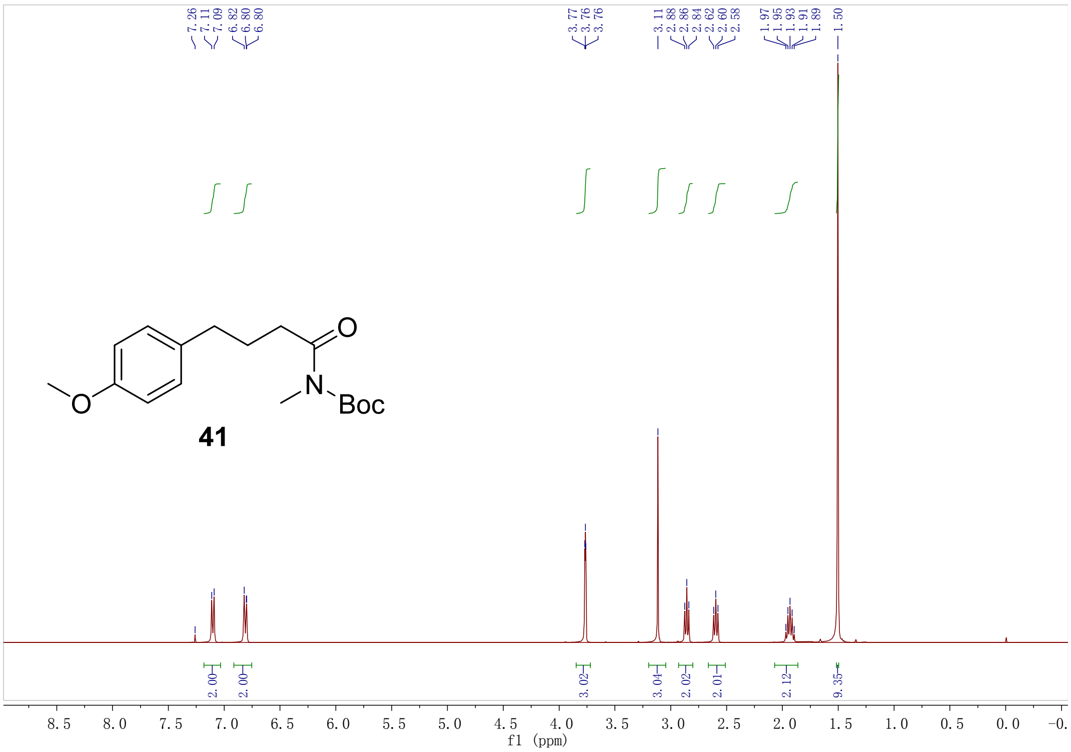

Supplementary Figure 41. ^{1}H NMR (400 MHz, $CDCl_3$) spectrum of compound **41**

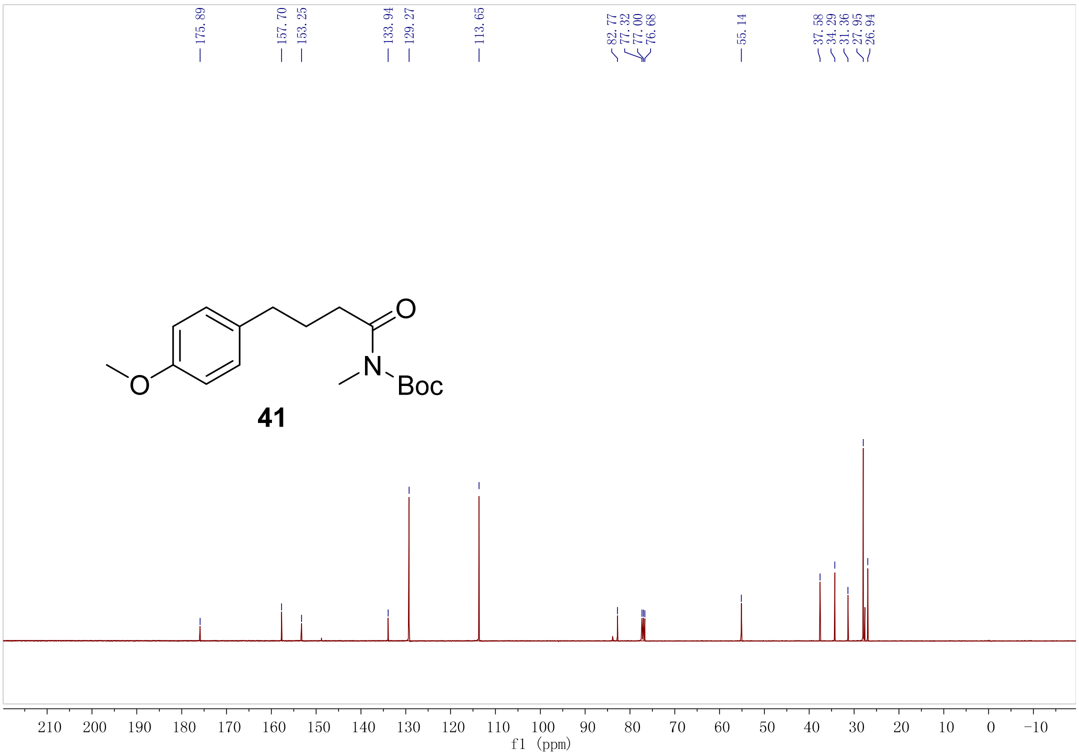

Supplementary Figure 42. ^{13}C NMR (400 MHz, $CDCl_3$) spectrum of compound **41**

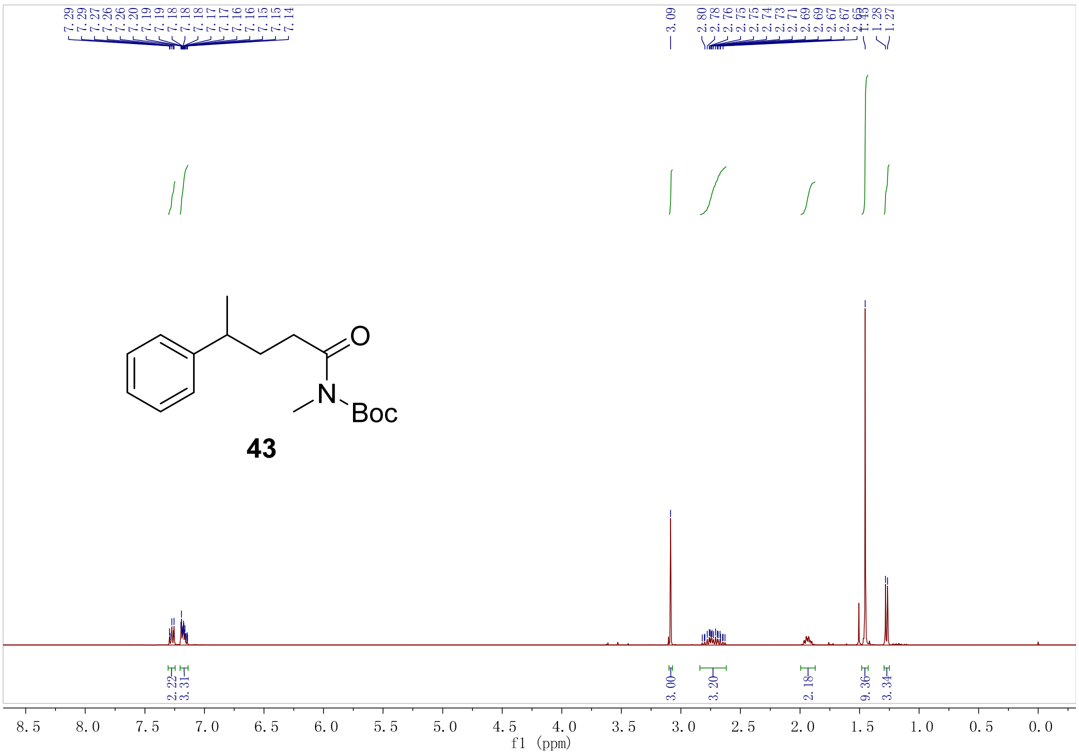

Supplementary Figure 43. ^{1}H NMR (400 MHz, $CDCl_3$) spectrum of compound **43**

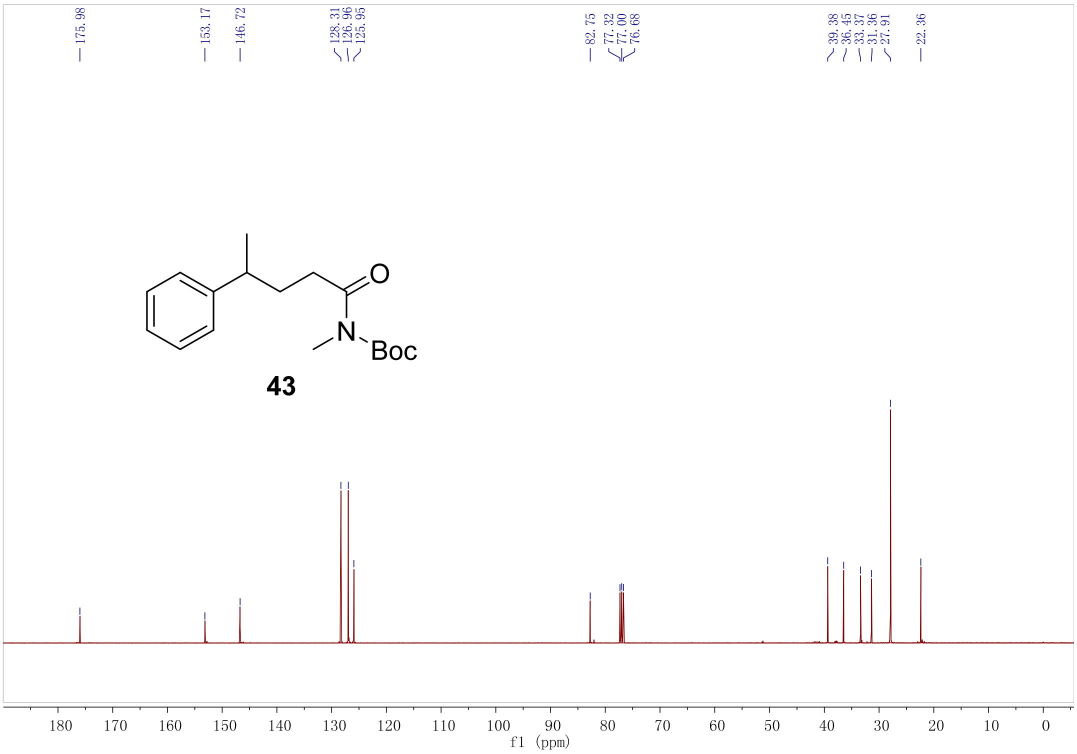

Supplementary Figure 44. ^{13}C NMR (400 MHz, $CDCl_3$) spectrum of compound **43**

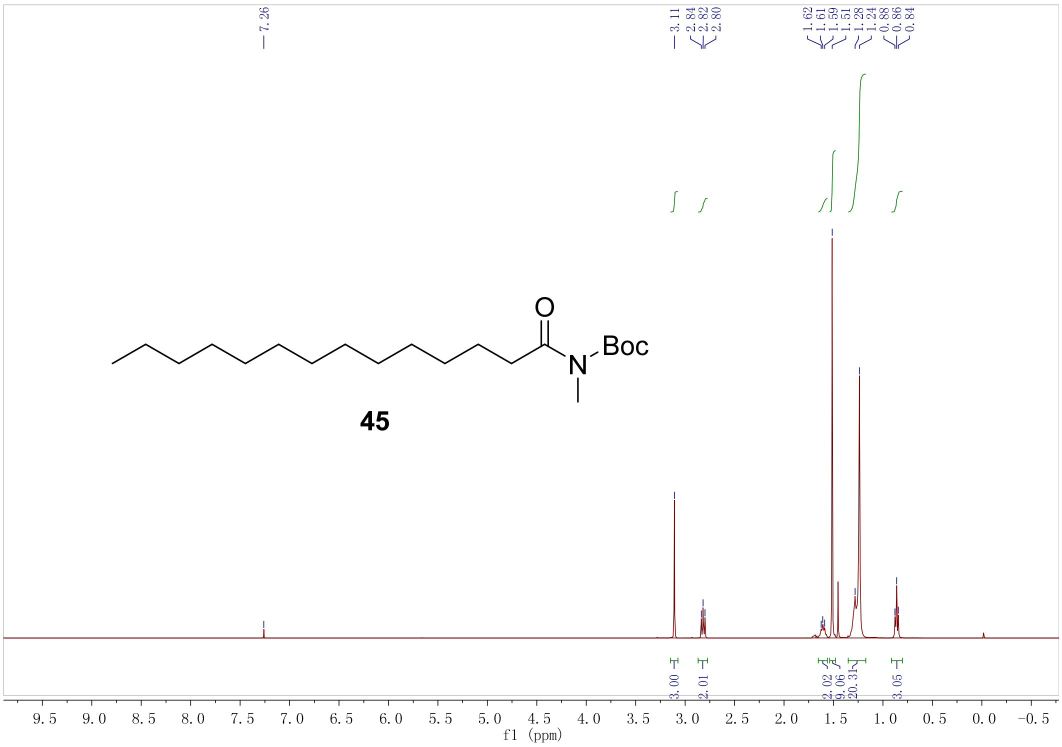

Supplementary Figure 45. ^{1}H NMR (400 MHz, $CDCl_3$) spectrum of compound **45**

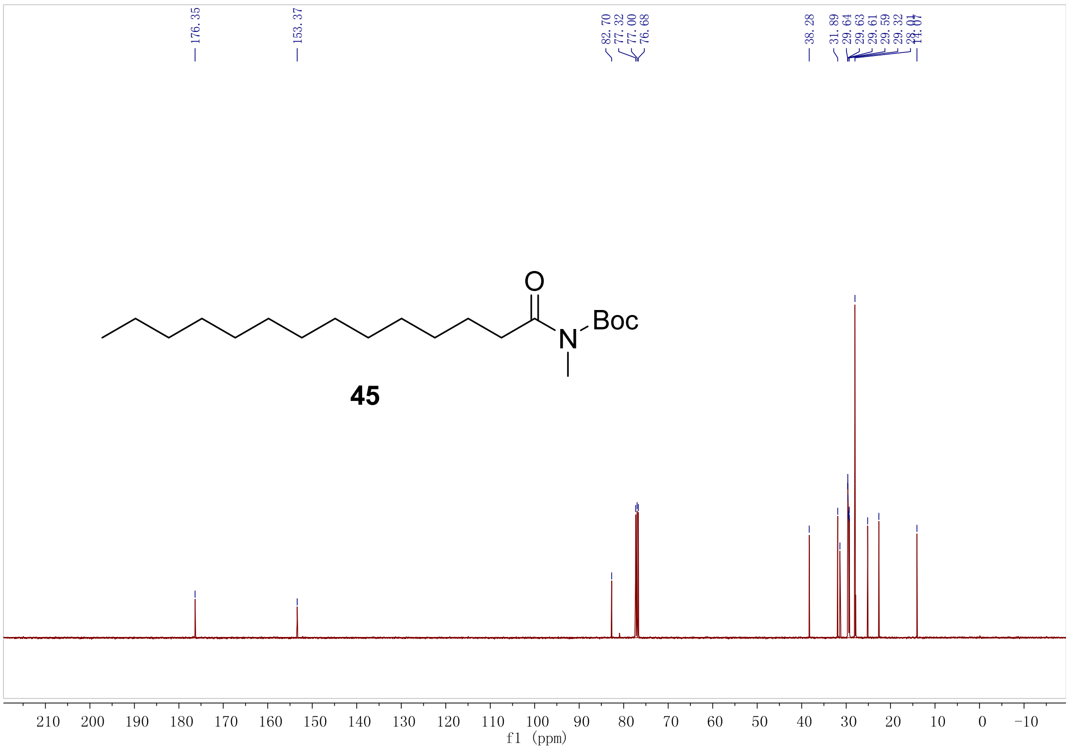

Supplementary Figure 46. ^{13}C NMR (400 MHz, $CDCl_3$) spectrum of compound **46**

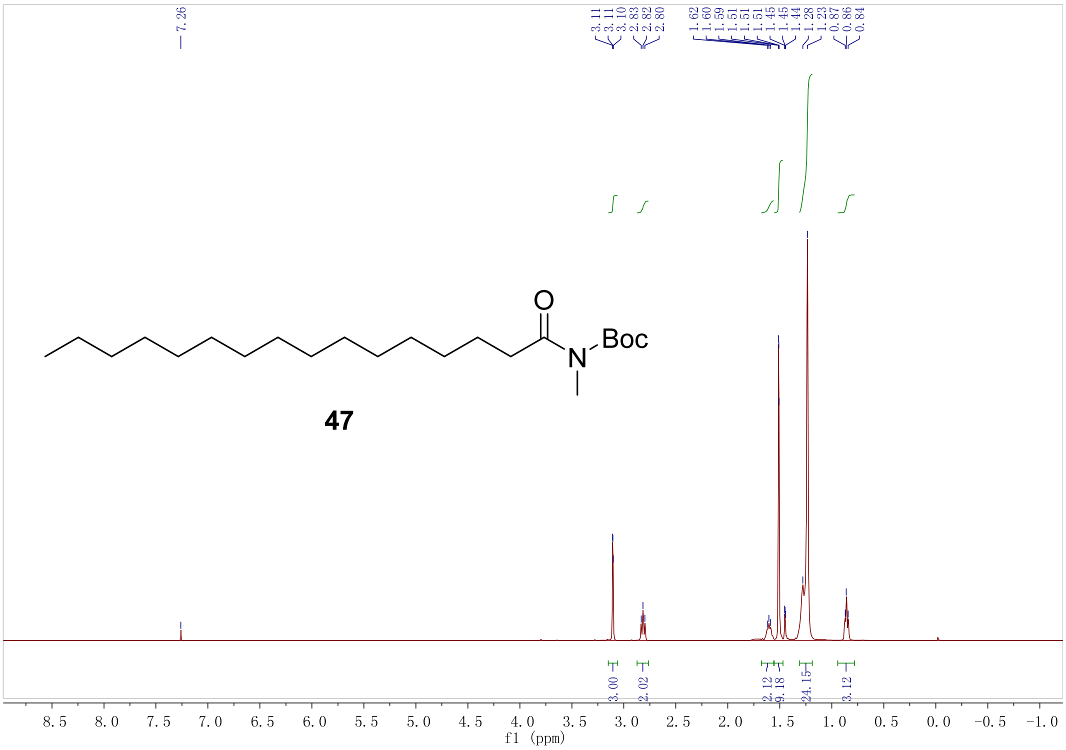

Supplementary Figure 47. ^{1}H NMR (400 MHz, $CDCl_3$) spectrum of compound **47**

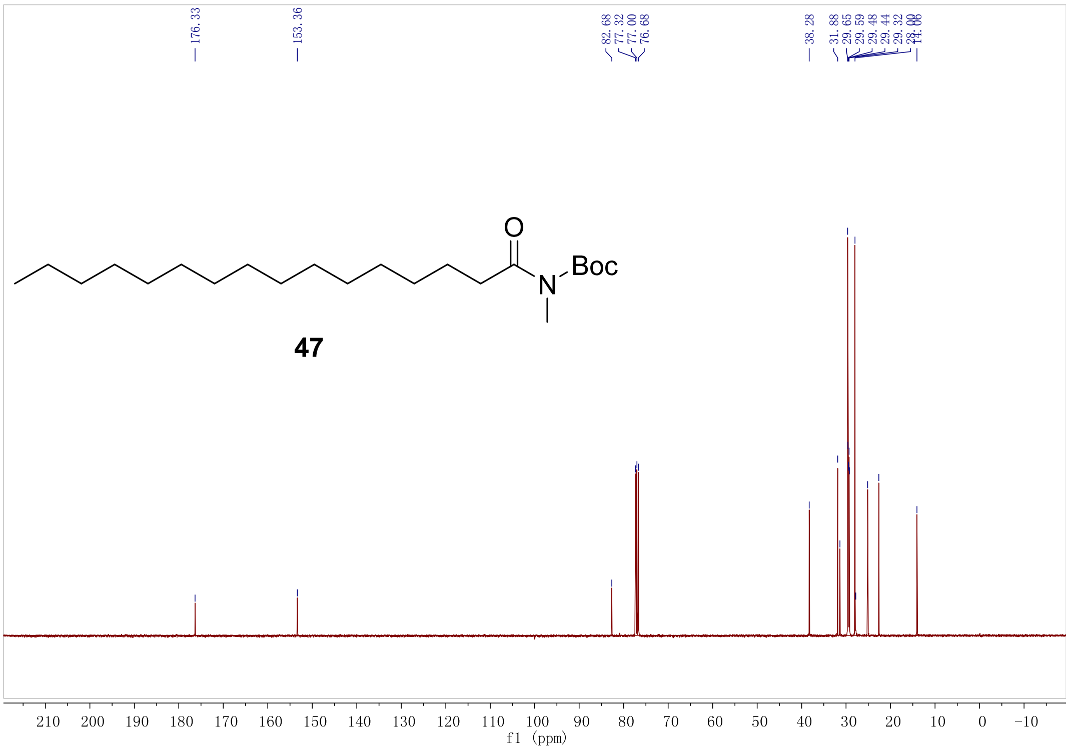

Supplementary Figure 48. ^{13}C NMR (400 MHz, $CDCl_3$) spectrum of compound **47**

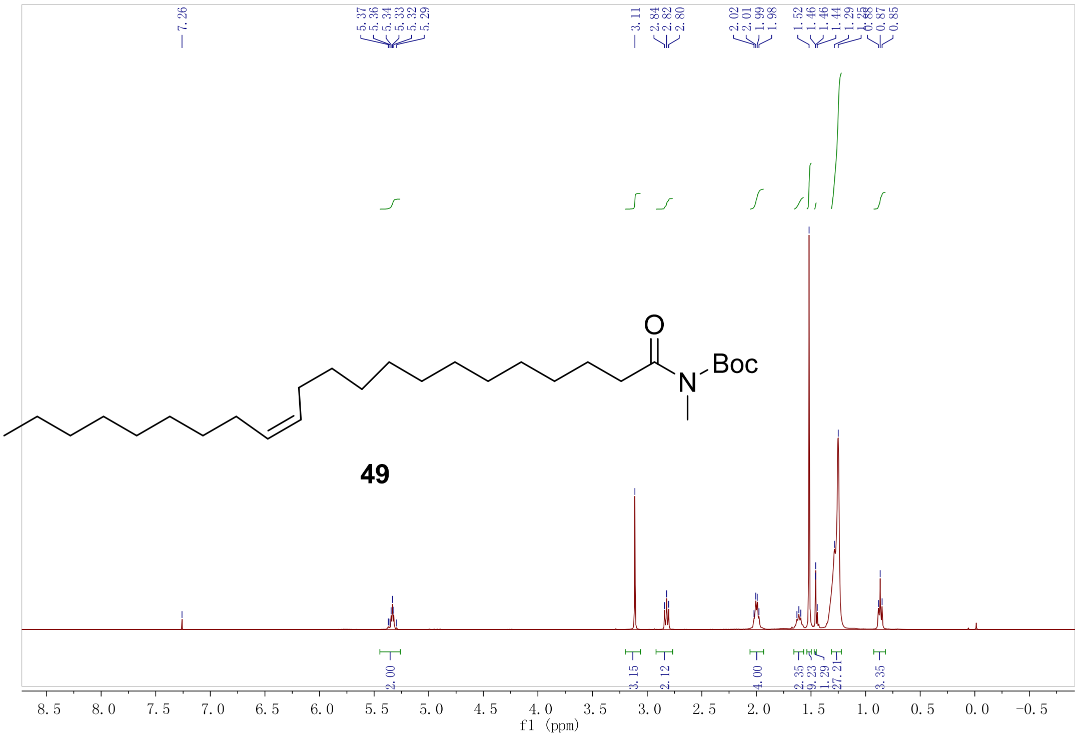

Supplementary Figure 49. ^{1}H NMR (400 MHz, $CDCl_3$) spectrum of compound **49**

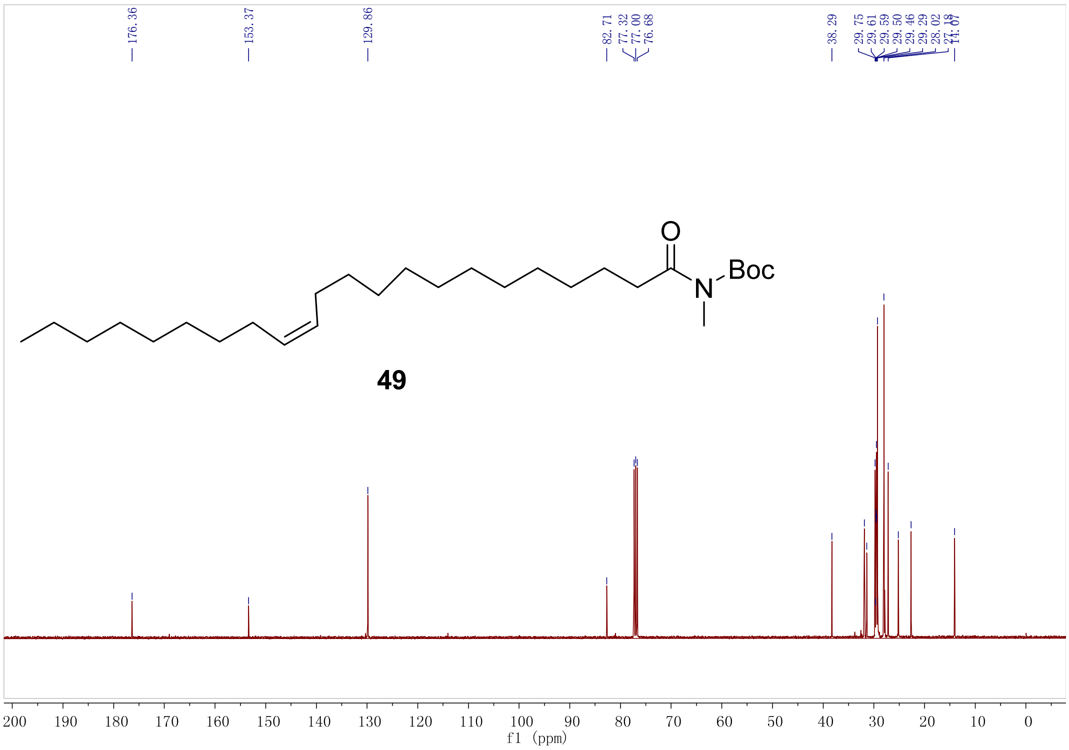

Supplementary Figure 50. ^{13}C NMR (400 MHz, $CDCl_3$) spectrum of compound **49**

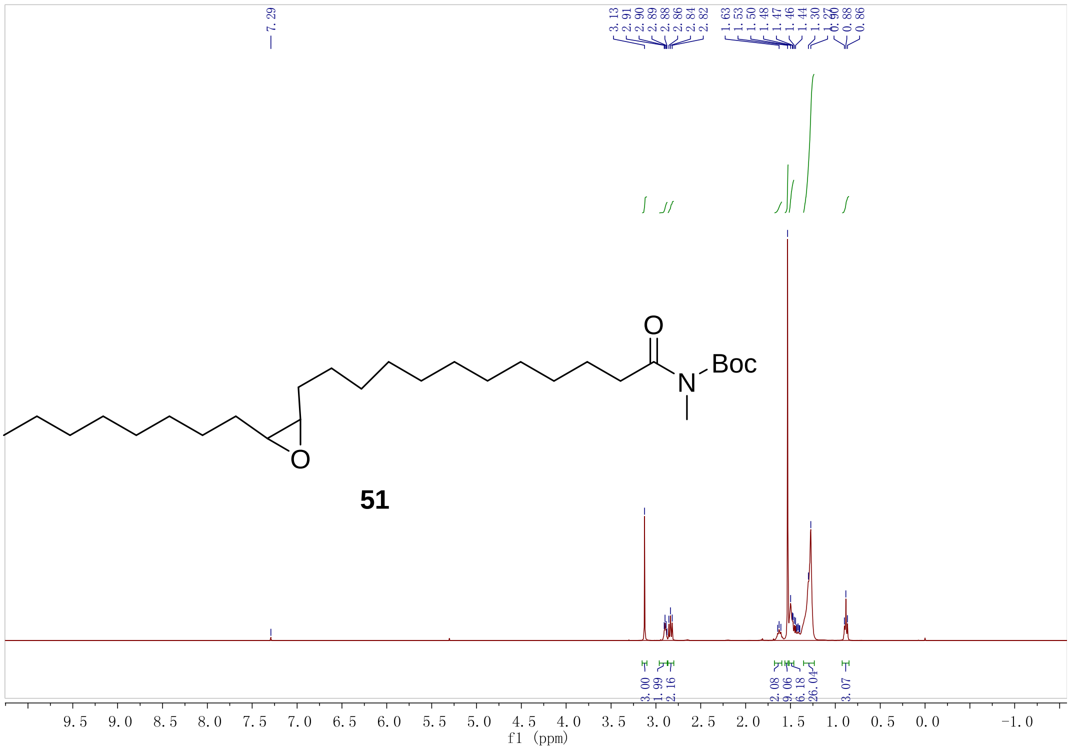

Supplementary Figure 51. ^{1}H NMR (400 MHz, $CDCl_3$) spectrum of compound **51**

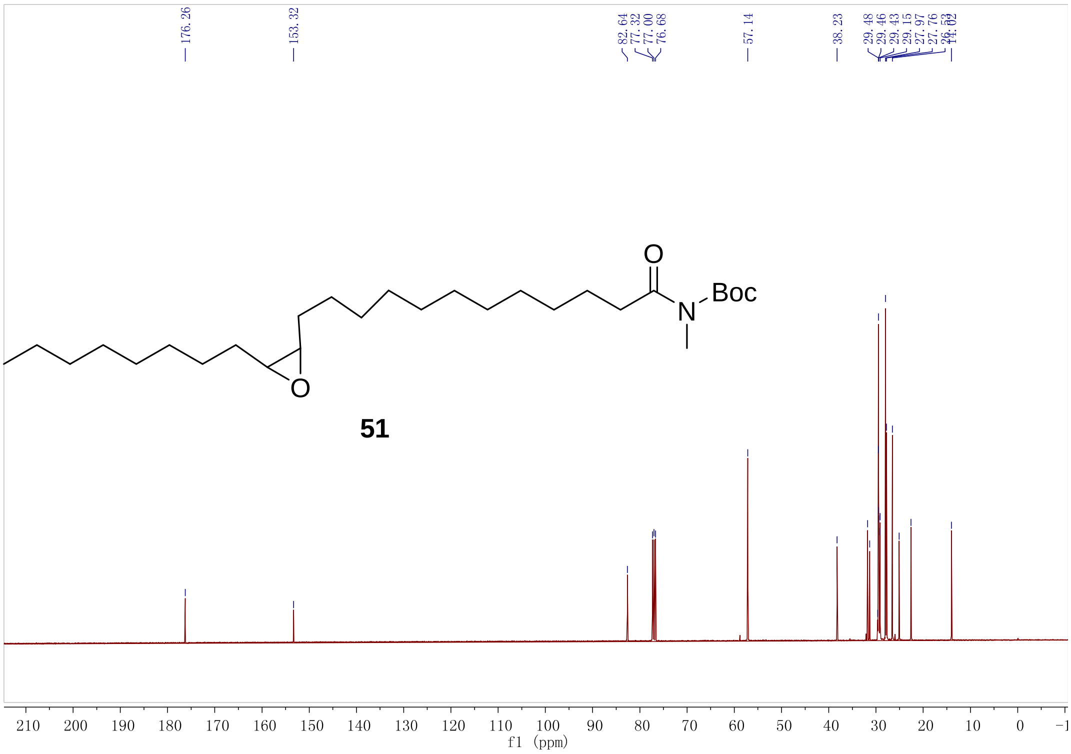

Supplementary Figure 52. ^{13}C NMR (400 MHz, $CDCl_3$) spectrum of compound **51**

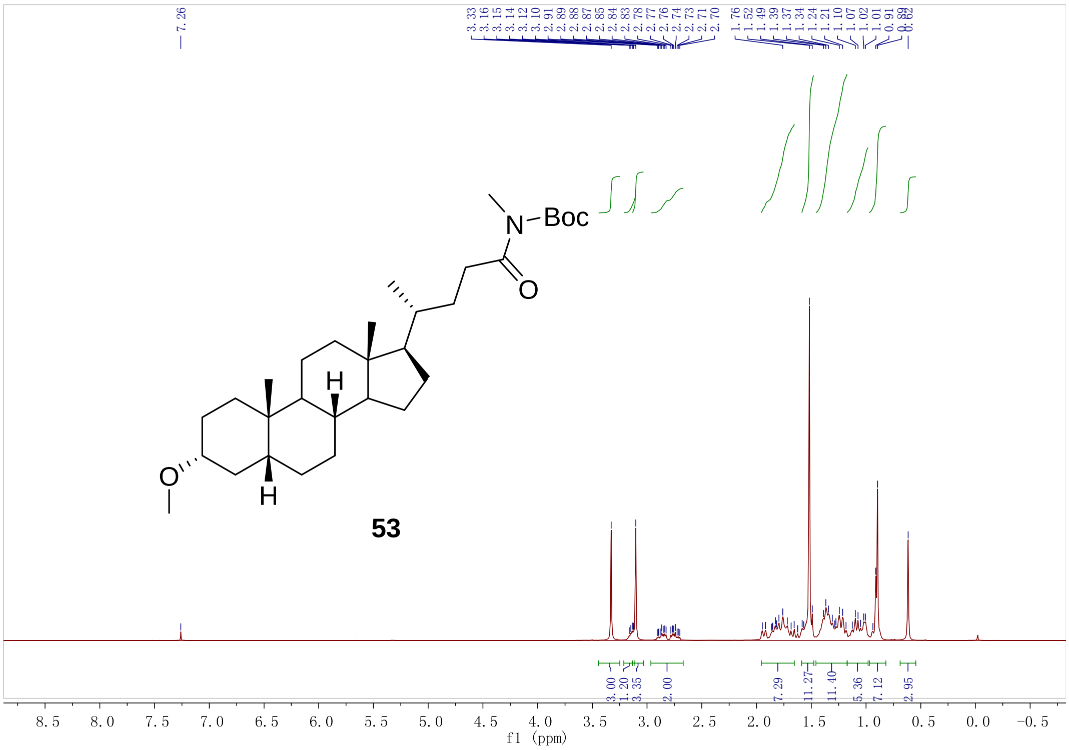

Supplementary Figure 53. ^{1}H NMR (400 MHz, $CDCl_3$) spectrum of compound **53**

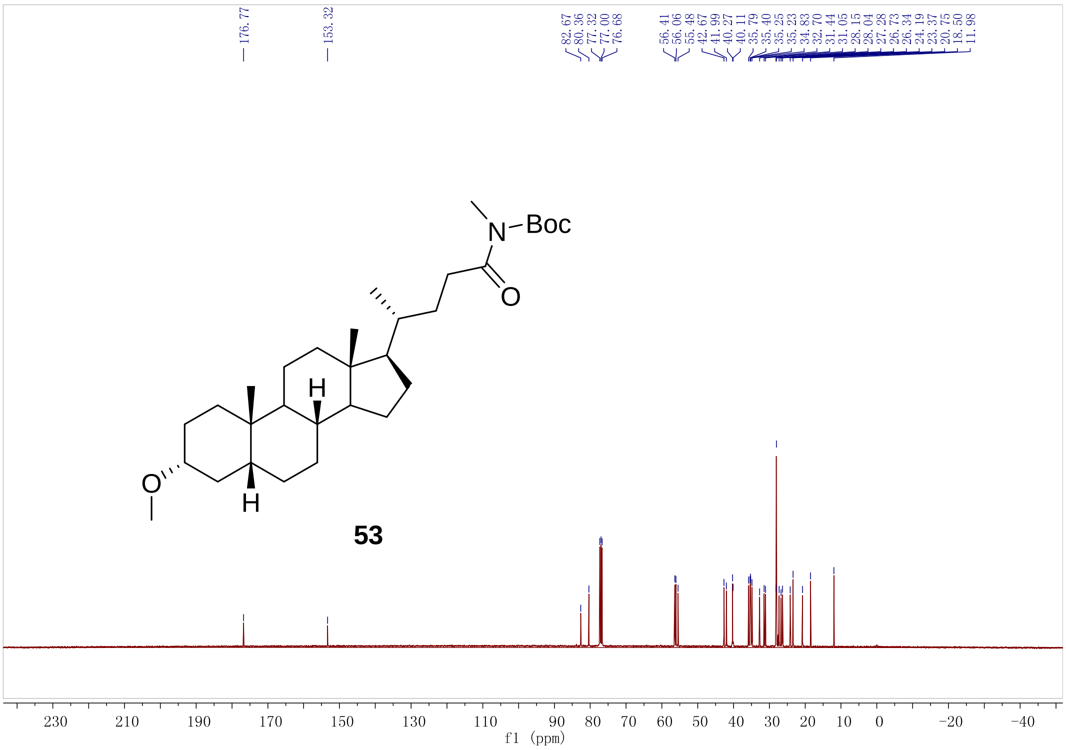

Supplementary Figure 54. ^{13}C NMR (400 MHz, $CDCl_3$) spectrum of compound **53**

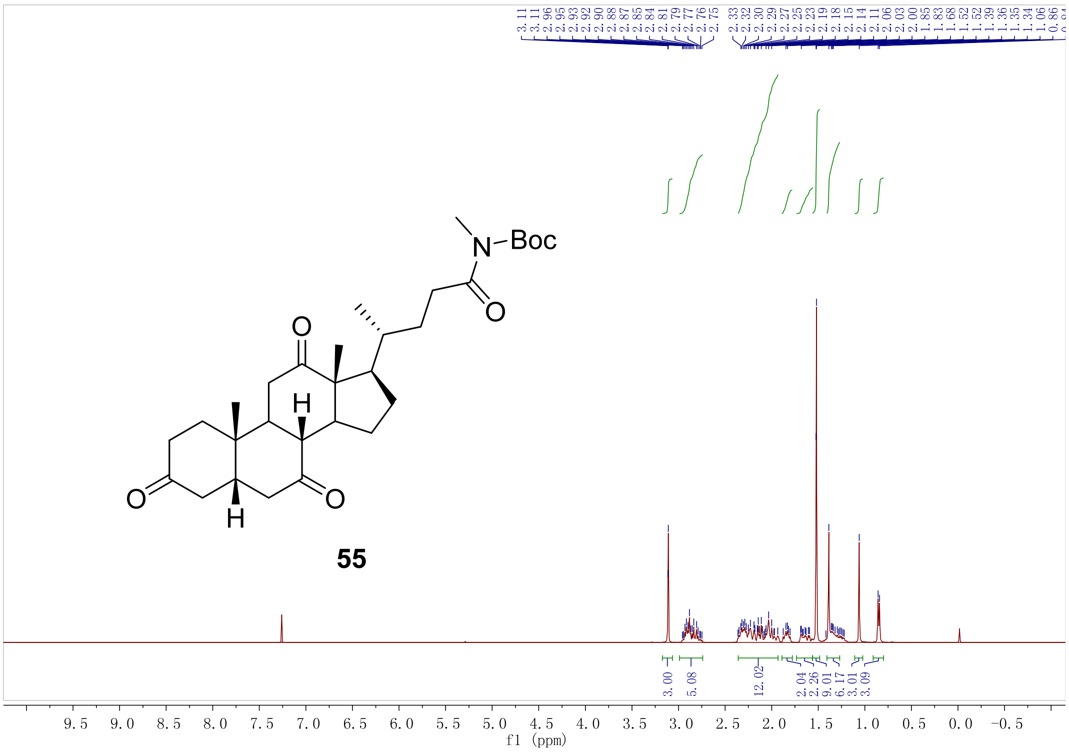

Supplementary Figure 55. ^{1}H NMR (400 MHz, $CDCl_3$) spectrum of compound **55**

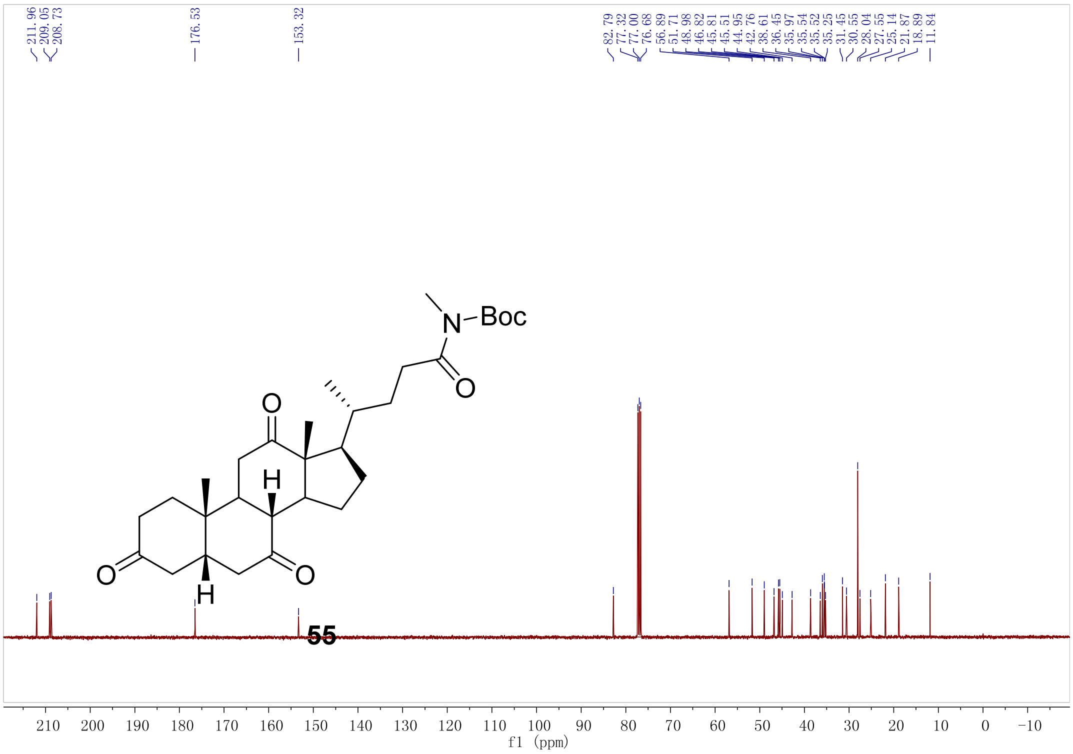

Supplementary Figure 56. ^{13}C NMR (400 MHz, $CDCl_3$) spectrum of compound **55**

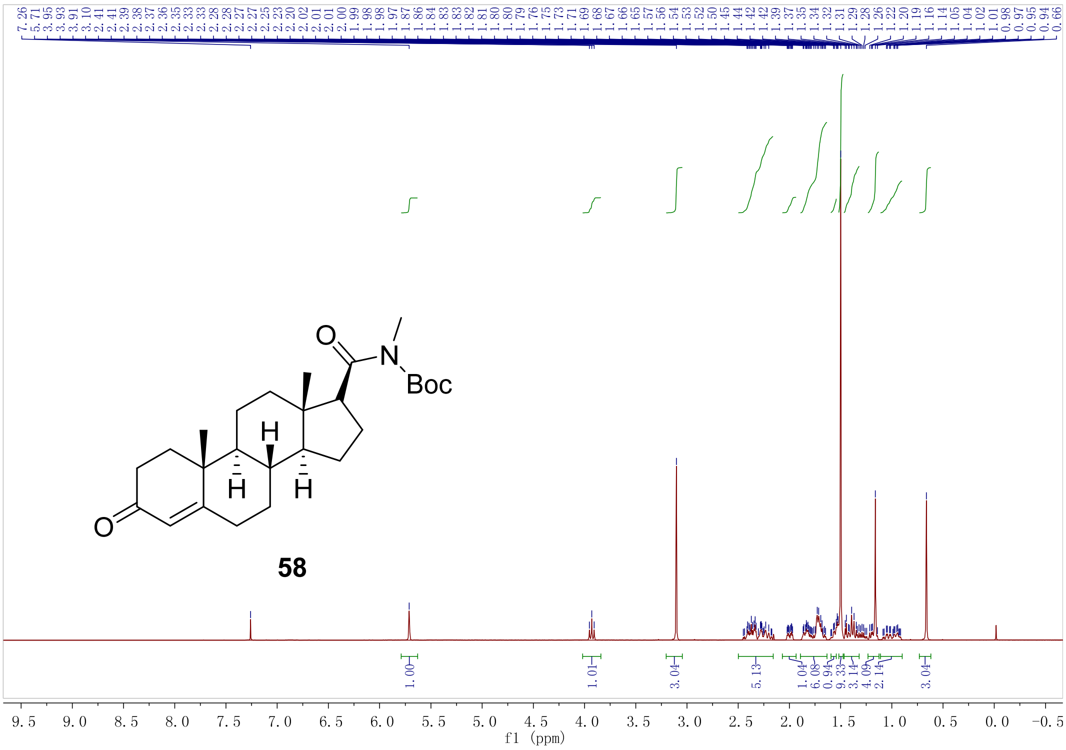

Supplementary Figure 57. ^{1}H NMR (400 MHz, $CDCl_3$) spectrum of compound **58**

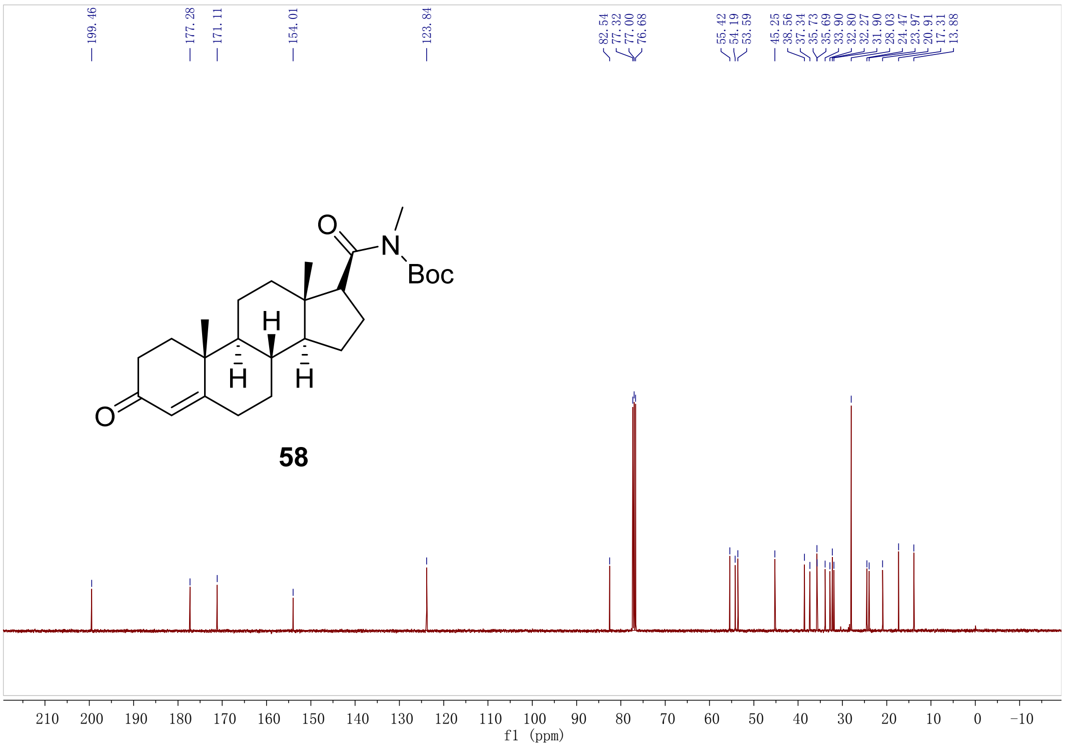

Supplementary Figure 58. ^{13}C NMR (400 MHz, $CDCl_3$) spectrum of compound **58**

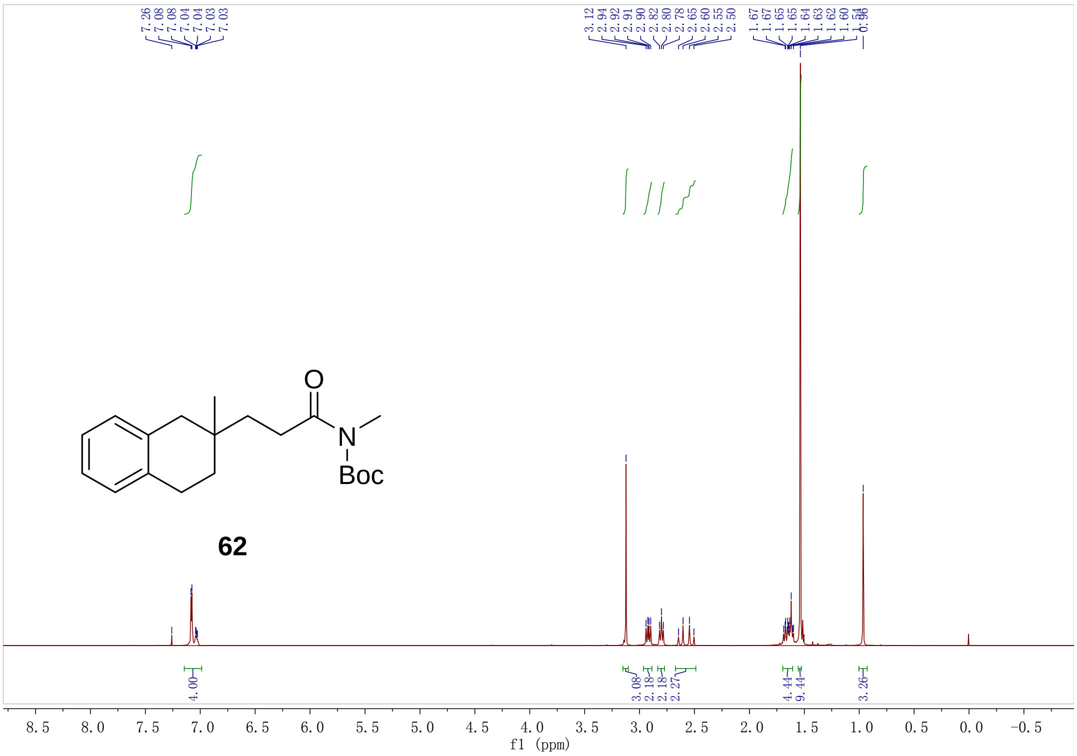

Supplementary Figure 59. ^{1}H NMR (400 MHz, $CDCl_3$) spectrum of compound **62**

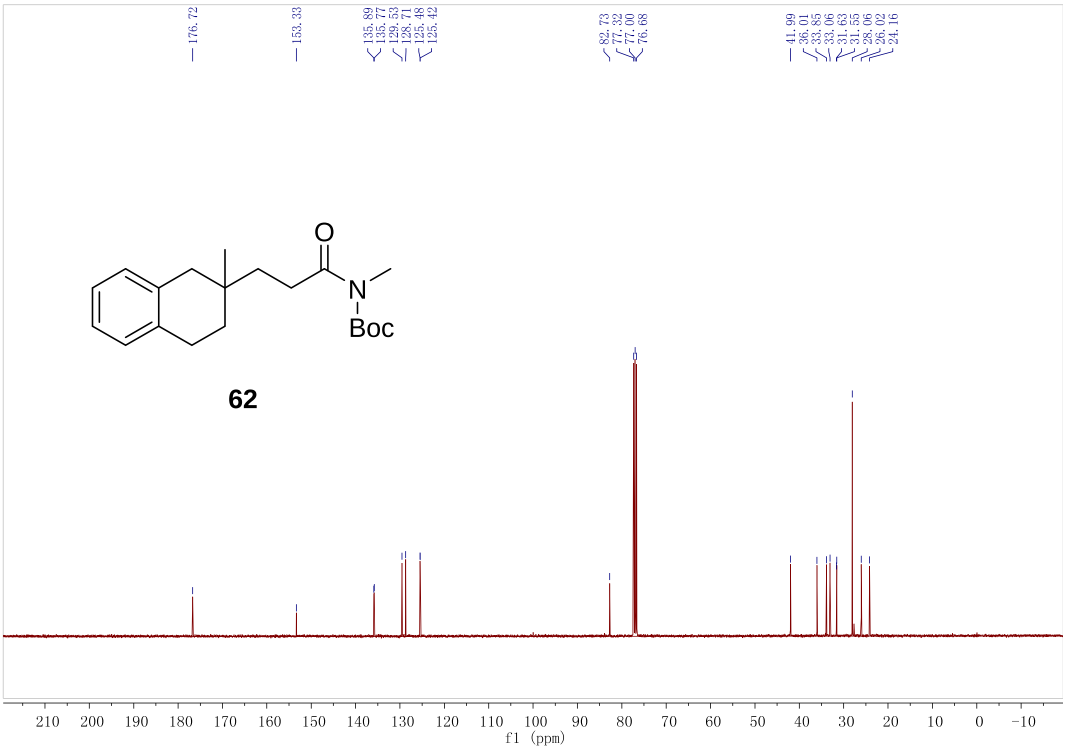

Supplementary Figure 60. ^{13}C NMR (400 MHz, $CDCl_3$) spectrum of compound **62**

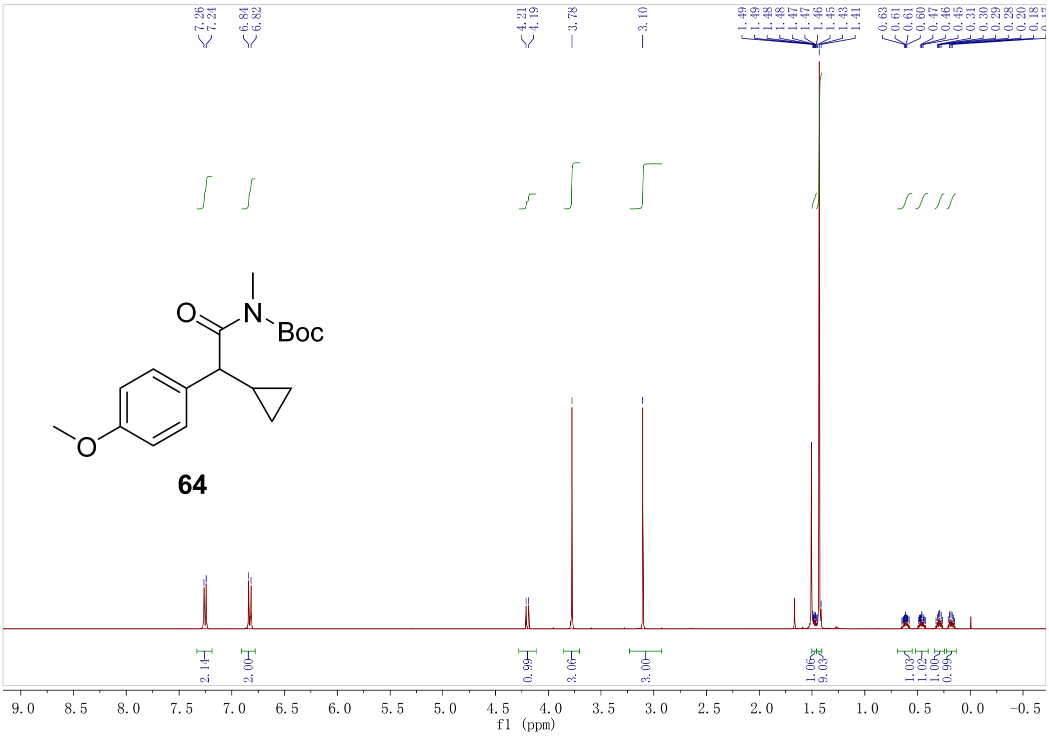

Supplementary Figure 61. ^{1}H NMR (400 MHz, $CDCl_3$) spectrum of compound **64**

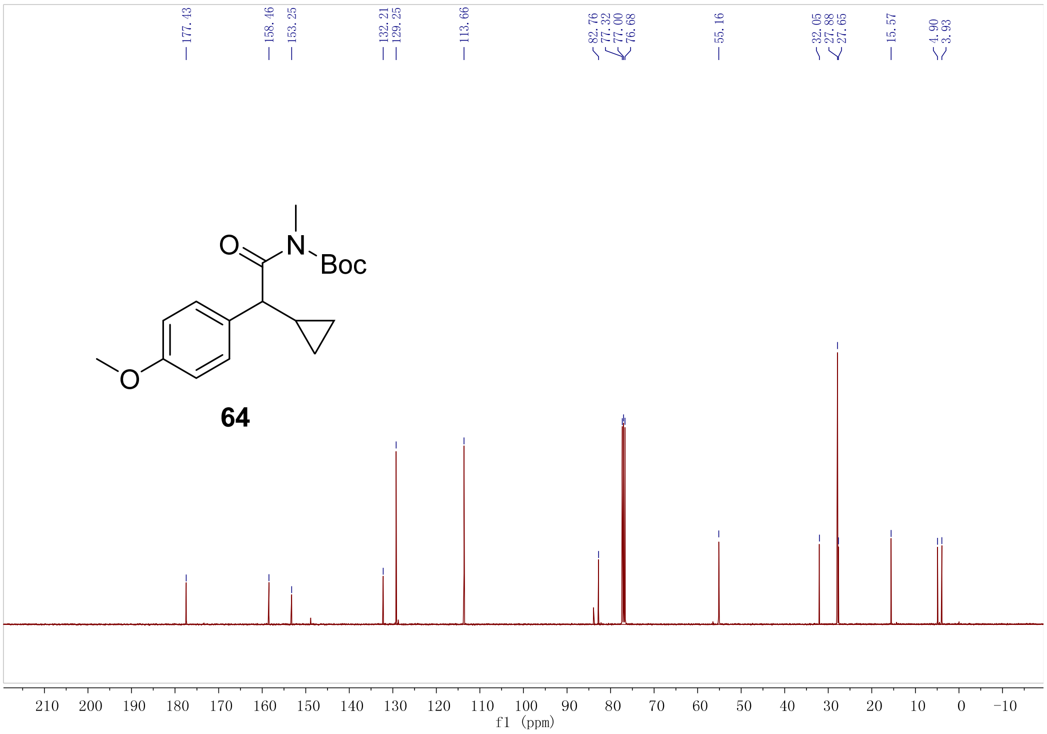

Supplementary Figure 62. ^{13}C NMR (400 MHz, $CDCl_3$) spectrum of compound **64**

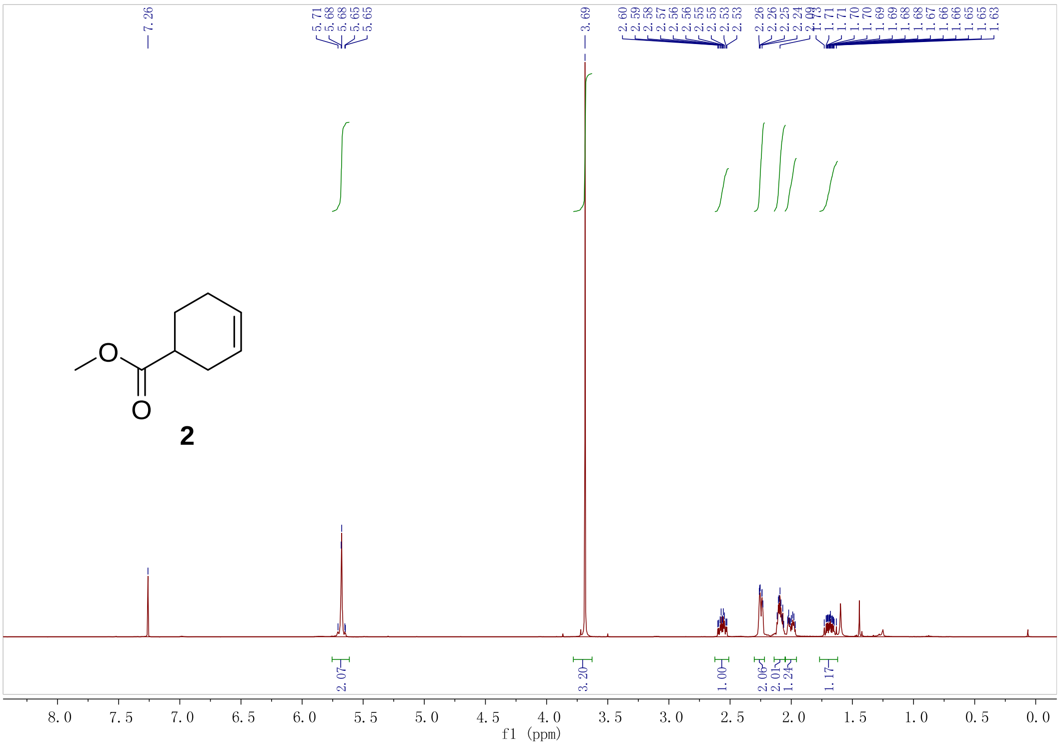

Supplementary Figure 63. ^{1}H NMR (400 MHz, $CDCl_3$) spectrum of compound **2**

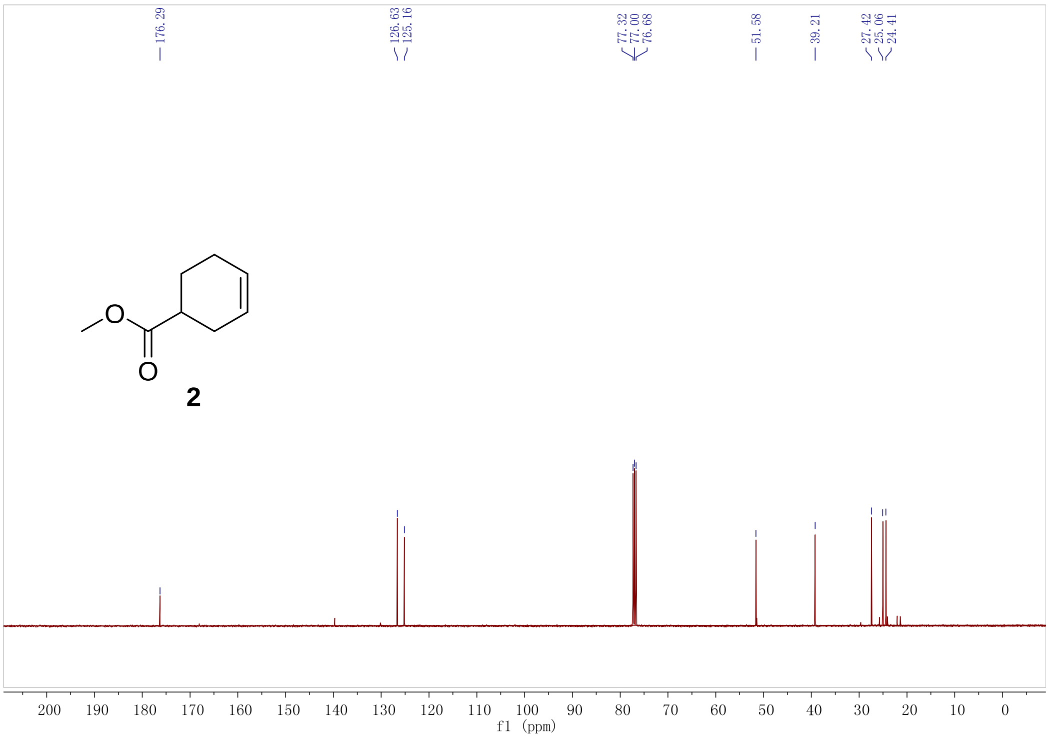

Supplementary Figure 64. ^{13}C NMR (400 MHz, $CDCl_3$) spectrum of compound **2**

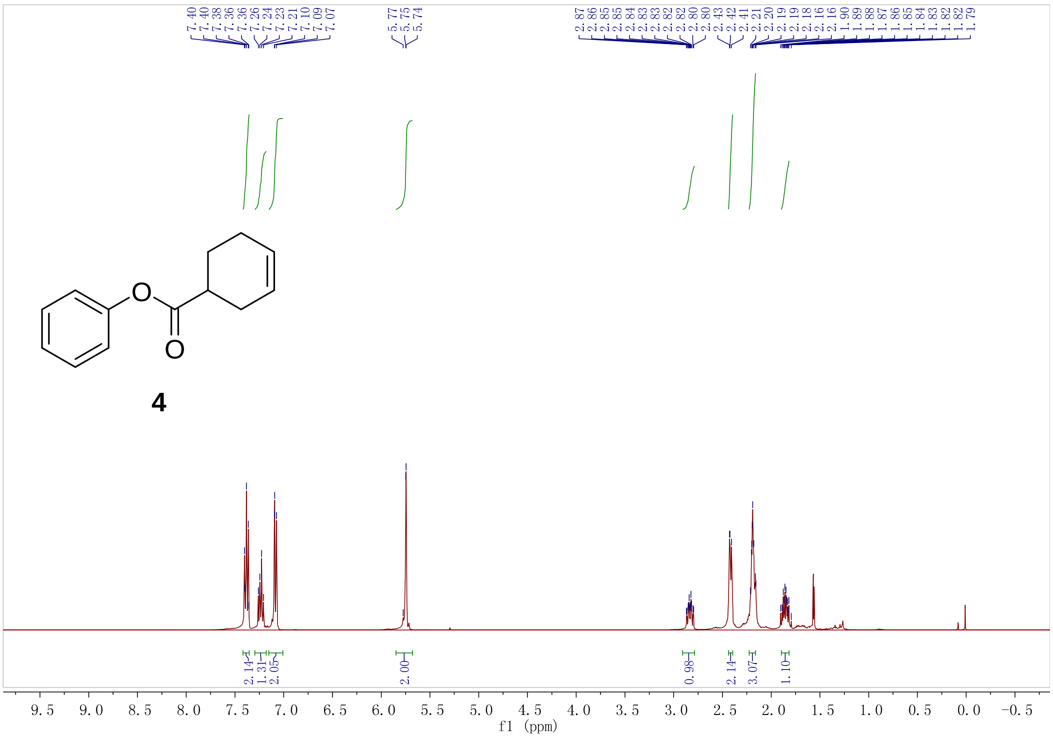

Supplementary Figure 65. ^{1}H NMR (400 MHz, $CDCl_3$) spectrum of compound **4**

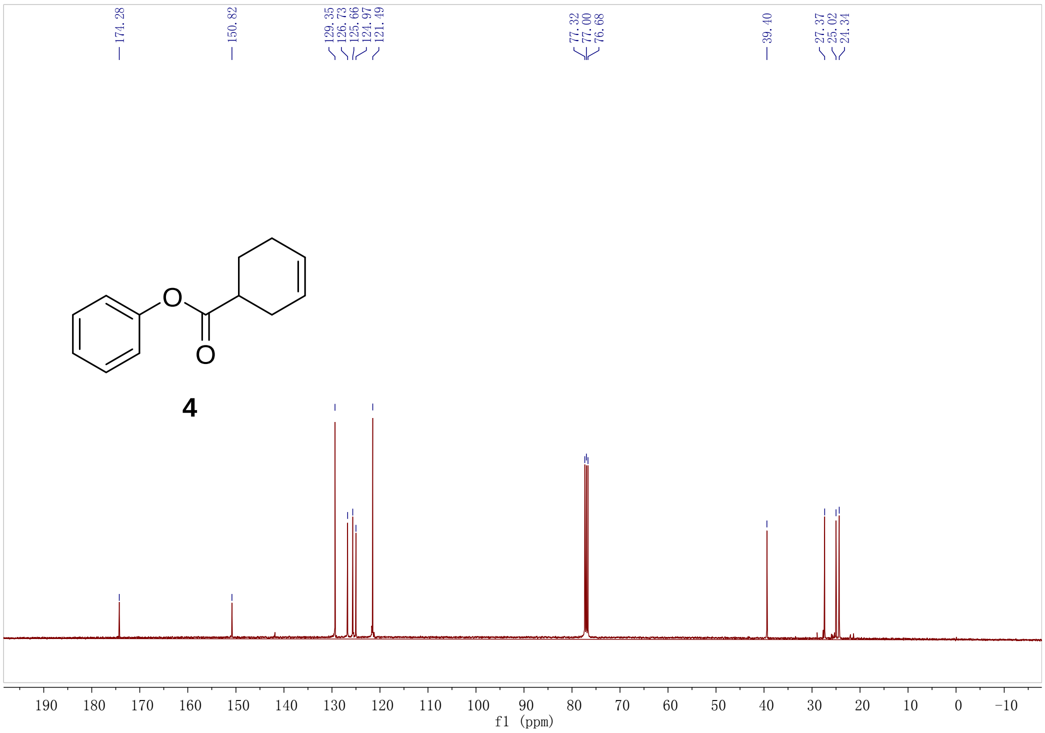

Supplementary Figure 66. ^{13}C NMR (400 MHz, $CDCl_3$) spectrum of compound **4**

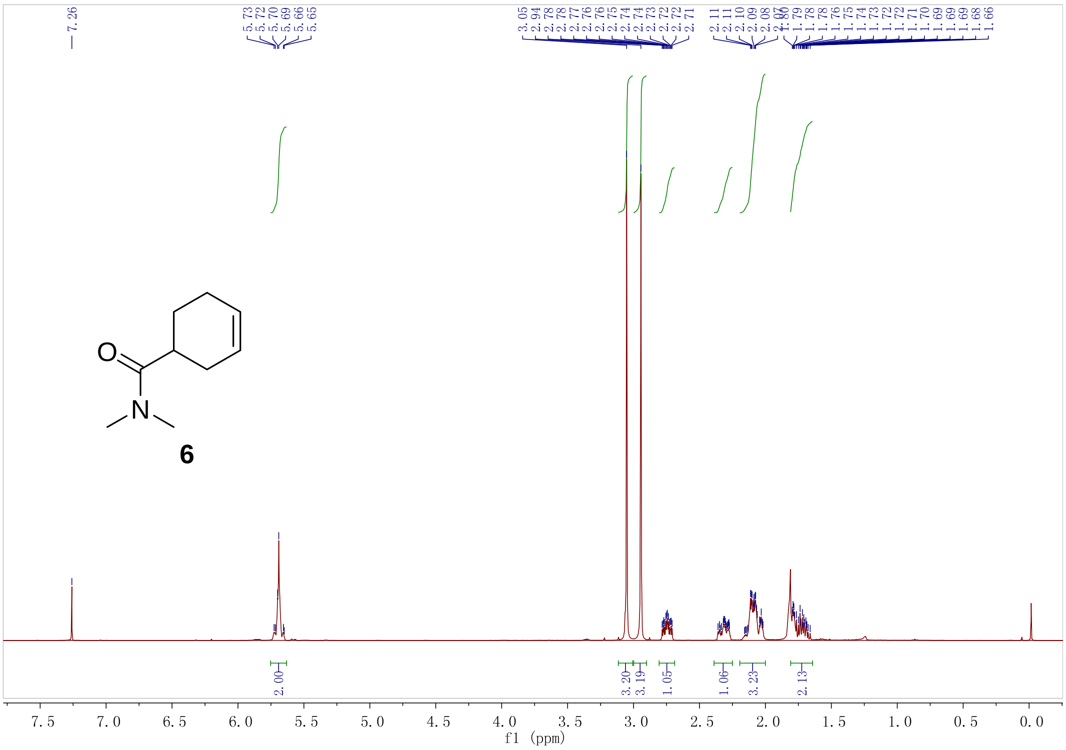

Supplementary Figure 67. ^{1}H NMR (400 MHz, $CDCl_3$) spectrum of compound **6**

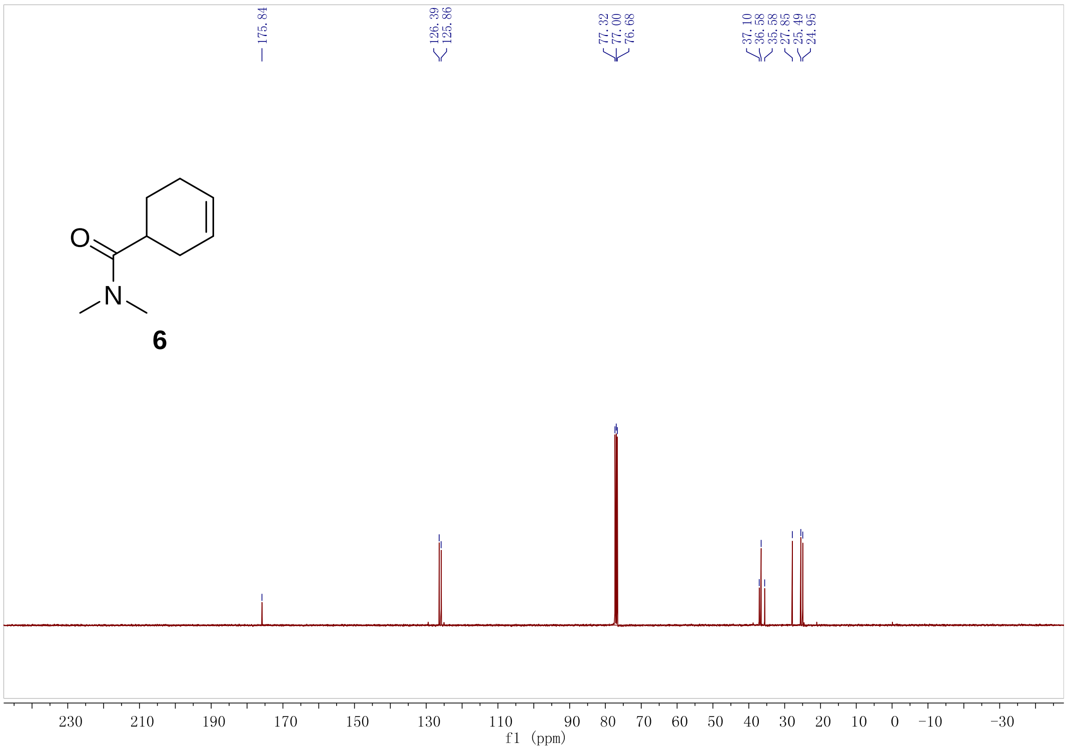

Supplementary Figure 68. ^{13}C NMR (400 MHz, $CDCl_3$) spectrum of compound **6**

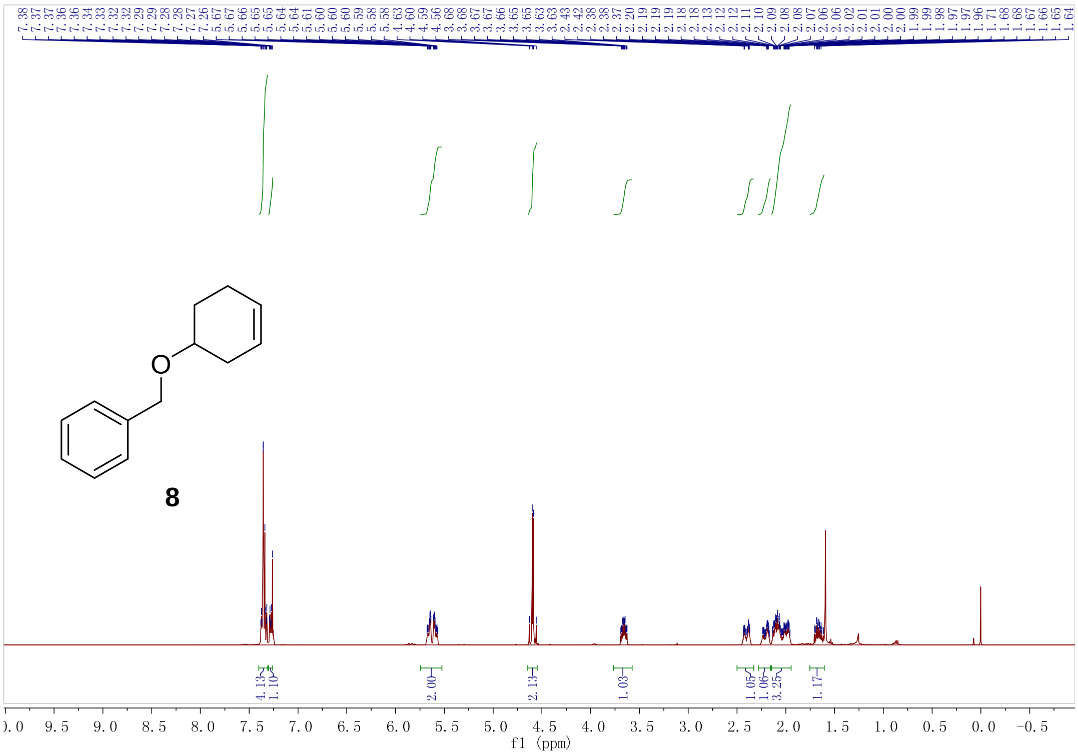

Supplementary Figure 69. ^{1}H NMR (400 MHz, $CDCl_3$) spectrum of compound **8**

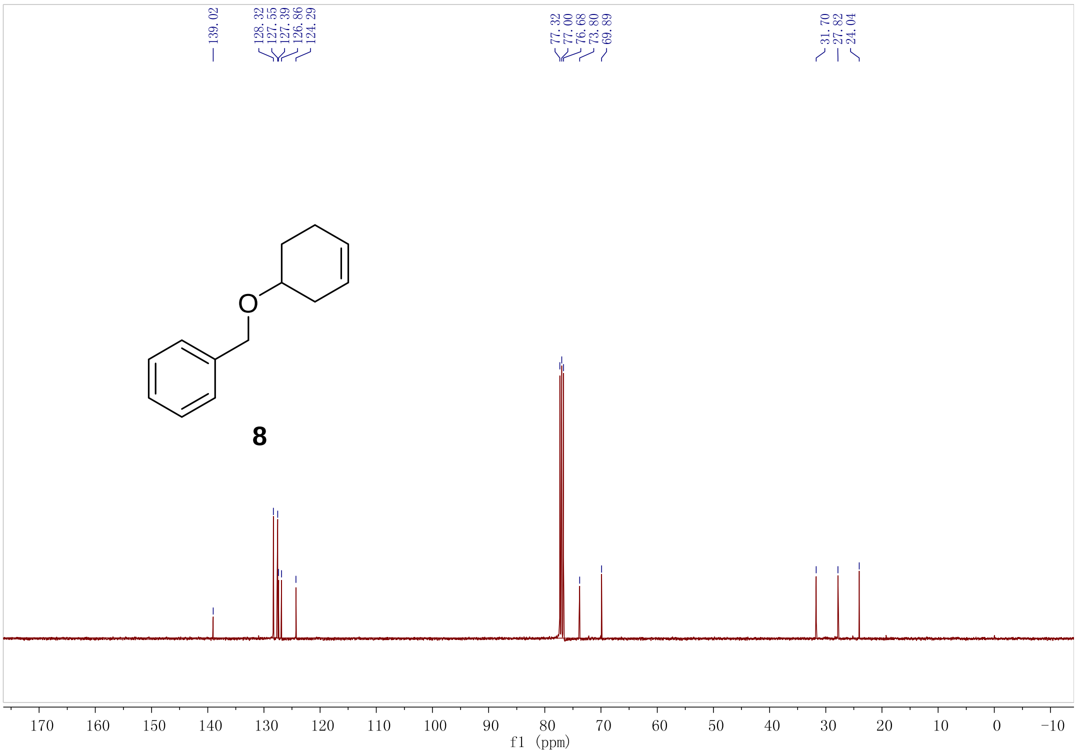

Supplementary Figure 70. ^{13}C NMR (400 MHz, $CDCl_3$) spectrum of compound **8**

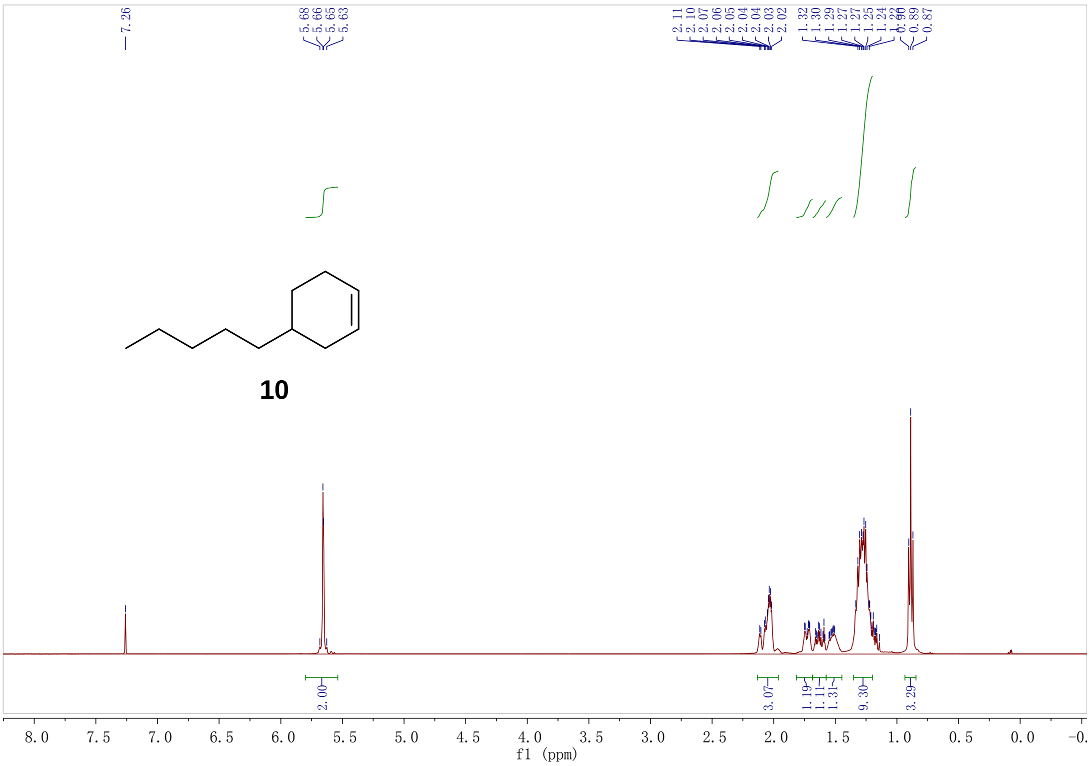

Supplementary Figure 71. ^{1}H NMR (400 MHz, $CDCl_3$) spectrum of compound **10**

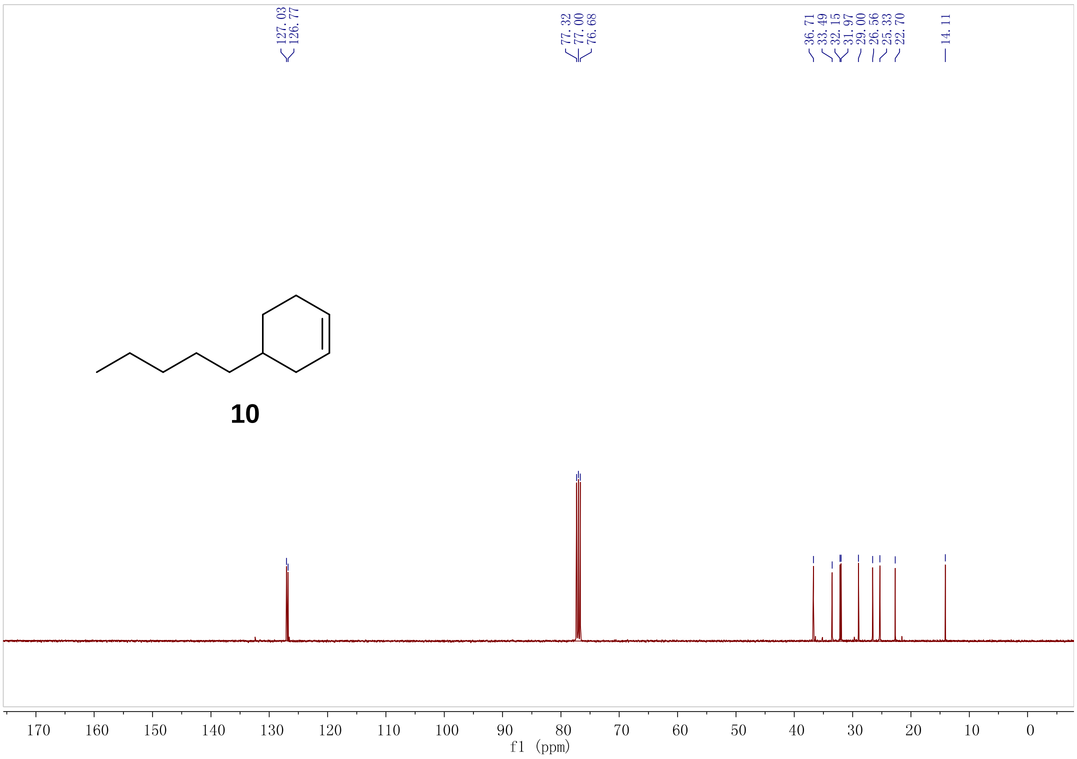

Supplementary Figure 72. ^{13}C NMR (400 MHz, $CDCl_3$) spectrum of compound **10**

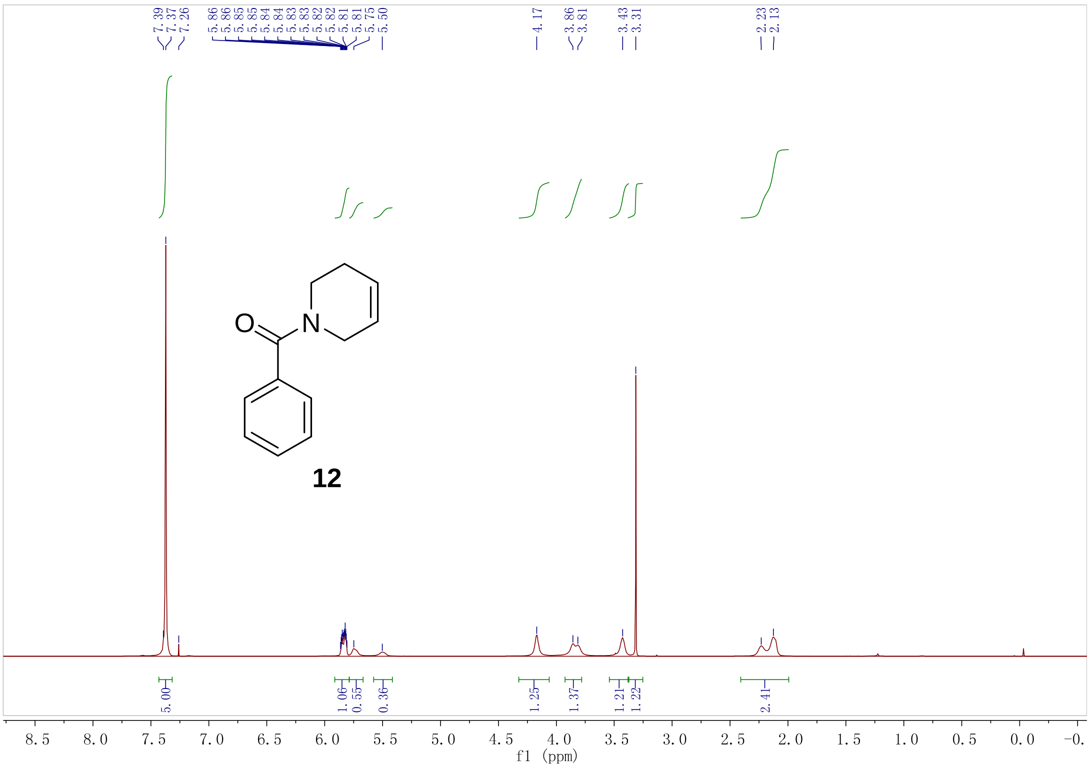

Supplementary Figure 73. ^{1}H NMR (400 MHz, $CDCl_3$) spectrum of compound **12**

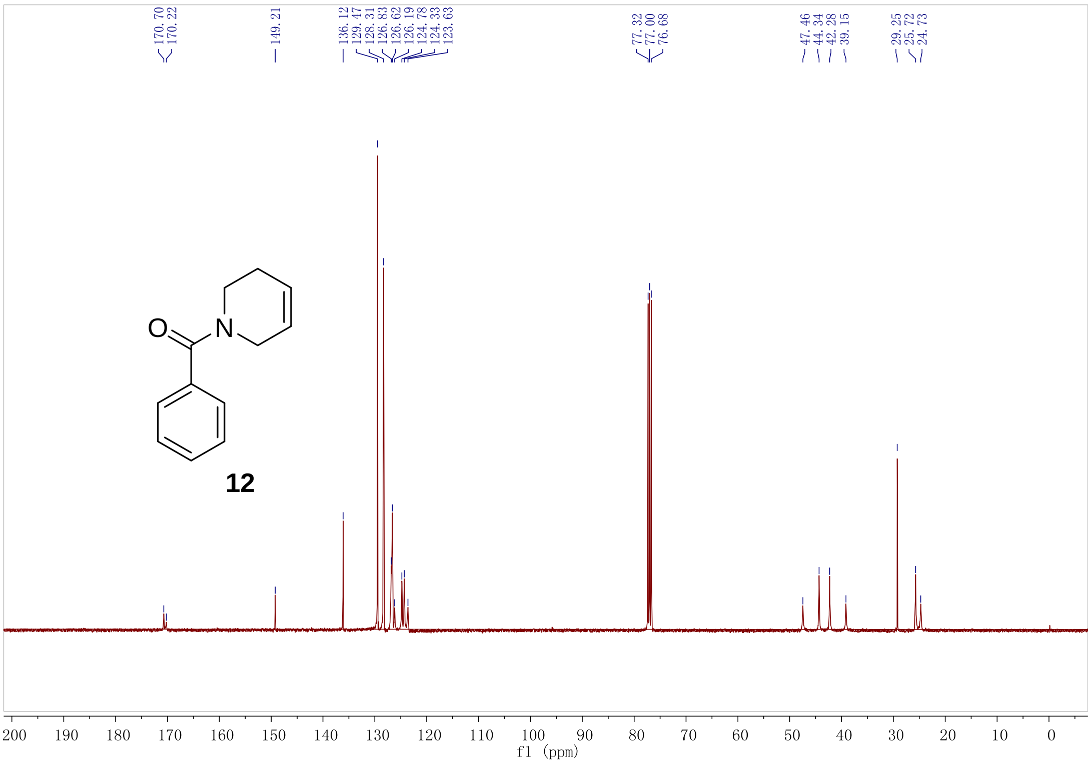

Supplementary Figure 74. ^{13}C NMR (400 MHz, $CDCl_3$) spectrum of compound **12**

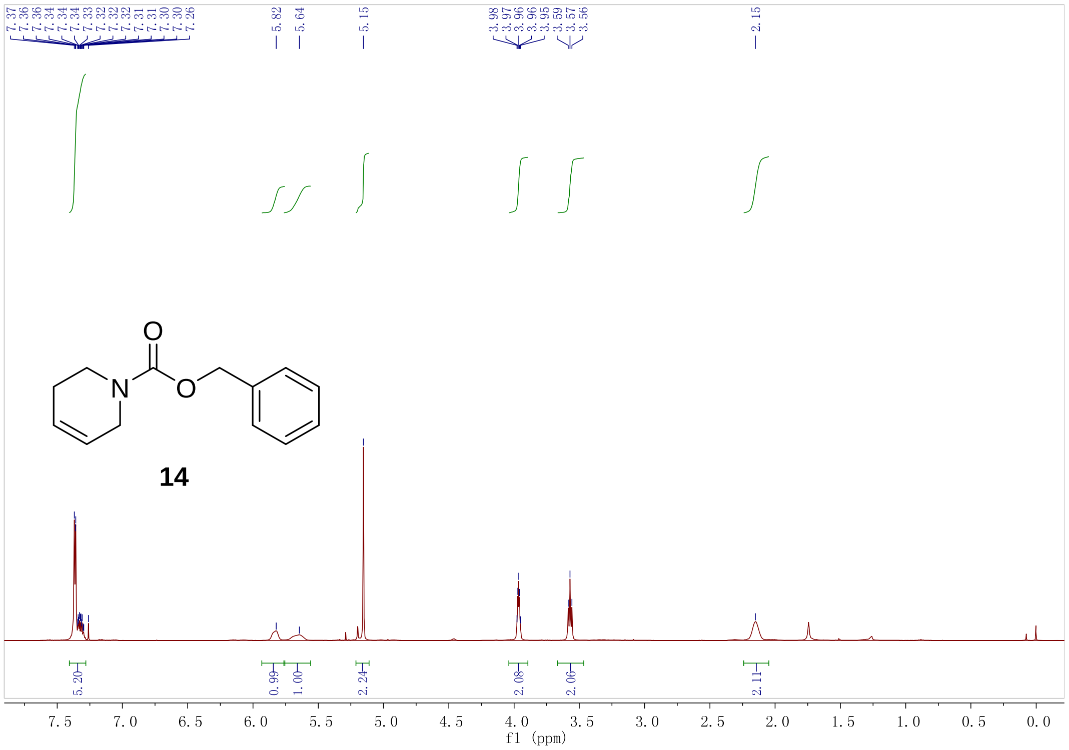

Supplementary Figure 75. ^{1}H NMR (400 MHz, $CDCl_3$) spectrum of compound **14**

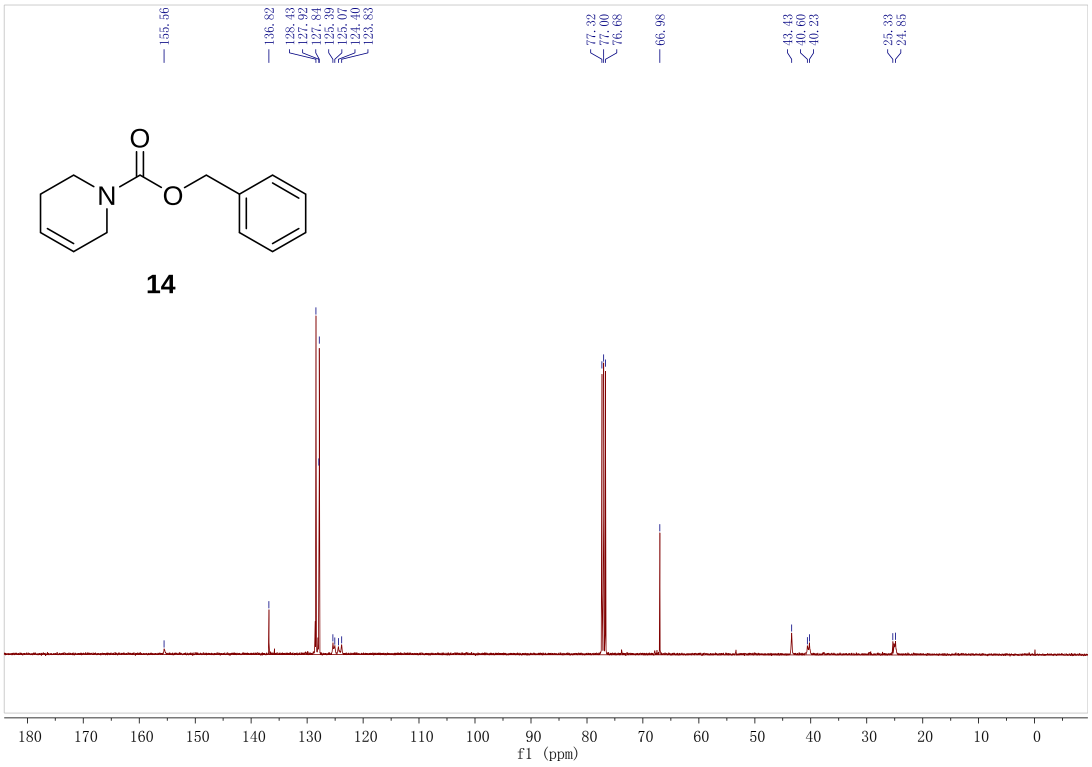

Supplementary Figure 76. ^{13}C NMR (400 MHz, $CDCl_3$) spectrum of compound **14**

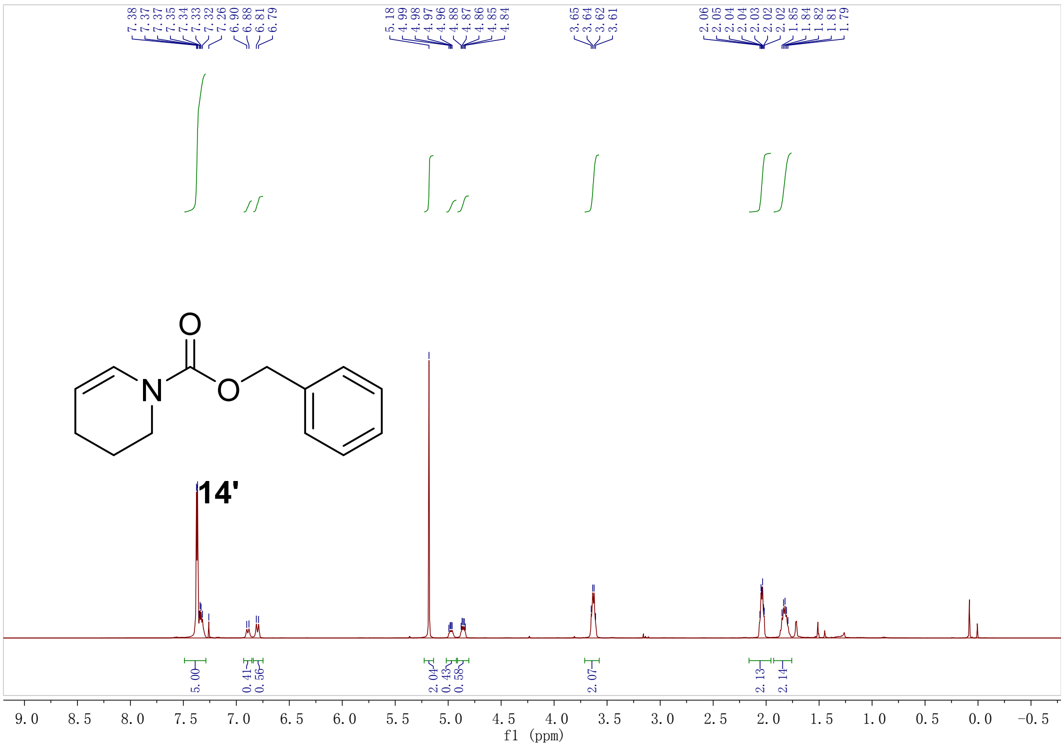

Supplementary Figure 77. ^{1}H NMR (400 MHz, $CDCl_3$) spectrum of compound **14'**

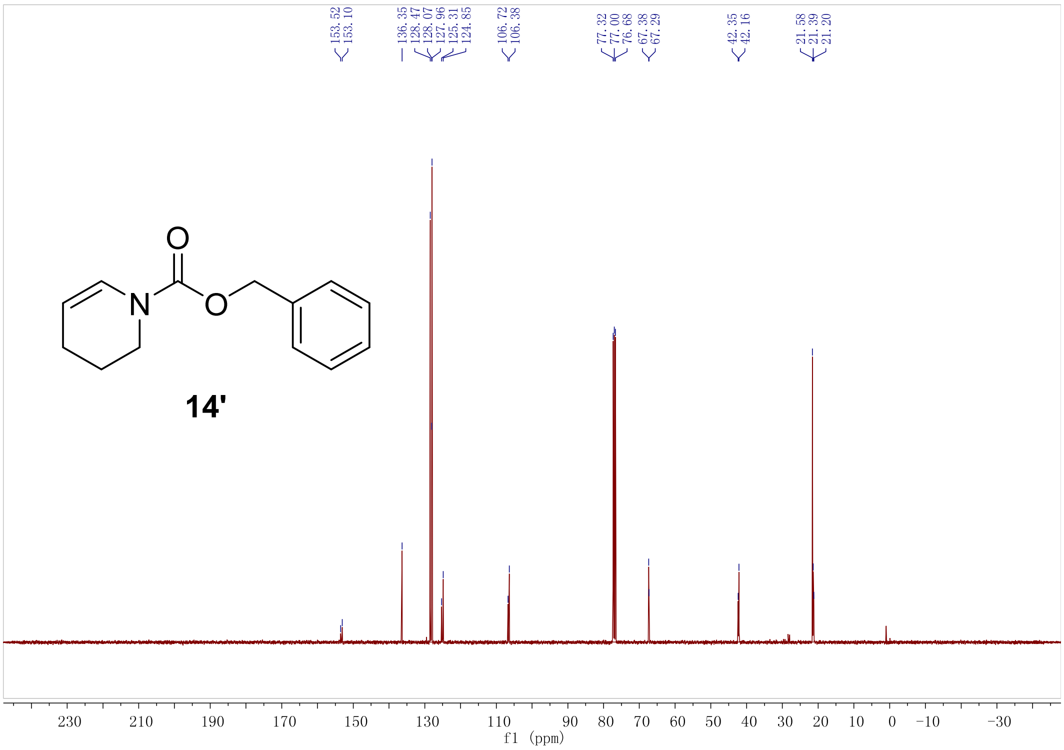

Supplementary Figure 78. ^{13}C NMR (400 MHz, $CDCl_3$) spectrum of compound **14'**

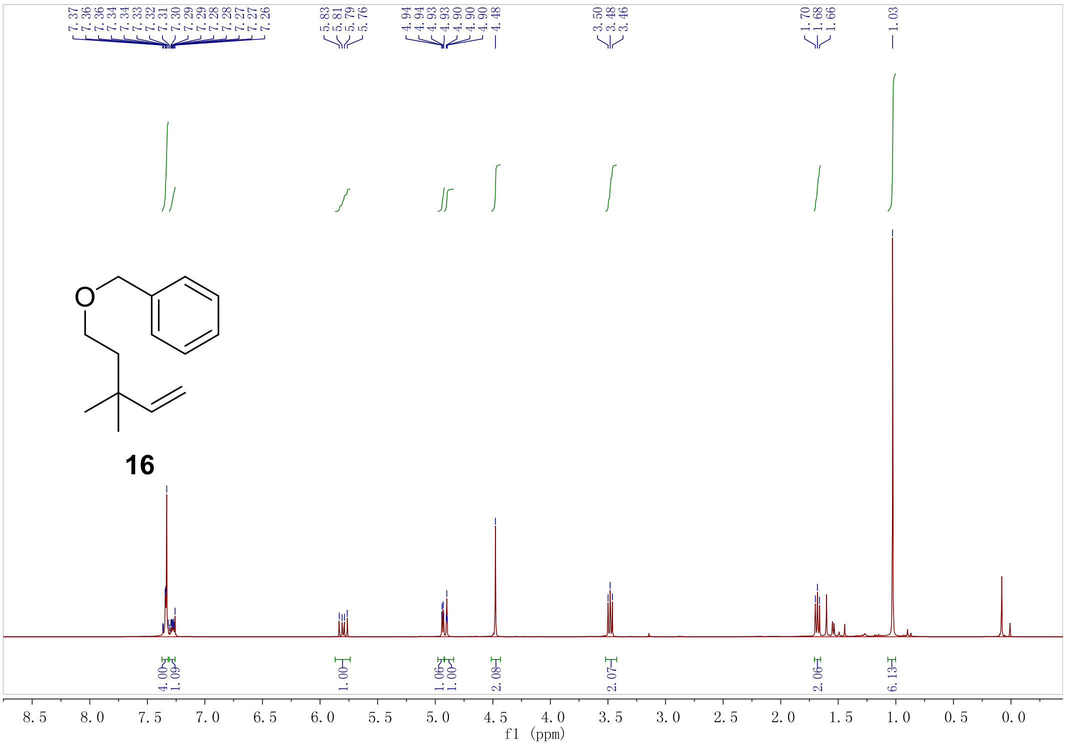

Supplementary Figure 79. ^{1}H NMR (400 MHz, $CDCl_3$) spectrum of compound **16**

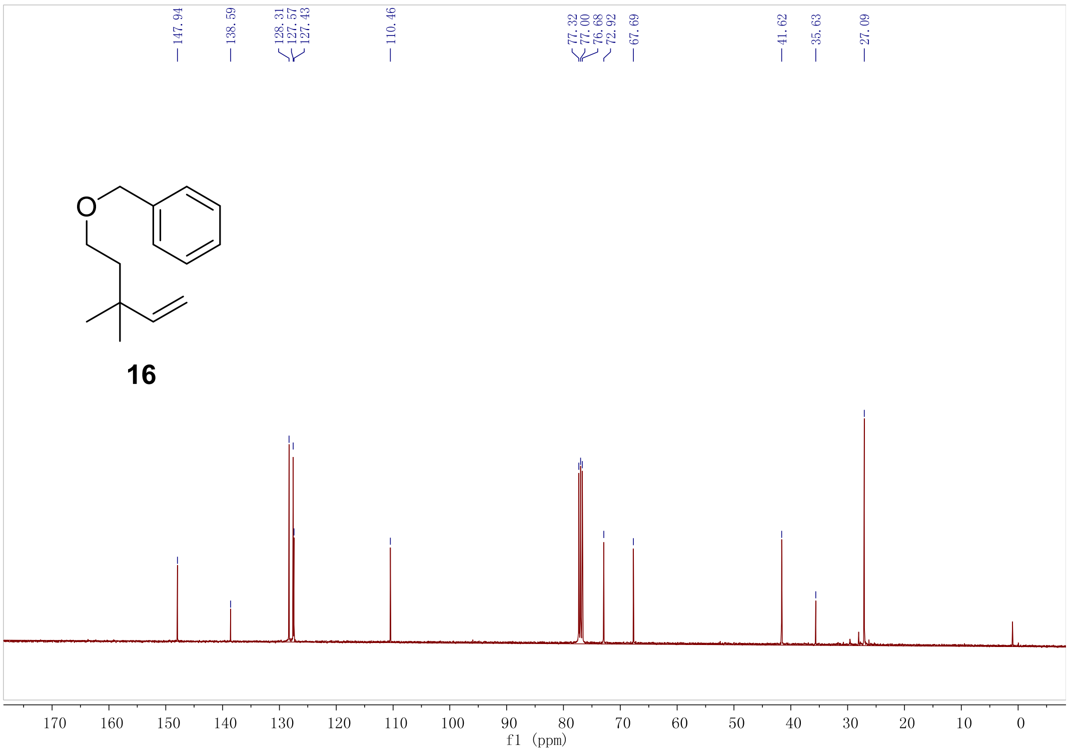

Supplementary Figure 80. ^{13}C NMR (400 MHz, $CDCl_3$) spectrum of compound **16**

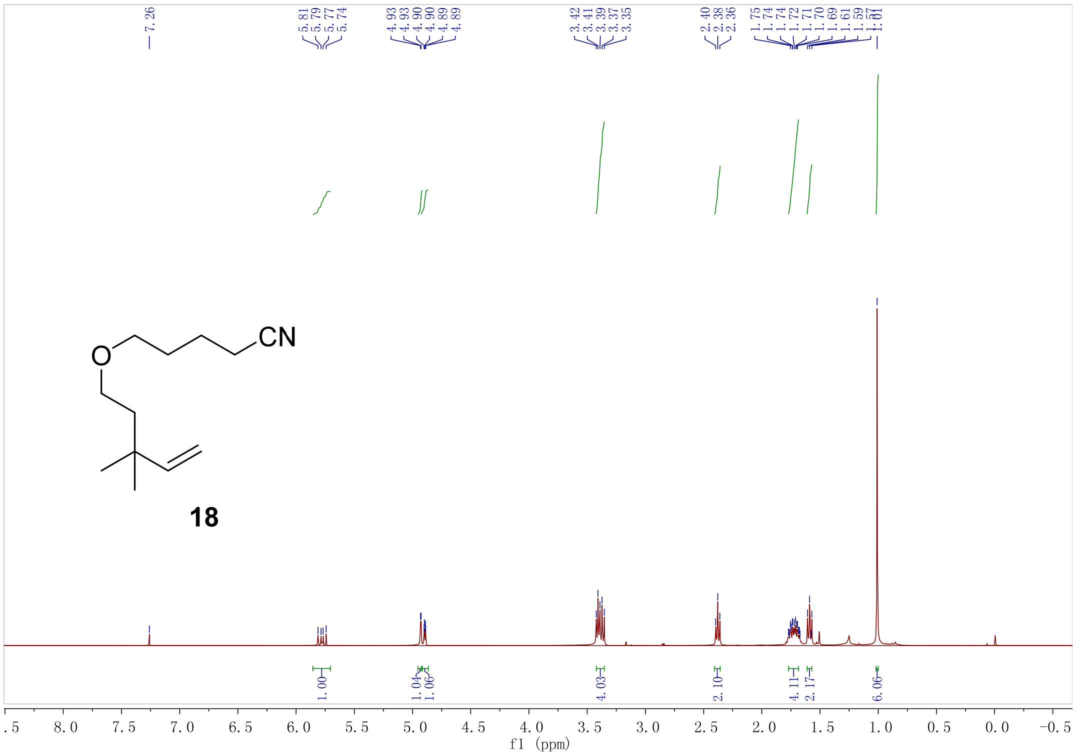

Supplementary Figure 81. ^{1}H NMR (400 MHz, $CDCl_3$) spectrum of compound **18**

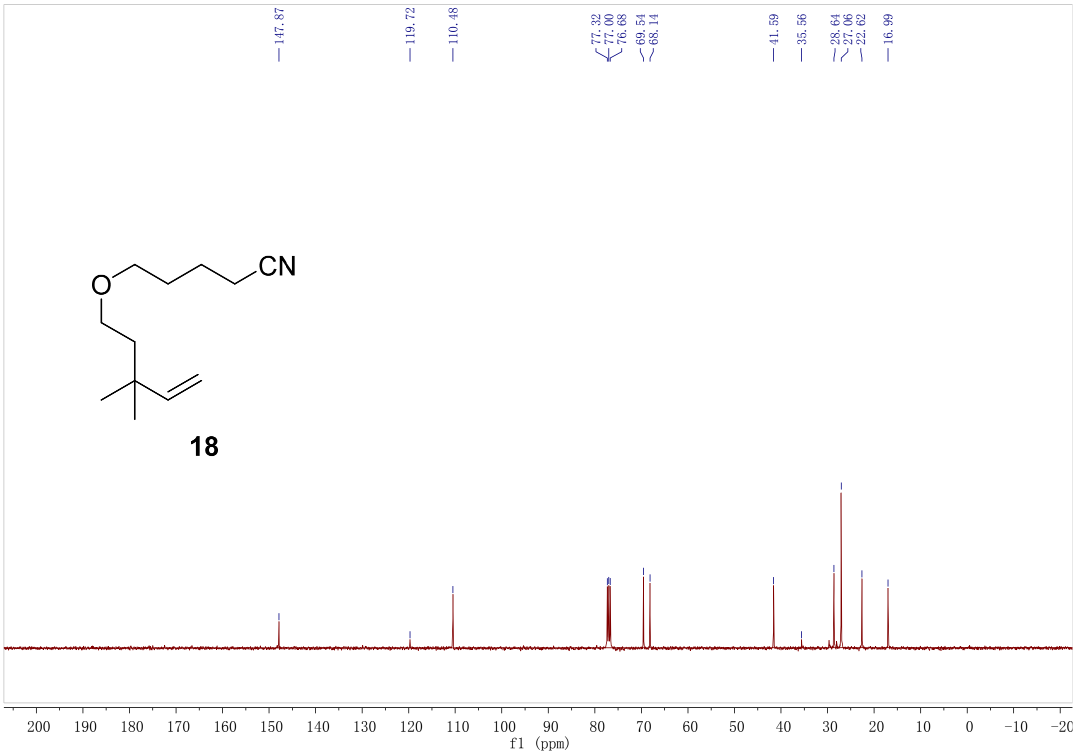

Supplementary Figure 82. ^{13}C NMR (400 MHz, $CDCl_3$) spectrum of compound **18**

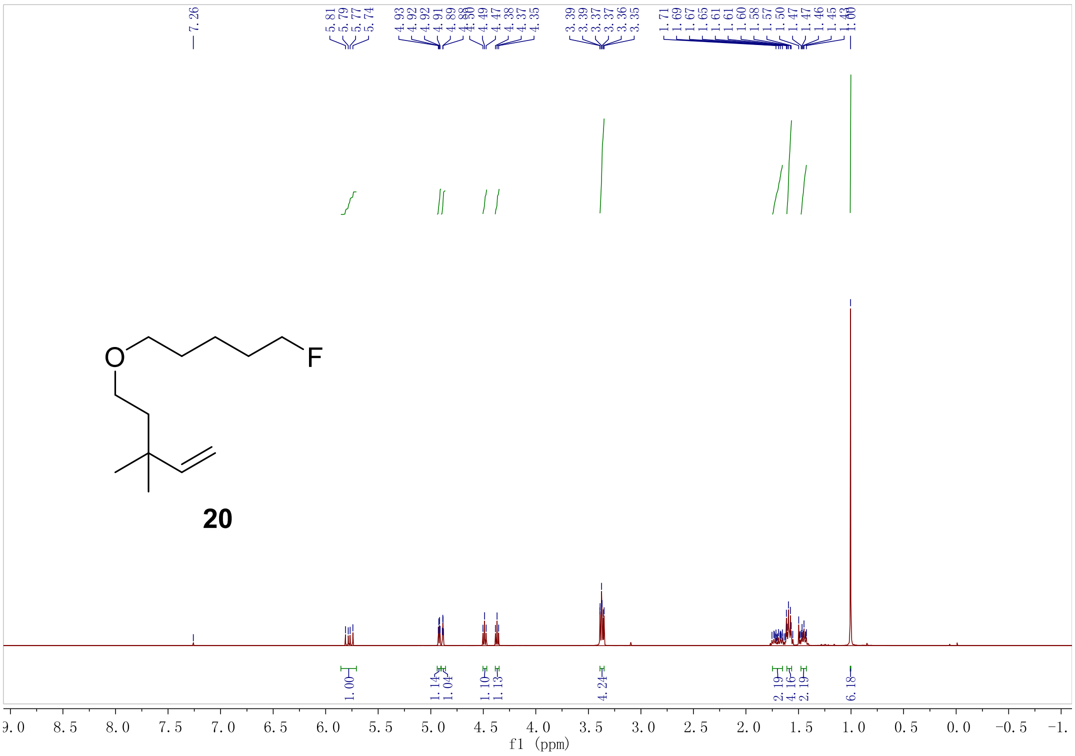

Supplementary Figure 83. ^{1}H NMR (400 MHz, $CDCl_3$) spectrum of compound **20**

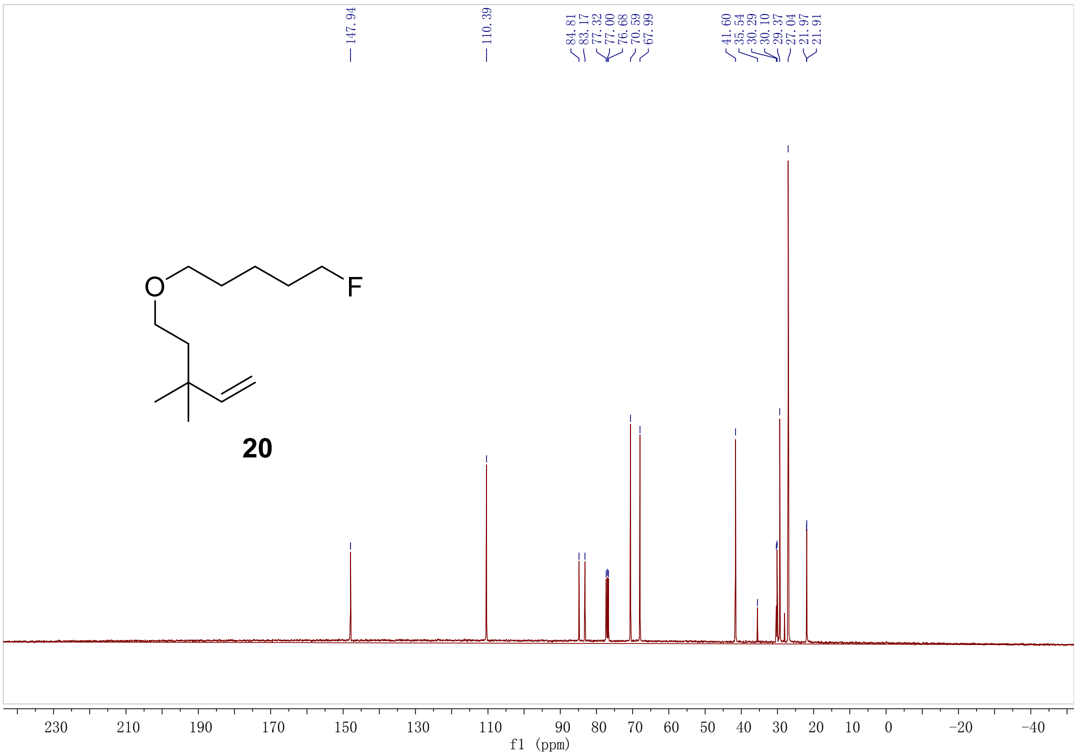

Supplementary Figure 84. ^{13}C NMR (400 MHz, $CDCl_3$) spectrum of compound **20**

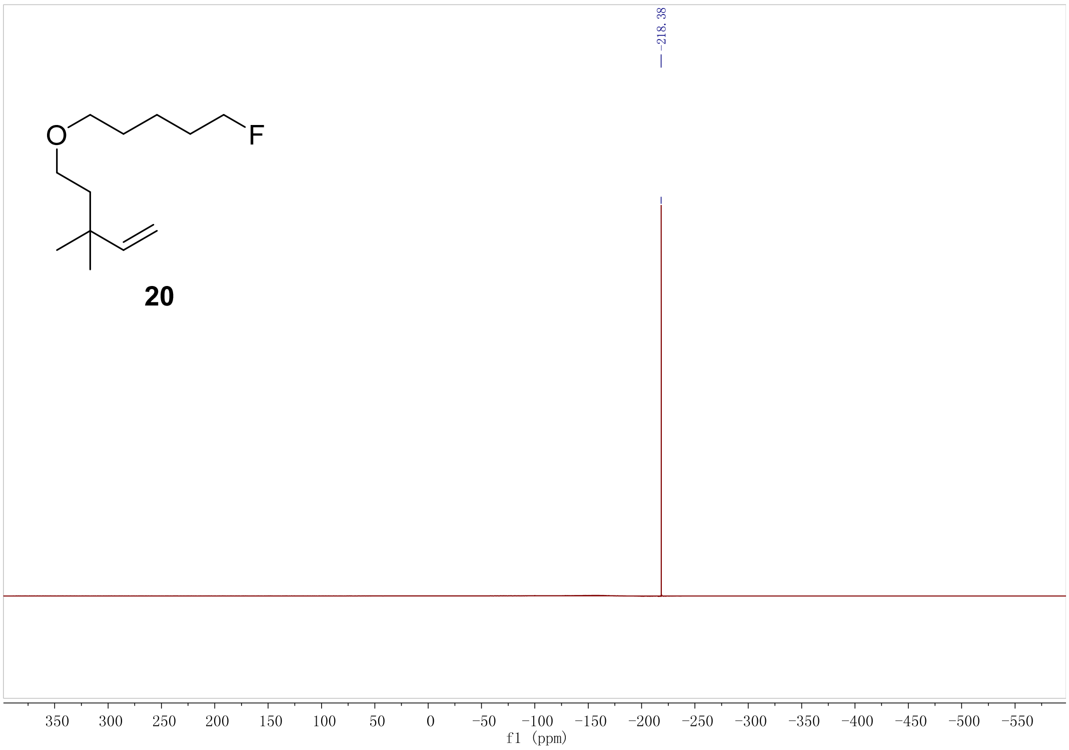

Supplementary Figure 85. ^{19}F NMR (377 MHz, $CDCl_3$) spectrum of compound **20**

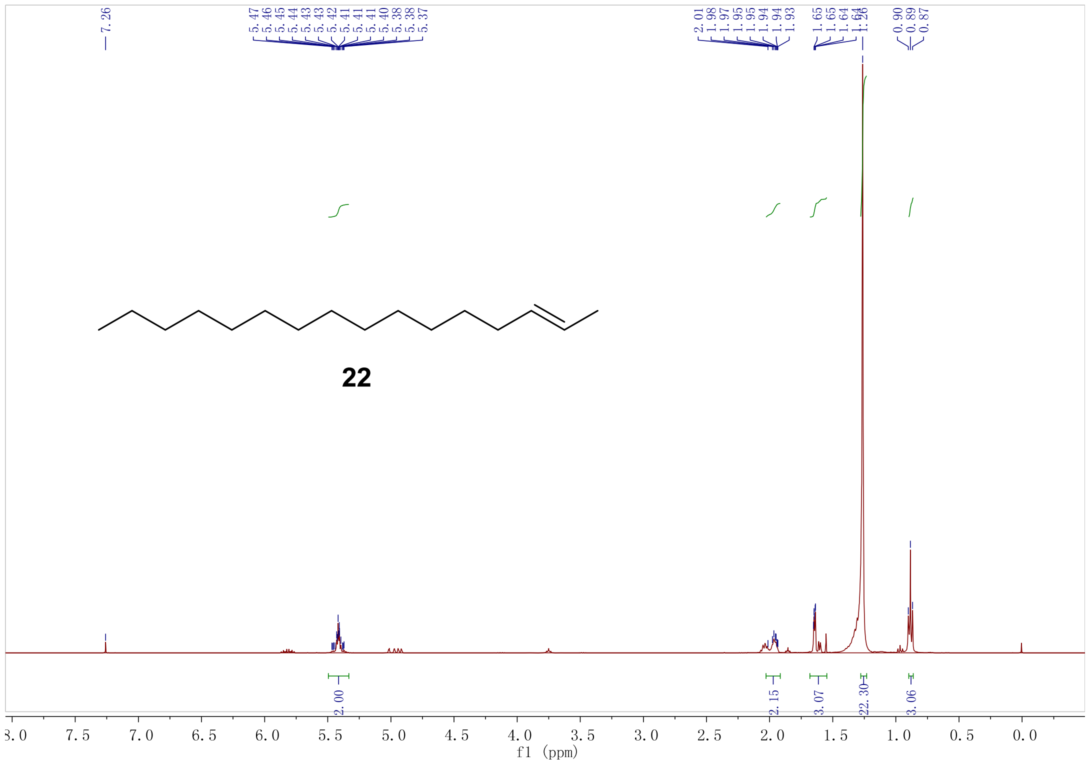

Supplementary Figure 86. ^{1}H NMR (400 MHz, $CDCl_3$) spectrum of compound **22**

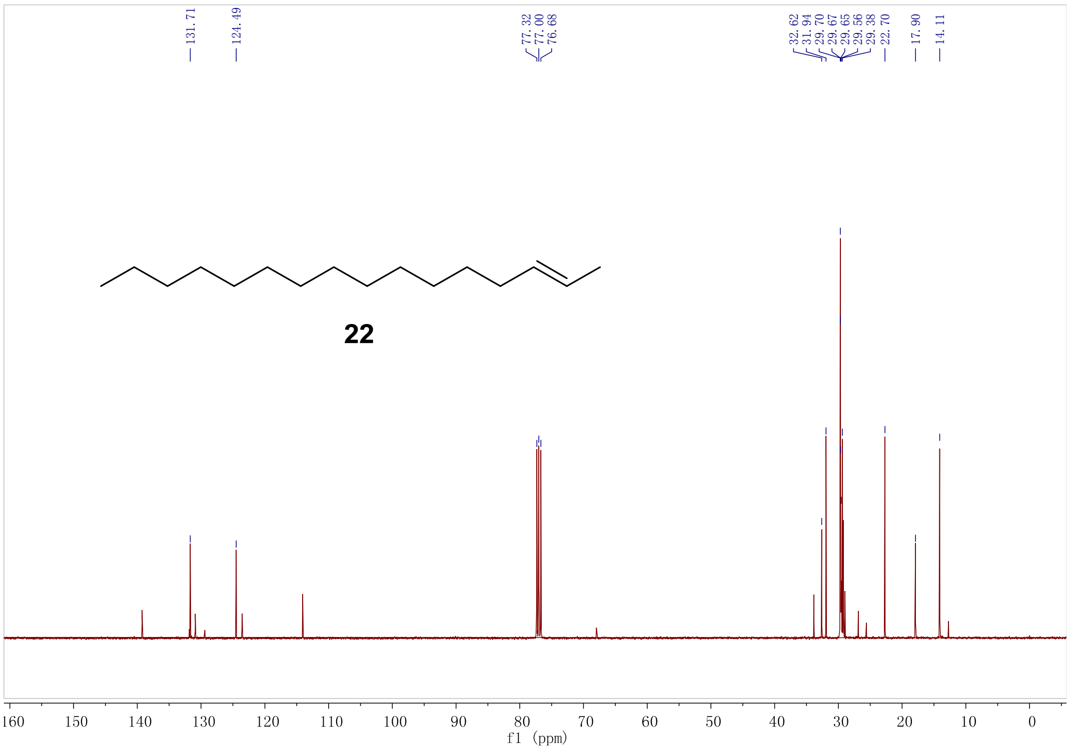

Supplementary Figure 87. ^{13}C NMR (400 MHz, $CDCl_3$) spectrum of compound **22**

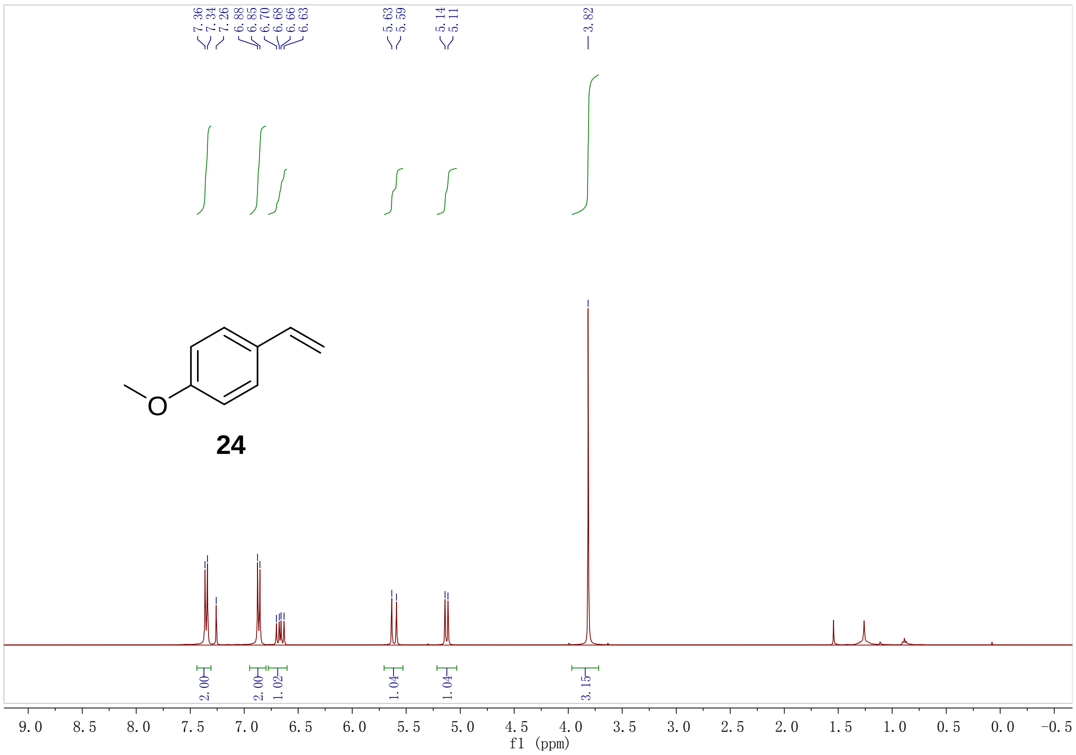

Supplementary Figure 88. ^{1}H NMR (400 MHz, $CDCl_3$) spectrum of compound **24**

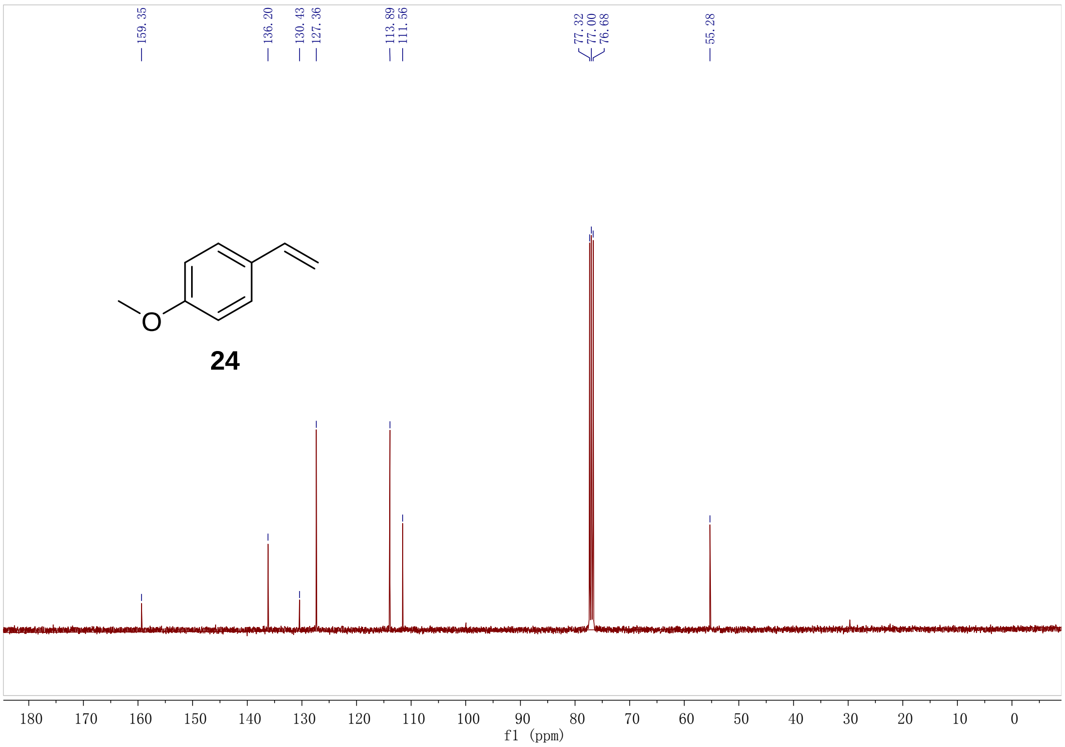

Supplementary Figure 89. ^{13}C NMR (400 MHz, $CDCl_3$) spectrum of compound **24**

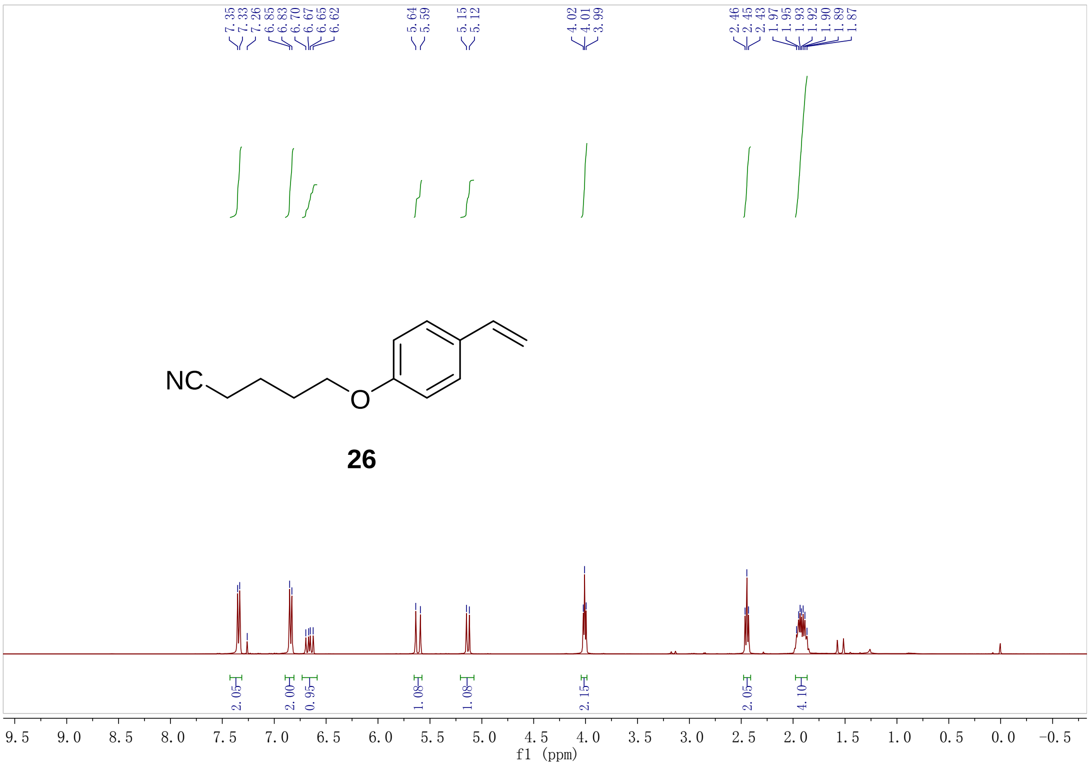

Supplementary Figure 90. ^{1}H NMR (400 MHz, $CDCl_3$) spectrum of compound **26**

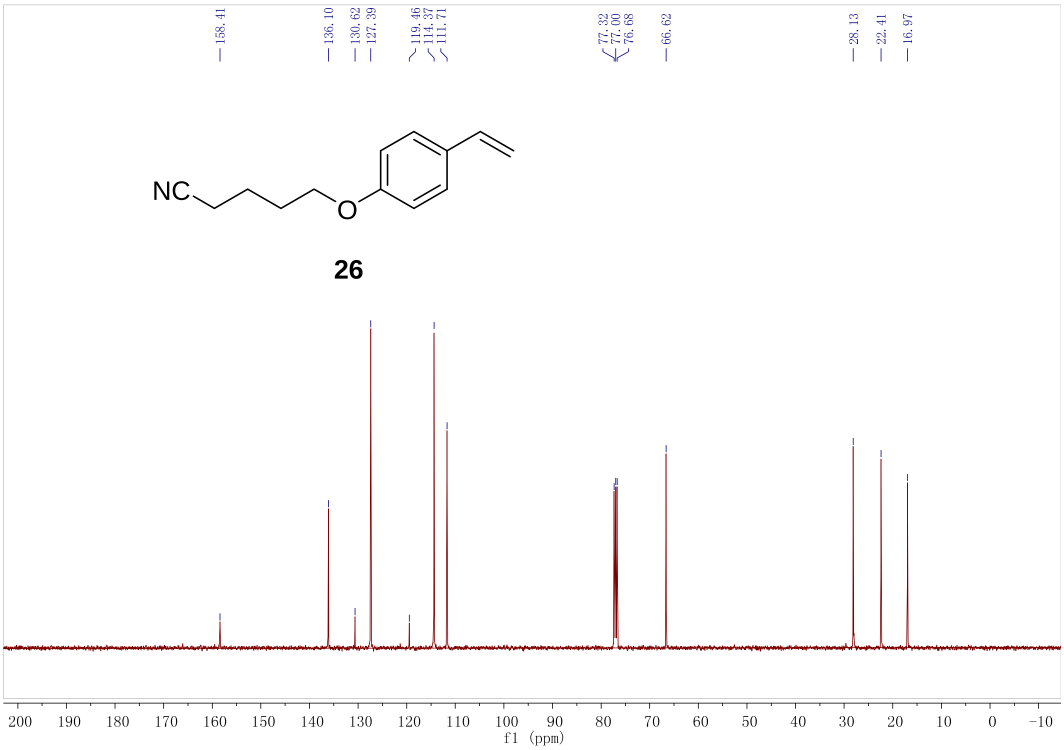

Supplementary Figure 91. ^{13}C NMR (400 MHz, $CDCl_3$) spectrum of compound **26**

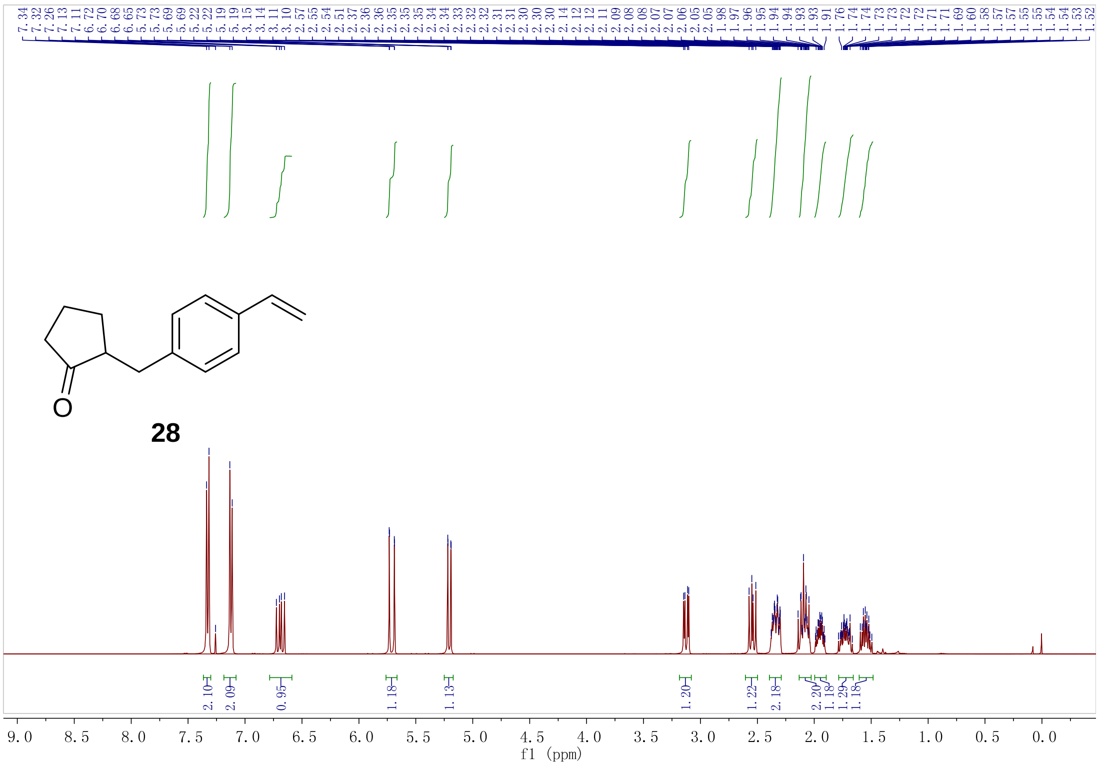

Supplementary Figure 92. ^{1}H NMR (400 MHz, $CDCl_3$) spectrum of compound **28**

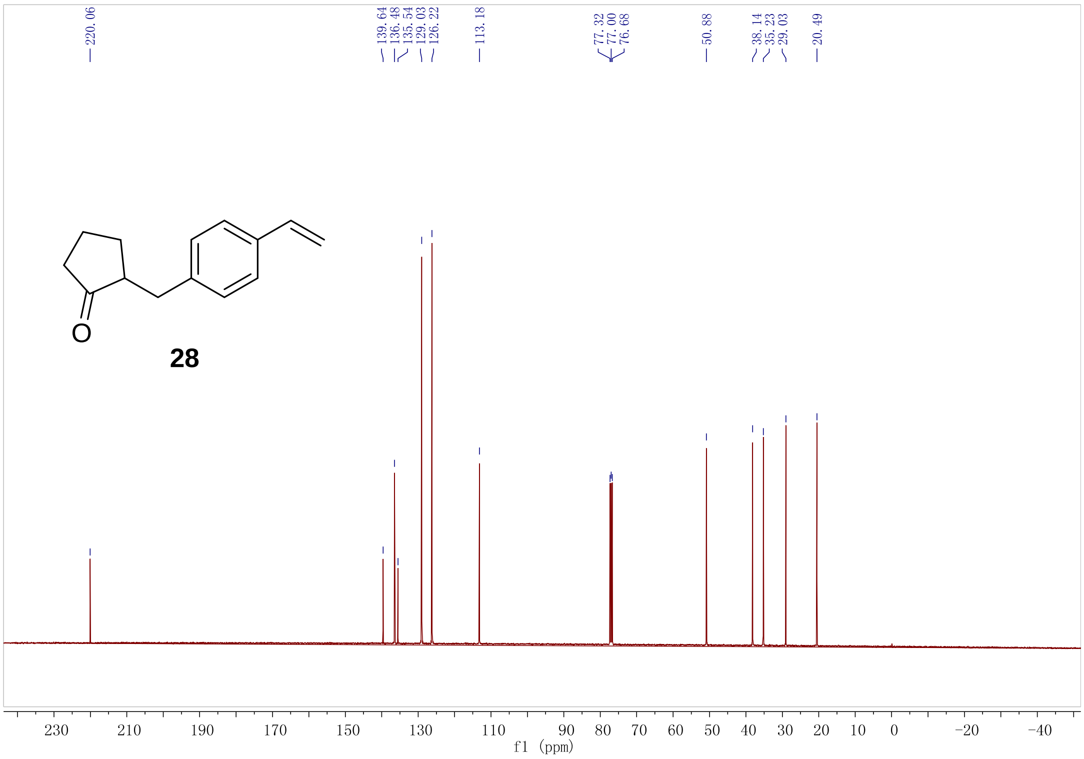

Supplementary Figure 93. ^{13}C NMR (400 MHz, $CDCl_3$) spectrum of compound **28**

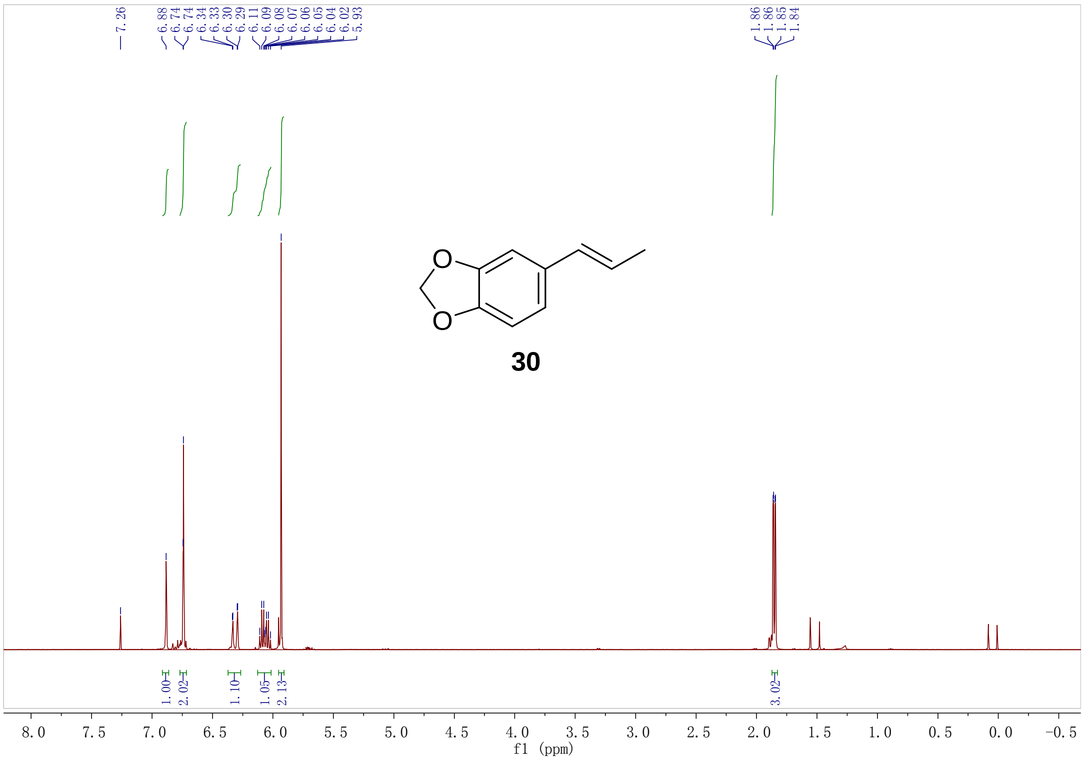

Supplementary Figure 94. ^{1}H NMR (400 MHz, $CDCl_3$) spectrum of compound **30**

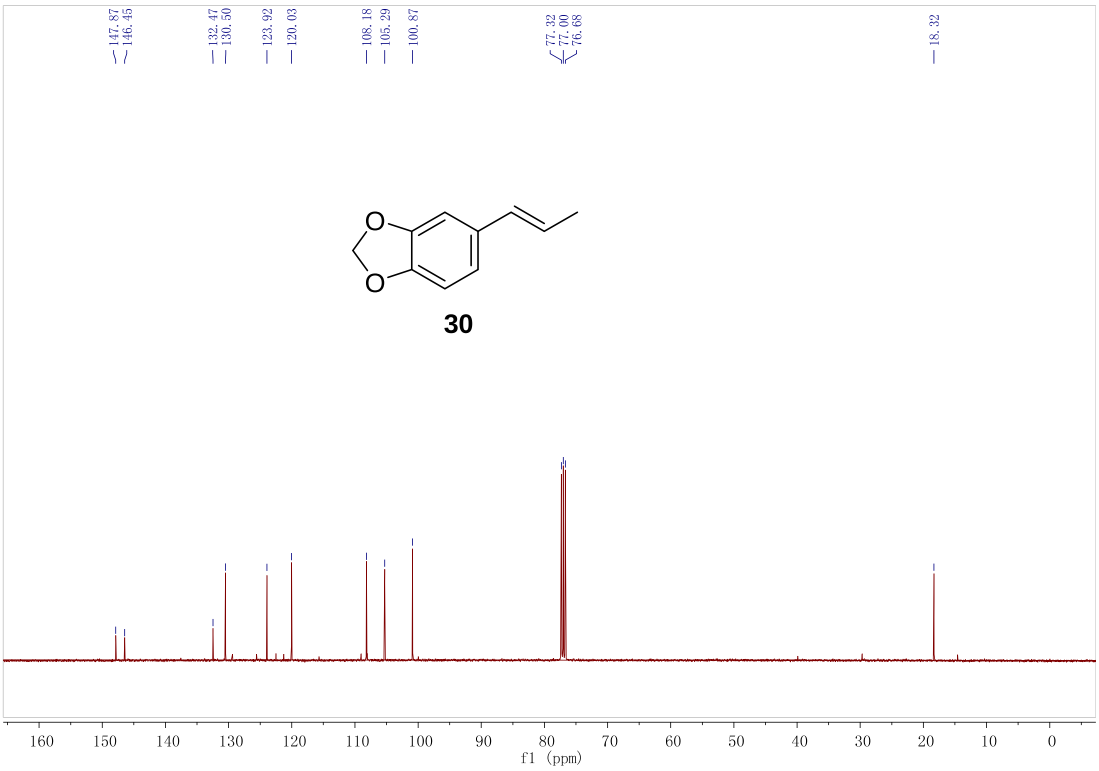

Supplementary Figure 95. ^{13}C NMR (400 MHz, $CDCl_3$) spectrum of compound **30**

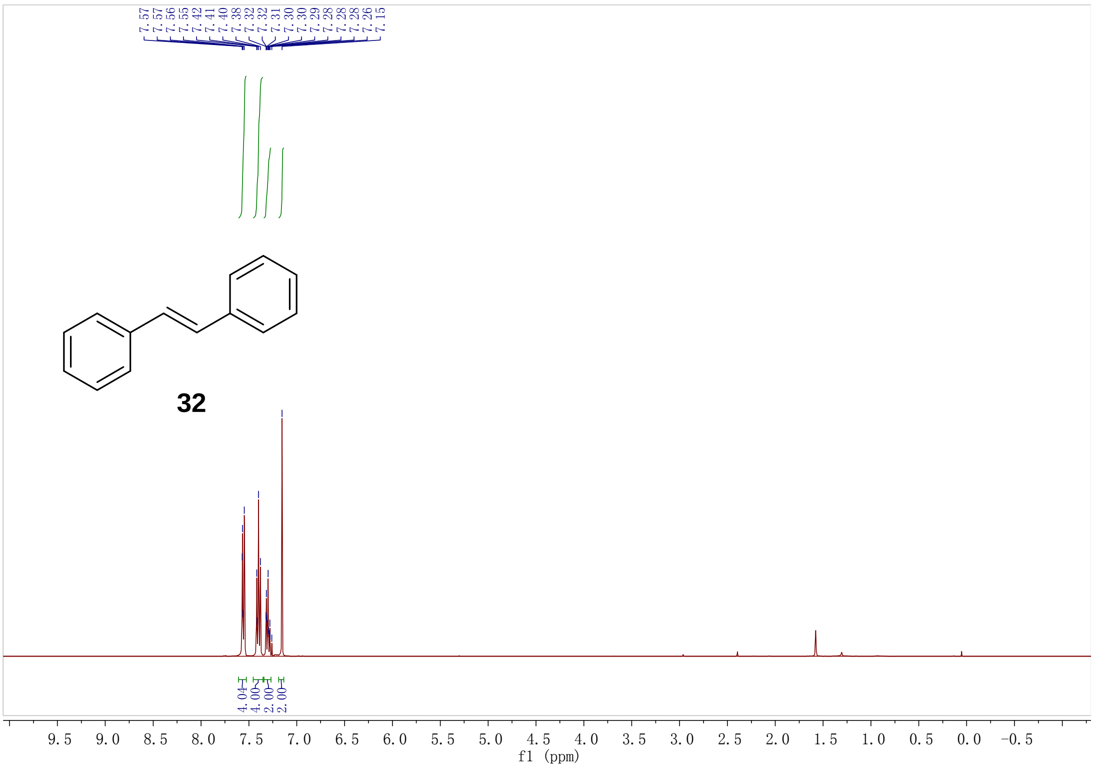

Supplementary Figure 96. ^{1}H NMR (400 MHz, $CDCl_3$) spectrum of compound **32**

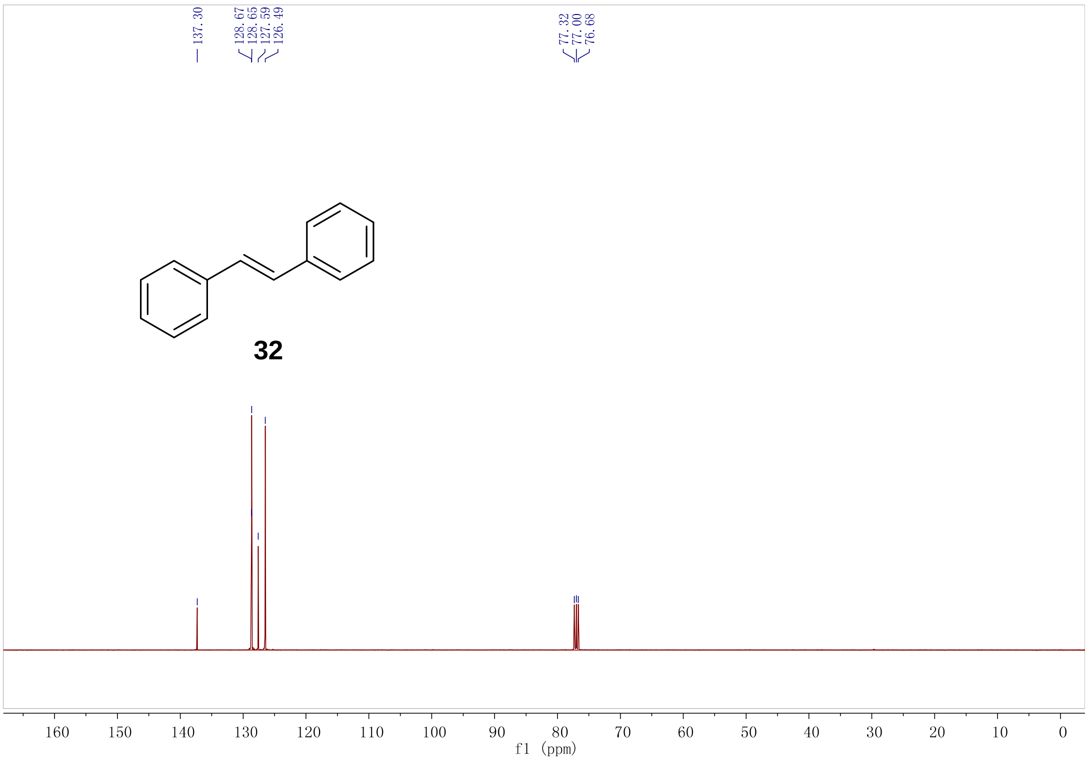

Supplementary Figure 97. ^{13}C NMR (400 MHz, $CDCl_3$) spectrum of compound **32**

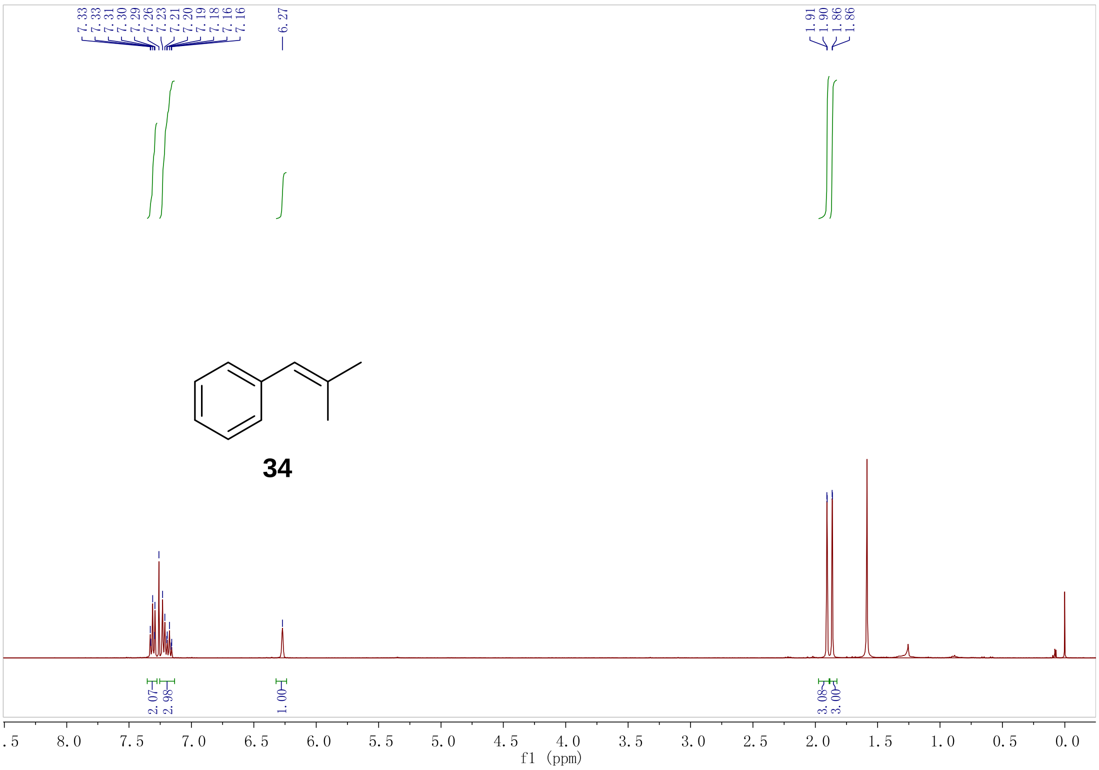

Supplementary Figure 98. ¹H NMR (400 MHz, $CDCl_3$) spectrum of compound **34**

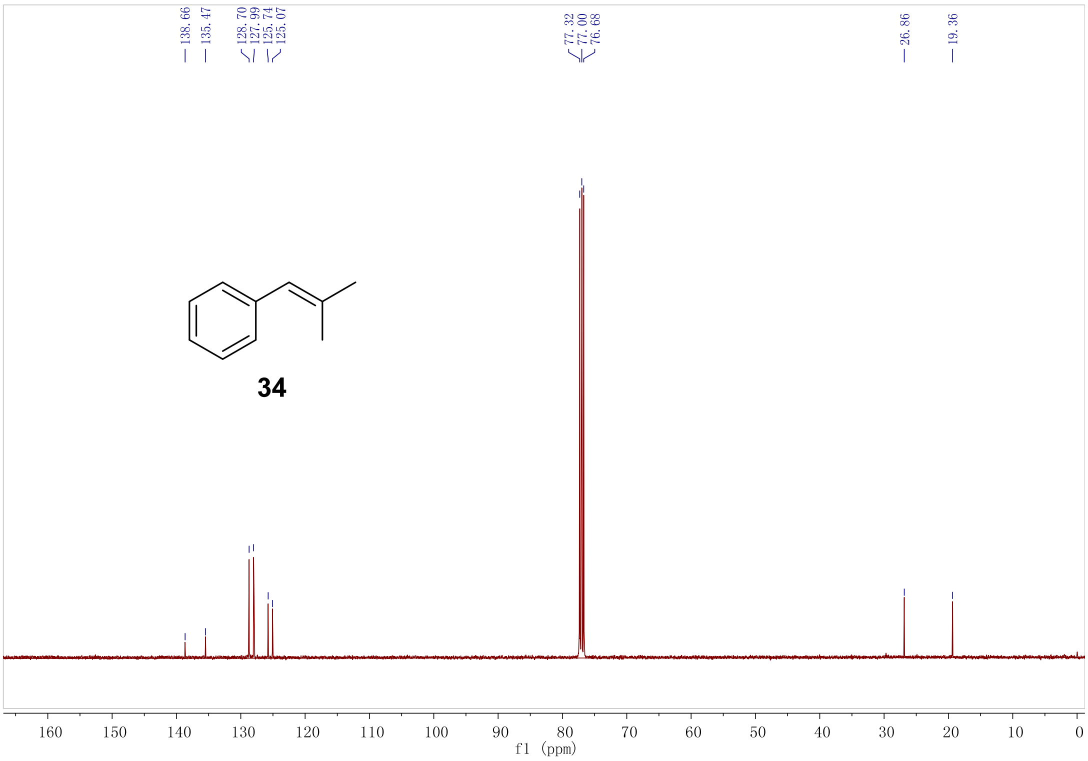

Supplementary Figure 99. ¹³C NMR (400 MHz, $CDCl_3$) spectrum of compound **34**

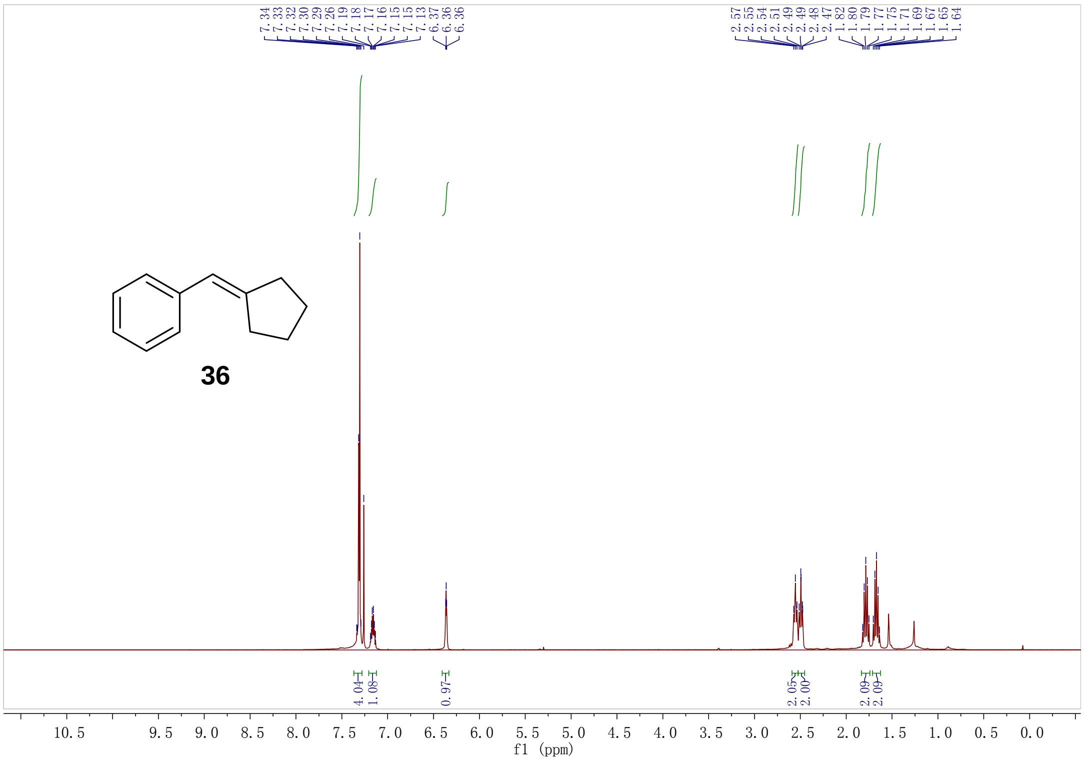

Supplementary Figure 100. ^{1}H NMR (400 MHz, $CDCl_3$) spectrum of compound **36**

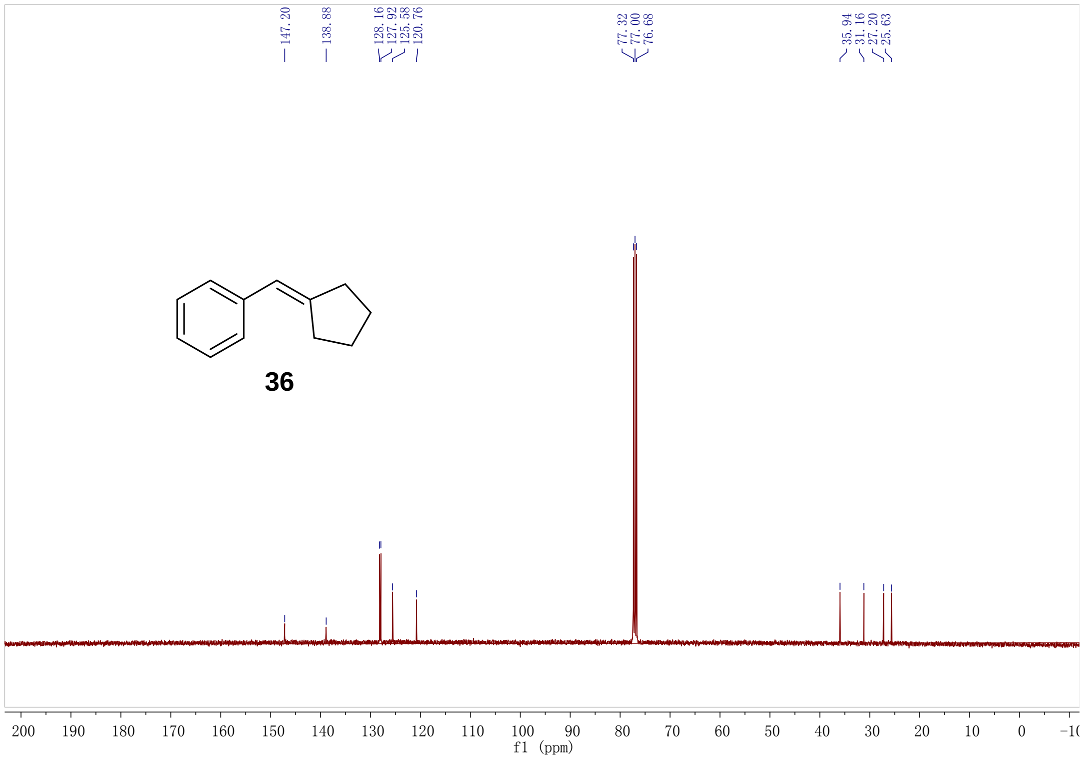

Supplementary Figure 101. ^{13}C NMR (400 MHz, $CDCl_3$) spectrum of compound **36**

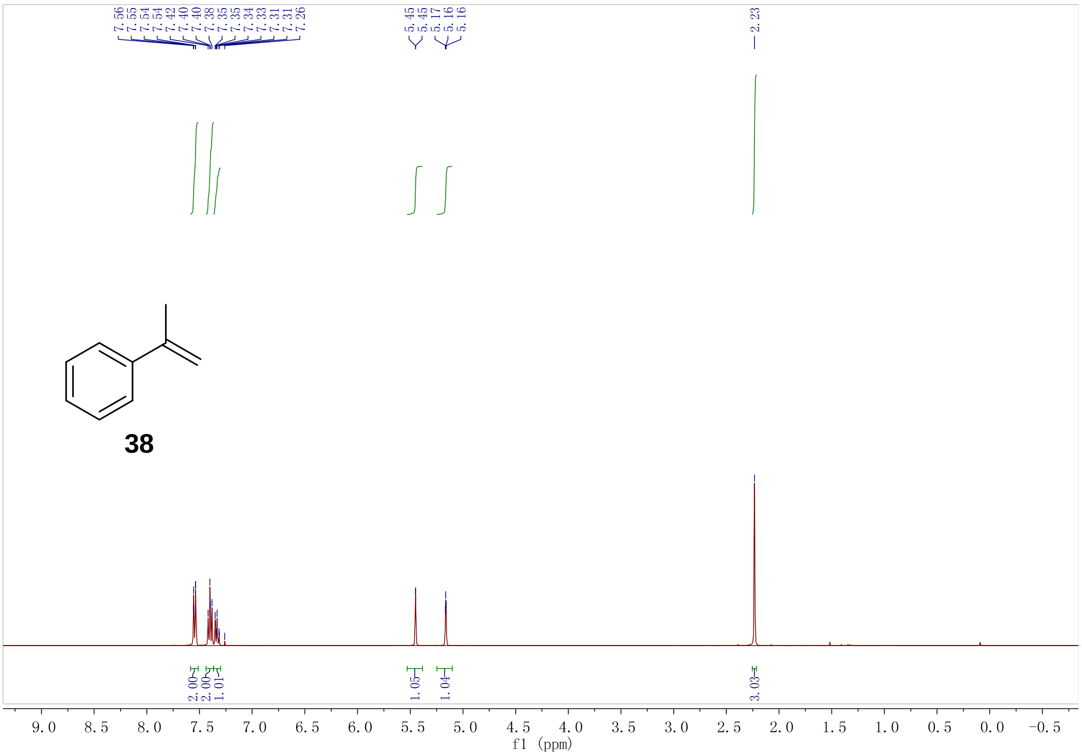

Supplementary Figure 102. ^{1}H NMR (400 MHz, $CDCl_3$) spectrum of compound **38**

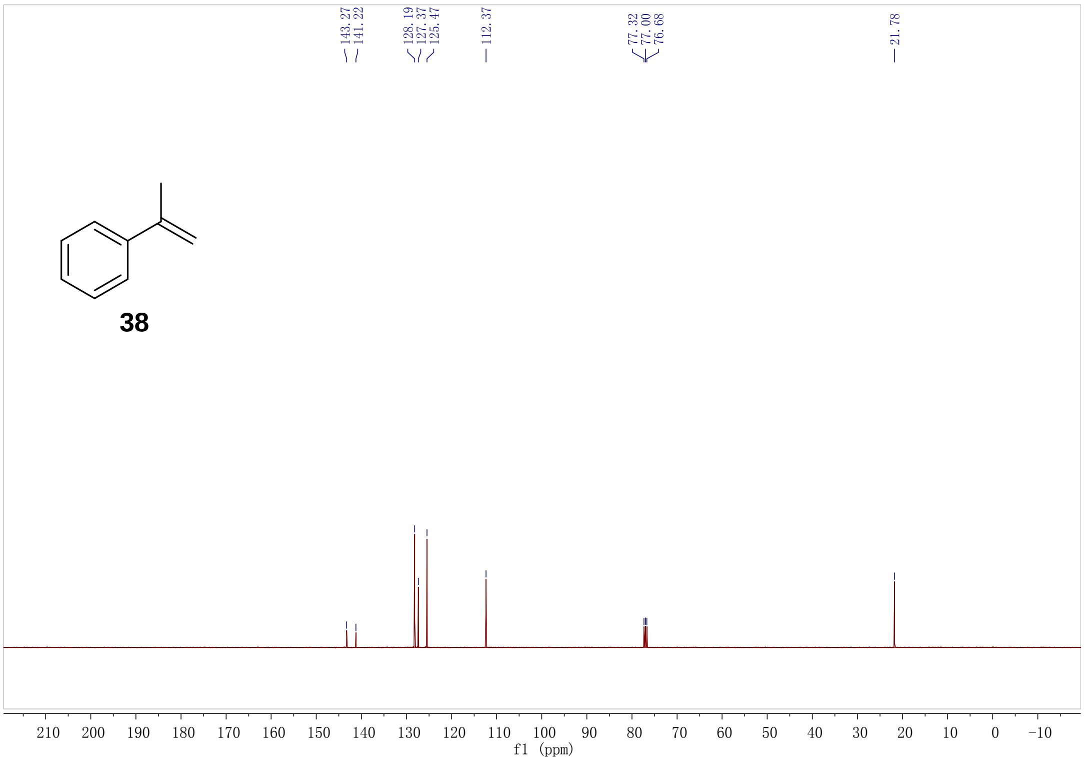

Supplementary Figure 103. ^{13}C NMR (400 MHz, $CDCl_3$) spectrum of compound **38**

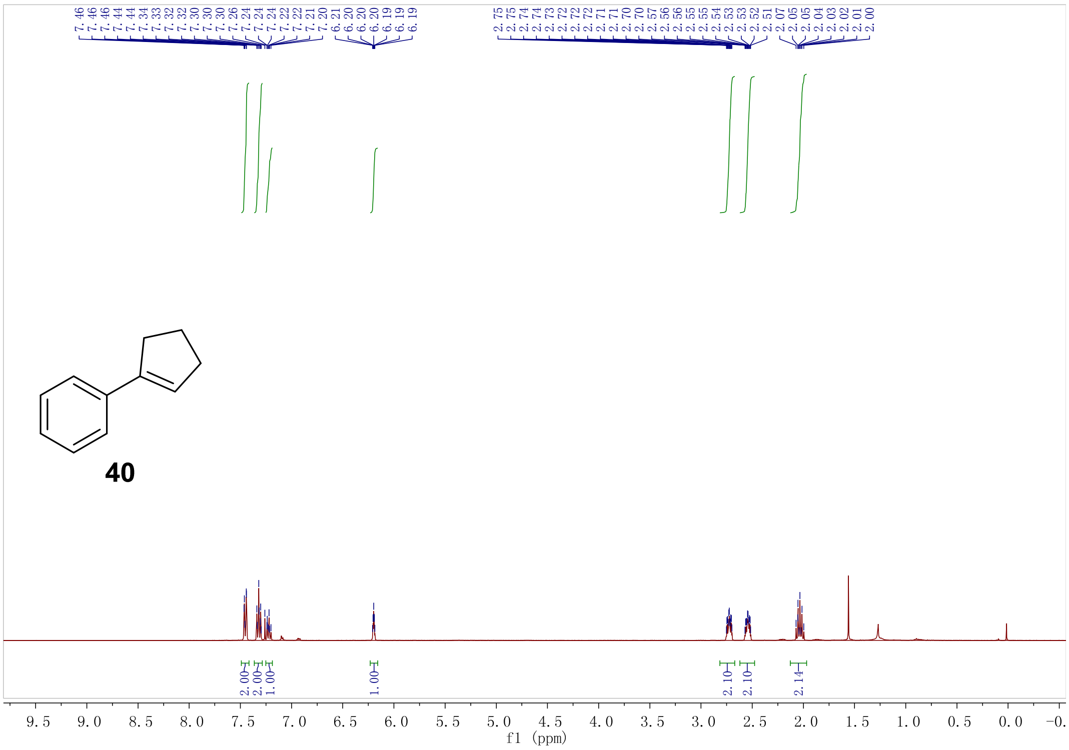

Supplementary Figure 104. ^{1}H NMR (400 MHz, $CDCl_3$) spectrum of compound **40**

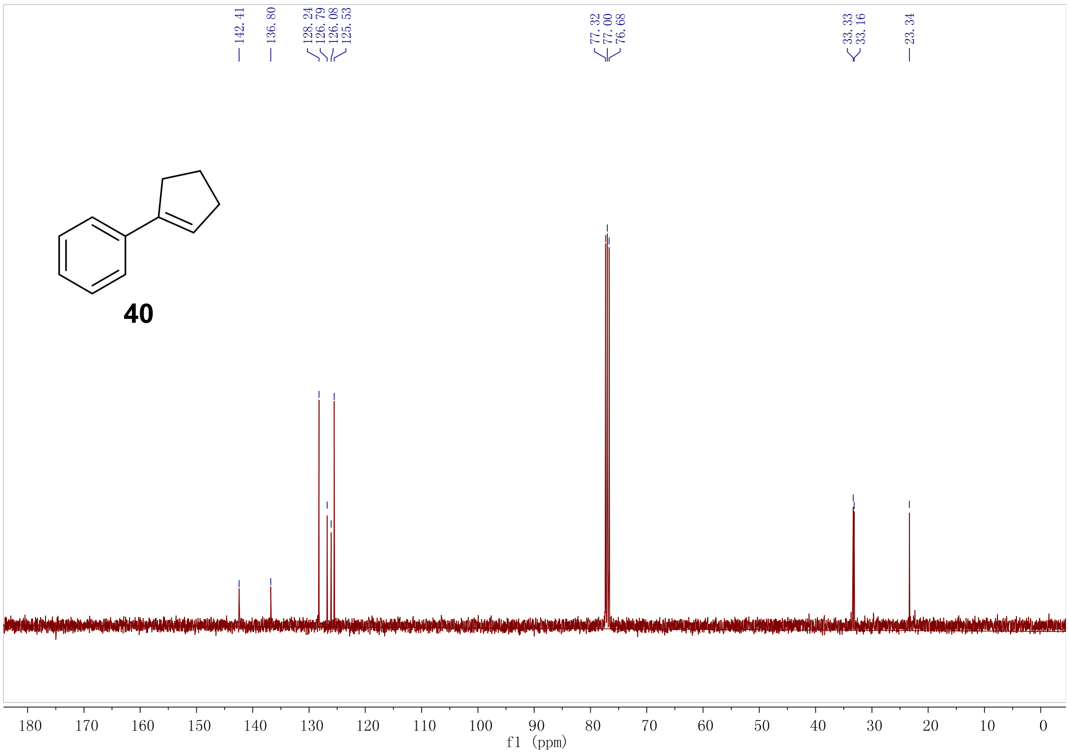

Supplementary Figure 105. ^{13}C NMR (400 MHz, $CDCl_3$) spectrum of compound **40**

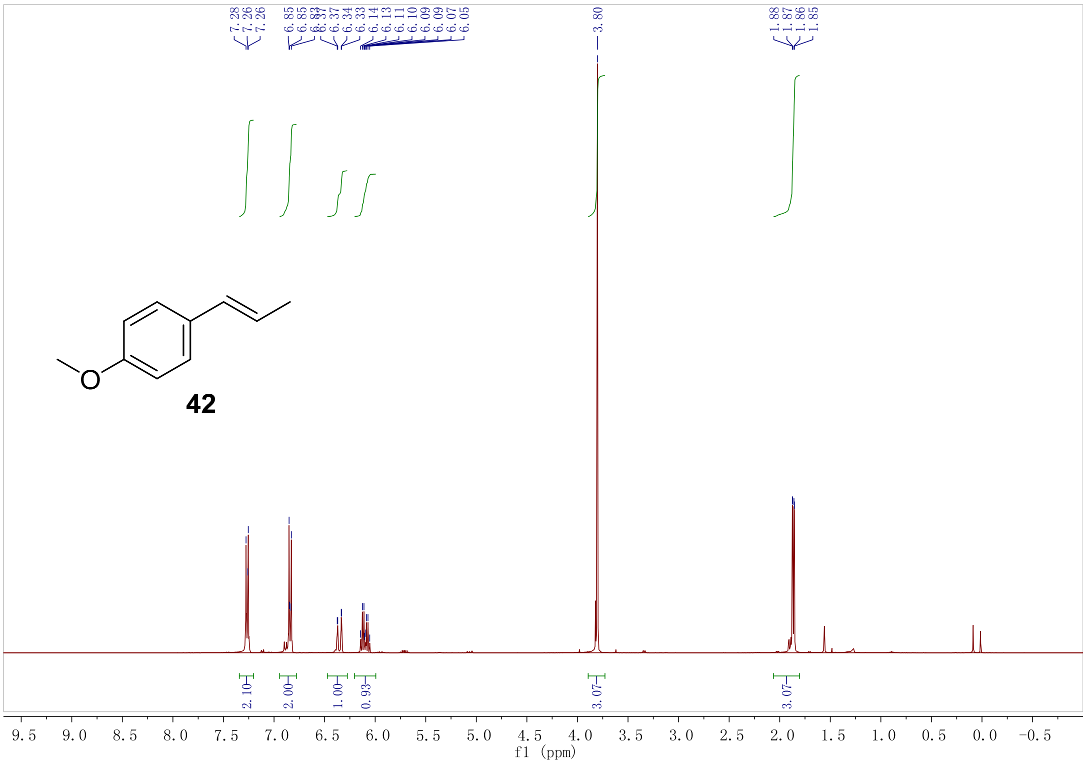

Supplementary Figure 106. ^{1}H NMR (400 MHz, $CDCl_3$) spectrum of compound **42**

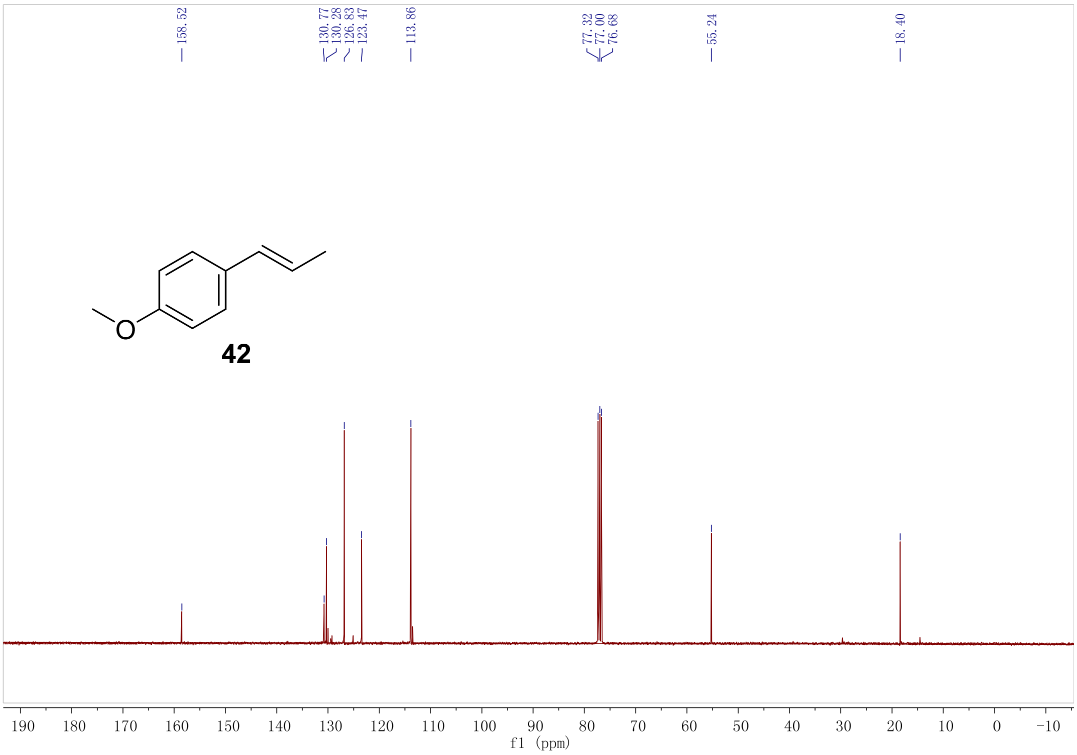

Supplementary Figure 107. ^{13}C NMR (400 MHz, $CDCl_3$) spectrum of compound **42**

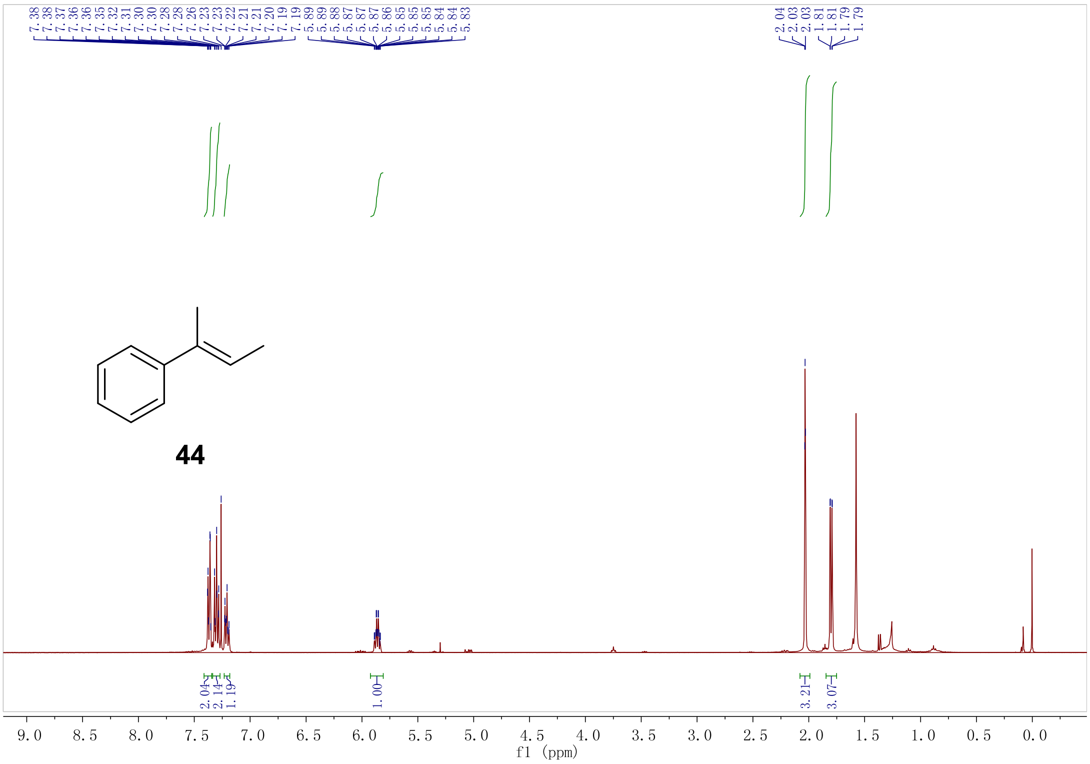

Supplementary Figure 108. ^{1}H NMR (400 MHz, $CDCl_3$) spectrum of compound **44**

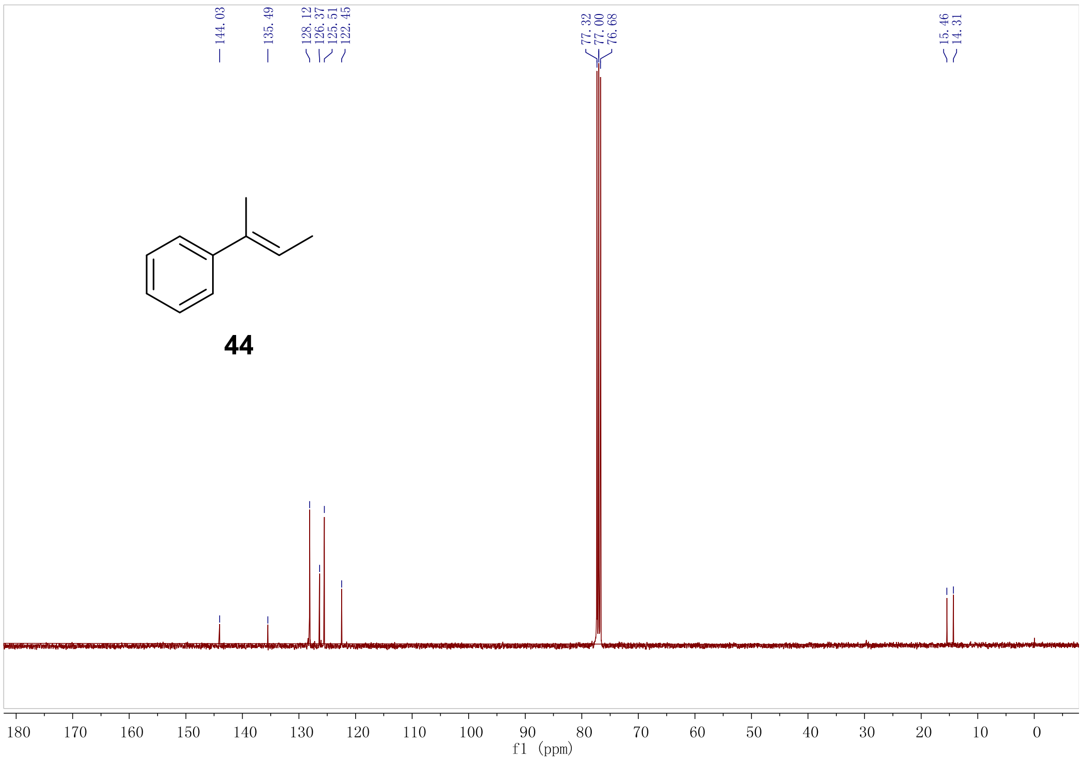

Supplementary Figure 109. ^{13}C NMR (400 MHz, $CDCl_3$) spectrum of compound **44**

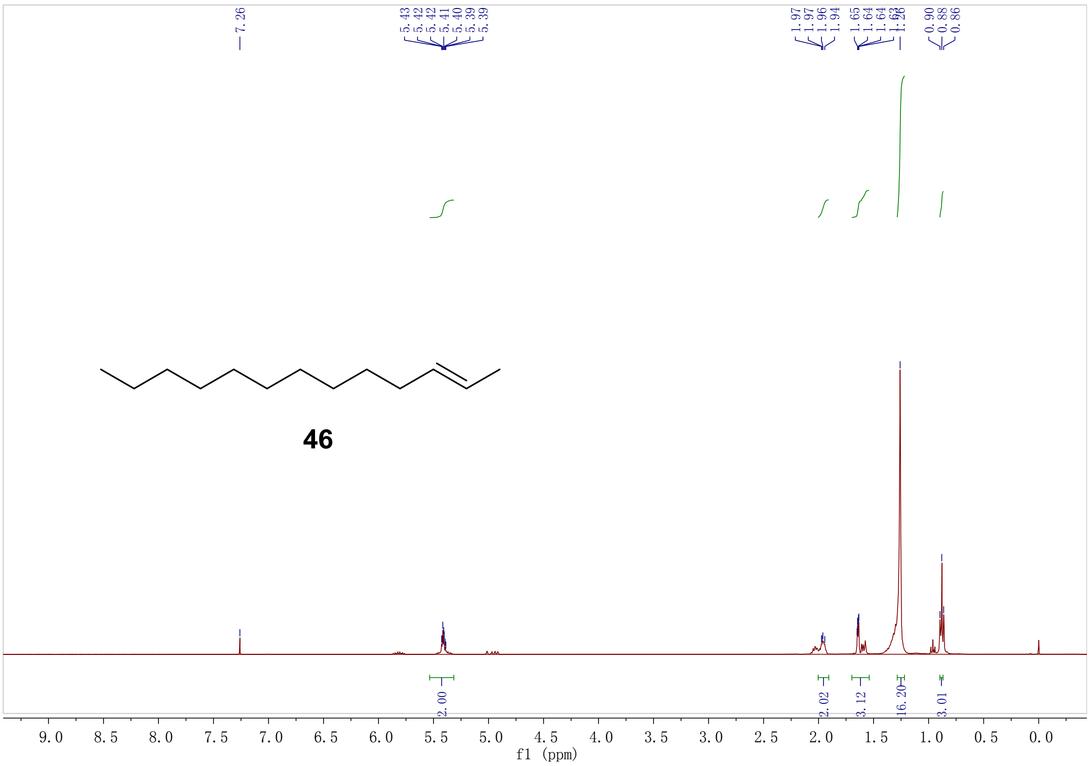

Supplementary Figure 110. ^{1}H NMR (400 MHz, $CDCl_3$) spectrum of compound **46**

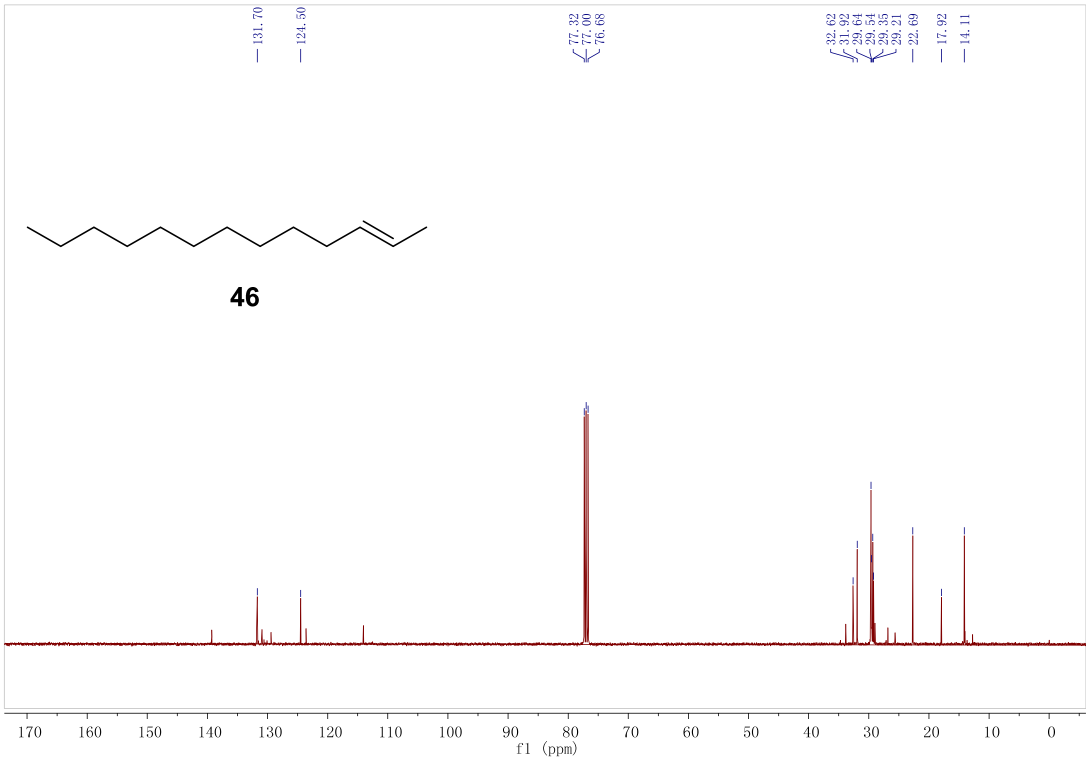

Supplementary Figure 111. ^{13}C NMR (400 MHz, $CDCl_3$) spectrum of compound **46**

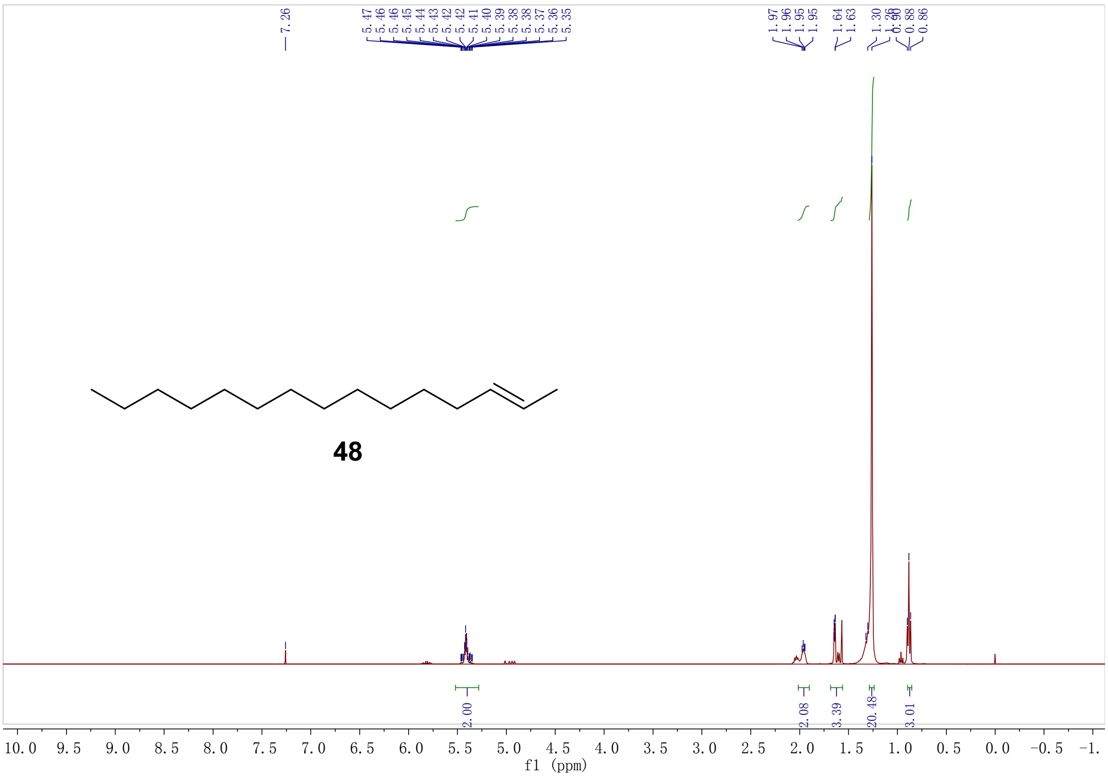

Supplementary Figure 112. ^{1}H NMR (400 MHz, $CDCl_3$) spectrum of compound **48**

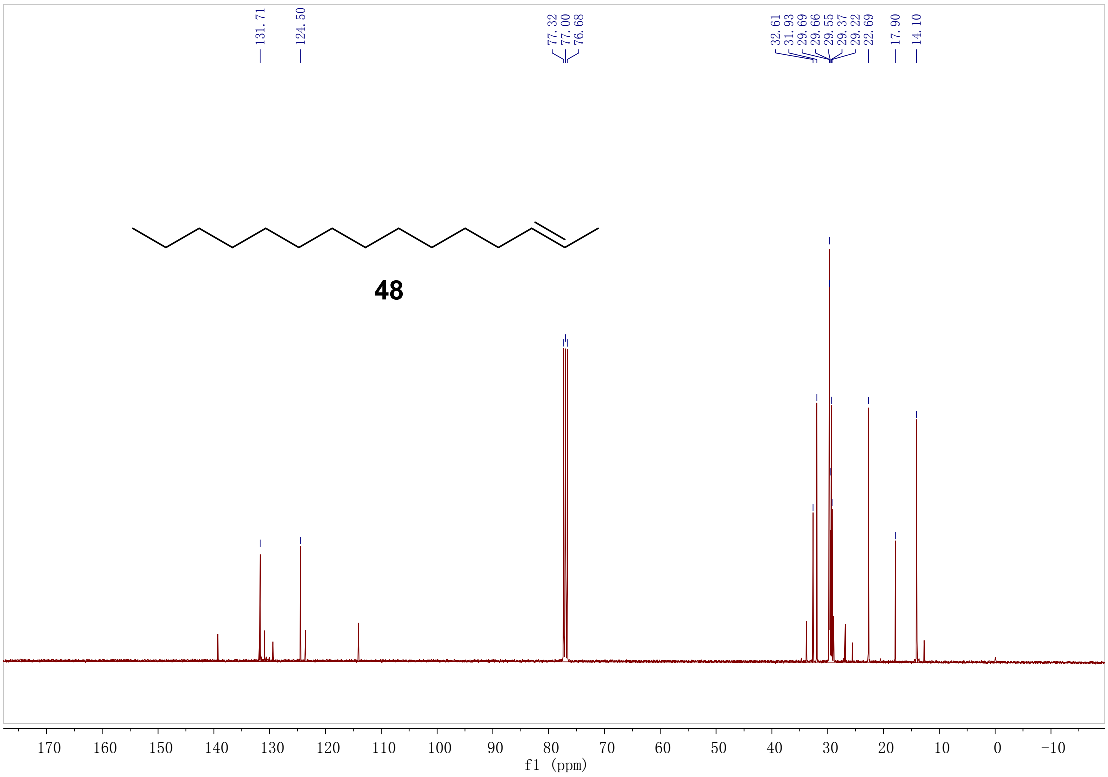

Supplementary Figure 113. ^{13}C NMR (400 MHz, $CDCl_3$) spectrum of compound **48**

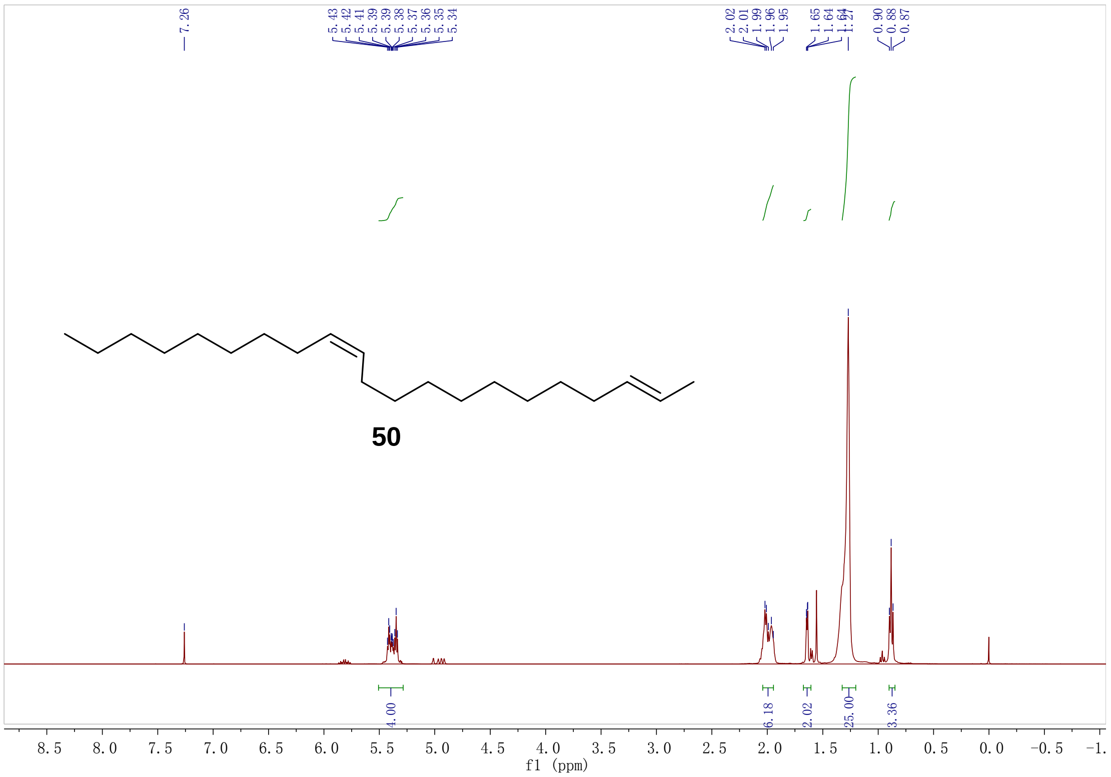

Supplementary Figure 114. ^{1}H NMR (400 MHz, $CDCl_3$) spectrum of compound **50**

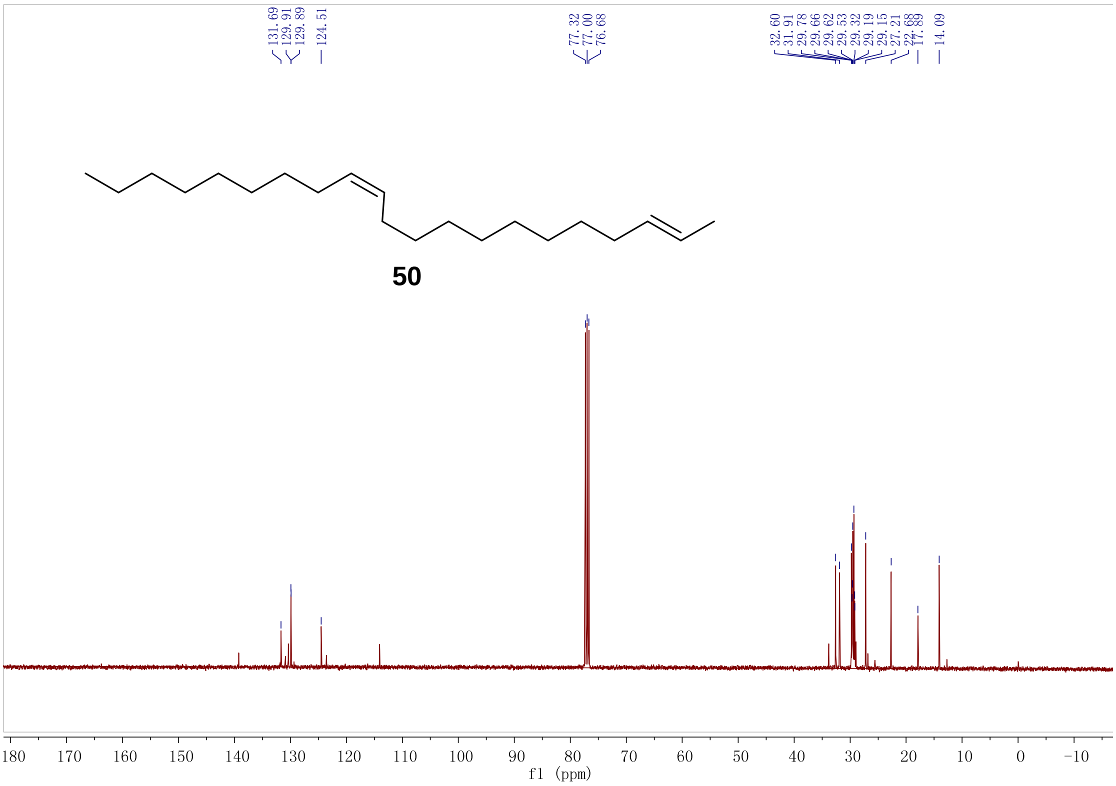

Supplementary Figure 115. ^{13}C NMR (400 MHz, $CDCl_3$) spectrum of compound **50**

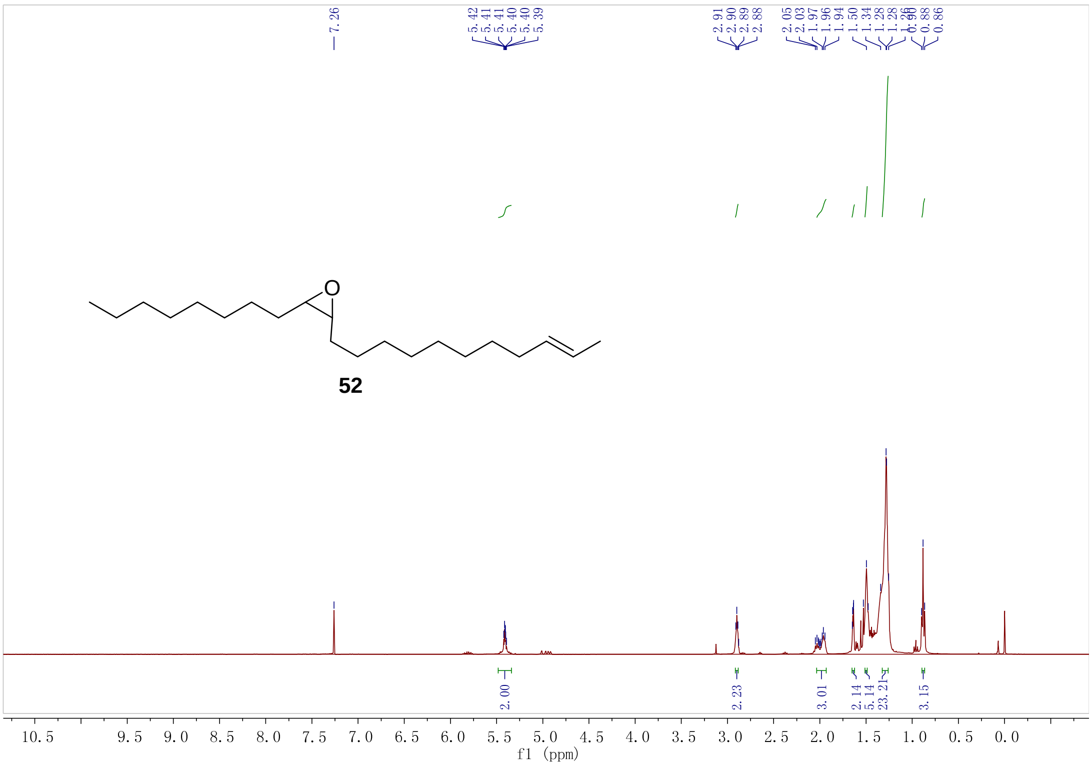

Supplementary Figure 116. ^{1}H NMR (400 MHz, $CDCl_3$) spectrum of compound **52**

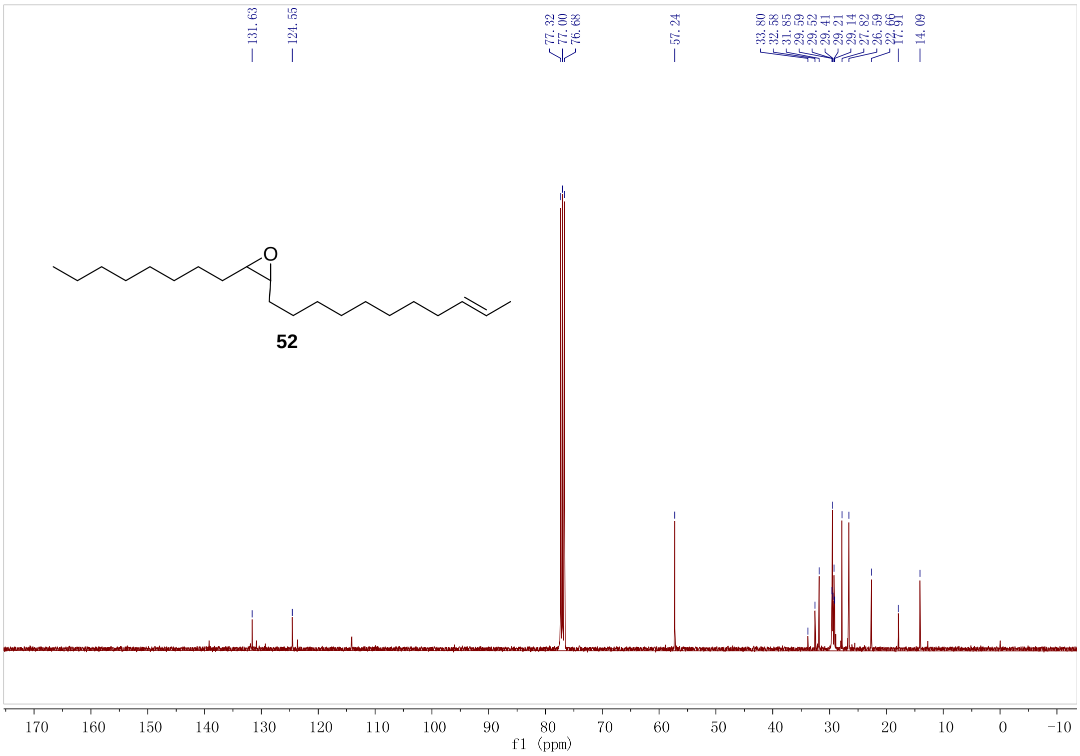

Supplementary Figure 117. ^{13}C NMR (400 MHz, $CDCl_3$) spectrum of compound **52**

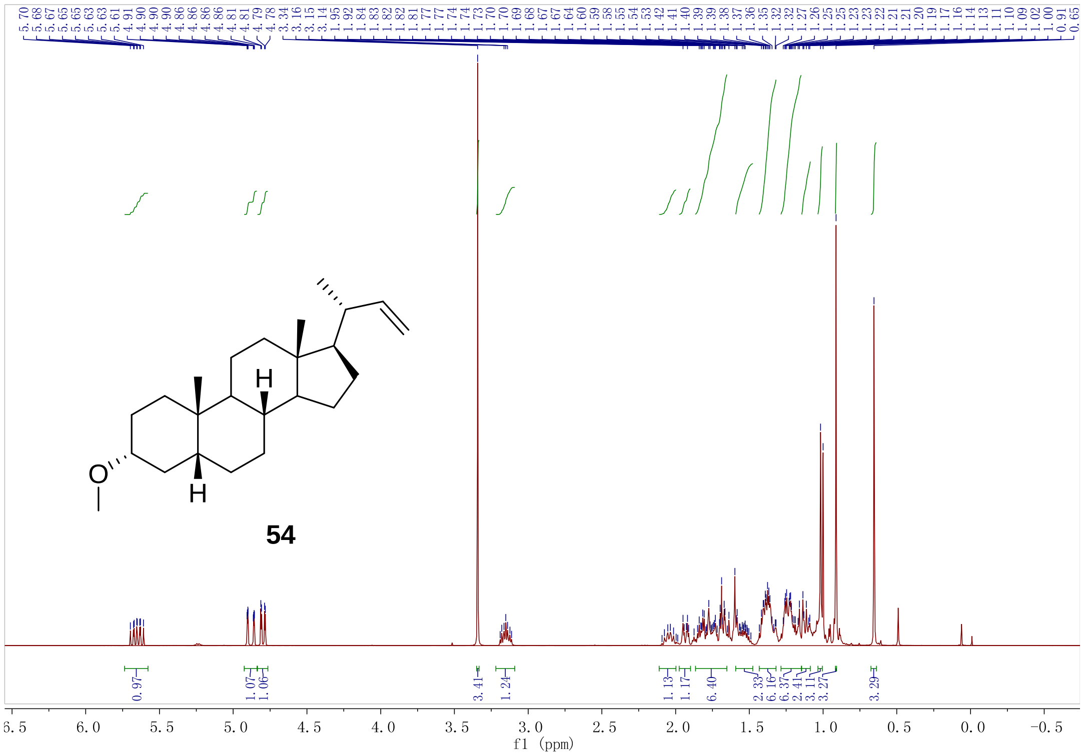

Supplementary Figure 118. ^{1}H NMR (400 MHz, $CDCl_3$) spectrum of compound **54**

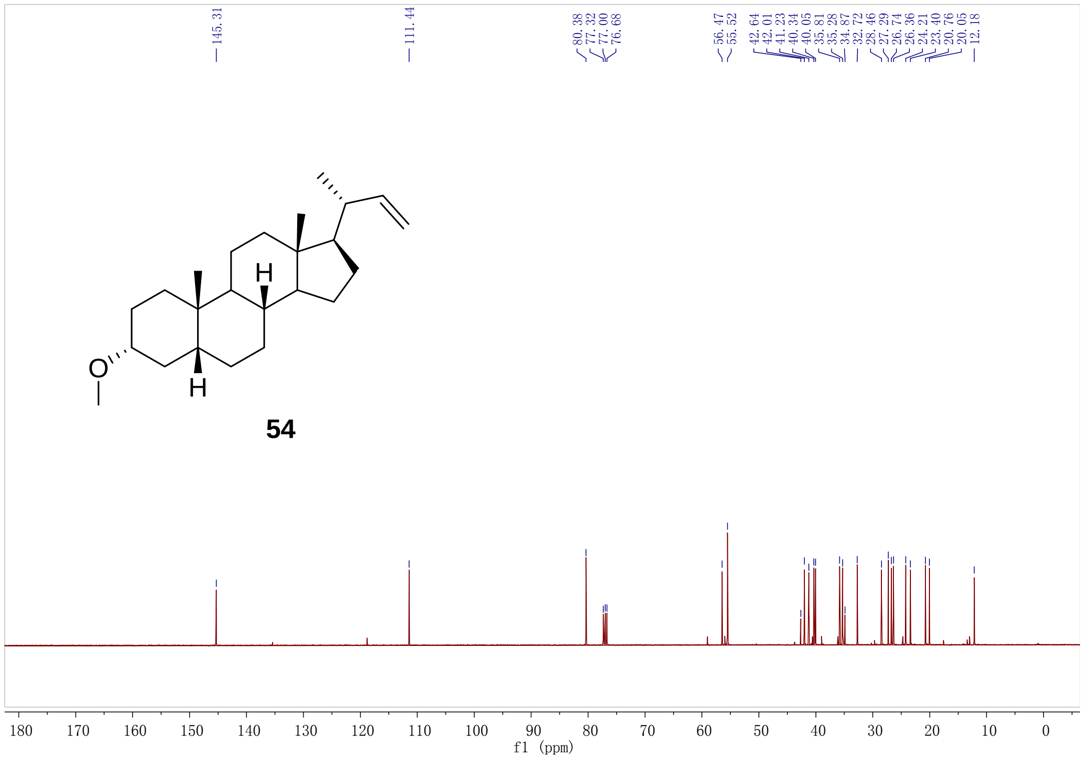

Supplementary Figure 119. ^{13}C NMR (400 MHz, $CDCl_3$) spectrum of compound **54**

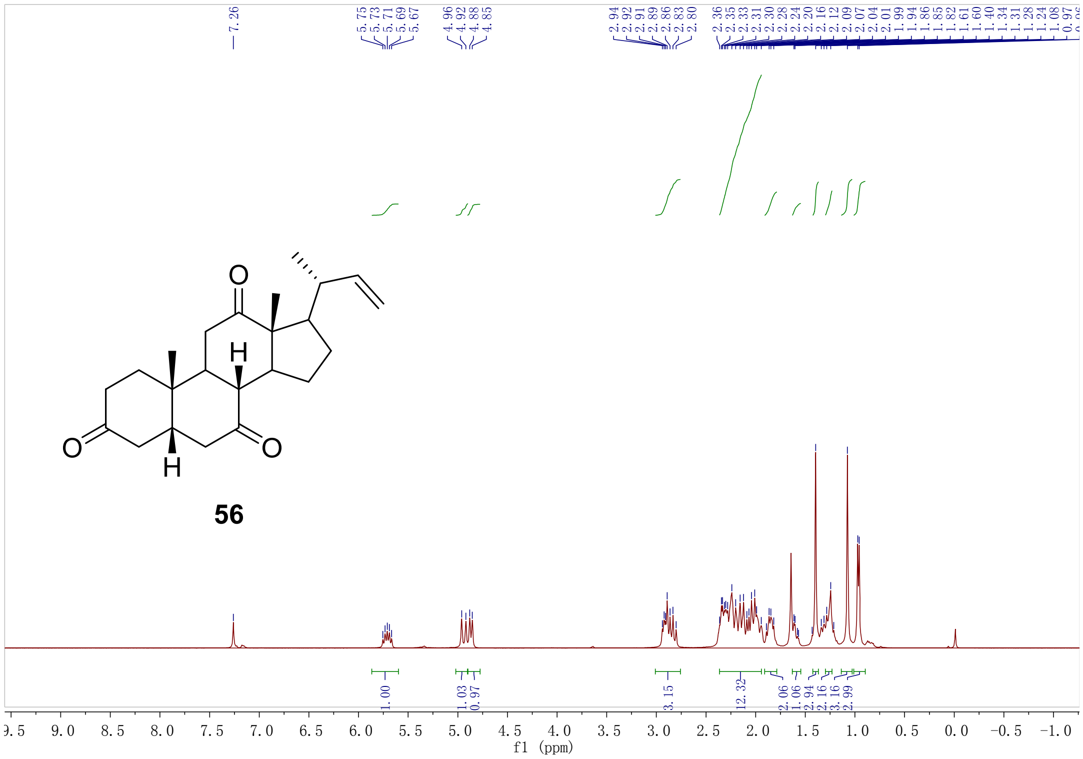

Supplementary Figure 120. ^{1}H NMR (400 MHz, $CDCl_3$) spectrum of compound **56**

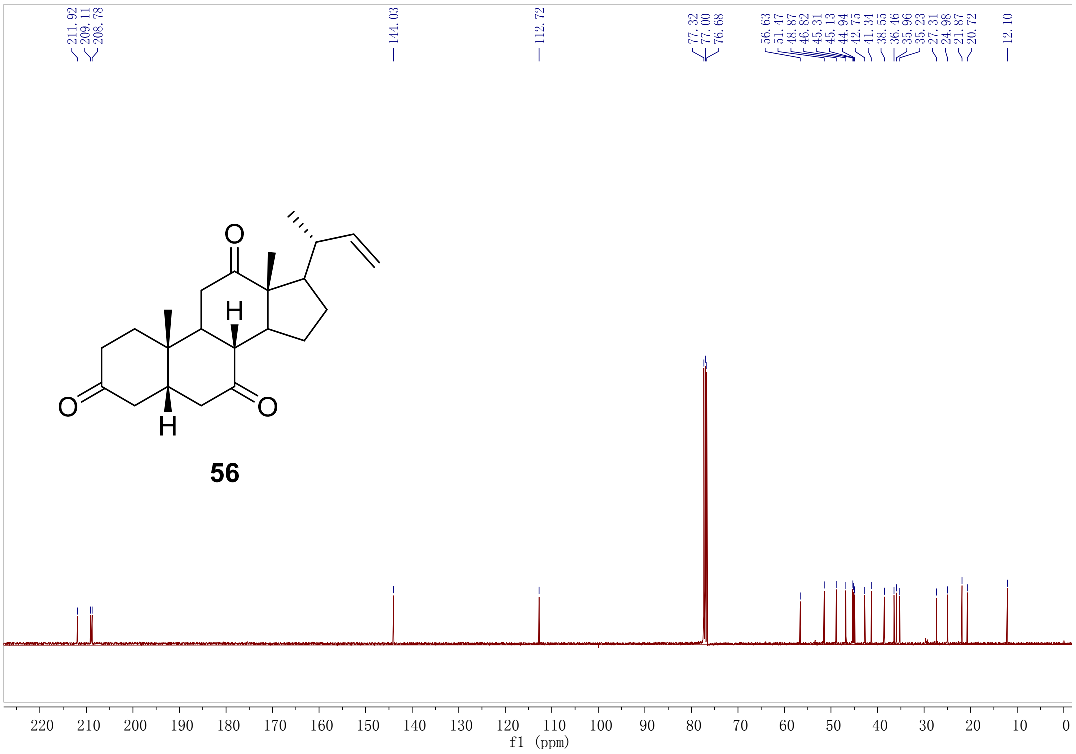

Supplementary Figure 121. ^{13}C NMR (400 MHz, $CDCl_3$) spectrum of compound **56**

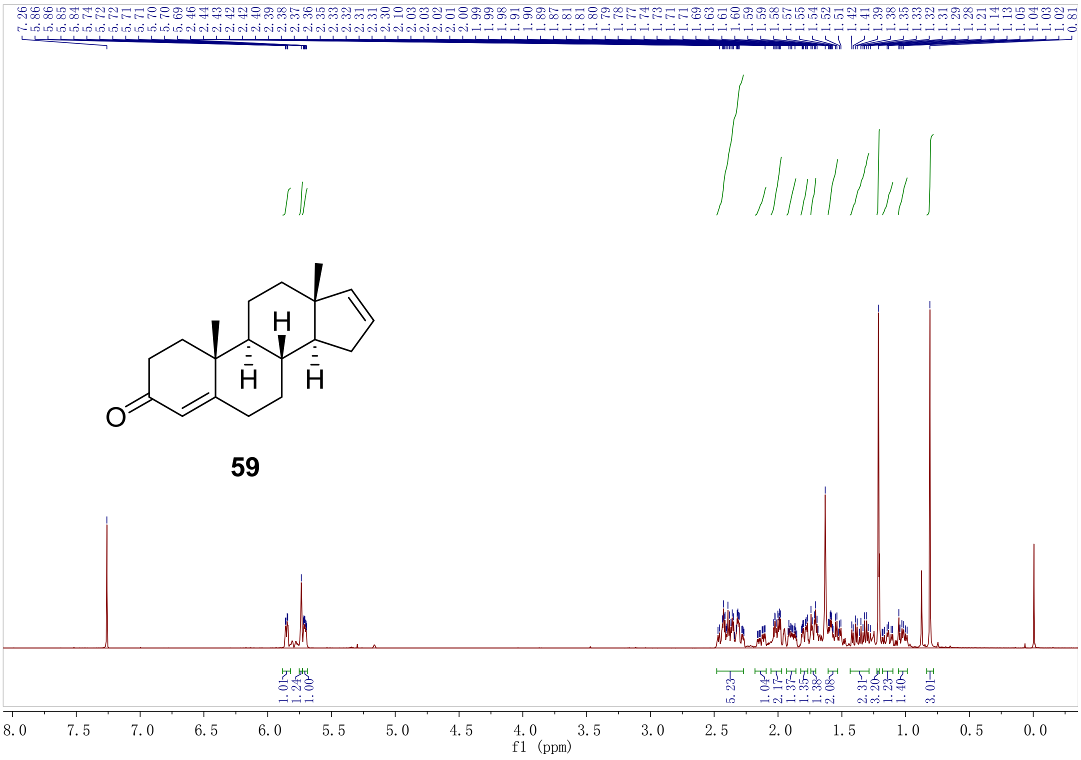

Supplementary Figure 122. ^{1}H NMR (400 MHz, $CDCl_3$) spectrum of compound **59**

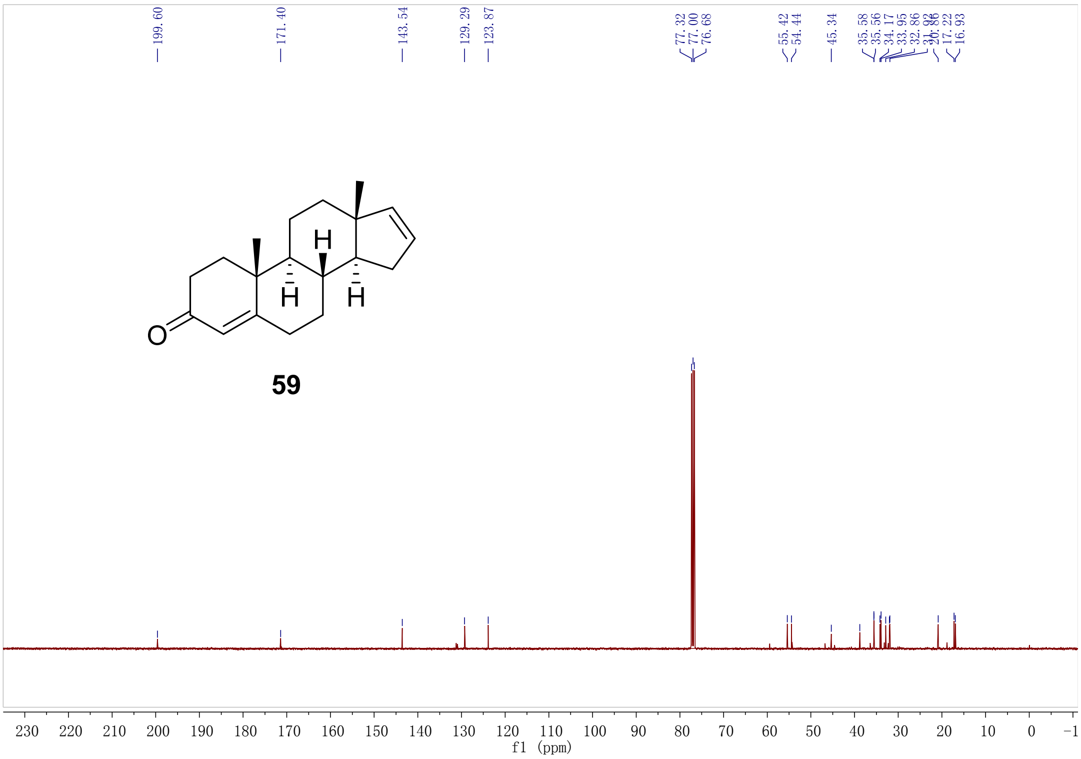

Supplementary Figure 123. ^{13}C NMR (400 MHz, $CDCl_3$) spectrum of compound **59**

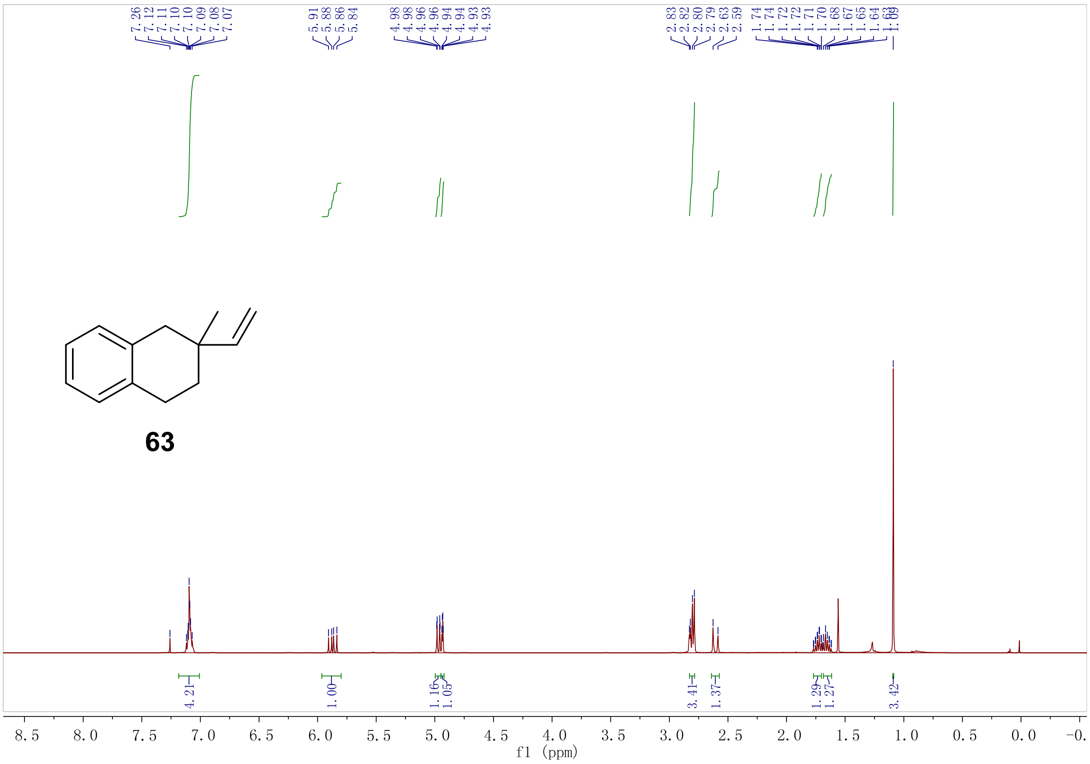

Supplementary Figure 124. ^{1}H NMR (400 MHz, $CDCl_3$) spectrum of compound **63**

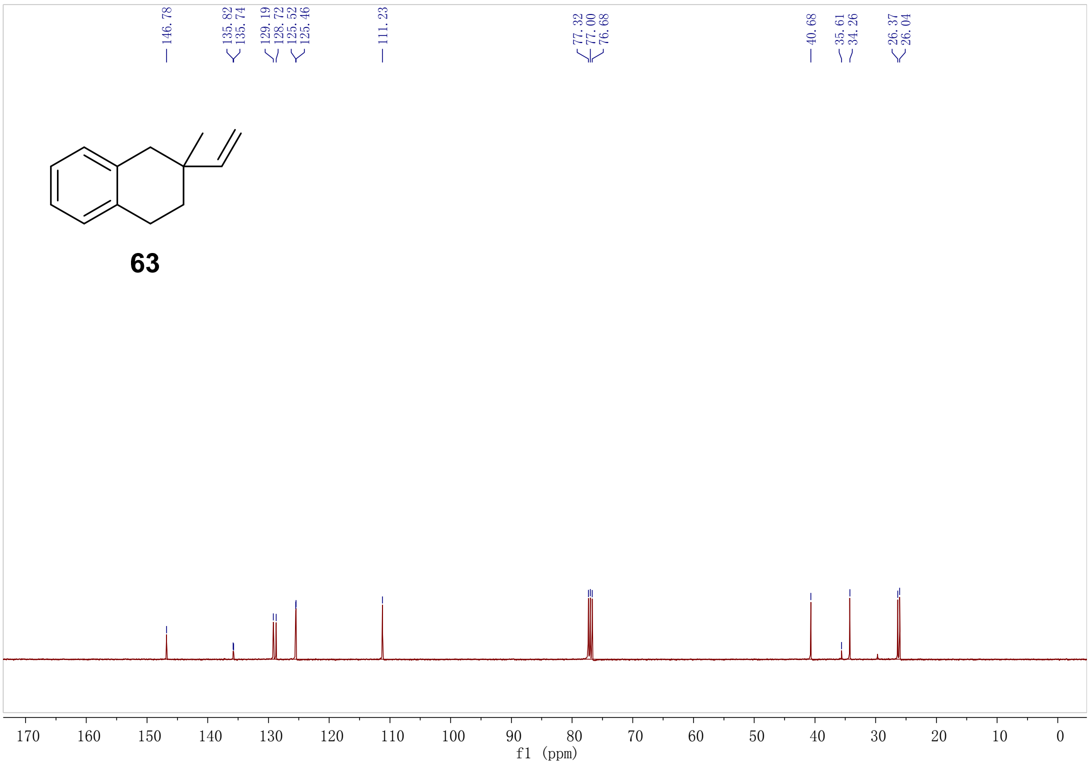

Supplementary Figure 125. ^{13}C NMR (400 MHz, $CDCl_3$) spectrum of compound **63**

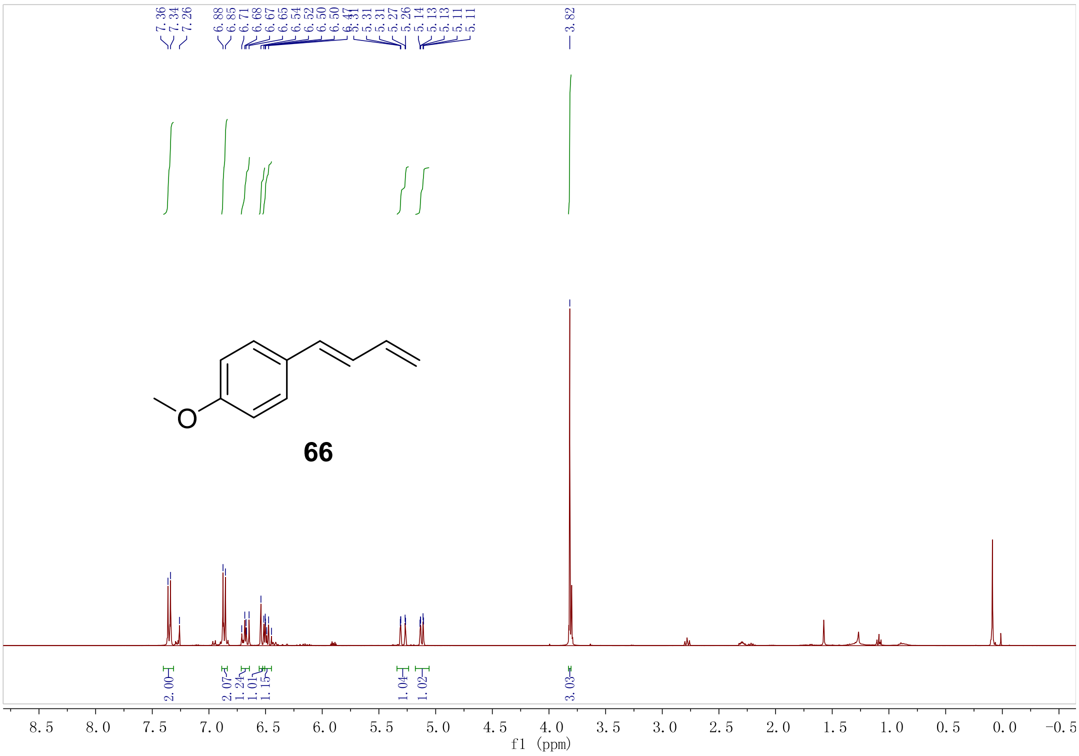

Supplementary Figure 126. ^{1}H NMR (400 MHz, $CDCl_3$) spectrum of compound **66**

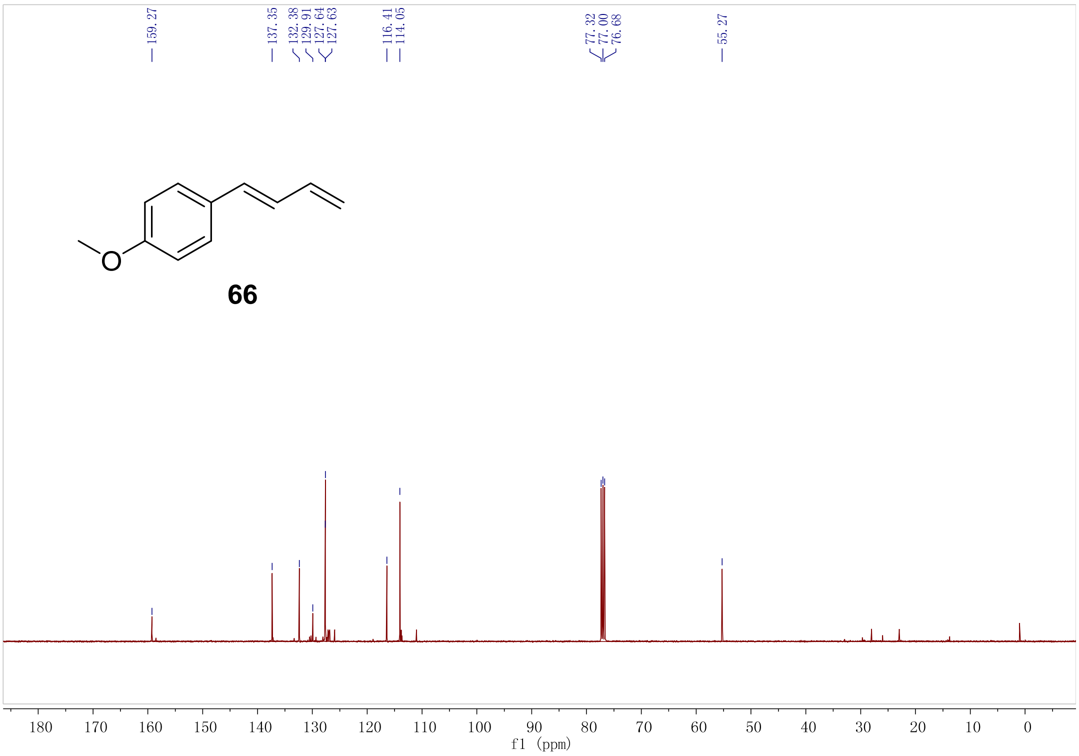

Supplementary Figure 127. ^{13}C NMR (400 MHz, $CDCl_3$) spectrum of compound **66**

Supplementary Methods

General Information

Unless otherwise noted, all reactions were performed under an argon atmosphere using flame-dried glassware. Toluene and cyclohexane were distilled over CaH_2.All new compounds were fully characterized. NMR-spectra were recorded on Bruker ARX-400 MHz. Mass spectra were conducted at Micromass Q-Tof instrument (ESI) and Agilent Technologies 5973N (EI). All reactions were carried out in flame-dried reaction vessels (25 mL) with Teflon screw caps under argon. Unless otherwise noted, materials obtained from commercial suppliers were used without further purification. $Ni(COD)_2$, K_3PO_4, KOAc, $Mg(OAc)_2$ and ICy•HCl were purchased from Acros without further purification. NaO^{t}Bu was purchased from Sigma-Aldrich. ICy was synthesized from ICy•HCl in the presence of NaO^{t}Bu.[1]

Preparation of Amide Substrates

Most of amides shown in Figure 3 were synthesized as follows:[2]

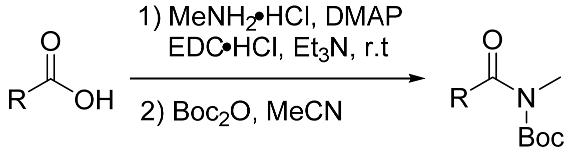

A mixture of the carboxylic acid (5.0 mmol, 1.0 equiv), $MeNH_2$•HCl (6.0 mmol, 1.2 equiv), 1-(3-dimethylaminopropyl)-3-ethylcarbodiimide hydrochloride (EDC•HCl: 6.0 mmol, 1.2 equiv), Et_3N (18.0 mmol, 3.6 equiv) and *N,N*-dimethylaminopyridine (DMAP: 6.0 mmol, 1.2 equiv) in CH_2Cl_2 (20 mL) was stirred for 2 h at room temperature. After quenching the reaction with $NaHCO_3$ aq., the mixture was extracted three times with CH_2Cl_2. The combined organic layer was dried over Na_2SO_4, filtrated, and then concentrated in vacuo. The resulting crude solid material was used in the subsequent step without further purification.

To a flask containing the crude material from the above step was added DMAP (2.0 mmol, 0.4 equiv) followed by acetonitrile (20 mL, 0.2 M). Boc_2O (10.0 mmol, 2.0 equiv) was added in one portion and the reaction vessel was flushed with Ar, then the reaction mixture was allowed to stir at 40 °C for 3 h. The reaction was quenched by

addition of 15 mL saturated aqueous $NaHCO_3$, transferred to a separatory funnel with EtOAc (25 mL) and H_2O (25 mL), and extracted with EtOAc (3 x 20 mL). The organic layers were combined, dried over Na_2SO_4, and evaporated under reduced pressure. The residue was purified by flash column chromatography to afford the corresponding amide.

***Trans*-methyl 4-((*tert*-butoxycarbonyl)(methyl)carbamoyl)cyclohexane-1-carboxylate (1)**

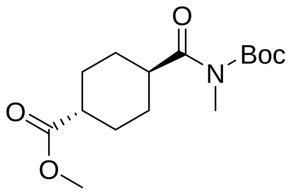

1

According to the general procedure, 5.0 mmol scale **1** (1.21 g, 81%) as a white solid was prepared from the corresponding carboxylic acid: **^{1}H NMR (400 MHz, $CDCl_3$)** δ 3.62 (s, 3H), 3.40 – 3.25 (m, 1H), 3.07 (s, 3H), 2.34 – 2.22 (m, 1H), 2.07 – 1.87 (m, 4H), 1.57 – 1.41 (m, 13H); **^{13}C NMR (101 MHz, $CDCl_3$)** δ 178.98, 175.99, 153.10, 82.91, 51.45, 43.85, 42.53, 31.82, 28.72, 28.15, 27.96; **HRMS m/z (ESI)** calcd for $C_{15}H_{25}NO_5$ $(M + Na)^+$ 322.1625, found 322.1629.

***Trans*-phenyl 4-((*tert*-butoxycarbonyl)(methyl)carbamoyl)cyclohexane-1-carboxylate (3)**

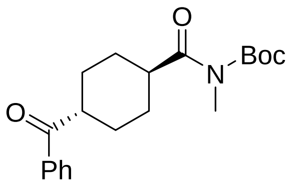

3

According to the general procedure, 5.0 mmol scale **3** (1.39 g, 77%) as a colourless oil was prepared from the corresponding carboxylic acid: **^{1}H NMR (400 MHz, $CDCl_3$)** δ 7.36 (t, J = 7.9 Hz, 2H), 7.21 (t, J = 7.4 Hz, 1H), 7.05 (dd, J = 5.3, 3.4 Hz, 2H), 3.49 – 3.37 (m, 1H), 3.13 (s, 3H), 2.62 – 2.50 (m, 1H), 2.22 (d, J = 11.2 Hz, 2H), 2.10 – 1.98 (m, 2H), 1.74 – 1.56 (m, 4H), 1.53 (d, J = 4.2 Hz, 9H); **^{13}C NMR (101 MHz, $CDCl_3$)** δ 178.94, 174.04, 153.14, 150.74, 129.32, 125.63, 121.44, 82.98, 43.85, 42.73, 31.87, 28.71, 28.14, 28.01; **HRMS m/z (ESI)** calcd for $C_{20}H_{27}NO_5$ $(M + Na)^+$ 384.1781, found 384.1784.

***Trans*-*tert*-butyl (4-(dimethylcarbamoyl)cyclohexane-1-carbonyl)(methyl)carbamate (5)**

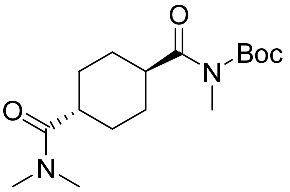

5

According to the general procedure, 5.0 mmol scale **5** (1.1 g, 71%) as a white solid was prepared from the corresponding carboxylic acid: **^{1}H NMR (400 MHz, CDCl$_3$)** δ 3.40 – 3.28 (m, 1H), 3.08 (s, 3H), 3.02 (s, 3H), 2.90 (s, 3H), 2.58 – 2.47 (m, 1H), 2.02 – 1.90 (m, 2H), 1.85 – 1.74 (m, 2H), 1.67 – 1.44 (m, 13H); **^{13}C NMR (101 MHz, CDCl$_3$)** δ 179.25, 175.42, 153.09, 83.09, 44.01, 39.89, 36.99, 35.46, 31.87, 28.96, 28.21, 27.97; **HRMS m/z (ESI)** calcd for $C_{16}H_{28}N_2O_4$ (M + Na)$^+$ 335.1941, found 335.1945.

***Trans-tert*-butyl (4-(benzyloxy)cyclohexane-1-carbonyl)(methyl)carbamate (7)**

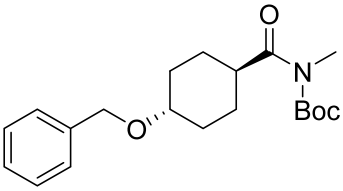

7

According to the general procedure, 5.0 mmol scale **7** (1.29 g, 74%) as a white solid was prepared from the corresponding carboxylic acid: **^{1}H NMR (400 MHz, CDCl$_3$)** δ 7.36 – 7.31 (m, 4H), 7.28 – 7.23 (m, 1H), 4.56 (s, 2H), 3.41 – 3.25 (m, 2H), 3.11 (s, 3H), 2.20 – 2.11 (m, 2H), 1.99 – 1.92 (m, 2H), 1.56 – 1.12 (m, 10H), 1.50 – 1.44 (m, 1H), 1.42 – 1.29 (m, 2H); **^{13}C NMR (101 MHz, CDCl$_3$)** δ 179.06, 153.13, 138.94, 128.27, 127.46, 127.34, 82.97, 76.81, 69.89, 43.95, 31.87, 31.57, 28.01, 27.99; **HRMS m/z (ESI)** calcd for $C_{20}H_{29}NO_4$ (M + Na)$^+$ 370.1989, found 370.1990.

***Trans-tert*-Butyl methyl(4-pentylcyclohexane-1-carbonyl)carbamate (9)**

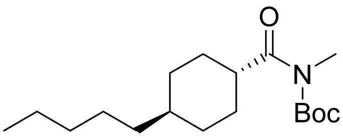

9

According to the general procedure, 5.0 mmol scale **9** (1.20 g, 77%) as a white solid was prepared from the corresponding carboxylic acid: **^{1}H NMR (400 MHz, CDCl$_3$)** δ 3.32 – 3.21 (m, 1H), 3.09 (s, 3H), 1.93 – 1.72 (m, 4H), 1.51 (s, 9H), 1.45 – 1.38 (m, 2H), 1.33 – 1.10 (m, 9H), 1.00 – 0.87 (m, 2H), 0.86 (t, *J* = 7.0 Hz, 3H); **^{13}C NMR (101 MHz, CDCl$_3$)** δ 179.80, 153.28, 82.70, 44.90, 37.24, 37.04, 32.49, 32.12, 31.86, 29.76, 28.02, 26.47, 22.62, 14.03; **HRMS m/z (ESI)** calcd for $C_{18}H_{33}NO_3$ (M + Na)$^+$ 334.2353, found 334.2357.

tert-Butyl (1-benzoylpiperidine-4-carbonyl)(methyl)carbamate (11)

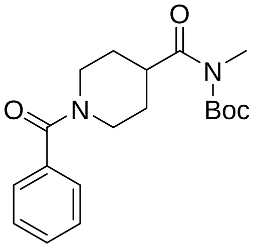

11

According to the general procedure, 5.0 mmol scale **11** (1.18 g, 68%) as a white solid was prepared from the corresponding carboxylic acid: **^{1}H NMR (400 MHz, $CDCl_3$)**, rotamers, δ 7.41 – 7.34 (m, 5H), 4.80 – 4.53 (m, 1H), 3.87 – 3.63 (m, 2H), 3.12 (s, 3H), 3.09 – 2.80 (m, 2H), 2.08 – 1.62 (m, 4H), 1.52 (s, 9H); **^{13}C NMR (101 MHz, $CDCl_3$)**, rotamers, δ 177.70, 170.30, 153.07, 136.13, 129.44, 128.39, 126.77, 83.14, 47.18, 42.73, 41.75, 31.89, 29.25, 28.80, 27.98; **HRMS m/z (ESI)** calcd for $C_{19}H_{26}N_2O_4$ $(M + Na)^+$ 369.1785, found 369.1789.

Benzyl 3-((tert-butoxycarbonyl)(methyl)carbamoyl)piperidine-1-carboxylate (13)

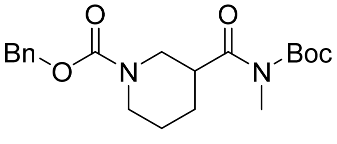

13

According to the general procedure, 5.0 mmol scale **13** (1.02 g, 54%) as a yellow solid was prepared from the corresponding carboxylic acid: **^{1}H NMR (400 MHz, $CDCl_3$)**, rotamers, δ 7.41 – 7.26 (m, 5H), 5.16 – 5.03 (m, 2H), 4.25 – 4.02 (m, 2H), 3.62 – 3.43 (m, 1H), 3.11 – 3.02 (m, 4H), 2.90 – 2.75 (m, 1H), 2.20 – 2.03 (s, 1H), 1.77 – 1.67 (s, 1H), 1.56 – 1.45 (m, 11H); **^{13}C NMR (101 MHz, $CDCl_3$)**, rotamers, δ 176.79, 155.09, 152.84, 136.76, 128.38, 127.85, 127.80, 83.20, 67.02, 46.49, 44.27, 43.09, 31.67, 28.29, 27.93, 24.54, 24.30; **HRMS m/z (ESI)** calcd for $C_{20}H_{28}N_2O_5$ $(M + Na)^+$ 399.1890, found 399.1893.

tert-Butyl (6-(benzyloxy)-4,4-dimethylhexanoyl)(methyl)carbamate (15)[3]

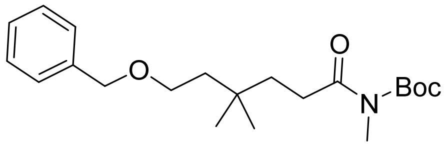

15

To a mixture of (((3-methylbut-3-en-1-yl)oxy)methyl)benzene (0.53 g, 3.0 mmol, 3.0 equiv) and $Fe(acac)_3$ (0.11 g, 0.30 mmol, 0.3 equiv) in DCE (8.0 mL) and ethylene glycol (2.0 mL) was added *tert*-butyl acryloyl(methyl)carbamate (0.19 g, 1.0 mol, 1.0 equiv), followed by $PhSiH_3$ (0.16 g, 1.5 mol, 1.5 equiv). The resulting mixture was heated to 60 °C with stirring for 90 min, then cooled to rt, and diluted with H_2O and brine. The aqueous layer was

extracted with Et_2O. The organic layers were combined, washed with brine, dried over $MgSO_4$, filtered, and concentrated under reduced pressure. The redresidue was then purified by column chromatography on silica gel (hexanes:EtOAc = 40:1) to furnish amide **15** (0.98g, 54%) as a colourless oil: **^{1}H NMR (400 MHz, $CDCl_3$)** δ 7.37 – 7.29 (m, 4H), 7.29 – 7.22 (m, 1H), 4.48 (s, 2H), 3.54 (t, *J* = 7.3 Hz, 2H), 3.12 (s, 3H), 2.91 – 2.77 (m, 2H), 1.64 – 1.56 (m, 4H), 1.52 (s, 9H), 0.92 (s, 6H); **^{13}C NMR (101 MHz, $CDCl_3$)** δ 176.62, 153.21, 138.54, 128.23, 127.47, 127.34, 82.63, 72.92, 67.29, 40.77, 36.95, 33.40, 31.76, 31.45, 28.00, 27.24; **HRMS m/z (ESI)** calcd for $C_{21}H_{33}NO_4$ $(M + Na)^+$ 386.2302, found 386.2306.

***tert*-Butyl (6-(4-cyanobutoxy)-4,4-dimethylhexanoyl)(methyl)carbamate (17)[3]**

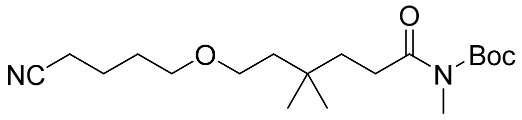

17

To a mixture of 5-((3-methylbut-3-en-1-yl) oxy)pentanenitrile (0.50 g, 3.0 mmol, 1.0 equiv) and $Fe(acac)_3$ (0.33 g, 0.90 mmol, 0.3 equiv) in DCE (16.0 mL) and ethylene glycol (3.0 mL) was added *tert*-butyl acryloyl(methyl)carbamate (1.71 g, 9.0 mol, 3.0 equiv), followed by $PhSiH_3$ (0.8 g, 7.5 mol, 2.5 equiv). The resulting mixture was heated to 60 °C with stirring for 120 min, then cooled to rt, and diluted with H_2O and brine. The aqueous layer was extracted with Et_2O. The organic layers were combined, washed with brine, dried over $MgSO_4$, filtered, and concentrated under reduced pressure. The redresidue was then purified by column chromatography on silica gel (hexanes:EtOAc = 5:1) to furnish amide **17** (0.65g, 61%) as a colourless oil: **^{1}H NMR (400 MHz, $CDCl_3$)** δ 3.44 – 3.37 (m, 4H), 3.08 (s, 3H), 2.82 – 2.75 (m, 2H), 2.34 (t, *J* = 6.8 Hz, 2H), 1.75 – 1.62 (m, 5H), 1.55 – 1.51 (m, 3H), 1.49 (s, 9H), 0.87 (s, 6H); **^{13}C NMR (101 MHz, $CDCl_3$)** δ 176.55, 153.18, 119.59, 82.61, 69.49, 67.71, 40.69, 36.85, 33.31, 31.66, 31.42, 28.55, 27.95, 27.18, 22.52, 16.85; **HRMS m/z (ESI)** calcd for $C_{19}H_{34}N_2O_4$ $(M + Na)^+$ 377.2411, found 377.2414.

***t*-Butyl (6-((5-fluoropentyl)oxy)-4,4-dimethylhexanoyl)(methyl)carbamate(19)[3]**

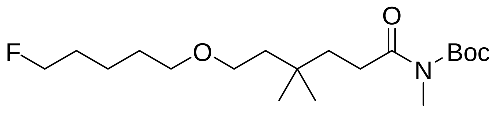

19

To a mixture of 1-fluoro-5-((3-methylbut-3-en-1-yl)oxy)pentane (0.52 g, 3.0 mmol, 1.0 equiv) and $Fe(acac)_3$ (0.33 g, 0.90 mmol, 0.3 equiv) in DCE (16.0 mL) and ethylene glycol (3.0 mL) was added *tert*-butyl acryloyl(methyl)carbamate (1.71 g, 9.0 mol, 3.0 equiv), followed by $PhSiH_3$ (0.8 g, 7.5 mol, 2.5 equiv). The resulting mixture was heated to 60 °C with stirring for 120 min, then cooled to rt, and diluted with H_2O and brine. The aqueous layer was extracted with Et_2O. The organic layers were combined, washed with brine, dried over $MgSO_4$, filtered, and concentrated under reduced pressure. The redresidue was then purified by column chromatography on silica gel (hexanes:EtOAc = 15:1) to furnish amide **19** (0.62g, 59%) as a colourless oil: **^{1}H NMR (400 MHz, $CDCl_3$)** δ 4.45 (t, J = 6.2 Hz, 1H), 4.33 (t, J = 6.1 Hz, 1H), 3.45 – 3.33 (m, 4H), 3.08 (s, 3H), 2.83 – 2.75 (m, 2H), 1.75 – 1.51 (m, 7H), 1.50 (s, 9H), 1.49 – 1.47 (m, 1H), 1.46 – 1.38 (m, 2H), 0.88 (s, 6H); **^{13}C NMR (101 MHz, $CDCl_3$)** 176.60, 153.20, 83.87 (d, J = 164.6 Hz), 82.60, 70.58, 67.59, 40.73, 36.91, 33.36, 31.67, 31.41, 30.12 (d, J = 19.2 Hz), 29.31, 27.96, 27.20, 21.88 (d, J = 5.1 Hz); **HRMS m/z (ESI)** calcd for $C_{19}H_{36}FNO_4$ $(M + Na)^+$ 384.2521, found 384.2523.

***tert*-Butyl methyl(2-methylhexadecanoyl)carbamate (21)**

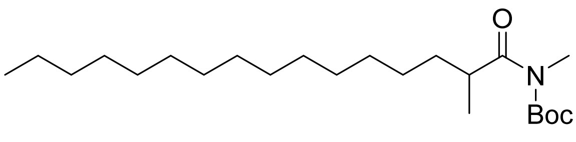

21

According to the general procedure, 5.0 mmol scale **21** (1.40g, 73%) as a colourless oil was prepared from 2-methylpalmitic acid: **^{1}H NMR (400 MHz, $CDCl_3$)** δ 3.60 – 3.48 (m, 1H), 3.10 (d, J = 2.2 Hz, 3H), 1.74 – 1.60 (m, 1H), 1.51 (d, J = 2.1 Hz, 9H), 1.29 – 1.18 (m, 25H), 1.12 (dd, J = 6.7, 2.3 Hz, 3H), 0.85 (dt, J = 6.9, 3.3 Hz, 3H); **^{13}C NMR (101 MHz, $CDCl_3$)** δ 180.60, 153.38, 82.69, 39.88, 34.53, 31.89, 31.81, 29.65, 29.49, 29.32, 27.98, 27.28, 22.65, 17.73, 14.07; **HRMS m/z (ESI)** calcd for $C_{23}H_{45}NO_3$ $(M + Na)^+$ 406.3292, found 406.3295.

***tert*-Butyl (3-(4-methoxyphenyl)propanoyl)(methyl)carbamate (23)**

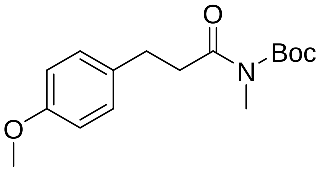

23

According to the general procedure, 5.0 mmol scale **23** (1.11 g, 76%) as a white solid was prepared from the corresponding carboxylic acid: **^{1}H NMR (400 MHz, $CDCl_3$)** δ 7.15 (d, J = 8.6 Hz, 2H), 6.82 (d, J = 8.5 Hz, 2H), 3.77 (d, J = 0.7 Hz, 3H), 3.21 – 3.09 (m, 5H), 2.90 (t, J = 7.7 Hz, 2H), 1.51 (s, 9H); **^{13}C NMR (101 MHz, $CDCl_3$)** δ 175.44, 157.84, 153.26, 133.34, 129.37, 113.74, 82.81, 55.19, 40.16, 31.40, 30.25, 27.98. **HRMS m/z (ESI)** calcd for $C_{16}H_{23}NO_4$ $(M + Na)^+$ 316.1519, found 316.1522.

***tert*-Butyl (3-(4-(4-cyanobutoxy)phenyl)propanoyl)(methyl)carbamate (25)**

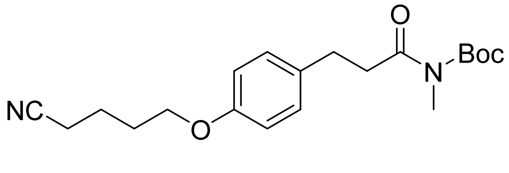

25

According to the general procedure, 5.0 mmol scale **25** (1.24 g, 69%) as a white solid was prepared from the corresponding carboxylic acid: **^{1}H NMR (400 MHz, $CDCl_3$)** δ 7.14 (d, *J* = 8.5 Hz, 2H), 6.79 (d, *J* = 8.5 Hz, 2H), 3.97 (t, *J* = 5.6 Hz, 2H), 3.18 – 3.13 (m, 2H), 3.12 (s, 3H), 2.89 (t, *J* = 7.7 Hz, 2H), 2.43 (t, *J* = 6.7 Hz, 2H), 1.97 – 1.81 (m, 4H), 1.50 (s, 9H); **^{13}C NMR (101 MHz, $CDCl_3$)** δ 175.36, 156.91, 153.23, 133.61, 129.41, 119.48, 114.26, 82.80, 66.55, 40.12, 31.38, 30.20, 28.13, 27.96, 22.40, 16.90. **HRMS m/z (ESI)** calcd for $C_{20}H_{28}N_2O_4$ $(M + Na)^+$ 383.1941, found 383.1942.

***tert*-Butyl methyl(2-(4-((2-oxocyclopentyl)methyl)phenyl)propanoyl)carbamate (27)**

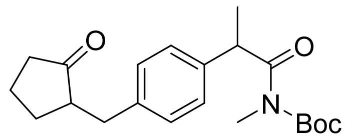

27

According to the general procedure, 5.0 mmol scale **27** (1.29g, 72%) as a colourless oil was prepared from the Loxoprofen: **^{1}H NMR (400 MHz, $CDCl_3$)** δ 7.20 – 7.12 (m, 2H), 7.10 – 7.02 (m, 2H), 5.02 – 4.90 (m, 1H), 3.16 – 3.03 (m, 4H), 2.54 – 2.40 (m, 1H), 2.30 (d, *J* = 7.8 Hz, 2H), 2.39 – 2.23 (m, 2H), 1.99 – 1.86 (m, 1H), 1.78 – 1.63 (m, 1H), 1.62 – 1.49 (m, 1H), 1.42 – 1.36 (m, 12H); **^{13}C NMR (101 MHz, $CDCl_3$)** δ 177.72, 153.12, 139.64, 138.27, 128.89,

128.80, 127.91, 82.72, 50.92, 44.99, 38.13, 35.15, 32.15, 29.14, 27.82, 20.48, 19.96; **HRMS m/z (ESI)** calcd for $C_{21}H_{29}NO_4$ $(M + Na)^+$ 382.1989, found 382.1992.

tert-Butyl (3-(benzo[d][1,3]dioxol-5-yl)-2-methylpropanoyl)(methyl)carbamate (29)

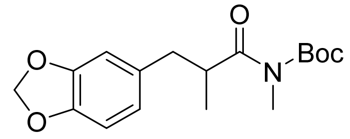

29

According to the general procedure, 5.0 mmol scale **29** (1.12g, 70%) as a white solid was prepared from the corresponding carboxylic acid: **^{1}H NMR (400 MHz, $CDCl_3$)** δ 6.73 – 6.65 (m, 2H), 6.61 (d, *J* = 7.9 Hz, 1H), 5.89 (s, 2H), 3.98 – 3.79 (m, 1H), 3.05 (s, 3H), 2.96 (dd, *J* = 13.4, 6.7 Hz, 1H), 2.50 (dd, *J* = 13.4, 7.9 Hz, 1H), 1.50 (s, 9H), 1.11 (d, *J* = 6.7 Hz, 3H); **^{13}C NMR (101 MHz, $CDCl_3$)** δ 179.65, 153.20, 147.36, 145.74, 133.72, 121.96, 109.37, 107.92, 100.66, 82.78, 41.98, 40.15, 31.76, 27.94, 17.16; **HRMS m/z (ESI)** calcd for $C_{17}H_{23}NO_5$ $(M + Na)^+$ 344.1468, found 344.1471.

tert-Butyl (2-cyclopentyl-2-phenylacetyl)(methyl)carbamate (31)

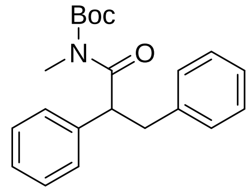

31

According to the general procedure, 5.0 mmol scale **31** (1.34g, 79%) as a colourless oil was prepared from the corresponding carboxylic acid: **^{1}H NMR (400 MHz, $CDCl_3$)** δ 7.32 – 7.06 (m, 10H), 5.37 – 5.28 (m, 1H), 3.45 (dd, *J* = 12.0, 8.0 Hz, 1H), 3.02 (s, 3H), 3.01 – 2.94 (m, 1H), 1.39 (s, 9H); **^{13}C NMR (101 MHz, $CDCl_3$)** δ 176.26, 152.97, 139.67, 139.42, 129.08, 128.52, 128.20, 128.05, 126.84, 125.96, 82.68, 52.61, 40.87, 32.02, 27.79; **HRMS m/z (ESI)** calcd for $C_{21}H_{25}NO_3$ $(M + Na)^+$ 362.1727, found 362.1731.

tert-Butyl methyl(3-methyl-2-phenylbutanoyl)carbamate (33)

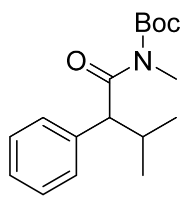

33

According to the general procedure, 5.0 mmol scale **33** (1.14 g, 78%) as a white solid was prepared from the corresponding carboxylic acid: **^{1}H NMR (400 MHz, $CDCl_3$)** δ 7.40 – 7.33 (m,

2H), 7.31 – 7.24 (m, 2H), 7.24 – 7.18 (m, 1H), 4.69 (d, *J* = 10.4 Hz, 1H), 3.07 (s, 3H), 2.53 – 2.39 (m, 1H), 1.49 (s, 9H), 1.02 (d, *J* = 6.5 Hz, 3H), 0.67 (d, *J* = 6.7 Hz, 3H); **^{13}C NMR (101 MHz, CDCl$_3$)** δ 177.18, 153.39, 139.19, 129.06, 128.12, 126.81, 82.71, 58.15, 32.55, 32.06, 27.96, 21.71, 20.32; **HRMS m/z (ESI)** calcd for $C_{17}H_{25}NO_3$ (M + Na)$^+$314.1727, found 314.1730.

tert-Butyl (2-cyclopentyl-2-phenylacetyl)(methyl)carbamate (35)

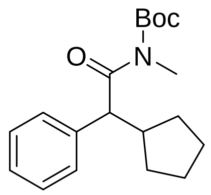

35

According to the general procedure, 5.0 mmol scale **35** (1.21 g, 76%) as a white solid was prepared from the corresponding carboxylic acid: **^{1}H NMR (400 MHz, CDCl$_3$)** δ 7.38 – 7.34 (m, 2H), 7.30 – 7.23 (m, 2H), 7.23 – 7.17 (m, 1H), 4.80 (d, *J* = 10.7 Hz, 1H), 3.07 (s, 3H), 2.77 – 2.54 (m, 1H), 2.03 – 1.84 (m, 1H), 1.73 – 1.53 (m, 3H), 1.51 – 1.38 (m, 10H), 1.37 – 1.12 (m, 2H), 1.10 – 0.96 (m, 1H); **^{13}C NMR (101 MHz, CDCl$_3$)** δ 177.17, 153.33, 139.75, 128.74, 128.09, 126.70, 82.62, 56.27, 44.51, 32.06, 31.68, 30.73, 27.91, 25.11, 24.78; **HRMS m/z (ESI)** calcd for $C_{19}H_{27}NO_3$ (M + Na)$^+$ 340.1883, found 340.1882.

tert-Butyl methyl(2-methyl-2-phenylpropanoyl)carbamate (37)

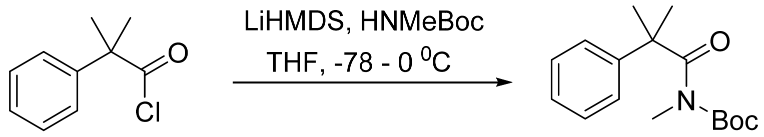

37

To a solution of HNMeBoc (5.0 mmol, 1 equiv) in dry THF, LiHMDS (7.5 mmol, 1.5 equiv) was added dropwise with continuous stirring at -78 0C. After stirring for 1 h, the acid chloride (6.0 mmol, 1.2 equiv) was added dropwise and allowed to stir for 1 h. The reaction was warmed to 0 ^{0}C and allowed to stir for 1 h. The reaction mixture was quenched with saturated solution of NH_4Cl. The reaction mixture was diluted with water and extracted with EtOAc (2 x 20 mL). The combined organic layer was dried over Na_2SO_4, filtrated, and then concentrated in vacuo. The residue was purified by flash column chromatography to afford the corresponding amide **37** (0.99g, 72%)

as a colourless oil: **^{1}H NMR (400 MHz, $CDCl_3$)** δ 7.34 – 7.22 (m, 2H), 7.20 – 7.10 (m, 3H), 3.12 (s, 3H), 1.66 (s, 6H), 1.12 (s, 9H); **^{13}C NMR (101 MHz, $CDCl_3$)** δ 181.13, 152.45, 145.92, 127.95, 125.71, 125.41, 82.18, 50.49, 33.72, 29.08, 27.39; **HRMS m/z (ESI)** calcd for $C_{16}H_{23}NO_3$ $(M + Na)^+$ 300.1570, found 300.1574.

***tert*-Butyl methyl(1-phenylcyclopentane-1-carbonyl)carbamate (39)**

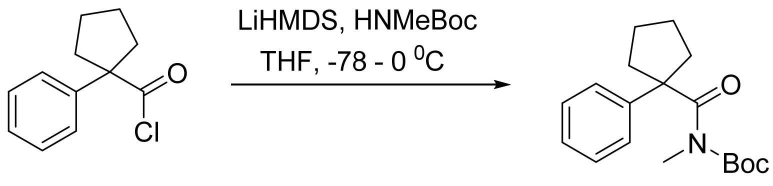

39

To a solution of HNMeBoc (5.0 mmol, 1 equiv) in dry THF, LiHMDS (7.5 mmol, 1.5 equiv) was added dropwise with continuous stirring at -78 ^{0}C. After stirring for 1 h, the acid chloride the acid chloride (6.0 mmol, 1.2 equiv) was added dropwise and allowed to stir for 1 h. The reaction was warmed to 0 ^{0}C and allowed to stir for 1 h. The reaction mixture was quenched with saturated solution of NH_4Cl. The reaction mixture was diluted with water and extracted with EtOAc (2 x 20 mL). The combined organic layer was dried over Na_2SO_4, filtrated, and then concentrated in vacuo. The residue was purified by flash column chromatography to afford the corresponding amide **35** (1.15g, 76%) as a colourless oil: **^{1}H NMR (400 MHz, $CDCl_3$)** δ 7.25 – 7.17 (m, 2H), 7.14 – 7.07 (m, 3H), 3.09 (s, 3H), 2.53 – 2.41 (m, 2H), 2.25 – 2.06 (m, 2H), 1.83 – 1.58 (m, 4H), 1.13 (s, 9H); **^{13}C NMR (101 MHz, $CDCl_3$)** δ 179.58, 152.29, 145.09, 127.81, 125.61, 125.50, 81.99, 61.69, 39.38, 33.37, 28.00, 27.42, 25.19; **HRMS m/z (ESI)** calcd for $C_{18}H_{25}NO_3$ $(M + Na)^+$ 326.1727, found 326.1730.

***tert*-Butyl (4-(4-methoxyphenyl)butanoyl)(methyl)carbamate (41)**

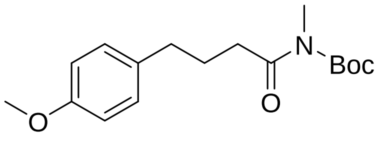

41

According to the general procedure, 5.0 mmol scale **41** (1.23g, 80%) as a colourless oil was prepared from the corresponding carboxylic acid: **^{1}H NMR (400 MHz, $CDCl_3$)** δ 7.10 (d, *J* = 8.2 Hz, 2H), 6.91 – 6.75 (m, 2H), 3.85 – 3.72 (m, 3H), 3.11 (s, 3H), 2.86 (t, *J* = 7.4 Hz, 2H), 2.60 (t, *J* =

7.7 Hz, 2H), 2.07 – 1.86 (m, 2H), 1.50 (s, 9H); **^{13}C NMR (101 MHz, $CDCl_3$)** δ 175.89, 157.70, 153.25, 133.94, 129.27, 113.65, 82.77, 55.14, 37.58, 34.29, 31.36, 27.95, 26.94; **HRMS m/z (ESI)** calcd for $C_{17}H_{25}NO_4$ (M + Na)$^+$ 330.3792, found 330.3794.

***tert*-Butyl methyl(4-phenylpentanoyl)carbamate (43)**

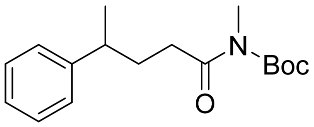

43

According to the general procedure, 5.0 mmol scale **43** (1.08g, 74%) as a colourless oil was prepared from the corresponding carboxylic acid: **^{1}H NMR (400 MHz, $CDCl_3$)** δ 7.31 – 7.25 (m, 2H), 7.20 – 7.14 (m, 3H), 3.09 (s, 3H), 2.84 – 2.62 (m, 3H), 1.99 – 1.87 (m, 2H), 1.45 (s, 9H), 1.27 (d, J = 7.0 Hz, 3H); **^{13}C NMR (101 MHz, $CDCl_3$)** δ 175.98, 153.17, 146.72, 128.31, 126.96, 125.95, 82.75, 39.38, 36.45, 33.37, 31.36, 27.91, 22.36; **HRMS m/z (ESI)** calcd for $C_{17}H_{25}NO_3$ (M + Na)+ 314.1727, found 314.1729.

***tert*-Butyl methyl(tetradecanoyl)carbamate (45)**

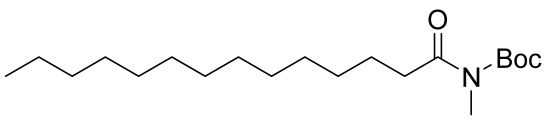

45

According to the general procedure, 5.0 mmol scale **45** (1.37g, 80%) as a colourless oil was prepared from myristic acid: **^{1}H NMR (400 MHz, $CDCl_3$)** δ 3.11 (s, 3H), 2.82 (t, J = 7.6 Hz, 2H), 1.65 – 1.56 (m, 2H), 1.51 (s, 9H), 1.34 – 1.16 (m, 20H), 0.86 (t, J = 6.8 Hz, 3H); **^{13}C NMR (101 MHz, $CDCl_3$)** δ 176.35, 153.37, 82.70, 38.28, 31.89, 31.39, 29.64, 29.63, 29.61, 29.59, 29.49, 29.45, 29.32, 29.26, 28.01, 25.16, 22.65, 14.07; **HRMS m/z (ESI)** calcd for $C_{20}H_{39}NO_3$ (M + Na)$^+$ 364.2822, found 364.2826.

***tert*-Butyl methyl(palmitoyl)carbamate (47)**

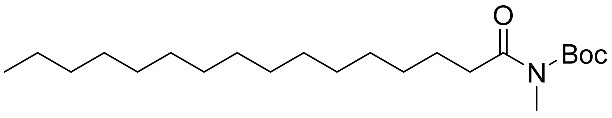

47

According to The general procedure, 5.0 mmol scale **47** (1.46g, 79%) as a colourless oil was prepared from palmitic acid: **^{1}H NMR (400 MHz, $CDCl_3$)** δ 3.15 – 3.06 (m, 3H),

2.82 (t, J = 7.5 Hz, 2H), 1.68 – 1.56 (m, 2H), 1.55 – 1.47 (m, 9H), 1.26 (d, J = 17.6 Hz, 24H), 0.86 (t, J = 5.5 Hz, 3H); **^{13}C NMR (101 MHz, CDCl$_3$)** δ 176.33, 153.36, 82.68, 38.28, 31.88, 31.37, 29.65, 29.59, 29.48, 29.44, 29.32, 29.25, 28.00, 27.83, 25.16, 22.64, 14.06; **HRMS m/z (ESI)** calcd for $C_{22}H_{43}NO_3$ (M + Na)$^+$ 392.3135, found 392.3139.

***tert*-Butyl (Z)-docos-13-enoyl(methyl)carbamate (49)**

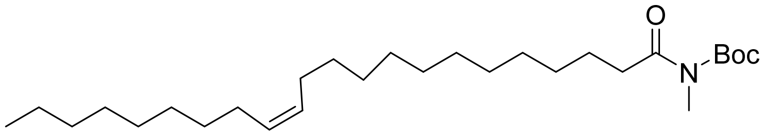

49

According to the general procedure, 5.0 mmol scale **49** (1.76g, 78%) as a colourless oil was prepared from cis-13-Docosenoic acid: **^{1}H NMR (400 MHz, CDCl$_3$)** δ 5.45 – 5.26 (m, 2H), 3.11 (s, 3H), 2.82 (t, J = 7.6 Hz, 2H), 2.06 – 1.93 (m, 4H), 1.66 – 1.57 (m, 2H), 1.52 (s, 9H), 1.46 (d, J = 0.7 Hz, 1H), 1.36 – 1.20 (m, 27H), 0.87 (t, J = 6.7 Hz, 3H); **^{13}C NMR (101 MHz, CDCl$_3$)** δ 176.36, 153.37, 129.86, 82.71, 38.29, 31.88, 31.40, 29.75, 29.67, 29.61, 29.59, 29.53, 29.50, 29.46, 29.29, 28.02, 27.18, 25.17, 22.65, 14.07; **HRMS m/z (ESI)** calcd for $C_{28}H_{53}NO_3$ (M + Na)$^+$ 474.3918, found 474.3919.

***tert*-Butyl methyl(12-(3-octyloxiran-2-yl)dodecanoyl)carbamate (51)**

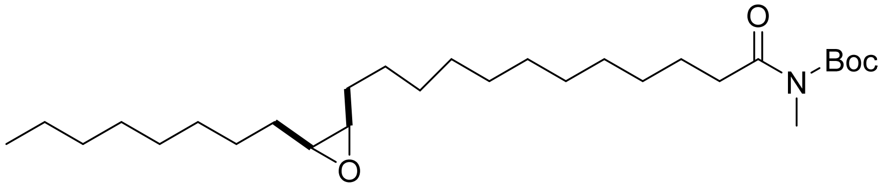

51

According to the general procedure, 5.0 mmol scale **51** (1.57g, 67%) as a colourless oil was prepared from the corresponding carboxylic acid: **^{1}H NMR (400 MHz, CDCl$_3$)** δ 3.13 (s, 3H), 2.94 – 2.87 (m, 2H), 2.86 – 2.80 (m, 2H), 1.68 – 1.60 (m, 2H), 1.53 (s, 9H), 1.51 – 1.46 (m, 6H), 1.37 – 1.22 (m, 26H), 0.88 (t, J = 6.8 Hz, 3H); **^{13}C NMR (101 MHz, CDCl$_3$)** δ 176.26, 153.32, 82.64, 57.14, 38.23, 31.78, 31.34, 29.62, 29.48, 29.46, 29.43, 29.40, 29.21, 29.15, 27.97, 27.76, 26.53, 25.11,

22.59, 14.02; **HRMS m/z (ESI)** calcd for $C_{28}H_{53}NO_3$ $(M + Na)^+$ 490.3867, found 490.3869.

***tert*-Butyl((*4R*)-4-((*3R,5R,8R,10S,13R,17R*)-3-methoxy-10,13-dimethylhexadecahydro-1*H*-cyclopenta[a]phenanthren-17-yl)pentanoyl)(methyl)carbamate (53)**

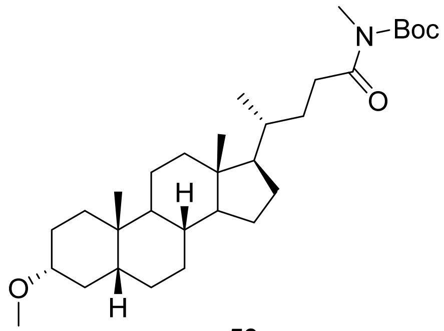

53

According to the general procedure, 5.0 mmol scale **47** (1.96 g, 78%) as a white solid was prepared from the derivative of lithocholic acid: **^{1}H NMR (400 MHz, $CDCl_3$)** δ 3.33 (s, 3H), 3.20 – 3.10 (m, 1H), 3.11 (s, 3H), 2.97 – 2.67 (m, 2H), 1.96 – 1.65 (m, 7H), 1.59 – 1.48 (m, 11H), 1.46 – 1.17 (m, 11H), 1.17 – 0.98 (m, 5H), 0.97 – 0.82 (m, 7H), 0.62 (s, 3H); **^{13}C NMR (101 MHz, $CDCl_3$)** δ 176.77, 153.32, 82.67, 80.36, 56.41, 56.06, 55.48, 42.67, 41.99, 40.27, 40.11, 35.79, 35.40, 35.25, 35.23, 34.83, 32.70, 31.44, 31.05, 28.15, 28.04, 27.28, 26.73, 26.34, 24.19, 23.37, 20.75, 18.50, 11.98; **HRMS m/z (ESI)** calcd for $C_{31}H_{53}NO_4$ $(M + Na)^+$ 526.3867, found 526.3870.

***tert*-Butyl ((*4R*)-4-((*5S,8R,10S,13R,17R*)-10,13-dimethyl-3,7,12-trioxohexadecahydro-1H-cyclopenta[*a*]phenanthren-17-yl)pentanoyl)(methyl)carbamate (55)**

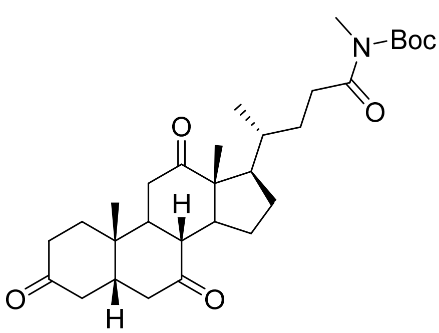

55

According to the general procedure, 5.0 mmol scale **55** (1.88 g, 73%) as a white solid was prepared from dehydrocholic acid: **^{1}H NMR (400 MHz, $CDCl_3$)** δ 3.11 (s, 3H), 2.99 – 2.74 (m, 5H), 2.36 – 1.93 (m, 12H), 1.89 – 1.78 (m, 2H), 1.72 – 1.58 (m, 2H), 1.52 (s, 9H), 1.41 – 1.27 (m, 6H), 1.06 (s, 3H), 0.85 (d, *J* = 6.4 Hz, 3H); **^{13}C NMR (101 MHz, $CDCl_3$)** δ 211.96, 209.05, 208.73, 176.53, 153.32, 82.79, 56.89, 51.71, 48.98, 46.82, 45.81, 45.51, 44.95, 42.76, 38.61, 36.45, 35.97, 35.54, 35.52, 35.25,

31.45, 30.55, 28.04, 27.55, 25.14, 21.87, 18.89, 11.84; **HRMS m/z (ESI)** calcd for $C_{30}H_{45}NO_6$ (M + Na)$^+$ 538.3139, found 538.3142.

***tert*-Butyl ((*8S,9S,10R,13S,14S*)-10,13-dimethyl-3-oxo-2,3,6,7,8,9,10,11,12,13,14,15,16,17-tetradecahydro-1H-cyclopenta[*a*]phenanthrene-17-carbonyl)(methyl)carbamate (58)**

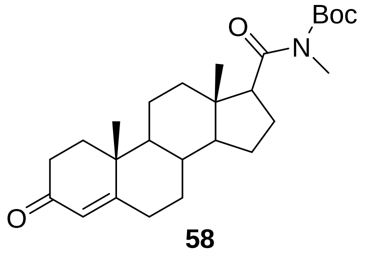

58

According to the general procedure, 5.0 mmol scale **58** (1.55 g, 72%) as a white solid was prepared from the testosterone 17*β*-carboxylic acid: **^{1}H NMR (400 MHz, $CDCl_3$)** δ 5.71 (s, 1H), 3.93 (t, *J* = 9.2 Hz, 1H), 3.10 (s, 3H), 2.50 – 2.16 (m, 5H), 2.04 – 1.94 (m, 1H), 1.89 – 1.63 (m, 6H), 1.53 – 1.51 (m, 1H), 1.50 (s, 9H), 1.47 – 1.32 (m, 3H), 1.23 – 1.13 (m, 4H), 1.11 – 0.90 (m, 2H), 0.66 (s, 3H); **^{13}C NMR (101 MHz, $CDCl_3$)** δ 199.46, 177.28, 171.11, 154.01, 123.84, 82.54, 55.42, 54.19, 53.59, 45.25, 38.56, 37.34, 35.73, 35.69, 33.90, 32.80, 32.27, 31.90, 28.03, 24.47, 23.97, 20.91, 17.31, 13.88; **HRMS m/z (ESI)** calcd for $C_{26}H_{39}NO_4$ (M + Na)$^+$ 452.2771, found 452.2774.

***tert*-Butyl methyl(2-methyl-1,2,3,4-tetrahydronaphthalene-2-carbonyl)carbamate (62)**[3]

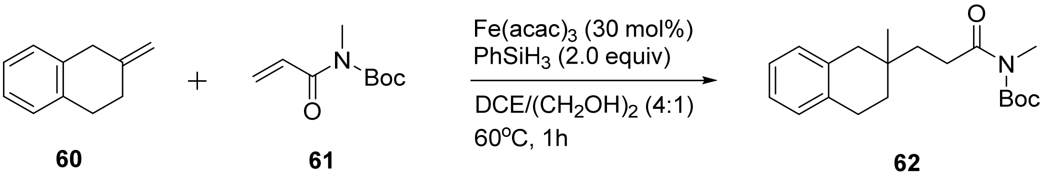

To a mixture of 2-methylene-1,2,3,4-tetrahydronaphthalene **60** (0.17 g, 1.2 mmol, 1.0 equiv) and $Fe(acac)_3$ (0.13 g, 0.36 mol, 0.3 equiv) in DCE (8.0 mL) and ethylene glycol (2.0 mL) was added *tert*-butyl acryloyl(methyl)carbamate **55** (0.56 g, 3.0 mol, 2.5 equiv), followed by $PhSiH_3$ (0.26 g, 2.4 mol, 2.0 equiv). The resulting mixture was heated to 60 °C with stirring for 60 min, then cooled to rt, and diluted with H_2O and brine. The aqueous layer was extracted with Et_2O. The organic layers were combined, washed with brine, dried over $MgSO_4$, filtered, and concentrated under

reduced pressure. The redresidue was then purified by column chromatography on silica gel (hexanes:EtOAc = 40:1) to furnish amide **62** (0.27 g, 67%) as a colourless oil: **^{1}H NMR (400 MHz, CDCl$_3$)** δ 7.15 – 6.99 (m, 4H), 3.12 (s, 3H), 2.92 (dd, *J* = 10.4, 6.4 Hz, 2H), 2.80 (t, *J* = 6.7 Hz, 2H), 2.57 (dd, *J* = 39.9, 16.3 Hz, 2H), 1.70 – 1.61 (m, 4H), 1.54 (s, 9H), 0.96 (s, 3H); **^{13}C NMR (101 MHz, CDCl$_3$)** δ 176.72, 153.33, 135.89, 135.77, 129.53, 128.71, 125.48, 125.42, 82.73, 41.99, 36.01, 33.85, 33.06, 31.63, 31.55, 28.06, 26.02, 24.16; **HRMS m/z (ESI)** calcd for $C_{20}H_{29}NO_3$ (M + Na)$^+$ 354.2040, found 354.2044.

***tert*-Butyl (2-cyclopropyl-2-(4-methoxyphenyl)acetyl)(methyl)carbamate (64)**

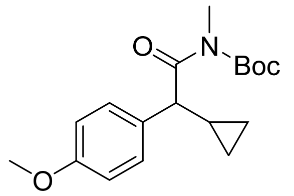

64

According to the general procedure, 5.0 mmol scale **64** (1.09g, 68%) as a colourless oil was prepared from the corresponding carboxylic acid: **^{1}H NMR (400 MHz, CDCl$_3$)** 7.25 (d, *J* = 8.7 Hz, 2H), 6.83 (d, *J* = 8.8 Hz, 2H), 4.20 (d, *J* = 9.8 Hz, 1H), 3.78 (s, 3H), 3.10 (s, 3H), 1.50 – 1.46 (m, 1H), 1.46 – 1.41 (m, 9H), 0.69 – 0.55 (m, 1H), 0.52 – 0.40 (m, 1H), 0.34 – 0.25 (m, 1H), 0.22 – 0.13 (m, 1H); **^{13}C NMR (101 MHz, CDCl$_3$)** δ 177.43, 158.46, 153.25, 132.21, 129.25, 113.66, 82.76, 55.16, 32.05, 27.88, 27.65, 15.57, 4.90, 3.93.

Initial Survey and Optimization

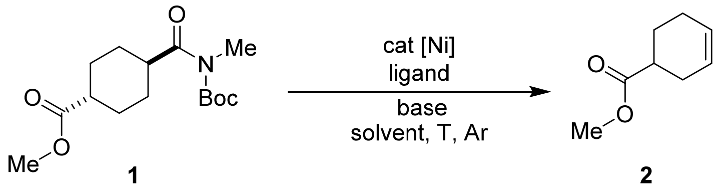

General procedure for Table 1. To a 25mL Schlenk flask equipped with a magnetic stir bar was charged with amide **1** (0.2 mmol). The tube was introduced in nitrogen-filled glovebox, and nickel salt (5-10 mol %), ligand (10-20 mol %), and base (0.5-3.0 equiv) were added. The tube with the mixture was taken out of the glovebox. Then solvent (1.5 mL) were added under argon. The formed mixture was stirred at 110-130 ^{o}C under Ar for 36 hours as monitored by TLC. The volatiles were

removed under reduced pressure, and the yield was determined by GC analysis with dodecane as an internal standard.

General Procedure for Ni-Catalyzed Retro-Hydroamidocarbonylation of Aliphatic Amides

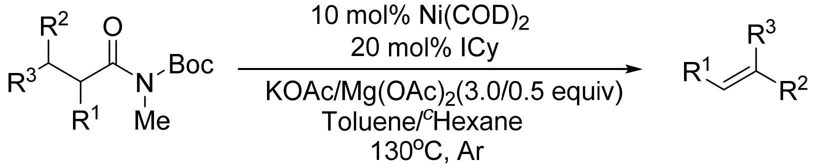

To a 25mL Schlenk flask equipped with a magnetic stir bar was charged with amide (0.2 mmol). The tube was introduced in nitrogen-filled glovebox, and $Ni(COD)_2$ (5.5 mg, 10 mol %), ICy (9.3 mg, 20 mol %), KOAc (58.9 mg, 3.0 equiv), $Mg(OAc)_2$ (14.2 mg, 0.5 equiv) were added. The tube with the mixture was taken out of the glovebox. Then cyclohexane (1.0 mL) and toluene (0.5 mL) were added under argon. The formed mixture was stirred at 130 ^{o}C under Ar for 36 hours as monitored by TLC and GC-MS. The solution was then cooled to room temperature. The crude product was further purified by column chromatography on silica gel.

Methyl cyclohex-3-ene-1-carboxylate (2)

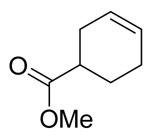

2

According to the general procedure, the reaction of 4-((*tert*-butoxycarbonyl)(methyl)carb-amoyl)cyclohexane-1-carboxylate **1** (59.9 mg, 0.2 mmol), $Ni(COD)_2$ (5.5 mg, 10 mol %), ICy (9.3 mg, 20 mol %), KOAc (58.9 mg, 3.0 equiv), $Mg(OAc)_2$ (14.2 mg, 0.5 equiv) cyclohexane (1.0 mL) and toluene (0.5 mL) under argon at 130 ^{o}C for 36 h under argon at 130 ^{o}C for 36 h after flash column chromatography on silica (*n*-pentane:AcOEt = 50:1) to afford 20.2 mg (72%) of **2** as a colourless oil: **^{1}H NMR (400 MHz, $CDCl_3$)** δ 5.75 – 5.61 (m, 2H), 3.69 (s, 3H), 2.62 – 2.51 (m, 1H), 2.30 – 2.22 (m, 2H), 2.14 – 2.05 (m, 2H), 2.05 – 1.96 (m, 1H), 1.77 – 1.62 (m, 1H); **^{13}C NMR (101 MHz, $CDCl_3$)** δ 176.29, 126.63, 125.16, 51.58, 39.21, 27.42, 25.06, 24.41; **ATR-FTIR (cm $^{-1}$):** 3133, 3132, 2361, 2360, 1699, 1653, 1559, 1558, 1541, 1457,

1401, 1398, 668, 458; **HRMS m/z (ESI)** calcd for $C_8H_{12}O_2$ $(M + H)^+$ 141.0910, found 141.0908. The spectral data for this compound matches that reported in the literature.[4]

Phenyl cyclohex-3-ene-1-carboxylate (4)

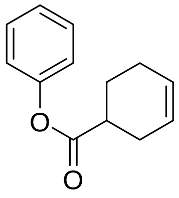

4

According to the general procedure, the reaction of 4-((*tert*-butoxycarbonyl)(methyl)carbamoyl)cyclohexane-1-carboxylate **3** (63.3 mg, 0.2 mmol), $Ni(COD)_2$ (5.5 mg, 10 mol %), ICy (9.3 mg, 20 mol %), KOAc (58.9 mg, 3.0 equiv), $Mg(OAc)_2$ (14.2 mg, 0.5 equiv) cyclohexane (1.0 mL) and toluene (0.5 mL) under argon at 130 ^oC for 36 h under argon at 130 ^oC for 36 h after flash column chromatography on silica (*n*-pentane:AcOEt = 20:1) to afford 22.1 mg (55%) of **4** as a colourless oil: **^{1}H NMR (400 MHz, $CDCl_3$)** δ 7.44 – 7.35 (m, 2H), 7.26 – 7.19 (m, 1H), 7.14 – 7.05 (m, 2H), 5.85 – 5.70 (m, 2H), 2.90 – 2.78 (m, 1H), 2.48 – 2.35 (m, 2H), 2.26 – 2.11 (m, 3H), 1.92 – 1.80 (m, 1H); **^{13}C NMR (101 MHz, $CDCl_3$)** δ 174.28, 150.82, 129.35, 126.73, 125.66, 124.97, 121.49, 39.40, 27.37, 25.02, 24.34; **ATR-FTIR (cm^{-1}):** 3405, 3127, 1757, 1683, 1618, 1401, 1194, 1133, 613; **HRMS m/z (ESI)** calcd for $C_{13}H_{14}O_2$ $(M + H)^+$ 203.1067, found 203.1069. The spectral data for this compound matches that reported in the literature.[5]

***N,N*-Dimethylcyclohex-3-ene-1-carboxamide (6)**

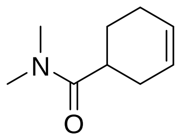

6

According to the general procedure, the reaction *t*-butyl (4-(dimethylcarbamoyl)cyclohexane-1-carbonyl)(methyl)carbamate **5** (62.4 mg, 0.2 mmol), $Ni(COD)_2$ (5.5 mg, 10 mol %), ICy (9.3 mg, 20 mol %), KOAc (58.9 mg, 3.0 equiv), $Mg(OAc)_2$ (14.2 mg, 0.5 equiv) cyclohexane (1.0 mL) and toluene (0.5 mL) under argon at 130 ^oC for 36 h after flash column chromatography on silica (*n*-pentane:AcOEt = 5:1) to afford afford 22.6 mg (74%) of **6** as a colourless oil: **^{1}H NMR (400 MHz, $CDCl_3$)** δ 5.75 – 5.63 (m, 2H), 3.05 (s, 3H), 2.94 (s, 3H), 2.78 – 2.71 (m, 1H), 2.39 – 2.25 (m, 1H), 2.19 – 2.00 (m, 3H), 1.81 – 1.64 (m, 2H); **^{13}C NMR (101 MHz, $CDCl_3$)** δ 175.84, 126.39, 125.86,

37.10, 36.58, 35.58, 27.85, 25.49, 24.95; **ATR-FTIR (cm $^{-1}$):** 3403, 3130, 1639, 1400, 1257, 1143, 728, 653, 618; **HRMS m/z (ESI)** calcd for $C_9H_{15}NO$ (M + Na)$^+$ 176.1046, found 176.1049.

((Cyclohex-3-en-1-yloxy)methyl)benzene (8)

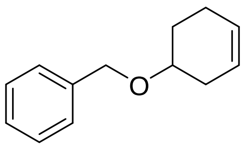

8

According to the general procedure, the reaction of *t*-Butyl (4-(benzyloxy)cyclohexane-1-carbonyl)(methyl)carbamate **7** (69.5 mg, 0.2 mmol), Ni(COD)$_2$ (5.5 mg, 10 mol %), ICy (9.3 mg, 20 mol %), KOAc (58.9 mg, 3.0 equiv), Mg(OAc)$_2$ (14.2 mg, 0.5 equiv), cyclohexane (1.0 mL) and toluene (0.5 mL) under argon at 130 ºC for 36 h after flash column chromatography on silica gel (*n*-pentane:AcOEt = 40:1) to afford 26.8 mg (71%) of **8** as a colourless oil: **^{1}H NMR (400 MHz, CDCl$_3$)** δ 7.40 – 7.31 (m, 4H), 7.30 – 7.26 (m, 1H), 5.74 – 5.52 (m, 2H), 4.70 – 4.51 (m, 2H), 3.77 – 3.57 (m, 1H), 2.50 – 2.33 (m, 1H), 2.28 – 2.15 (m, 1H), 2.15 – 1.95 (m, 3H), 1.75 – 1.61 (m, 1H); **^{13}C NMR (101 MHz, CDCl$_3$)** δ 139.02, 128.32, 127.55, 127.39, 126.86, 124.29, 73.80, 69.89, 31.70, 27.82, 24.04; **ATR-FTIR (cm $^{-1}$):** 3405, 3134, 1682, 1496, 1400, 1097, 734, 696, 658, 558; **HRMS m/z (ESI)** calcd for $C_{13}H_{16}O$ (M + H)$^+$ 189.1274, found 189.1277. The spectral data for this compound matches that reported in the literature.[6]

4-Pentylcyclohex-1-ene (10)

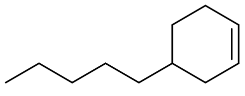

10

According to the general procedure, the reaction of *t*-Butyl methyl(4-pentylcyclohexane-1-carbonyl)carbamate **9** (62.2 mg, 0.2 mmol), Ni(COD)$_2$ (5.5 mg, 10 mol %), ICy (9.3 mg, 20 mol %), KOAc (58.9 mg, 3.0 equiv), Mg (OAc)$_2$(14.2 mg, 0.5 equiv), cyclohexane (1.0 mL) and toluene (0.5 mL) under argon at 130 ºC for 36 h after flash column chromatography on silica gel (*n*-pentane) to afford 19.2 mg (63%) of **10** as a colourless oil: **^{1}H NMR (400 MHz, CDCl$_3$)** δ 5.80 – 5.54 (m, 2H), 2.13 – 1.96 (m, 3H), 1.82 – 1.69 (m, 1H), 1.68 – 1.57 (m, 1H), 1.57 – 1.45 (m, 1H), 1.35 – 1.20 (m, 9H), 0.89 (t, *J* = 6.9 Hz, 3H); **^{13}C NMR (101 MHz, CDCl$_3$)** δ 127.03,

126.77, 36.71, 33.49, 32.15, 31.97, 29.00, 26.56, 25.33, 22.70, 14.11; **ATR-FTIR (cm $^{-1}$):** 3405, 3134, 1682, 1400, 560; **HRMS m/z (ESI)** calcd for $C_{11}H_{20}$ (M + Na)$^+$ 175.14570, found 175.1460.

(3,6-Dihydropyridin-1(2H)-yl)(phenyl)methanone (12)

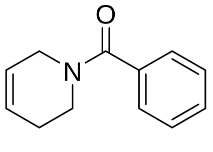

12

According to the general procedure, the reaction of *t*-Butyl (1-benzoylpiperidine-4-carbonyl)(methyl)carbamate **11** (69.3 mg, 0.2 mmol), Ni(COD)$_2$ (5.5 mg, 10 mol %), ICy (9.3 mg, 20 mol %), KOAc (58.9 mg, 3.0 equiv), Mg(OAc)$_2$ (14.2 mg, 0.5 equiv), cyclohexane (1.0 mL) and toluene (0.5 mL) under argon at 130 ºC for 36 h after flash column chromatography on silica gel (*n*-pentane:AcOEt = 2:1) to afford 26.8 mg (71%) of **12** as a colourless oil: **^{1}H NMR (400 MHz, CDCl$_3$)**, rotamers, δ 7.40 – 7.32 (m, 5H), 5.87 – 5.80 (m, 1H), 5.78 – 5.66 (m, 0.6H), 5.58 – 5.41 (m, 0.4H), 4.23 – 4.10 (m, 1H), 3.91 – 3.77 (m, 1H), 3.50 – 3.38 (m, 1H), 3.31 (s, 1H), 2.3 – 2.0 (m, 2H); **^{13}C NMR (101 MHz, CDCl$_3$)**, rotamers, δ 170.70, 170.22, 149.21, 136.12, 129.47, 128.31, 126.83, 126.62, 126.19, 124.78, 124.33, 123.63, 47.46, 44.34, 42.28, 39.15, 29.25, 25.72, 24.73; **ATR-FTIR (cm $^{-1}$):** 3403, 3134, 2927, 2841, 1687, 1661, 1577, 1400, 1344, 1288, 1261, 1154, 1049, 1025, 1001, 972, 858, 789, 758, 729, 706, 657, 632, 566, 482, 424; **HRMS m/z (ESI)** calcd for $C_{12}H_{13}NO$ (M + H)$^+$ 188.1070, found 188.1070. The spectral data for this compound matches that reported in the literature.[7]

Benzyl 3,6-dihydropyridine-1(2*H*)-carboxylate (14) & Benzyl 3,4-dihydropyridine-1(2*H*)-carboxylate (14')

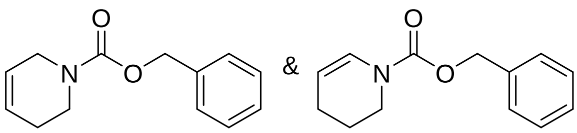

14 **14'**

According to the general procedure, the reaction of benzyl 3-((*tert*-butoxycarbonyl)(methyl)carbamoyl)piperidine-1-carboxylate **13** (75.3 mg, 0.2 mmol), Ni(COD)$_2$ (5.5 mg, 10 mol %), ICy (9.3 mg, 20 mol %), KOAc (58.9 mg, 3.0 equiv), Mg(OAc)$_2$ (14.2 mg, 0.5 equiv), cyclohexane (1.0 mL) and

toluene (0.5 mL) under argon at 130 ºC for 36 h (the ratio of **14** and **14'** (2:1) was identified by crude ^{1}H NMR) after flash column chromatography on silica gel (*n*-pentane:AcOEt = 5:1) to afford 22.1 mg (51%) of **14** and 10.5 mg (24%) of **14'**. **14**: **^{1}H NMR (400 MHz, $CDCl_3$)**, rotamers, δ 7.41 – 7.28 (m, 5H), 5.82 (br s, 1H), 5.64 (br s, 1H), 5.15 (s, 2H), 4.04 – 3.89 (m, 2H), 3.57 (t, *J* = 5.7 Hz, 2H), 2.24 – 2.04 (m, 2H); **^{13}C NMR (101 MHz, $CDCl_3$)** , rotamers, δ 155.56, 136.82, 128.43, 127.92, 127.84, 125.39, 125.07, 124.40, 123.83, 66.98, 43.43, 40.60, 40.23, 25.33, 24.85; **ATR-FTIR (cm $^{-1}$)**: 3404, 3133, 3035, 2842, 1702, 1498, 1401, 1360, 1337, 1282, 1237, 1217, 1198, 1147, 1106, 1050, 1026, 974, 912, 765, 698, 658, 602, 557; **HRMS m/z (ESI)** calcd for $C_{13}H_{15}NO_2$ $(M + Na)^+$ 240.0995, found 240.0998; **14'**: **^{1}H NMR (400 MHz, $CDCl_3$)**, rotamers, δ 7.49 – 7.29 (m, 5H), 6.89 (d, *J* = 8.3 Hz, 0.4H), 6.80 (d, *J* = 8.4 Hz, 0.6H), 5.18 (s, 2H), 5.03 – 4.92 (m, 0.4H), 4.91 – 4.81 (m, 0.6H), 3.71 – 3.58 (m, 2H), 2.16 – 1.95 (m, 2H), 1.89 – 1.76 (m, 2H); **^{13}C NMR (101 MHz, $CDCl_3$)** , rotamers, δ 153.52, 153.10, 136.35, 128.47, 128.07, 127.96, 125.31, 124.85, 106.72, 106.38, 67.38, 67.29, 42.35, 42.16, 21.58, 21.39, 21.20; **ATR-FTIR (cm $^{-1}$):** 3405, 3134, 1703, 1401, 1255, 1226, 1107, 1053, 697, 554; **HRMS m/z (ESI)** calcd for $C_{13}H_{15}NO_2$ $(M + Na)^+$ 240.0995, found 240.0996. The spectral data for this compound matches that reported in the literature.[8-9]

(((3,3-Dimethylpent-4-en-1-yl)oxy)methyl)benzene (16)

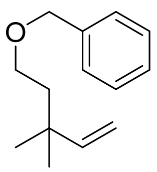

16

According to the general procedure, the reaction *tert*-Butyl (6-(benzyloxy)-4,4-dimethylhexanoyl)(methyl)carbamate **15** (72.7 mg, 0.2 mmol), $Ni(COD)_2$ (5.5 mg, 10 mol %), ICy (9.3 mg, 20 mol %), KOAc (58.9 mg, 3.0 equiv), $Mg(OAc)_2$ (14.2 mg, 0.5 equiv), cyclohexane (1.0 mL) and toluene (0.5 mL) under argon at 130 ºC for 36 h after flash column chromatography on silica gel (*n*-pentane) to afford 25.7 mg (63%) of **16** as a colourless oil: **^{1}H NMR (400 MHz, $CDCl_3$)** δ 7.37 – 7.32 (m, 4H), 7.31 – 7.26 (m, 1H), 5.80 (dd, *J* = 17.8, 10.4 Hz, 1H), 4.93 (dd, *J* = 5.2, 1.3 Hz, 1H), 4.90 (t, *J* = 1.6 Hz, 1H), 4.48 (s, 2H), 3.52 – 3.42 (m, 2H), 1.71 – 1.65 (m, 2H), 1.03 (s, 6H); **^{13}C NMR (101 MHz, $CDCl_3$)** δ 147.94, 138.59, 128.31, 127.57, 127.43, 110.46, 72.92,

67.69, 41.62, 35.63, 27.09; **ATR-FTIR (cm $^{-1}$):** 3405, 3134, 1682, 1511, 1401, 559; **HRMS m/z (ESI)** calcd for $C_{14}H_{20}O$ $(M + Na)^+$ 227.1406, found 227.1409.

5-((3,3-Dimethylpent-4-en-1-yl)oxy)pentanenitrile (18)

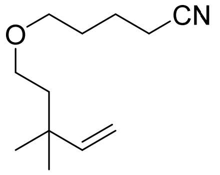

18

According to the general procedure, the reaction *tert*-butyl (6-(4-cyanobutoxy)-4,4-dimethylhexanoyl)(methyl)carbamate **17** (70.9 mg, 0.2 mmol), $Ni(COD)_2$ (5.5 mg, 10 mol %), ICy (9.3 mg, 20 mol %), KOAc (58.9 mg, 3.0 equiv), $Mg(OAc)_2$ (14.2 mg, 0.5 equiv), cyclohexane (1.0 mL) and toluene (0.5 mL) under argon at 130 ºC for 36 h after flash column chromatography on silica gel (n-Pentane:AcOEt = 10:1) to afford 18.0 mg (46%) of **18** as a colourless oil: **^{1}H NMR (400 MHz, $CDCl_3$)** δ 5.78 (dd, *J* = 17.8, 10.4 Hz, 1H), 4.93 (d, *J* = 1.5 Hz, 1H), 4.89 (dd, *J* = 4.4, 1.3 Hz, 1H), 3.42 – 3.35 (m, 4H), 2.38 (t, *J* = 6.9 Hz, 2H), 1.77 – 1.68 (m, 4H), 1.59 (t, *J* = 6.1 Hz, 2H), 1.01 (s, 6H); **^{13}C NMR (101 MHz, $CDCl_3$)** δ 147.87, 119.72, 110.48, 69.54, 68.14, 41.59, 35.56, 28.64, 27.06, 22.62, 16.99; **ATR-FTIR (cm $^{-1}$):** 3442, 3083, 2960, 2929, 2889, 2246, 1720, 1640, 1458, 1427, 1364, 1115, 1003, 912, 688; **HRMS m/z (ESI)** calcd for $C_{12}H_{21}NO$ $(M + Na)^+$ 218.1515, found 218.1513.

5-((3,3-Dimethylpent-4-en-1-yl)oxy)pentanenitrile (20)

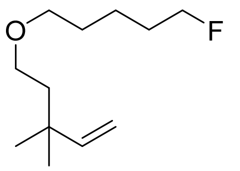

20

According to the general procedure, the reaction *t*-butyl (6-((5-fluoropentyl)oxy)-4,4-dimethylhexanoyl)(methyl)carbamat e **19** (72.3 mg, 0.2 mmol), $Ni(COD)_2$ (5.5 mg, 10 mol %), ICy (9.3 mg, 20 mol %), KOAc (58.9 mg, 3.0 equiv), $Mg(OAc)_2$ (14.2 mg, 0.5 equiv), cyclohexane (1.0 mL) and toluene (0.5 mL) under argon at 130 ºC for 36 h after flash column chromatography on silica gel (*n*-pentane) to afford 27.5 mg (68%) of **20** as a colourless oil: **^{1}H NMR (400 MHz, $CDCl_3$)** δ 5.78 (dd, *J* = 17.8, 10.4 Hz, 1H), 4.92 (dd, *J* = 4.8, 1.4 Hz, 1H), 4.88 (dd, *J* = 2.4, 1.4 Hz, 1H), 4.49 (t, *J* = 6.2 Hz, 1H), 4.37 (t, *J* = 6.1 Hz, 1H), 3.39 – 3.35 (m, 4H), 1.75 – 1.65 (m, 2H), 1.63 – 1.57 (m, 4H), 1.48 – 1.42 (m, 2H), 1.00 (s, 6H); **^{13}C NMR (101 MHz, $CDCl_3$)** δ 147.94, 110.39, 83.99 (d, *J* = 165.6 Hz), 70.59, 67.99, 41.60, 35.54, 30.20 (d, *J* = 19.2

Hz), 29.37, 27.04, 21.94 (d, J = 6.1 Hz); **^{19}F NMR (377 MHz, $CDCl_3$)** δ -218.38; **ATR-FTIR (cm $^{-1}$):** 3443, 2961, 2866, 1640, 1460, 1363, 1115, 1046, 1002, 911, 736, 689; **HRMS m/z (ESI)** calcd for $C_{12}H_{23}FO$ $(M + H)^+$ 203.1806, found 203.1802.

(*E*)-hexadec-2-ene (22)

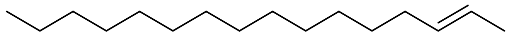

22

According to the general procedure, the reaction of tert-butyl methyl(2-methyl hexadecanoyl)carbamate **21** (76.7 mg, 0.2 mmol), Ni(COD)$_2$ (5.5 mg, 10 mol %), ICy (9.3 mg, 20 mol %), KOAc (58.9 mg, 3.0 equiv), Mg(OAc)$_2$ (14.2 mg, 0.5 equiv) cyclohexane (1.0 mL) and toluene (0.5 mL) under argon at 130 ºC for 36 h after flash column chromatography on silica gel (*n*-pentane) to afford 27.9 mg (62%) of **22** as a colourless oil (2-alkene selectivity based on ^{1}H NMR: 77%): **^{1}H NMR (400 MHz, $CDCl_3$)** δ 5.49 – 5.33 (m, 2H), 2.02 – 1.92 (m, 2H), 1.68 – 1.55 (m, 3H), 1.32 – 1.20 (m, 22H), 0.88 (d, J = 8.0 Hz, 3H); **^{13}C NMR (101 MHz, $CDCl_3$)** δ 131.71, 124.49, 32.62, 31.94, 29.70, 29.67, 29.65, 29.56, 29.38, 22.70, 17.90, 14.11; **ATR-FTIR (cm $^{-1}$):** 3405, 3134, 2958, 2925, 2854, 1681, 1401, 964, 909, 721, 558; **HRMS m/z (ESI)** calcd for $C_{16}H_{32}$ $(M + H)^+$ 225.2577, found 225.2580. The spectral data for this compound matches that reported in the literature.[10]

1-Methoxy-4-vinylbenzene (24)

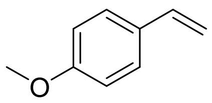

24

According to the general procedure, the reaction tert-butyl (3-(4-methoxyphenyl)propanoyl)(methyl)carbamate **23** (58.7 mg, 0.2 mmol), Ni(COD)$_2$ (5.5 mg, 10 mol %), ICy (9.3 mg, 20 mol %), KOAc (58.9 mg, 3.0 equiv), Mg(OAc)$_2$(14.2 mg, 0.5 equiv), cyclohexane (1.0 mL) and toluene (0.5 mL) were added under argon at 130 ºC for 36 h after flash column chromatography on silica gel (n-Pentane:AcOEt = 100:1) to afford 19.1 mg (71%) of **24** as a colourless oil: **^{1}H NMR (400 MHz, $CDCl_3$)** δ 7.35 (d, J = 8.7 Hz, 2H), 6.87 (d, J = 8.7 Hz, 2H), 6.67 (dd, J = 17.6, 10.9 Hz, 1H), 5.61 (d, J = 17.6 Hz, 1H), 5.13 (d, J = 10.9 Hz, 1H), 3.82 (s, 3H); **^{13}C NMR (101 MHz, $CDCl_3$)** δ 159.35, 136.20, 130.43, 127.36, 113.89, 111.56, 55.28; **ATR-FTIR (cm $^{-1}$):** 3406, 3135, 1682, 1400, 560;

EI-MS (m/z, relative intensity): 134 (M^+, 30), 133 (84), 119 (100), 90 (75), 65 (56). The spectral data for this compound matches that reported in the literature.[11]

5-(4-Vinylphenoxy)pentanenitrile (26)

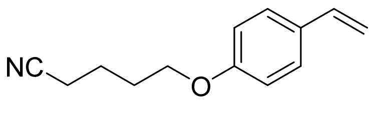

26

According to the general procedure, the reaction tert-butyl (3-(4-(4-cyanobutoxy)phenyl)propanoyl)(methyl)carbamate **25** (72.1 mg, 0.2 mmol), $Ni(COD)_2$ (5.5 mg, 10 mol %), ICy (9.3 mg, 20 mol %), KOAc (58.9 mg, 3.0 equiv), $Mg(OAc)_2$(14.2 mg, 0.5 equiv), cyclohexane (1.0 mL) and toluene (0.5 mL) were added under argon at 130 ºC for 36 h after flash column chromatography on silica gel (n-Pentane:AcOEt = 10:1) to afford 26.6 mg (66%) of **26** as a colourless oil: **^{1}H NMR (400 MHz, $CDCl_3$)** δ 7.34 (d, J = 8.2 Hz, 2H), 6.84 (d, J = 8.2 Hz, 2H), 6.66 (dd, J = 17.6, 10.9 Hz, 1H), 5.61 (d, J = 17.6 Hz, 1H), 5.13 (d, J = 10.9 Hz, 1H), 4.01 (t, J = 5.5 Hz, 2H), 2.45 (t, J = 6.7 Hz, 2H), 1.98 – 1.86 (m, 4H); **^{13}C NMR (101 MHz, $CDCl_3$)** δ158.41, 136.10, 130.62, 127.39, 119.46, 114.37, 111.71, 66.62, 28.13, 22.41, 16.97; **ATR-FTIR (cm $^{-1}$):** 3396, 2923, 2245, 1628, 1607, 1574, 1510, 1472, 1425, 1289, 1245, 1175, 1116, 1060, 991, 956, 905, 836, 739, 498; **HRMS m/z (ESI)** calcd for $C_{13}H_{15}NO$ $(M + H)^+$ 202.1226, found 202.1222.

.

2-(4-Vinylbenzyl)cyclopentan-1-one (28)

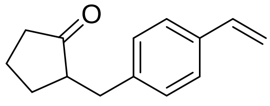

28

According to the general procedure, the reaction of *tert*-butyl methyl(2-(4-((2-oxocyclopentyl)methyl)phenyl)propanoyl)carbamate **27** (71.9 mg, 0.2 mmol), $Ni(COD)_2$ (5.5 mg, 10 mol %), ICy (9.3 mg, 20 mol %), KOAc (58.9 mg, 3.0 equiv), $Mg(OAc)_2$ (14.2 mg, 0.5 equiv), cyclohexane (1.0 mL) and toluene (0.5 mL) under argon at 130 ºC for 36 h after flash column chromatography on silica gel (*n*-pentane) to afford 25.2 mg (63%) of **28** as a colourless oil: **^{1}H NMR (400 MHz, $CDCl_3$)** δ 7.33 (d, J = 8.1 Hz, 2H), 7.12 (d, J = 8.0 Hz, 2H), 6.69 (dd, J = 17.6, 10.9 Hz, 1H), 5.71 (dd, J = 17.6, 0.9 Hz, 1H), 5.20 (dd, J = 10.9, 0.8 Hz, 1H), 3.12 (dd, J = 13.9, 4.2 Hz, 1H), 2.54 (dd, J = 13.9, 9.4 Hz, 1H), 2.41 – 2.27 (m, 2H), 2.17 – 2.03 (m, 2H), 2.02 – 1.89 (m, 1H), 1.81 – 1.70 (m,

1H), 1.61 – 1.47 (m, 1H); **^{13}C NMR (101 MHz, $CDCl_3$)** δ 220.06, 139.64, 136.48, 135.54, 129.03, 126.22, 113.18, 50.88, 38.14, 35.23, 29.03, 20.49; **ATR-FTIR (cm $^{-1}$):** 3405, 3134, 1738, 1682, 1512, 1401, 1151, 557; **HRMS m/z (ESI)** calcd for $C_{14}H_{16}O$ $(M + H)^+$ 201.1274, found 201.1278.

(*E*)-5-(Prop-1-en-1-yl)benzo[*d*][1,3]dioxole (30)

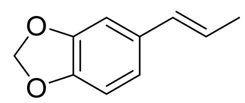

30

According to the general procedure, the reaction of *tert*-butyl (3-(benzo[d][1,3]dioxol-5-yl)-2-methylpropanoyl)(methyl)carbamate **29** (64.3 mg, 0.2 mmol), Ni(COD)$_2$ (5.5 mg, 10 mol %), ICy (9.3 mg, 20 mol %), KOAc (58.9 mg, 3.0 equiv), Mg(OAc)$_2$ (14.2 mg, 0.5 equiv), cyclohexane (1.0 mL) and toluene (0.5 mL) under argon at 130 ºC for 36 h after flash column chromatography on silica gel (*n*-pentane:AcOEt = 50:1) to afford 20.8 mg (64%) of **30** as a colourless oil: **^{1}H NMR (400 MHz, $CDCl_3$)** δ 6.88 (s, 1H), 6.79 – 6.71 (m, 2H), 6.31 (dd, J = 15.7, 1.5 Hz, 1H), 6.11 – 6.01 (m, 1H), 5.93 (s, 2H), 1.85 (dd, J = 6.6, 1.6 Hz, 3H); **^{13}C NMR (101 MHz, $CDCl_3$)** δ 147.87, 146.45, 132.47, 130.50, 123.92, 120.03, 108.18, 105.29, 100.87, 18.32; **ATR-FTIR (cm $^{-1}$):** 3405, 3134, 1682, 1400, 560; **HRMS m/z (ESI)** calcd for $C_{10}H_{10}O_2$ $(M + Na)^+$ 185.0573, found 185.0577. The spectral data for this compound matches that reported in the literature.[12]

(*E*)-1,2-Diphenylethene (32)

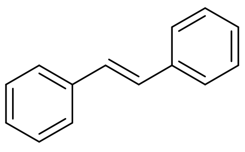

32

According to the general procedure, the reaction of *tert*-butyl (2,3-diphenylpropanoyl)(methyl)carbamate **31** (67.9 mg, 0.2 mmol), Ni(COD)$_2$ (5.5 mg, 10 mol %), ICy (9.3 mg, 20 mol %), KOAc (58.9 mg, 3.0 equiv), Mg(OAc)$_2$ (14.2 mg, 0.5 equiv), cyclohexane (1.0 mL) and toluene (0.5 mL) under argon at 130 ºC under Ar for 36 h after flash column chromatography on silica gel (*n*-pentane) to afford 29.8 mg (83%) of **32** as a white solid: **^{1}H NMR (400 MHz, $CDCl_3$)** δ 7.59 –7.53 (m, 4H), 7.45 – 7.36 (m, 4H), 7.34 – 7.27 (m, 2H), 7.15 (s, 2H); **^{13}C NMR (101 MHz, $CDCl_3$)** δ 137.30, 128.67, 128.65, 127.59, 126.49; **ATR-FTIR (cm $^{-1}$):** 3405, 3134, 3021, 1682, 1495,

1452, 1400, 1072, 984, 963, 909, 764, 693, 540, 527; **EI-MS (m/z, relative intensity):** 180 (M^+, 94), 179 (100), 165 (85), 152 (34), 115 (9), 102 (20), 89 (48), 76 (32). The spectral data for this compound matches that reported in the literature.[13]

(2-Methylprop-1-en-1-yl)benzene (34)

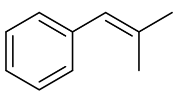

34

According to the general procedure, the reaction of *tert*-butyl methyl(3-methyl-2-phenylbutanoyl)carbamate **33** (58.3 mg, 0.2 mmol), $Ni(COD)_2$ (5.5 mg, 10 mol %), ICy (9.3 mg, 20 mol %), KOAc (58.9 mg, 3.0 equiv), $Mg(OAc)_2$ (14.2 mg, 0.5 equiv), cyclohexane (1.0 mL) and toluene (0.5 mL) under argon at 130 ºC for 36 h after flash column chromatography on silica gel (*n*-pentane) to afford 20.4 mg (77%) of **34** as a colourless oil: **^{1}H NMR (400 MHz, $CDCl_3$)** δ 7.35 – 7.28 (m, 2H), 7.25 – 7.13 (m, 3H), 6.27 (s, 1H), 1.91 (d, *J* = 1.3 Hz, 3H), 1.86 (d, *J* = 1.2 Hz, 3H); **^{13}C NMR (101 MHz, $CDCl_3$)** δ 138.66, 135.47, 128.70, 127.99, 125.74, 125.07, 26.86, 19.36; **ATR-FTIR (cm $^{-1}$):** 3405, 3134, 1681, 1400, 558; **HRMS m/z (ESI)** calcd for $C_{10}H_{12}$ $(M + Na)^+$ 155.0831, found 155.0833. The spectral data for this compound matches that reported in the literature.[14]

(Cyclopentylidenemethyl)benzene (36)

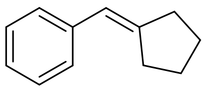

36

According to the general procedure, the reaction of *tert*-butyl (2-cyclopentyl-2-phenylacetyl)(methyl)carbamate **35** (63.5 mg, 0.2 mmol), $Ni(COD)_2$ (5.5 mg, 10 mol %), ICy (9.3 mg, 20 mol %), KOAc (58.9 mg, 3.0 equiv), $Mg(OAc)_2$ (14.2 mg, 0.5 equiv), cyclohexane (1.0 mL) and toluene (0.5 mL) under argon at 130 ºC for 36 h after flash column chromatography on silica gel (*n*-pentane) to afford 27.2 mg (82%) of **36** as a colourless oil: **^{1}H NMR (400 MHz, $CDCl_3$)** δ 7.37 – 7.28 (m, 4H), 7.21 – 7.12 (m, 1H), 6.40 – 6.34 (m, 1H), 2.59 – 2.52 (m, 2H), 2.53 – 2.44 (m, 2H), 1.85 – 1.73 (m, 2H), 1.74 – 1.61 (m, 2H); **^{13}C NMR (101 MHz, $CDCl_3$)** δ 147.20, 138.88, 128.16, 127.92, 125.58, 120.76, 35.94, 31.16, 27.20, 25.63; **ATR-FTIR (cm $^{-1}$):** 3405, 3134, 2957, 1682, 1495, 1401, 1075, 910, 861, 738, 694, 513; **HRMS m/z (ESI)** calcd for

$C_{12}H_{13}$ (M + H)$^+$ 159.1168, found 159.1169. The spectral data for this compound matches that reported in the literature.[15]

Prop-1-en-2-ylbenzene (38)

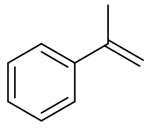

38

According to the general procedure, the reaction of *tert*-butyl methyl(2-methyl-2-phenylpropanoyl)carbamate **37** (55.5 mg, 0.2 mmol), Ni(COD)$_2$ (5.5 mg, 10 mol %), ICy (9.3 mg, 20 mol %), KOAc (58.9 mg, 3.0 equiv), Mg(OAc)$_2$ (14.2 mg, 0.5 equiv), cyclohexane (1.0 mL) and toluene (0.5 mL) under argon at 130 ºC for 36 h after flash column chromatography on silica gel (*n*-pentane) to afford 15.1 mg (64%) of **38** as a colourless oil: **^{1}H NMR (400 MHz, CDCl$_3$)** δ 7.55 (dd, *J* = 8.0, 0.8 Hz, 2H), 7.40 (dd, *J* = 8.0, 6.7 Hz, 2H), 7.37 – 7.30 (m, 1H), 5.45 (d, *J* = 0.7 Hz, 1H), 5.25 – 5.10 (m, 1H), 2.23 (s, 3H); **^{13}C NMR (101 MHz, CDCl$_3$)** δ 143.27, 141.22, 128.19, 127.37, 125.47, 112.37, 21.78; **ATR-FTIR (cm $^{-1}$):** 3406, 3136, 2949, 1679, 1496, 1405, 1076, 912, 863, 740, 696, 516. The spectral data for this compound matches that reported in the literature.[16]

Cyclopent-1-en-1-ylbenzene (40)

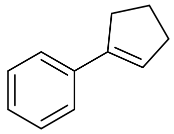

40

According to the general procedure, the reaction of *tert*-butyl methyl(1-phenylcyclopentane-1-carbonyl)carbamate **39** (60.7 mg, 0.2 mmol), Ni(COD)$_2$ (5.5 mg, 10 mol %), ICy (9.3 mg, 20 mol %), KOAc (58.9 mg, 3.0 equiv), Mg(OAc)$_2$ (14.2 mg, 0.5 equiv), cyclohexane (1.0 mL) and toluene (0.5 mL) under argon at 130 ºC for 36 h after flash column chromatography on silica gel (*n*-pentane) to afford 19.1 mg (66%) of **40** as a colourless oil: **^{1}H NMR (400 MHz, CDCl$_3$)** δ 7.49 – 7.42 (m, 2H), 7.36 – 7.29 (m, 2H), 7.25 – 7.19 (m, 1H), 6.23 – 6.16 (m, 1H), 2.78 – 2.68 (m, 2H), 2.59 – 2.49 (m, 2H), 2.08 – 1.98 (m, 2H); **^{13}C NMR (101 MHz, CDCl$_3$)** δ 142.41, 136.80, 128.24, 126.79, 126.08, 125.53, 33.33, 33.16, 23.34; **ATR-FTIR (cm $^{-1}$):** 3405, 3134, 1681, 1401; **HRMS m/z (ESI)** calcd for $C_{11}H_{12}$ (M + H)$^+$ 145.1012, found 145.1014. The spectral data for this compound matches that reported in the literature.[17]

1-Methoxy-4-(prop-1-en-1-yl)benzene (42)

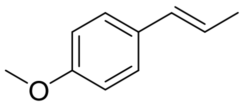

42

According to the general procedure, the reaction of *tert*-butyl (4-(4-methoxyphenyl)butanoyl)(methyl)carbamate **41** (61.5 mg, 0.2 mmol), Ni(COD)$_2$ (5.5 mg, 10 mol %), ICy (9.3 mg, 20 mol %), KOAc (58.9 mg, 3.0 equiv), Mg(OAc)$_2$ (14.2 mg, 0.5 equiv), cyclohexane (1.0 mL) and toluene (0.5 mL) under argon at 130 ºC for 36 h after flash column chromatography on silica gel (*n*-pentane:AcOEt = 100:1) to afford 21.6 mg (73%) of **42** as a colourless oil: **^{1}H NMR (400 MHz, CDCl$_3$)** δ 7.36 – 7.21 (m, 2H), 6.92 – 6.78 (m, 2H), 6.35 (dd, *J* = 15.7, 1.5 Hz, 1H), 6.15 – 6.02 (m, 1H), 3.80 (s, 3H), 1.86 (dd, *J* = 6.6, 1.6 Hz, 3H); **^{13}C NMR (101 MHz, CDCl$_3$)** δ 158.52, 130.77, 130.28, 126.83, 123.47, 113.86, 55.24, 18.40; **ATR-FTIR (cm $^{-1}$):** 3405, 3134, 1682, 1503, 1490, 1400, 1248, 1192, 1105, 1041, 961, 938, 860, 818, 780, 559; **EI-MS (m/z, relative intensity):** 148 (M$^+$, 100), 117 (60), 105 (50), 77 (40). The spectral data for this compound matches that reported in the literature.[18]

(*E*)-But-2-en-2-ylbenzene (44)

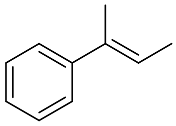

44

According to the general procedure, the reaction of *tert*-butyl methyl(4-phenylpentanoyl)carbamate **43** (58.3 mg, 0.2 mmol), Ni(COD)$_2$ (5.5 mg, 10 mol %), ICy (9.3 mg, 20 mol %), KOAc (58.9 mg, 3.0 equiv), Mg(OAc)$_2$ (14.2 mg, 0.5 equiv), cyclohexane (1.0 mL) and toluene (0.5 mL) under argon at 130 ºC for 36 h after flash column chromatography on silica gel (*n*-pentane) to afford 17.5 mg (66%) of **44** as a colourless oil: **^{1}H NMR (400 MHz, CDCl$_3$)** δ 7.41 – 7.33 (m, 2H), 7.34 – 7.27 (m, 2H), 7.24 – 7.17 (m, 1H), 5.92 – 5.81 (m, 1H), 2.08 – 1.99 (m, 3H), 1.80 (dd, *J* = 6.9, 1.0 Hz, 3H); **^{13}C NMR (101 MHz, CDCl$_3$)** δ 144.03, 135.49, 128.12, 126.37, 125.51, 122.45, 15.46, 14.31; **ATR-FTIR (cm $^{-1}$):** 3404, 3133, 1681, 1401, 748, 690; **HRMS m/z (ESI)** calcd for $C_{10}H_{12}$ (M + H)$^+$ 133.1012, found 133.1008. The spectral data for this compound matches that reported in the literature.[19]

(*E*)-Tridec-2-ene (46)

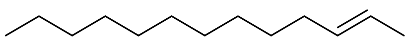

46

According to the general procedure, the reaction of *tert*-butyl methyl(tetradecanoyl)carba mate **45** (68.3 mg, 0.2 mmol), Ni(COD)$_2$ (5.5 mg, 10 mol %), ICy (9.3 mg, 20 mol %), KOAc (58.9 mg, 3.0 equiv), Mg(OAc)$_2$ (14.2 mg, 0.5 equiv), cyclohexane (1.0 mL) and toluene (0.5 mL) under argon at 130 ºC for 36 h after flash column chromatography on silica gel (*n*-pentane) to afford 24.2 mg (66%) of **46** as a colourless oil (2-alkene selectivity based on ^{1}H NMR: 80%): **^{1}H NMR (400 MHz, CDCl$_3$)** δ 5.54 – 5.32 (m, 2H), 2.01 – 1.90 (m, 2H), 1.70 – 1.54 (m, 3H), 1.29 – 1.20 (m, 16H), 0.88 (t, J = 8.0 Hz, 3H); **^{13}C NMR (101 MHz, CDCl$_3$)** δ 131.70, 124.50, 32.62, 31.92, 29.64, 29.54, 29.35, 29.21, 22.69, 17.92, 14.11; **ATR-FTIR (cm $^{-1}$):** 3405, 3134, 1682, 1401, 556; **EI-MS (m/z, relative intensity):** 182 (M$^+$, 34), 154 (6), 125 (12), 111 (29), 97 (72), 70 (78), 69 (88), 55 (100). The spectral data for this compound matches that reported in the literature.[20]

(*E*)-Pentadec-2-ene (48)

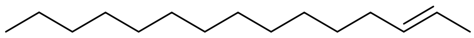

48

According to the general procedure, the reaction of *tert*-butyl methyl(palmitoyl)car bamate **47** (73.9 mg, 0.2 mmol), Ni(COD)$_2$ (5.5 mg, 10 mol %), ICy (9.3 mg, 20 mol %), KOAc (58.9 mg, 3.0 equiv), Mg(OAc)$_2$ (14.2 mg, 0.5 equiv), cyclohexane (1.0 mL) under argon at 130 ºC for 36 h after flash column chromatography on silica gel (*n*-pentane) to afford 25.6 mg (61%) of **48** as a colourless oil (2-alkene selectivity based on ^{1}H NMR: 82%): **^{1}H NMR (400 MHz, CDCl$_3$)** δ 5.52 – 5.28 (m, 2H), 2.01 – 1.90 (m, 2H), 1.68 – 1.56 (m, 3H), 1.28 – 1.23 (m, 20H), 0.88 (7, *J* = 8.0 Hz, 3H); **^{13}C NMR (101 MHz, CDCl$_3$)** δ 131.71, 124.50, 32.61, 31.93, 29.69, 29.66, 29.55, 29.37, 29.22, 22.69, 17.90, 14.10; **ATR-FTIR (cm $^{-1}$):** 3404, 3133, 2960, 2925, 2854, 1682, 1400, 964, 559; **EI-MS (m/z, relative intensity):** 210 (M$^+$, 10), 182 (4), 139 (6), 125 (18), 111 (49), 83 (95), 69 (100). The spectral data for this compound matches that reported in the literature.[21]

(*2E,12Z*)-henicosa-2,12-diene (50)

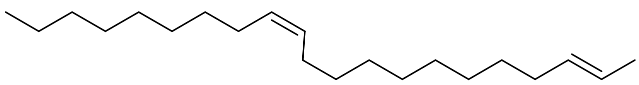

50

According to the general procedure, the reaction of tert-butyl (Z)-docos-13-enoyl (methyl)carbamate **49** (90.3 mg, 0.2 mmol), $Ni(COD)_2$ (5.5 mg, 10 mol %), ICy (9.3 mg, 20 mol %), KOAc (58.9 mg, 3.0 equiv), $Mg(OAc)_2$ (14.2 mg, 0.5 equiv) , cyclohexane (1.0 mL) and toluene (0.5 mL) under argon at 130 ºC for 36 h after flash column chromatography on silica gel (*n*-pentane) to afford 26.7 mg (46%) of **50** as a colourless oil (2-alkene selectivity based on ^{1}H NMR: 77%): **^{1}H NMR (400 MHz, $CDCl_3$)** δ 5.51 – 5.29 (m, 4H), 2.04 – 1.94 (m, 6H), 1.68 – 1.56 (m, 2H), 1.34 – 1.23 (m, 25H), 0.88 (t, *J* = 8.0 Hz, 3H); **^{13}C NMR (101 MHz, $CDCl_3$)** δ 131.69, 129.91, 129.89, 124.51, 32.60, 31.91, 29.78, 29.66, 29.62, 29.53, 29.32, 29.19, 29.15, 27.21, 22.68, 17.89, 14.09; **ATR-FTIR (cm $^{-1}$):** 3405, 3133, 2925, 2854, 1682, 1401, 965, 722, 558; **HRMS m/z (ESI)** calcd for $C_{21}H_{40}$ $(M + H)^+$ 293.3203, found 293.3206.

(*E*)-2-Octyl-3-(undec-9-en-1-yl)oxirane (52)

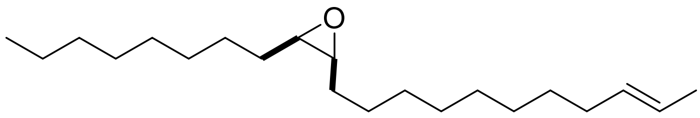

52

According to the general procedure, the reaction of *tert*-butyl methyl(12-(3-octyloxiran-2-yl)dodecanoyl)carbamate **51** (93.5 mg, 0.2 mmol), $Ni(COD)_2$ (5.5 mg, 10 mol %), ICy (9.3 mg, 20 mol %), KOAc (58.9 mg, 3.0 equiv), $Mg(OAc)_2$ (14.2 mg, 0.5 equiv) , cyclohexane (1.0 mL) and toluene (0.5 mL) under argon at 130 ºC for 36 h after flash column chromatography on silica gel (*n*-pentane) to afford 26.5 mg (43%) of **52** as a colourless oil (2-alkene selectivity based on ^{1}H NMR: 76%): **^{1}H NMR (400 MHz, $CDCl_3$)** δ 5.48 – 5.32 (m, 2H), 2.95 – 2.85 (m, 2H), 2.08 – 1.92 (m, 3H), 1.65 – 1.62 (m, 2H), 1.52 – 1.46 (m, 5H), 1.34 – 1.24 (m, 23H), 0.88 (t, *J* = 8.0 Hz, 3H); **^{13}C NMR (101 MHz, $CDCl_3$)** δ 131.63, 124.55, 57.24, 33.80, 32.58, 31.85, 29.59, 29.52, 29.41, 29.21, 29.14, 27.82, 26.59, 22.66, 17.91, 14.09; **ATR-FTIR (cm $^{-1}$):** 2927, 2925, 2855, 1645, 1465, 965, 914, 747; **HRMS m/z (ESI)** calcd for $C_{21}H_{40}O$ $(M + Na)^+$ 331.2971, found 331.2966.

(*3R,5R,8R,10S,13R,17R*)-17-((*R*)-but-3-en-2-yl)-3-methoxy-10,13-dimethylhexadecahydro-1*H*-cyclopenta[*a*]phenanthrene (54)

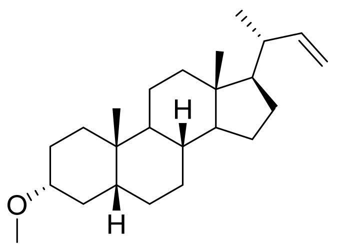

54

According to the general procedure, the reaction of tert-butyl ((*4R*)-4-((*5R,8R,10S,13R,17R*)-3-methoxy-10,13-dimethylhexadecahydro-1H-cyclopenta[*a*]phenanthren-17-yl)pentanoyl)(methyl)carbamate **53** (100.6 mg, 0.2 mmol), Ni(COD)$_2$ (5.5 mg, 10 mol %), ICy (9.3 mg, 20 mol %), KOAc (58.9 mg, 3.0 equiv), Mg(OAc)$_2$ (14.2 mg, 0.5 equiv), cyclohexane (1.0 mL) and toluene (0.5 mL) under argon at 130 ºC under Ar for 36 h after flash column chromatography on silica gel (*n*-pentane:AcOEt = 15:1) to afford 34.8 mg (51%) of **54** (the yield was 66% using 20% Ni(COD)$_2$) as a white solid: **^{1}H NMR (400 MHz, CDCl$_3$)** δ 5.65 (ddd, *J* = 17.1, 10.2, 8.4 Hz, 1H), 4.88 (ddd, *J* = 17.1, 2.0, 0.7 Hz, 1H), 4.80 (dd, *J* = 10.2, 2.0 Hz, 1H), 3.34 (s, 3H), 3.22 – 3.09 (m, 1H), 2.11 – 2.01 (m, 1H), 1.98 – 1.90 (m, 1H), 1.86 – 1.65 (m, 6H), 1.59 – 1.48 (m, 2H), 1.43 – 1.32 (m, 6H), 1.29 – 1.15 (m, 6H), 1.14 – 1.09 (m, 2H), 1.02 (d, *J* = 8.0 Hz, 3H), 0.91 (s, 3H), 0.65 (s, 3H); **^{13}C NMR (101 MHz, CDCl$_3$)** δ 145.31, 111.44, 80.38, 56.47, 55.52, 42.64, 42.01, 41.23, 40.34, 40.05, 35.81, 35.28, 34.87, 32.72, 28.46, 27.29, 26.74, 26.36, 24.21, 23.40, 20.76, 20.05, 12.18; **ATR-FTIR (cm $^{-1}$):** 3405, 3134, 2935, 2866, 1683, 1400, 1174, 1102, 995, 907, 559; **HRMS m/z (ESI)** calcd for $C_{24}H_{40}O$ (M + Na)$^+$ 367.2971, found 367.2974. The spectral data for this compound matches that reported in the literature.[22]

(*5S,8R,10S,13R,17R*)-17-((*R*)-But-3-en-2-yl)-10,13-dimethyltetradecahydro-3H-cyclopenta[*a*]phenanthrene-3,7(*4H*)-dione (56)

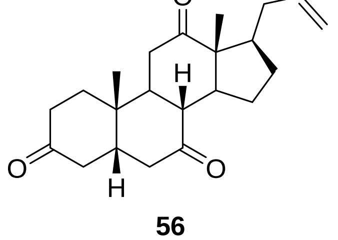

56

According to the general procedure, the reaction of tert-butyl ((*4R*)-4-((*5S,8R,10S,13R,17R*)-10,13-dimethyl-3,7-dioxohexadecahydro-1H-cyclopenta[*a*]phenanthren-17-yl)pentanoyl)(methyl)carbamate **55** (100.3 mg, 0.2 mmol), Ni(COD)$_2$ (5.5 mg, 10 mol %), ICy (9.3 mg, 20 mol %), KOAc (58.9 mg, 3.0 equiv), Mg(OAc)$_2$ (14.2 mg, 0.5 equiv), cyclohexane

(1.0 mL) and toluene (0.5 mL) under argon at 130 ºC for 36 h after flash column chromatography on silica gel (*n*-pentane:AcOEt = 5:1) to afford 31.5 mg (46%) of **56** as a white solid: **^{1}H NMR (400 MHz, $CDCl_3$)** δ 5.87 – 5.60 (m, 1H), 4.94 (d, *J* = 17.1 Hz, 1H), 4.87 (d, *J* = 10.0 Hz, 1H), 3.15 – 2.69 (m, 3H), 2.45 – 1.94 (m, 12H), 1.90 – 1.77 (m, 2H), 1.64 – 1.54 (m, 1H), 1.40 (s, 3H), 1.34 – 1.20 (m, 2H), 1.08 (s, 3H), 0.96 (d, *J* = 6.3 Hz, 3H); **^{13}C NMR (101 MHz, $CDCl_3$)** δ 211.92, 209.11, 208.78, 144.03, 112.72, 56.63, 51.47, 48.87, 46.82, 45.31, 45.13, 44.94, 42.75, 41.34, 38.55, 36.46, 35.96, 35.23, 27.31, 24.98, 21.87, 20.72, 12.10; **ATR-FTIR (cm^{-1}):** 2962, 1478, 1441, 1413, 1378, 1317, 1249, 1134, 700, 645; **HRMS m/z (ESI)** calcd for $C_{23}H_{32}O_3$ $(M + Na)^+$ 379.2244, found 379.2246.

Androstadienone (59)

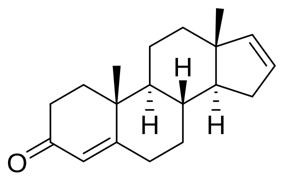

59

According to the general procedure, the reaction of *tert*-butyl ((*10R,13S*)-10,13-dimethyl-3-oxo-2,3,6,7,8,9,10,11, 12,13,14, 15,16,17-tetradecahydro-1*H*-cyclopenta[*a*]phenanthrene-17-carbonyl)(methyl)carbamate **58** (85.9 mg, 0.2 mmol), Ni(COD)$_2$ (5.5 mg, 10 mol %), ICy (9.3 mg, 20 mol %), KOAc (58.9 mg, 3.0 equiv), Mg(OAc)$_2$ (14.2 mg, 0.5 equiv), cyclohexane (1.0 mL) and toluene (0.5 mL) under argon at 130 ºC for 36 h after flash column chromatography on silica gel (*n*-pentane: AcOEt = 5:1) to afford 23.2 mg (43%) of **59** as a white solid: **^{1}H NMR (400 MHz, $CDCl_3$)** δ 5.85 (dd, *J* = 5.7, 1.6 Hz, 1H), 5.74 (s, 1H), 5.73 – 5.69 (m, 1H), 2.47 – 2.27 (m, 4H), 2.17 – 2.09 (m, 1H), 2.04 – 1.97 (m, 2H), 1.91 – 1.86 (m, 1H), 1.82 – 1.77 (m, 1H), 1.75 – 1.68 (m, 2H), 1.59 – 1.49 (m, 2H), 1.43 – 1.28 (m, 2H), 1.21 (s, 3H), 1.18 – 1.10 (m, 1H), 1.06 – 0.99 (m, 1H), 0.81 (s, 3H); **^{13}C NMR (101 MHz, $CDCl_3$)** δ 199.60, 171.40, 143.54, 129.29, 123.87, 55.42, 54.44, 45.34, 38.80, 35.58, 35.56, 34.17, 33.95, 32.86, 32.02, 31.92, 20.86, 17.22, 16.93; **ATR-FTIR (cm^{-1}):** 3405, 3134, 2852, 1678, 1616, 1401, 1229, 719, 561; **HRMS m/z (ESI)** calcd for $C_{19}H_{26}O$ $(M + Na)^+$ 293.1876, found 293.1879. The spectral data for this compound matches that reported in the literature.[23]

2-Methyl-2-vinyl-1,2,3,4-tetrahydronaphthalene (63)

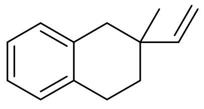

63

According to the general procedure, the reaction of *tert*-butyl methyl(3-(2-methyl-1,2,3,4-tetrahydronaphthalen-2-yl) propanoy l)carbamate **62** (66.3 mg, 0.2 mmol), $Ni(COD)_2$ (5.5 mg, 10 mol %), ICy (9.3 mg, 20 mol %), KOAc (58.9 mg, 3.0 equiv), $Mg(OAc)_2$ (14.2 mg, 0.5 equiv), cyclohexane (1.0 mL) and toluene (0.5 mL) under argon at 130 ºC for 36 h after flash column chromatography on silica gel (*n*-pentane) to afford 24.2 mg (70%) of **63** as a colourless oil: **^{1}H NMR (400 MHz, $CDCl_3$)** δ 7.18 – 7.01 (m, 4H), 5.87 (dd, *J* = 17.5, 10.8 Hz, 1H), 4.97 (dd, *J* = 9.9, 1.2 Hz, 1H), 4.93 (dd, *J* = 3.2, 1.3 Hz, 1H), 2.83 – 2.78 (m, 3H), 2.61 (d, *J* = 16.5 Hz, 1H), 1.77 – 1.70 (m, 1H), 1.69 – 1.61 (m, 1H), 1.09 (s, 3H); **^{13}C NMR (101 MHz, $CDCl_3$)** δ 146.78, 135.82, 135.74, 129.19, 128.72, 125.52, 125.46, 111.23, 40.68, 35.61, 34.26, 26.37, 26.04; **ATR-FTIR (cm^{-1}):** 3404, 3133, 2856, 1674, 1405, 1229, 708, 561; **HRMS m/z (ESI)** calcd for $C_{13}H_{16}$ $(M + Na)^+$ 195.1144, found 195.1148.

Radical Ring-Opening Experiments

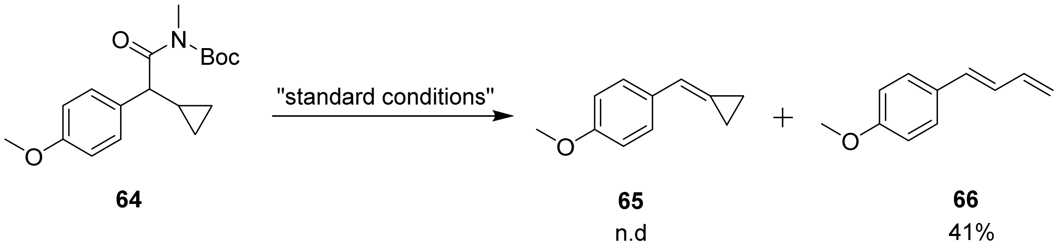

According to the general procedure, the mixture was stirred at 130 ºC under Ar for 36 hours. The solution was then cooled to room temperature, and the solvent was removed under vaccum directly. Monitored by GC-MS and H NMR, the products **65** were not observed. The crude product was purified by flash column chromatography on silica gel (*n*-pentane:AcOEt = 100:1) to afford 13.2 mg (41%) **66**[24] as a colourless oil: **^{1}H NMR (400 MHz, $CDCl_3$)** δ 7.35 (d, *J* = 8.7 Hz, 2H), 6.87 (d, *J* = 8.8 Hz, 2H), 6.72 – 6.64 (m, 1H), 6.56 – 6.51 (m, 1H), 6.53 – 6.45 (m, 1H), 5.34 – 5.24 (m, 1H), 5.18 – 5.06 (m, 1H), 3.82 (s, 3H); **^{13}C NMR (101 MHz, $CDCl_3$)** δ 159.27, 137.35, 132.38, 129.91, 127.64, 127.63, 116.41, 114.05, 55.27.

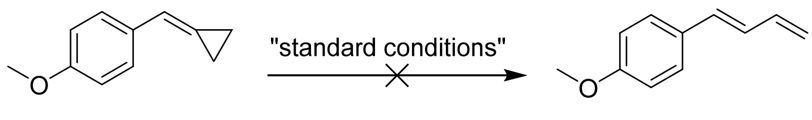

65 **66**

According to the general procedure, the mixture was stirred at 130 ºC under Ar for 36 hours. The solution was then cooled to room temperature. The products **66** were not observed by GC-MS and H NMR.

Radical Trapping Experiments

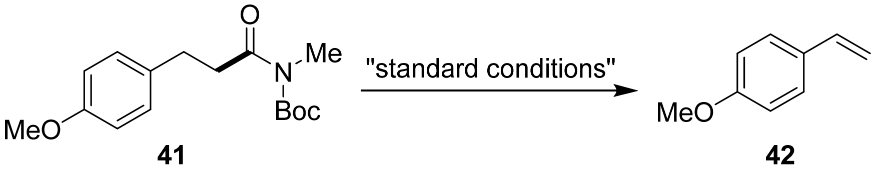

41 **42**

	**42**
	70%
with 0.2 eq TEMPO	trace
with 1.0 eq BHT	52%
with 2.0 eq BHT	28%
with 2.0 eq DHA	56%

Radical inhibitors including TEMPO, 9,10-dihydroanthracene and BHT was added in standard conditions, respectively. The product yields were determined by flash column chromatography on silica gel.

Supplementary References

1. Ortega, N., Richter, C. & Glorius, F. N-Formylation of amines by methanol activation. *Org. Lett.* **15**, 1776-1779 (2013).

2. Weires, N. A., Baker, E. L., Garg, N. K. Nickel-catalysed Suzuki-Miyaura coupling of amides. *Nat. Chem.* **8**, 75-79 (2016).

3. Lo, J. C., Yabe, Y. & Baran, P. S. A practical and catalytic reductive olefin coupling. *J. Am. Chem. Soc.* **136**, 1304-1307 (2014).

4. Movassaghi, M. & Ahmad O. K. A stereospecific palladium-catalyzed route to monoalkyldiazenes for mild allylic reduction. *Angew. Chem. Int. Ed.* **47**, 8909-8912

(2008).

5. Duchateau, R., Meerendonk, W. J., Yajjou, L., Staal, B. B. P., Koning, C. E. & Cruter, G. J. M. Ester-functionalized polycarbonates obtained by copolymerization of ester-substituted oxiranes and carbon dioxide: A MALDI-ToF-MS analysis study. *Macromolecules* **39**, 7900-7908 (2006).

6. Kadyrov, R. Low catalyst loading in ring-closing metathesis reactions. *Chem. Eur. J.* **19**, 1002-1012 (2013).

7. Wang, W., Cui, J. Q., Lu, X. X., Padakanti, P. k., Xu, J. B., Parsons, S. M., Luedtke, R. R., Rath, N. P. & Tu, Z. D. Synthesis and in vitro biological evaluation of carbonyl group-containing analogues for σ1 receptors. *J. Med. Chem.* **54**, 5362-5372 (2011).

8. Solares, L. F., Lavandera, I., Gotor-Fernandez, V., Brieva, R. & Gotor, V. Biocatalytic preparation of enantioenriched 3,4-dihydroxypiperidines and theoretical study of Candida antarctica lipase B enantioselectivity. *Tetrahedron* **62**, 3284-3291 (2006).

9. Okitsu, O., Suzuki, R. & Kobayashi, S. Efficient synthesis of piperidine derivatives. Development of metal triflate-catalyzed diastereoselective nucleophilic substitution reactions of 2-methoxy- and 2-acyloxypiperidines. *J. Org. Chem.* **66**, 809-823 (2001).

10. Schmidt, A., Nodling, A. R. & Hilt, G. An alternative mechanism for the cobalt-catalyzed isomerization of terminal alkenes to (Z)-2-alkenes. *Angew. Chem. Int. Ed.* **54**, 801-804 (2015).

11. Iwasaki, T., Miyata, Y., Akimoto, R., Fujiii, Y., Kuniyasu, H. & Kambe, N. Diarylrhodates as promising active catalysts for the arylation of vinyl ethers with Grignard reagents. *J. Am. Chem. Soc.* **136**, 9260-9263 (2014).

12. Joshi, B. P., Sharma, A. & Sinha, A. K. Ultrasound-assisted convenient synthesis of hypolipidemic active natural methoxylated (*E*)-arylalkenes and arylalkanones Tetrahedron **61**, 3075-3080 (2005).

13. Fu, S. M., Chen, N. Y., Liu, X. F., Shao, Z. H., Luo, S. P. & Liu, Q. Ligand-controlled cobalt-catalyzed transfer hydrogenation of alkynes: stereodivergent synthesis of Z- and E-alkenes. *J. Am. Chem. Soc.* **138**, 8588-8594 (2016).

14. Frlan, R., Sova, M., Gobec, S., Stavber, G. & Casar, Z. Cobalt-catalyzed cross-

coupling of grignards with allylic and vinylic bromides: use of sarcosine as a natural ligand. *J. Org. Chem.* **80**, 7803-7809 (2015).

15. Fruchey, E. R., Monks, B. M., Patterson, A. M. & Cook, S. P. Palladium-catalyzed alkyne insertion/reduction route to trisubstituted olefins. *Org. Lett.***15**, 4362-4365 (2013).

16. Krasovskiy, A. L., Haley, S., Voigtritter, K. & Lipshutz, B. H. Stereoretentive Pd-catalyzed Kumada-Corriu couplings of alkenyl halides at room temperature. *Org. Lett.* **16**, 4066-4069 (2014).

17. Rono, L. J., Yayla, H. G., Wang, D. Y., Armstrong, M. F. & Knoeles, R. R. Enantioselective photoredox catalysis enabled by proton-coupled electron transfer: development of an asymmetric aza-pinacol cyclization. *J. Am. Chem. Soc.* **135**, 17735-17735 (2013).

18. Erdogan, G. & GrotJahn, D. B. Supported imidazolylphosphine catalysts for highly (E)-selective alkene isomerization. *Org. Lett.* **16**, 2818-2821(2014).

19. Nave, S., Sonawane, R. P., Elford, T. G. & Aggarwal, V. K. Protodeboronation of tertiary boronic esters: asymmetric synthesis of tertiary alkyl stereogenic centers. *J. Am. Chem. Soc.* **132**, 17096-17098 (2010).

20. Hodgson, D. M., Fleming, M. J. & Stanway, S. J. Alkenes from terminal epoxides using lithium 2,2,6,6-tetramethylpiperidide and organolithiums or grignard reagents. *J. Am. Chem. Soc.* **126**, 12250-12251 (2004).

21. Fall, Y., Doucet, H. & Santelli, M. Palladium-catalysed suzuki cross-coupling of primary alkylboronic acids with alkenyl halides. *Appl. Organometal. Chem.* **22**, 503-509 (2008).

22. Ghavtadze, N., Melkonyan, F. S., Gulevich, A. V., Huang, C. & Gevorgyan, V. Conversion of 1-alkenes into 1,4-diols through an auxiliary-mediated formal homoallylic C-H oxidation. *Nat. Chem.* **6**, 122-125 (2016).

23. Nahar, L., Turner, A. B. & Sarker, S. D. Convenient synthesis of monomeric steroids from steroidal oxalate dimers using flash vacuum pyrolysis (FVP). *Turk. J. Chem.* **34**, 359-366 (2010).

24. Luo, S. X., Cannon, J. S., Taylor, B. L. H., Engle, K. M., Houk, K. N. & Grubbs,

R. H. Z-Selective cross-metathesis and homodimerization of 3*E*-1,3-dienes: reaction optimization, computational analysis, and synthetic applications. *J. Am. Chem. Soc.* **138**, 14039-14046 (2016).
